# From Plant Infectivity to Growth Patterns: The Role of Blue-Light Sensing in the Prokaryotic World

**DOI:** 10.3390/plants3010070

**Published:** 2014-01-27

**Authors:** Aba Losi, Carmen Mandalari, Wolfgang Gärtner

**Affiliations:** 1 Department of Physics and Earth Sciences, University of Parma, v.le G.P. Usberti 7/a, Parma I-43124, Italy; E-Mail: carmen.mandalari@fis.unipr.it; 2 Max Planck Institute for Chemical Energy Conversion, Stiftstrasse 34-36, Mülheim 45470, Germany; E-Mail: wolfgang.gaertner@cec.mpg.de

**Keywords:** LOV domain, BLUF domain, plant-pathogen interaction, phylogeny

## Abstract

Flavin-based photoreceptor proteins of the LOV (Light, Oxygen, and Voltage) and BLUF (Blue Light sensing Using Flavins) superfamilies are ubiquitous among the three life domains and are essential blue-light sensing systems, not only in plants and algae, but also in prokaryotes. Here we review their biological roles in the prokaryotic world and their evolution pathways. An unexpected large number of bacterial species possess flavin-based photosensors, amongst which are important human and plant pathogens. Still, few cases are reported where the activity of blue-light sensors could be correlated to infectivity and/or has been shown to be involved in the activation of specific genes, resulting in selective growth patterns. Metagenomics and bio-informatic analysis have only recently been initiated, but signatures are beginning to emerge that allow definition of a *bona fide* LOV or BLUF domain, aiming at better selection criteria for novel blue-light sensors. We also present here, for the first time, the phylogenetic tree for archaeal LOV domains that have reached a statistically significant number but have not at all been investigated thus far.

## 1. Introduction

**Figure 1:**
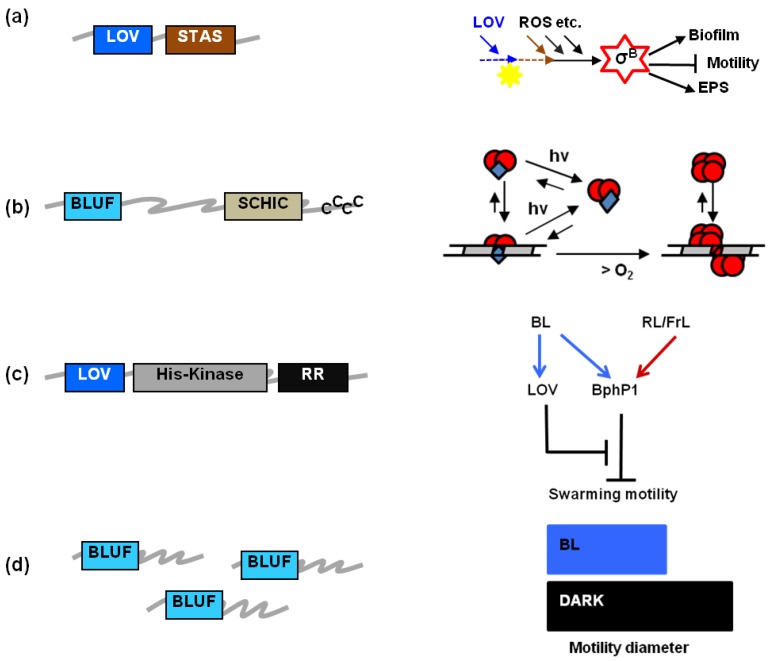
Examples of light-regulated *in vivo* effects for bacterial flavin-binding receptor (Fl-Blues), whose architecture is shown on the left: (**a**) A LOV protein from *L. monocytogenes* affects motility and growth patterns by activating the transcription factor σ^B^, acting together with other proteins and signals [1]; (**b**) AppA from *R. sphaeroides* senses BL through its BLUF domain and oxygen through its SCHIC domain; a simplified model for its action is presented on the right hand side (modified from [3]): AppA (blue squares) acts as an antirepressor for photosynthesis gene expression by binding the dimeric repressor PpsR (red circles), a complex that can also associate to DNA; illumination reduces the affinity of AppA-PpsR_2_ for DNA and PpsR_8_ can bind to its target sequence, switching off gene expression. Increased O_2_ concentration favors PpsR_8_ binding; (**c**) A LOV-kinase from *P. syringae* pv *syringae* promotes swarming motility by releasing the inhibition mediated by bacteriophytochrome 1 (BphP1) [4]; (**d**) Three similar BLUF proteins additively regulate the twitching motility of *Acinetobacter baylyi* in a light-dependent pathway [5].

A blue-light sensing, flavin-binding receptor (Fl-Blue) that regulates growth patterns and biofilm formation in *Listeria monocytogenes* (Figure 1a) [1], a single, two-cofactor Fl-Blue that integrates blue-light sensing with the detection of the cellular redox state in the facultative phototroph *Rhodobacter sphaeroides* [2,3] (Figure 1b), a third Fl-Blue that forms a signaling network with a phytochrome-related protein in regulating swarming motility of *Pseudomonas syringae* (Figure 1c) [4], and last, but not least, multiple Fl-Blue that show additive effects during light-induced inhibition of twitching motility in *Acinetobacter* *baylyi* (Figure 1d) [5]: these are only few examples of recent research achievements in the growing field of bacterial flavin-based photosensing [6], a research area, which emerged during the last decade and is rapidly changing our understanding of how prokaryotes exploit blue-light as a source of information. Most importantly, we are beginning to realize that bacterial blue-light perception integrates with other environmental cues and metabolic signals [7], e.g., temperature [8,9], redox state [10], and salt stress [11,12].

**Figure 2:**
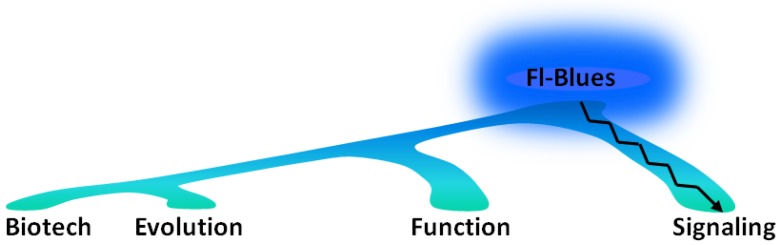
Research topics for flavin-binding, blue-light receptors (Fl-Blues). In addition to their mechanism of activation, signal propagation and signal transduction, their function *in vivo* is being actively investigated. The wide-spread occurrence of Fl-Blues in the prokaryotic world prompts investigation of their evolution pathways and phylogenetic history [6]. Flavins are ubiquitous chromophores, thus, favoring intracellular biotechnological applications, where Fl-Blues are being exploited as fluorescent reporters, photochromic proteins for nanoscopy, or photoactivable tools for optogenetics.

Fl-Blues [13] are becoming increasingly interesting proteins for a number of issues (Figure 2). Their activation and signaling mechanisms, based on non-isomerizable chromophores, have established novel paradigms in photosensory biology and photobiophysics. The biological functions of Fl-Blues have been and are still well-documented in plants, however, are now mirrored by a growing, albeit still limited, understanding of their functional roles in bacteria. The widespread occurrence of Fl-Blues offers a unique possibility of exploring the evolution and ecological significance of blue-light through soluble photoreceptors. Last, but not least, Fl-Blues have a large potential as tools for light-control of cellular functions (optogenetics) [14], and as fluorescent reporters in conditions not suitable for green-fluorescent proteins and derivatives, e.g., anaerobic/microaerobic systems or small viral genomes [15].

Fl-Blues can be classified as three distinct types: Light-Oxygen-Voltage (LOV), Blue-Light-sensing Using Flavin (BLUF), and cryptochrome (Cry) [13]. LOV and BLUF domains are relatively small and compact folds (*ca.* 100–110 aa), binding a single flavin chromophore (FMN, flavin mononucleotide, or FAD, flavin adenine dinucleotide). Their photosensing and photoresponding properties, characterized by defined photochemical reactions and spectral features (*vide infra*) are linked to diverse effector/regulator functions in a large array of different proteins [6]. Cry, instead are two-chromophore proteins, structurally and functionally related to the DNA-repairing enzymes photolyases (PL), with which they form the large Cry/PL family [16,17]. Contrary to PL, which catalyze the light-dependent repair of UV-induced DNA lesions [18], Cry proteins mainly function as sensors and have lost or show a strongly reduced capability of DNA repair activity [17]. The divergent Cry/PL superfamily [19], spread with diverse functional significance in Archaea, Bacteria, and Eukarya (including animals), is still a matter of debate as for the distinction between sensorial and photo-activated PL-like activity and will not be dealt further here. The reader is referred to recent manuscripts [17,20,21] dealing with key questions, such as: (a) the still controversially discussed photoactivation mechanisms of Cry proteins, relying on antenna chromophores and flavin-centered photoinduced electron transfer reactions; (b) the role of light-activation (e.g., in bacteria, plants, invertebrates) *versus* light-independent functions *in vivo* (e.g., in humans); and (c) the long-lasting question of Cry involvement in photo-magnetoreception in migratory birds, via the formation of spin-correlated radical pairs upon blue light excitation.

Here, we will focus on LOV and BLUF proteins, given their peculiar relevance and versatility in the prokaryotic world and their link to their plants counterparts [6,13]. The manuscript is structured as follows: a brief summary of photoactivation and light-to-signal propagation mechanisms, followed by a comprehensive update on known biological functions of LOV and BLUF proteins in bacteria. We will conclude providing novel results of our most recent survey of databases for prokaryotic LOV and BLUF domains, presenting and applying novel search criteria based on sequence patterns. This genome-mining has brought forth LOV proteins widely present in Archaea, for which we will provide for the first time a phylogenetic, distance-tree analysis of LOV domains.

### 1.1. Photoactivation of LOV and BLUF Domains

**Figure 3:**
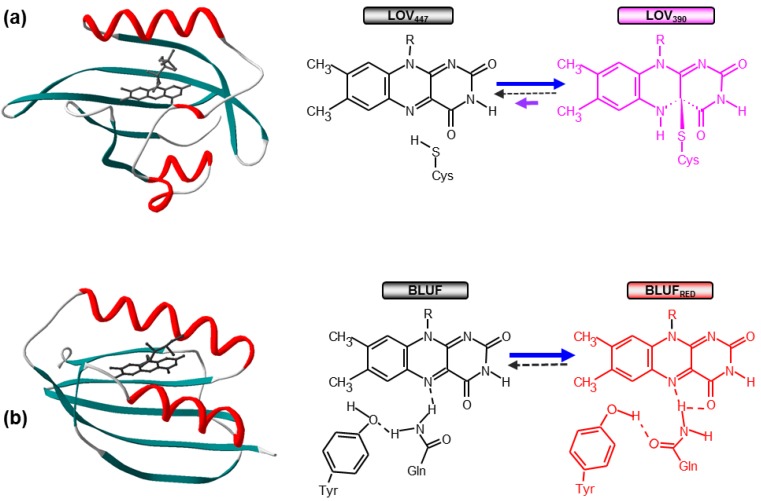
Left, three-dimensional structures of (**a**) LOV and (**b**) BLUF domains (core regions) showing the bound flavin chromophore (in black) and the secondary structure elements (red: helices, blue: strands). Right, simplified photocycles: (**a**) the blue light-induced formation of a covalent adduct for LOV domains (subscripts indicate the absorption maxima) that reverts thermally to the parental state or can partially be photoreverted with UVA/violet light [29], see [30] for a detailed description; (**b**) blue light-induced rearrangements of hydrogen bonds in BLUF domains, causing a reversible red-shift in the absorption spectrum (BLUF_RED_). This rearrangement involves a conserved Tyr-Gln couple that mediates fast (ps scale) and reversible electron and proton transfer reactions, potentially including Gln tautomeric states [33,34,35]; see [7] for a detailed discussion.

Flavins can switch between different oxidation states, referred to as “*ox*” = fully oxidized, “*sq*” = one electron reduced or semiquinonic form, “*hq*” = hydroquinone, two electron reduced form. *Sq* and *hq* can be protonated/deprotonated with pK_a_-values of *ca.* 8 and 6, respectively [22]. All these species show different absorbance and photoreactivity features in the UVB-, UVA-, and visible range. The absorption spectrum of *ox* is composed of three major ππ* bands, centered at *ca.* 446, 370 and 265 nm and characterizes the dark-adapted state of LOV and BLUF proteins (Figure 3). The *sq* species is strongly red shifted, with a maximum at *ca.* 650 nm, while *hq* species shows an unstructured spectrum with a maximum in the UVB range [21]. Upon light excitation, the redox potential dramatically shifts from ca. −0.3 V to +1.9 V, *i.e*., photoexcited flavins state are strongly oxidant. Other important photophysical properties are the high triplet quantum yield (*ca.* 0.5–0.7) of the *ox* state and its noticeable fluorescence quantum yield of 0.25–0.3 (for FMN and riboflavin) [22].

The photocycle of LOV domains starts from the dark state LOV_447_ (the subscript indicates the absorption maximum) in which *ox* FMN is non-covalently bound within an amphipatic cavity (Figure 3a). The photoprocess involves the transient formation of a covalent bond between FMN-C(4a) and a conserved cysteine (lit-state or LOV_390_), via the short μs decay of the FMN triplet state.

Formation of LOV_390_ involves the establishment of new C(4a)-S and N-H(5) bonds, suggested to proceed via the fast decay of a FMNH^•^-H_2_CS^•^ radical pair [23]. The driving force for the thermal recovery reaction to LOV_447_ is given by the high energy content of LOV_390_ (*ca.* 110–140 kJ/mol) that suggests a strained protein conformation. Nevertheless, breakage of the covalent FMN-C(4a) bond implies a high activation barrier of *ca.* 100 kJ/mol, thus, rendering the overall duration of the photocycle strongly temperature dependent and sensitive to amino acids substitutions, especially in close vicinity of the chromophore [24,25,26]. The thermal recovery rate is also highly variable among LOV domains, despite their high structural homology, ranging from few seconds to several hours around 20–25 °C [27,28]. Intriguingly, LOV domains appear to be photochromic systems, in that LOV_390_ can be photoswitched back to LOV_447_, yet with low efficiency, by using UVA/violet light [29,30,31]. The first bacterial protein for which a LOV-type photocycle has been identified, is YtvA from the soil bacterium *Bacillus subtilis* (*Bs*YtvA) [32]. 

In BLUF domains light excitation of the FAD chromophore induces a reversible red-shifted absorption spectrum, corresponding to the putative signaling state BLUF_RED_ [7]. This spectral change is dictated by a hydrogen bonds- (HB-) switch reaction involving N(5), O(4), and a conserved tyrosine-glutamine couple (Figure 3b) [33]. The lifetime for recovery of the dark-adapted state ranges from a few seconds to several minutes. BLUF_RED_ formation involves light-driven electron and proton transfer from the conserved tyrosine residue to FAD, followed by HB rearrangement and radical-pair recombination [34]. Electron transfer could also facilitate glutamine tautomerization, a mechanism that has been put forward based on theoretical calculations to account for discrepancies, in structural studies, regarding the orientation of Gln lateral chain [35]. LOV and BLUF photocycles require that the flavin chromophore is fully oxidized in the dark-adapted state, raising the possibility that these photoreceptors could be transiently light-insensitive under reducing conditions, that may occur within the cell [10,36]. The first bacterial protein for which a BLUF-type photocycle has been characterized, is AppA from the facultative phototroph *Rhodobacter sphaeroides* [37] (*Rs*AppA).

### 1.2. From Photochemistry to Signaling: Structural Issues and Signal Propagation Mechanisms

LOV and BLUF domains are minimal and compact photosensing modules (*ca.* 100–110 aa) with characteristic and well-defined α/β folds (Figure 3). The secondary structure elements of the core domains are conventionally named AβBβCαDαEαFαGβHβIβ (LOV) and β_1_α_1_β_2_β_3_α_2_β_3_ (BLUF) [27]. In LOV domains the five antiparallel β-strands form a robust β-sheet surface that anchors the flexible helical connector CαDαEαFα. The reactive cysteine is located within the Dα-Eα loop, at the *N*-terminal side of helix E. In BLUF domains the β-scaffold is also formed by five strands, partially organized in a parallel arrangement and basically running perpendicular to the isoalloxazine ring. The two Tyr and Gln residues that determine BLUF photocycle are placed on β_1_ and β_3_ respectively. Variable, mostly helical regions flank the α/β core and are often part of interfaces in oligomeric structures [13].

In full length LOV and BLUF proteins, these small and structurally well defined, globular photosensing units are linked to diverse effector domains (see Subsection 1.4 and Results and Discussion) that determine the functionality of the protein itself [6,38]; in prokaryotic Fl-Blues typical associated functions are represented by HK (histidine kinases) of the two-component systems [39], phosphatases of the SPOIIE (Sporulation stage II, protein E *C*-terminal/Protein phosphatase 2C-related) type, cyclases, and phosphodiesterases for the bacterial second messenger c-di-GMP (cyclic diguanylate) [40]. Quite frequently, DNA-binding proteins of the helix-turn-helix (HTH) type and STAS (Sulphate Transporter and AntiSigma factor antagonist) domains can be found [6]. A large number of LOV and BLUF domains are stand-alone units, where the core is solely flanked by variable helical regions (referred to as “short” LOV or BLUF). The majority of prokaryotic LOV proteins are HK, whereas most BLUF proteins are of the “short” type [41,42]. Additional sensing or regulating domains are in many cases present, especially in LOV proteins (see Supplementary Material S1 and S2 for updated lists). The photochemical events depicted in Subsection 1.1 trigger intradomain and intraprotein signal propagation, via conformational changes that travel from the chromophore cavity to molecular surfaces, ultimately affecting interdomain and/or protein-protein interactions [7,43].

The high variability and modularity of LOV and BLUF proteins still allows identification of some common mechanisms for signal propagation and -delivery. In LOV domains the extended β-sheet is a true chromophore/environment interface: on the cavity side it hosts residues directly interacting with the isoalloxazine ring of FMN, affecting chromophore stability, spectroscopic properties, and the dynamics of the photocycle [26]; at its external surface side, the β-sheet contacts helical regions flanking the LOV-core or effector domains, or is part of LOV-LOV dimerization interfaces [13]. As an example, it is now generally accepted that a conserved “flipping” glutamine (Q123 in *Bs*YtvA), located on strand Iβ and directly interacting with the chromophore, is important both in signal propagation and for the dynamics of the photocycle [12,25].

The importance of the LOV-core β-sheet during signal transmission has been first highlighted for plant phototropins (phot). Phot are light-regulated Sr/Thr-kinases bearing two LOV domains in tandem, where BL illumination triggers the unfolding and detachment of the so-called Jα-linker that connects LOV2 to the kinase domain, stimulating autophosphorylation of phot [44]. In the dark-adapted state Jα instead is mostly docked on the β-scaffold of LOV2. The light-induced unfolding of Jα presently constitutes the basis for most LOV-based optogenetic applications [14,45].

An alternative mechanism of signaling has been proposed for *Bs*YtvA that forms dimers stabilized via the LOV domain β-sheet [46,47], whereby the Jα-linker is arranged as a coiled-coil in the dimeric structure: signal transmission to a linked effector domain should proceed via a torque mechanism of the coiled Jα-linker [48]. This proposal has received strong support from the published structure of a chimeric protein, SHK YF1, bearing the LOV domain of *Bs*YtvA fused to a heterologous kinase [49]. A third variant of signal transmission in LOV proteins has been described for EL222 from *Erythrobacter litoralis*. EL222 bears a HTH motif, and is able to bind to its target only after being illuminated with BL and having undergone dimerization [50]. In the dark-adapted state, a helix involved in HTH dimerization is sequestered into interactions with the β-sheet surface of the LOV domain, and the latter also competes for the same surface of HTH devoted to DNA-binding [51]. For other LOV proteins, dimerization involving the central β-sheet surface and/or helical caps located *N*- or *C*-terminally to the LOV core, are seen as dynamic processes important for signaling, but the relevance of this phenomenon in full-length proteins is unclear [13]. Nevertheless, we can state that, as a whole, the scenario for early signal propagation and transmission steps in LOV proteins, points to a general involvement and variations of a β-sheet/helical cap mechanism on the LOV domain itself.

Signal propagation and transduction mechanisms in BLUF proteins have been recently reviewed [7,13,33]. Recent structural information on *Rs*AppA [3], combined with previous studies on *Escherichia coli* (*Ec*) YcgF, *Synechocystis Sy*PixD, *Klebsiella pneumoniae* (*Kp*), and other BLUF proteins, suggest that the β-sheet (in particular β_5_) and helices α_3_ and α_4_ (*C*-terminal to β_5_ and flanking the BLUF core) are important for signal propagation. This is reminiscent of the β-sheet/helical cap mechanism depicted above for LOV domains. In *Kp*BlrP1, BLUF is associated to an EAL domain with phosphodiesterase activity (hydrolysis of c-di-GMP) [40]. The *Kp*BlrP1-BLUF is monomeric, whereas the full-length protein is dimeric and enzymatically active, regulated via allosteric communication between the two monomers [52]. Full length *Ec*YcgF, structurally similar to *Kp*BlrP1, also exists in a fast and temperature-dependent monomer-dimer equilibrium: light excitation results in transient dimerization of the monomeric species, thought to be important for signaling [8,53].

An important concept has been acquired in recent times such that light is able to switch-on the LOV and BLUF domains, but a photoactivated photosensing domain does not necessarily correspond to an activated associated functional activity [7]. Today, we understand such processes as dynamic shifts in equilibria, e.g., the Jα-linker in the dark-adapted state of phot-LOV2 is “mostly docked” on the β-sheet surface and light activation of LOV2 shifts the equilibrium to “mostly undocked”: in other words, these kind of photoreceptors seem to possess also in the dark a constitutive activity of the associated function, *i.e.*, they are intrinsically noisy [38]. This issue is extremely important when LOV and BLUF proteins are exploited as photoswitches in optogenetics [54].

Molecular downstream partners for bacterial LOV and BLUF proteins are known only for few cases (the reader is referred to recent literature on this subject [3,7,13,33,55]). In the following subsection we focus instead on biological roles of prokaryotic LOV and BLUF proteins.

### 1.3. The Significance of Prokaryotic LOV and BLUF Proteins *in Vivo*

It is not yet possible to delineate a general concept about the physiological roles of LOV and BLUF proteins in bacteria: genome mining (*vide infra*) and structural/functional studies *in vitro* proceed obviously much faster that photophysiological investigations, but some basic ideas are starting to emerge. BL seems to affect, through LOV and BLUF proteins, certain growth and metabolic patterns that imply differential gene expression, with relevance to complex phenomena, such as cell adhesion and biofilm formation. A special attention is presently devoted to the role of these photoreceptors in animal and plant pathogens, but also in environmental and symbiotic bacteria. It is also emerging that in many cases Fl-Blues have both light-dependent and light-independent roles, and some examples will be given below.

In *Bacillus subtilis* the LOV protein YtvA is a constitutive part of the stressosome, a large molecular hub that integrates environmental stimuli and initiates a signaling cascade, ultimately up-regulating the alternative transcription factor sigmaB (σ^B^) [56]. σ^B^ is one of the key components in the general stress response (GSR) and controls the transcription of 150–200 genes in *B. subtilis* [57]. In transformant cells overexpressing YtvA, GSR is readily induced by BL, but in WT *B. subtilis* the effect is more moderate and BL plays a role as stressor only if associated to an additional salt stress [11]. Mutagenesis studies *in vivo* have identified residues responsible for light-to-signal transduction [12] and a quantitative model has recently been built, correlating the photocycle dynamics *in vitro* with the efficiency of BL effects *in vivo* [31]. Nevertheless, it is clear that YtvA also can exert light-independent effects: *B. subtilis* cells overexpressing YtvA show an enhanced activation of σ^B^, even when YtvA is mutated at the Cys62 position, the residue essential for a functional photocyle; Cys62 is nevertheless required for the BL-induced response in the absence of another stress [58]. For the related bacterium *Bacillus* *amyloliquefaciens* BL inhibition of antifungal lipopeptide synthesis has been reported [59], but this observation has not yet been linked to the YtvA-like photoreceptor [60] of this species.

The important animal pathogen *Listeria monocytogenes* also bears a YtvA-like photoreceptor, Lmo0799, which up-regulates σ^B^ in response to BL and is also involved in red light-driven up-regulation of σ^B^ via a still unknown mechanism [61,62]. In addition, in this pathogen, full induction of genes controlled by σ^B^ requires both BL and salt stress stimuli, with an only partial and weaker effect of BL alone. Very recently, a spectacular colony differentiation mediated by Lmo0799 and coordinated by σ^B^ has been described [1]. Alternating dark-light periods result in the formation of opaque and translucent rings on agar plates. Opaque rings contain bacteria with an increased amount of EPS (extracellular polymeric substances) and with a better survival rate towards hydrogen peroxide, whose level also has a direct effect on Lmo0799 expression. This has led to the hypothesis that bacteria in the opaque rings are more resistant against stress mediated by ROS (reactive oxygen species): as ROS are induced within the cell under light condition, the photosensing activity of Lmo0799 is apparently integrated to the ROS-mediated activation of σ^B^. Additionally, BL activation of Lmo0799 inhibits motility via an antisense RNA, whereas the photoreceptor is not involved in σ^B^-coordinated biofilm formation [1]. Importantly, in *L. monocytogenes*, light alone is able to elicit the stress responses, whereas in *B.* *subtilis* overexpression of YtvA or additional salt stress is required [11]. Despite the fact that Lmo0799, *in vitro*, is thermally quite unstable and starts to lose FMN above 26 °C [62], BL-induced activation of σ^B^ and σ^B^-controlled transcription of stress genes, still occurs at 37 °C [1]. Instead, BL effects on flagellar motility and hence on pattern formation/colony morphology can be observed at temperatures of 23–27 °C only because at higher temperatures (e.g., 37 °C) the transcription of flagellar genes is inhibited [1], *i.e*., BL and temperature cross-talk seems to be, at least for the case of Lmo0799, only apparent (*vide infra* for reported examples of BL and temperature interplay). BL activation of Lmo0799 also results in enhanced invasiveness towards enterocytes, pointing to some role of this photoreceptor during infectivity, although not yet fully clarified and established [61]. One should keep in mind that *Listeria* also represent a major threat to humans, as, e.g., listeriosis has the highest mortality rate of all food-borne infections, therefore, these studies could be extremely important for food industry and human health.

In addition, *L. monocytogenes* prokaryotic LOV proteins are present in a large number of animal pathogens and they are considered to be involved in infectivity (see Supplementary Material S1) by influencing bacterial lifestyle. The first break-through publication about the physiological role of a bacterial LOV protein in a human pathogen was published in 2007, when Swartz *et al*. demonstrated that, in *Brucella abortus*, blue light stimulates infection of host cells through a LOV-HK [63], although the detailed mechanism of this phenomenon is not known. Other bacteria important for human health for which Fl-Blues have been demonstrated to play a physiological role are opportunistic pathogens of the genus *Acinetobacter*, microorganisms that exhibit surface-associated motility (e.g., twitching), regulated by blue-light via BLUF proteins. The first report came from Mussi *et al*., who discovered that cells of *Acinetobacter baumannii* formed spreading colonies in the dark but non-spreading colonies under blue-light: absence of the short-BLUF protein BlsA removed this inhibitory effect of blue-light [9]. This effect of BLUF-based blue-light sensing has also been reported for several other *Acinetobacter* species [64]. Accordingly, three short-BLUF proteins act together in inhibiting twitching motility in *Acinetobacter baylyi ADP1*, thus, also suggesting a possible redundant role for multiple photoreceptors of the same type [5] (*vide infra*). Additional effects mediated by BLUF proteins comprise an increased infectivity towards *Candida albicans* cells for *Acinetobacter baumannii* and inhibition of biofilm formation under blue-light [9]. Biofilm formation is instead stimulated by BLUF proteins in *A. baylyi ADP1* and other *Acinetobacter* strains [64]. Biofilm maturation is also promoted in *E. coli* by the photoreceptor YcgF, a BLUF photoreceptor according to the signaling pathway outlined in Section 1.2 [53]. The YcgF-YcgE system (photoreceptor-repressor) integrates BL and other stress signals, being induced at low temperature and under starvation conditions. It is not yet clear which is the mechanism of cross-talk with temperature, namely temperature dependence of gene expression [53], or employing a monomer-dimer equilibrium of YcgF, as was also proposed [8].

During the last two years, investigations have been initiated also on plant pathogens aiming to identify a possible influence of bacterial photoreceptors (often resembling plant counterparts, such as phototropins and phytochromes), on responses related to plant-pathogen interactions, such as motility, adhesion to leaves, infectivity, and virulence: is the bacterial pathogen able to sense the same light environment as a host plant and to adapt accordingly its lifestyle? Some answers to this intriguing question are beginning to emerge (see Table 1). A thorough study on the impact of a LOV-HK on growth, selected metabolic pathways, motility, and virulence, of *Xanthomonas axonopodis* pv*. citri* (*Xac*), an important plant pathogen, has been recently published [65]. The deletion mutant *Xac∆lov* shows important differences to *Xac*WT, indicating that the LOV-HK protein participates in the formation of flagella and survival under oxidative stress; *Xac∆lov* has a larger swarming motility, altered twitching motility and increased production of extracellular polysaccharide (EPS): these different behavioral properties are nevertheless not influenced by light. Biofilm formation is also altered, being initially impaired and at a later stage promoted in *Xac∆lov*, a time-scaling possibly related to the impaired flagella synthesis and concomitant larger production of EPS. Most importantly, light regulates bacterial adhesion and virulence via the *Xac*LOV protein: (i) cell adhesion is positively controlled by light, but it is strongly impaired in the deletion mutant, although light-control is not cancelled, suggesting the participation of other photoreceptors (*Xac* possesses also two BLUF and a Bph1-like protein) [6]; (ii) In darkness, *Xac*WT and *Xac∆lov* provoke similar plant necrotic lesions, but under light-conditions *Xac*WT exerts a protective effect against necrosis: extensive necrosis is observed upon infection with *Xac∆lov*, whereas infection with *Xac*WT induces canker, but a negligible level of necrosis. As a whole, *Xac*LOV has been demonstrated to participate in some bacterial growth patterns and metabolic pathways, to confer resistance against oxidative stress and to control virulence towards *citrus* [65]. In the related bacterium *Xanthomonas campestris* pv*. campestris*, bacterial growth was studied under different light conditions. No dark-light effects on the growth of WT bacteria were observed, whereas a deletion mutant of the LOV-HK protein exhibits a much smaller growth rate under BL [66]. *Xac*LOV seems instead not to influence growth of *X. axonopodis* pv*. citri*.

Similar aspects came forward from studies conducted on another agronomically important pathogen, *Pseudomonas syringae* pv. *tomato* (*Pst*). The *Pst*LOV kinase inhibits bacterial growth and the expression of genes controlled by principal and alternative sigma-factors (e.g., regulation of exponential growth, general stress response, secondary metabolite production *etc*.) [67]. Most importantly, *Pst*LOV seems to mediate BL-driven reduction of virulence in infected plants [67,68], and a concomitant increased bacterial adhesion to leaves [68] (as in *X. axonopodis* pv*. citri*, see above). This might be related to a reduced invasiveness and proliferation within leaves, mediated by BL via this photoreceptor [67], possibly via a strongly reduced number of flagella [68]. In *P. syringae* pv. *syringae* (*Pss*) the effects of light on swarming motility have been studied in greater detail, as a LOV-HK protein and the two bacteriophytochromes (BphP1 and BphP2) are present in this organism. A striking result is a signaling network between LOV-HK and BphP1, where the latter acts as a negative regulator of swarming motility in response to both blue and red/far-red light. In the BL region, though, LOV-HK positively regulates swarming motility, by suppressing the inhibiting effects of BphP1 [4]. These results obviously imply that BphP1 is also a sensor for BL, besides red/far-red light. Note that in *Pst*, BL seems instead to inhibit swarming motility via the LOV-HK protein, although the effects of BphP1 have not yet been reported for this organism [6]. With this respect, it is important to note that BL has an inhibiting effect on *Pst* virulence towards plant leaves, while the opposite is true for red light, suggesting a role of BphPs in this organism as well [68].

As a whole, the results up to now available for LOV proteins in plant pathogens of the genus *Xanthomonas* and *Pseudomonas* indicate that, upon BL activation, they regulate motility and bacterial growth patterns, promote bacterial adhesion to plant leaves, and inhibit certain aspects of virulence. The cross-talk with other photosensors is still largely unexplored, but is likely to play an important role both for bacterial lifestyle and virulence [4,65,68].

Plant-bacteria interaction are also important for symbiotic relationships: in the nitrogen-fixing *Rhizobium leguminosarum* a photoactivated LOV-HK protein inhibits EPS and biofilm formation and flagella production, and it reduces bacterial proliferation within plant roots. Conversely the number of competent (N_2_ fixing) root nodules in host plants is increased: the biological advantages of these modifications induced by BL are not clear, but they might be important to optimize infection and N_2_ fixation at different soil level [69]. BL-dependent inhibition of biofilm formation via the short-BLUF protein PapB, has also been reported for the purple bacterium *Rhodopseudomonas palustris* [70]. A BL-regulated motility response, *i.e*., positive phototaxis in *Synechocystis* sp. PCC6803, has been shown to be dependent on the short-BLUF protein PixD (also Slr1694), acting together with other, still unidentified, photoreceptors [33] (see Table 1 and Table 2).

**Table 1:** Known photobiological effects mediated by Light, Oxygen, and Voltage (LOV)-proteins in bacteria.

	LOV protein	Blue light-regulated phenomena
*B. subtilis*	*Bs*YtvA	Activation of σ^B^ stress factor ^a^ [11,12,31,56]
*B. amyloliquefaciens*	YtvA-like?	Antifungal lipopeptide synthesis ^a^ [59]
*L. monocytogenes*	Lmo0799 (YtvA-like)	σ^B^–mediated invasiveness, swimming motility, salt stress ^a^ [61]; colony differentiation [1]
*R. leguminosarum*	LOV-HK	Biofilm; EPS ^b^; flagella; proliferation within roots; competence for N_2_ fixing [69]
*C. crescentus*	LovK	Cell-cell adhesion [71,72] ^c^
*E. litoralis*	EL222	Binding to DNA consensus sequences [73]
*B. abortus*	LOV-HK	Infectivity [63]
*X. axonopodis* pv*. citri*	*Xac*LOV	Adhesion; virulence [65]
*X. campestris* pv*. campestris*		Growth [66]
*P. syringae* pv*. tomato*	*Pst*LOV	Growth; motility (swarming); adhesion; virulence; invasiveness; σ factor gene expression [67,68]
*P. syringae* pv*. syringae*	*Pss*LOV	Motility (swarming) ^d^ [4]
*R. sphaeroides*	*Rs*LOV	Genes for photosynthesis and photo-oxidative stress under σ^F^ control [74] ^e^

^a^: additionally, red-light effects have been described; ^b^: EPS = exopolysaccharides; ^c^: co-regulation by cell-redox state; ^d^: signaling network with bacteriophytochrome 1 (BphP1) protein; ^e^: possible signaling network with AppA.

**Table 2:** Known photobiological effects by Blue Light sensing Using Flavins (BLUF)-proteins in bacteria.

	BLUF protein	Blue light-regulated phenomena
*R. sphaeroides*	*Rs*AppA	Photosynthesis gene transcription; [37]; integration of light and redox sensing ^a^ [3,75,76]
*Synechocystis* sp. PCC6803	PixD/Slr1694	Phototaxis [33]
*Acinetobacter*	BLUF	Surface motility; biofilm [64]
*A. baumannii*	BLUF	Virulence; surface motility; biofilm ^b^ [9]
*A. baylyi ADP1*	BLUF	Surface motility; biofilm ^b^ [5,64]
*R. palustris*	PapB	Biofilm [70]
*E. coli*	YcgF	Biofilm ^b^ [53,77]

^a^: Possible signaling network with CryB [78] and a LOV protein[69]; ^b^: co-regulation by temperature.

Another relatively well-characterized organism is the free living bacterium *Caulobacter crescentus*. Here, a two-component system formed by a LOV-HK (LovK) and its cognate response regulator LovR has been identified as a negative regulator of the general stress pathway, under the control of the σ^T^ factor [72]. This negative regulation of transcription during the general stress pathway seems to be light-independent, but genetic and biochemical data suggest that the photosensory LovK-LovR two-component system positively regulates a cellular attachment factor in response to BL, possibly related to food availability at different depths of the water column [71]. It is not clear how the described light-independent and BL-dependent responses integrate using the same two-component system. According to the authors one possible explanation is the fact that LovK photochemistry is conditioned by the cell redox state: the redox potential of a living cell is close to the midpoint potentials measured for LOV- and BLUF-bound flavins, *i.e*., −250/300 mV [10,36], which might cause electrochemical reduction of the flavin and renders the photochemistry impossible as a fully oxidized chromophore is necessary [13].

Finally we refer to *Rhodobacter sphaeroides*, a paradigmatic example of how BL sensing relies on diverse Fl-Blues, interplaying with each other and being connected to other environmental stimuli. *R. sphaeroides* can switch between photosynthesis and aerobic respiration via a complex regulation mechanism that includes the BLUF protein *Rs*AppA (Figure 1d). As outlined in the figure, AppA binds the dimeric repressor PpsR constitutively at low oxygen tension, whereas under fully aerobic conditions PpsR_2_ is released from AppA and binds to the promoter of certain photosynthesis genes, repressing their transcription [79]. These responses are light-independent, but at intermediate oxygen concentration, BL reduces the affinity of AppA for PpsR_2_ that can now bind to its target DNA sequence [3]. Thus, *Rs*AppA integrates both BL and redox signaling [75]. Redox sensing relies chiefly on the heme binding SCHIC (Sensor Containing Heme Instead of Cobalamin) domain, possibly involving a *C*-terminal cysteine-rich sequence [2,80] and the photoactive flavin bound within the BLUF domain [36]. Recently the *Rs*LOV protein has been shown to act synergistically with *Rs*AppA and to down-regulate the expression of genes involved in photosynthesis and in photo-oxidative stress response under control of the σ^F^ factor [74]. As a further complication, an interaction between *Rs*AppA and a Cry protein has been demonstrated [78]. Apparently, the activity of these systems in *R. sphaeroides* is aimed to maximize photosynthesis under favorable conditions and to reduce it if the risk of photooxidative damage is high (see Table 2).

### 1.4. Distribution and Evolutionary Patterns of Prokaryotic LOV and BLUF Domains

Thus far, we have reported on examples where effects of LOV- and BLUF-domain containing proteins have been identified, either via their physiological properties that are monitored through their signaling domain-in many cases an easily controllable enzyme activity, or via their impact on their host organisms in case these bacteria are identified as plant or animal/human pathogens. In nearly all these cases, Fl-Blues with canonical sequence motifs were investigated. As the routine approach for, e.g., host pathogen interactions are to be studied, genomes of the pathogens are screened for the, thus far, well-known signatures of their light sensing domains. The upcoming of more and more genomic sequence information, however, calls for more sophisticated and, thus, routinely handable search criteria. Thus, despite the fact that the enormous increase of genomic information causes more laborious investigations, the large number of bacteria sequenced offers also the potential to extract and to deduce more powerful search motifs. We, thus, dedicate the following section to the description of new search parameters and demonstrate their capability by presenting a distance tree for all so far available archaeal genomes.

Distance trees and selection criteria for *bona fide* prokaryotic LOV and BLUF domains have been reported several times and were again generated recently [6]. The worldwide distribution of such photosensing units has been proven to hold also in metagenomes [81,82,83]. By the help of, here presented, new search criteria, we can now demonstrate the wide distribution of LOV proteins in archaea, with a still completely unexplored relevance, given that the majority of photosensory research with these microorganisms has been devoted to the prevalent opsin-based receptors [84].

In the following Results and Discussion section, we report novel results of database mining for prokaryotic Fl-Blues by using sequence logos and search patterns that significantly facilitate the search. A few novel functions associated to photosensing domains have been uncovered. The quite high number of archaeal LOV proteins permits now to identify two distinct superfamilies within this superkingdom and to highlight notable differences with bacteria, as for functional distributions. The construction of a distance-tree, presented here for the first time with reference to archaeal LOV domains, indicates that for archaeal LOV domains clustering occurs mainly according to groups, and not in relation to putative molecular functions, that is, instead, a prominent feature for bacteria [6].

## 2. Results and Discussion

### 2.1. The Sequence Logos and Searching Patterns

**Figure 4:**
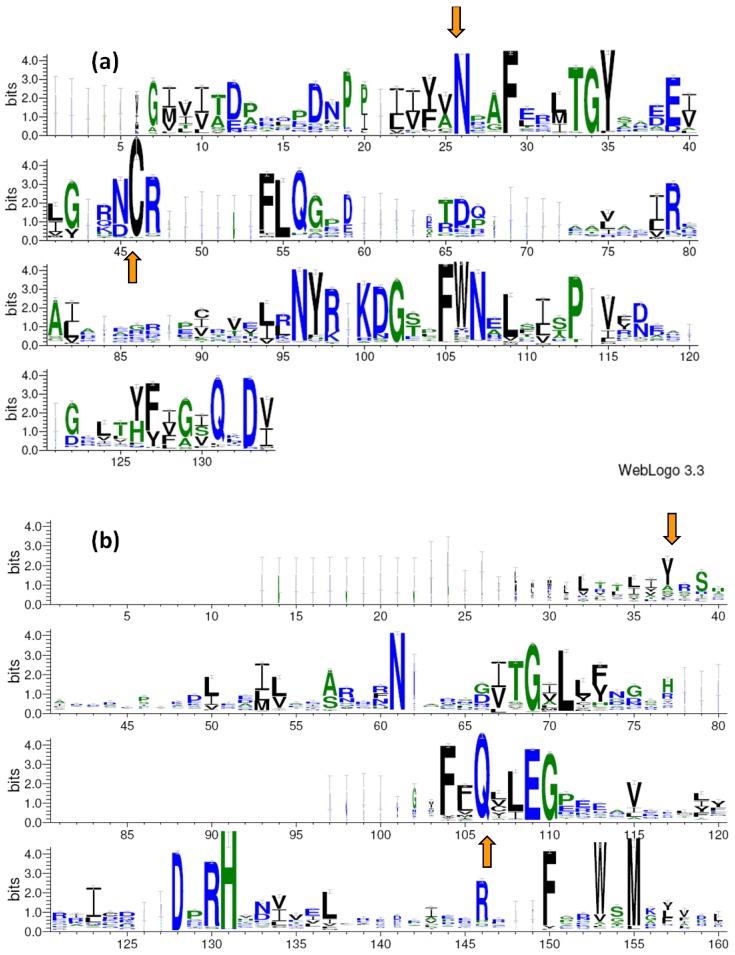
Amino acids sequence logos [85,86] for (**a**) LOV and (**b**) BLUF domains. The vertical axis gives the value of Shannon entropy expressed in bits, the horizontal axis lists the aa number of the primary structure. Colors of the one-letter codes are related to the hydrophobicity: blue, hydrophilic aa (R, K, D, E, N, Q) green, neutral (S, G, H, T, A, P), black, hydrophobic (Y, V, M, C, L, F, I, W). Orange arrows mark the beginning and end of the interval used to define the subsequent search patterns.

Surveys in databases yield so-called sequence logos [85,86], obtained from an alignment of *ca.* 2000 protein sequences (eukaryotic and prokaryotic), for each of the two photosensing domains (Figure 4). It is evident that, beyond the essential presence of amino acids functional to the photocycle, other residues are highly conserved. From these residues we built sequence patterns that highly facilitated the subsequent search [87] for prokaryotic LOV and BLUF domains (see Experimental Section for details). The patterns we used for LOV domains was: [NS]-*x*(2)-[FPGHSA]-*x*(4)-[GEQR]-*x*(9,11)-C, where *x* is any amino acid and the terminal C is the cysteine forming the covalent bond during the photocycle. For BLUF domains we used: Y-*x*(21,27)-[NG]-*x*(8,9)-[LMVIFTK]-*x*(6,14)-[FALIYCV]-*x*(1)-Q, where the two residues at the *N*- and *C*-terminal ends are the Tyr-Gln reactive pair. To avoid exceedingly stringent criteria, which would miss some hits, we extended in some positions the choice among amino acids similar to those super-conserved. The length of variable intervals is obtained from alignment and from structural considerations of secondary structure elements.

**Figure 5:**
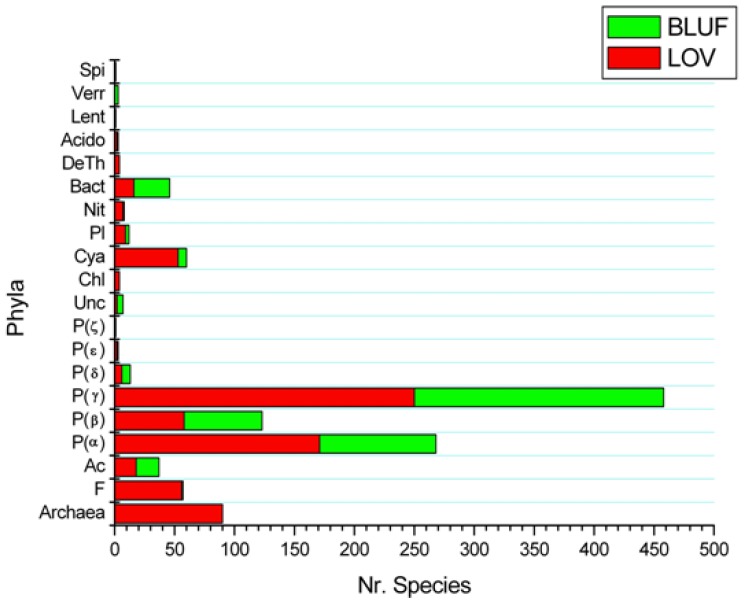
Histogram of distribution for species bearing at least one LOV (red) or/and one BLUF (green) protein in the diverse phyla (for proteobacteria also classes are given). Spi = Spirochaetes; Verr = Verrucomicrobia; Lent = Lentisphaerae; Acido = Acidobacteria; DeTh = Deinococcus-Thermus; Bact = Bacteroidetes/Chlorobi; Nit = Nitrospirae; Pl = Planctomycetes; Cya = Cyanobacteria; Chl = Chloroflexi; Unc = Uncultured; P = Proteobacteria; Ac =Actinobacteria; F = Firmicutes; see Supplementary Material S4 for a detailed list.

With this algorithm we identified 1390 LOV proteins in 1031 bacterial strains (658 species), 167 archaeal LOV proteins in 86 strains (82 species), and 1705 bacterial BLUF proteins in 1282 strains (453 species; many strains are from *E. coli*, *Acinetobacter* and *K. pneumoniae*). The results are summarized in a histogram (Figure 5), where one can observe the distribution of LOV and BLUF in different phyla and classes. For detailed information on orders and different strains of the same species see Supplementary Material S1, S2, and S3.

### 2.2. LOV and BLUF Domains in Bacteria

The LOV and BLUF scenario for superkingdom bacteria has not changed much with respect to our last analysis [6]. Of the 1390 LOV proteins detected, *ca.* 50% are HK of the two-component systems [39], in some cases built with additional sensing and regulatory domains. PAS domains (Per Arnt Sim), the superfamily to which LOV domains belong [88] are often present, possibly with a regulative function or further sensing function. About 18% bacterial LOV proteins bear GGDEF or GGDEF+EAL domains that act as cyclases and phosphodiesterases for c-di-GMP formation or hydrolysis [40]. Short-LOV proteins are present in about the same percentage, whereas 8.5% of the proteins are YtvA-like, solely found in the Firmicutes. Only eight proteins carry a HTH motif for DNA-binding, in HTH+LOV or LOV+HTH arrangements, configuring one-component systems [89]. In addition, 20 proteins are one-component systems where a LOV domain is directly linked to a response regulator (RR).

BLUF proteins are still dominated by short-proteins (64%) and by the large occurrence of EAL domains (34%), putatively involved in c-di-GMP metabolism, sometimes these domains are degenerated and enzymatically inactive EAL domains, as illustrated above for YcgF in *E. coli* [53] (Supplementary Material S2). A notable new entry is a BLUF protein found in *Enterococcus gallinarum*, the first of this type in a member of the phylum Firmicutes.

In 162 species/strains both LOV and BLUF proteins are present. Some major examples of bacteria literally “packed” with LOV and BLUF proteins are given in Table 3. There are presently no hints about the molecular functioning of a possible BL-sensing network in these organisms (with the exception of *R. sphaeroides*, see Table 1) outlined. We recall that the largest co-presence of soluble photoreceptors in bacteria is given by the LOV and bilin-GAF superfamilies [6].

**Table 3:** Examples of bacteria having multiple LOV and BLUF proteins in co-presence.

	Class (phylum)/order	LOV proteins	BLUF proteins
*Aureimonas ureilytica*	P(α)/Rhizobiales	2 × LOV-HK	2 × short-BLUF
*Methylobacterium populi* ^a^	P(α)/Rhizobiales	7 × LOV-HK 1 × short LOV	2 × short-BLUF
*Sphingobium xenophagum*	P(α)/Sphingomonadales	2 × LOV-RR 1 × LOV-HK	2 × short-BLUF
			
*Herminiimonas arsenicoxydans*	P(β)/Burkholderiales	3 × LOV-GGDEF/EAL	1 × short-BLUF
*Methylomicrobium alcaliphilum*	P(γ)/Methylococcales	2 × short LOV 1 × LOV-HK	3 × short-BLUF 1 × BLUF-GGDEF/EAL

^a^: all bacteria of the genus *Methylobacterium* are rich in LOV, BLUF and bilin-binding domains (see Supplementary Material S1 and S2).

### 2.3. LOV in Archaea: DNA-Binding Proteins and Kinases

**Figure 6:**
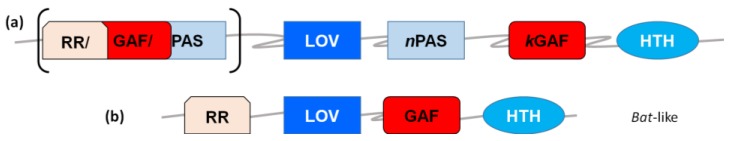
(**a**) General architecture for archaeal LOV-HTH proteins: in most cases a photosensing LOV domain is preceded by a response regulator (RR), a GAF or a PAS domain, and followed by a variable number of PAS (*n* = 0–8) and GAF (*k* = 0–2) domains; at the *C*-terminus of the protein, there is a DNA-binding domain of the helix-turn-helix (HTH) type; (**b**) One of the arrangements detected resembles the *Bat* protein of *Halobacterium salinarum*, a redox-sensing transcription regulator that has been reported to mediate production of bacteriorhodopsin in the purple membrane [91]. *H. salinarum Bat* has been described as a putative light-sensor, but it does not bear the cysteine involved in the photocycle of LOV domains.

Application of above outlined search criteria yielded 167 archaeal LOV proteins in 85 species (Supplementary Material S3) of Euryarchaeota, belonging to the two classes of *Halobacteria* and, screened for the first time, *Methanomicrobia*. The most striking difference between archaeal and bacterial LOV proteins is that a large percentage (30%) of the halobacterial protagonists carry fused HTH effector domains (all from *Halobacteria*), whereas this motif is found in only 0.6% of all bacteria. The general architecture of these putative photo-regulated DNA-binding proteins is depicted in Figure 6 and can be generalized as (RR/GAF/ or PAS)+LOV+*n*PAS+*k*GAF+HTH, with *n* = 0–8, and *k* = 0–2, where: RR = response regulator, receiver domain; PAS = Per Arnt Sim domain (the same fold as LOV domains); GAF = domain found in cGMP-specific phosphodiesterases, cyanobacterial adenylate cyclases, and formate hydrogen lyase transcription activator FhlA [90] (see Supplementary Material S4 for a detailed legend). One of the detected arrangements closely resembles the *Bat* protein of *Halobacterium salinarum*, a redox- and putatively light-sensing transcription regulator that mediates production of bacteriorhodopsin in the purple membrane [91]. *H. salinarum* Bat bears a PAS domain lacking the reactive cysteine of canonical LOV proteins. The remaining archaeal proteins are LOV-kinases of the two-component systems [39], with solely RR, GAF, and PAS domains as additional building units. We note that none of the archaeal LOV proteins have been investigated so far.

**Figure 7:**
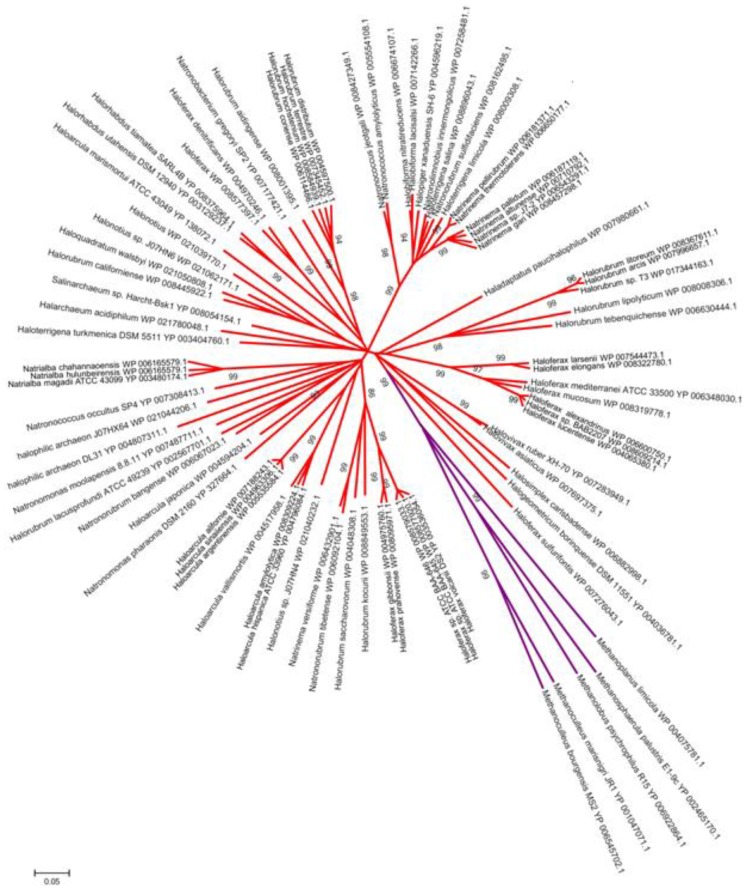
Unrooted distance tree for archaeal LOV domains, obtained with the neighboring-joining algorithm. On the nodes we report the bootstrap values >80; red branches are related to *Halobacteria*; purple lines mark *Methanomicrobia* that cluster separately as a class.

The distance-tree built with archaeal LOV domains (Figure 7) again depicts a scenario different from the bacterial ones, for which major deteminants of group clustering were the effector/regulative domains associated to the photosensing units [6]. In the case of archaea, LOV domains of *Methanomicrobia* cluster separately from *Halobacteria*, emphasizing the difference in metabolism and habitats for these two classes. It is still too early, nevertheless, to draw any stringent conclusion on the evolutionary history of LOV domains in archaea, given that up to now only two classes are represented.

## 3. Experimental

To build the sequence logos of Figure 4, prokaryotic and eukaryotic candidates for LOV and BLUF domains were searched by using the BLAST (Basic Local Alignment Search Tool) network service at the National Center for Biotechnology Information (NCBI) [87]. The LOV core of *B. subtilis* YtvA and the BLUF domain of tll0078 (TePixJ) from *Thermosynechococcus elongatus* strain BP-1, respectively, served as seed sequences, as previously described [6]. From the recovered sequences a sequence logo was built for the two domains, employing 2,000 sequences in each case and removing those without aligned functional amino acids, by using WebLogo [86] and Shannon’s entropy to quantify the uncertainty of positions [85] (Figure 5). From Logo we defined two patterns in Prosite language [90], in order to obtain a signature that may facilitate the BLAST search. The two patterns, that were then used to search for prokaryotic LOV and BLUF domains are: [NS]-*x*(2)-[FPGHSA]-*x*(4)-[GEQR]-*x*(9,11)-C for LOV domains; Y-*x*(21,27)-[NG]-*x*(8,9)-[LMVIFTK]-*x*(6,14)-[FALIYCV]-*x*(1)-Q for BLUF domains, where *x* is any amino acid. With these pattern we performed a search for LOV and BLUF domains over all annotated prokaryotic genome sequences (completed or in progress), within the non-redundant protein sequence database and the SwissProt/UniProt Knowledgebase [92], at the time point of 31 October 2013, and using 10 as threshold for the PHI-BLAST algorithm.

Alignment of the recovered sequences was performed with Clustal W using BLOSUM as a protein weight matrix without negative values [93]. Distance-trees were built based on the obtained alignments, employing MEGA5 [94] according to the neighbor-joining. The resulting tree is unrooted and does not provide information on a common ancestor for all sequences. Furthermore, the paths between internal and external nodes are not related to the evolutionary time, not yielding a temporal resolution, but only revealing information on changes of protein sequences and on their putative functions.

Protein domains were detected by using the InterProScan network service at the European Bioinformatics Institute (EBI) [95]. Detailed results, protein accession codes and domain analysis are reported in Supplementary Materials S1, S2, and S3.

## 4. Conclusions

Photophysiological studies of BL-sensing in prokaryots, as mediated by non-membrane LOV and BLUF proteins, are just now starting to accumulate. Only the demonstration of light-regulated changes in the growth behavior and the infectivity of selected animal and plant pathogens that can cause deleterious social and economic impact slowly convinces (micro)biologists of the important role of BL-sensing photoreceptors. As far as can be deduced thus far, BL seems to operate both as a stress factor and as a source of environmental information able to elicit adaptative responses in metabolism, growth patterns, and interactions with other organisms. It is still a fully open and fascinating question, if and how these Fl-Blues exert an effect in the large number of radiotolerant, extremophilic, and xenobiotic-metabolizing bacteria that are prominently represented in our lists of LOV- and BLUF-carrying prokaryots. The ever-growing information of genomic information now calls for more elaborate tools to screen new genomes (and metagenomes) for the presence of BL-photoreceptor genes and the identification of their genomic neighborhood. Though, still in its infancy, search criteria are now being developed that allow a more sophisticated search for potential photoreceptors, and also allow suggesting scenarios by which BL-photoreceptors have an impact on the gene regulation. The usefulness of such search criteria has been demonstrated here for the first time for archaea in which a manifold of BL-sensing photoreceptors could be identified. None of these archaeal species have been probed so far for their response to and lifestyle changes caused by blue light. It has now to be accepted that the generic stress response mechanisms, well studied in several bacterial species, have now to be extended by the impact elicited by blue light.

## Supplementary Files

Supplementary File 1
