# Supplementary material for: From Plant Infectivity to Growth Patterns: The Role of Blue-Light Sensing in the Prokaryotic World
Source: Plants (Basel). 2014 Jan 27;3(1):70–94. doi: 10.3390/plants3010070 (PMC4844311; doi:10.3390/plants3010070)
Supplement: Supplementary File 1 [file plants-03-00070-s001.zip › plants-46379-supplementary-final/Supplementary Material - S1 - LOV bacteria.pdf]

Bacterial LOV domains In yellow: species/strain also possessing BLUF domains (see S2)

Nr.	Species/strain	Group	Accession nr. Uniprot	Accession nr. NCBI	aa nr.	Domains	Prot. Nr.
1	*Amphibacillus jilinensis*	F		WP_017473228.1	262	LOV+STAS	1
2	*Aneurinibacillus aneurinilyticus*	F		WP_021620417.1	491	PAS+LOV+SPOIIE	2
3	*Bacillus amyloliquefaciens*	F		WP_017418405.1	276	LOV+STAS	3
	Bacillus amyloliquefaciens ATCC 23350	F	E1UNE3	YP_003921412.1	261	LOV+STAS	4
	Bacillus amyloliquefaciens CAU-B946	F	H2ABK8	YP_005131472.1	261	LOV+STAS	5
	Bacillus amyloliquefaciens FZB42	F	A7Z7V9	YP_001422316.1	261	LOV+STAS	6
4	*Bacillus amyloliquefaciens subsp. plantarum AS43.3*	F	L0BRW8	YP_007187501.1	261	LOV+STAS	7
	Bacillus amyloliquefaciens subsp. plantarum NAU-B3	F		CDH96675.1	261	LOV+STAS	8
	Bacillus amyloliquefaciens subsp. plantarum UCMB5033	F	S6G2H0	YP_008413843.1	276	LOV+STAS	9
	Bacillus amyloliquefaciens subsp. plantarum UCMB5036	F	M1XEM3	YP_007498348.1	261	LOV+STAS	10
	Bacillus amyloliquefaciens subsp. plantarum UCMB5113	F	S6FTN1	YP_008422089.1	276	LOV+STAS	11
	Bacillus amyloliquefaciens subsp. plantarum YAU B9601-Y2	F	H8XFK1	YP_005422280.1	276	LOV+STAS	12
	Bacillus amyloliquefaciens TA208	F	F4E8V5	YP_005542621.1/YP_003921412.1	261	LOV+STAS	13
	Bacillus amyloliquefaciens XH7	F	G0IGZ5	YP_005551014.1/YP_003921412.1	261	LOV+STAS	14
	Bacillus amyloliquefaciens (Bacillus velezensis)	F	F4EJR7	YP_005546869.1/YP_003921412.1	261	LOV+STAS	15
5	*Bacillus atrophaeus*	F	R0MMM0	WP_010789563.1	273	LOV+STAS	16
	Bacillus atrophaeus str. 1942	F	E3E2E8	YP_003974446.1	263	LOV+STAS	17
6	*Bacillus endophyticus*	F		WP_019392897.1	259	LOV+STAS	18
7	*Bacillus macauensis ZFHKF-1*	F	I8AIW7	WP_007201639.1	263	LOV+STAS	19
8	*Bacillus megaterium*	F		WP_016764751.1	260	LOV+STAS	20
	Bacillus megaterium ATCC 12872	F	D5DX18	YP_003563516.1	260	LOV+STAS	21
	Bacillus megaterium DSM 319	F	D5DGT4	YP_003598275.1	214	LOV+STAS	22
	Bacillus megaterium WSH-002	F	G2RIW6	YP_005494307.1	260	LOV+STAS	23
9	*Bacillus mojavensis*	F		WP_010331556.1	261	LOV+STAS	24
10	*Bacillus pumilus*	F		WP_019743696.1	267	LOV+STAS	25
				WP_017367889.1	267	LOV+STAS	26
				WP_019743575.1	262	LOV+STAS	27
				WP_017368190.1	262	LOV+STAS	28
	Bacillus pumilus ATCC 7061	F	B4ANP6	WP_003217570.1	267	LOV+STAS	29
			B4AJ24	WP_003214834.1/YP_001488493.1	262	LOV+STAS	30
	Bacillus pumilus SAFR-032	F	A8FGG2	YP_001487889.1	267	LOV+STAS	31
			A8FI66	YP_001488493.1	262	LOV+STAS	32
11	*Bacillus selenitireducens ATCC 700615*	F	D6XXH5	YP_003698598.1	256	LOV+STAS	33
12	*Bacillus siamensis*	F		WP_016938511.1	261	LOV+STAS	34
13	*Bacillus sp. 5B6*	F	I2HUE6	WP_007613218.1	261	LOV+STAS	35
14	*Bacillus sp. HYC-10*	F	K2NQI4	WP_008354841.1	262	LOV+STAS	36
			K2NB57	WP_008356458.1	279	LOV+STAS	37
15	*Bacillus sp. JS*	F	I0F7W3	YP_006232825.1	261	LOV+STAS	38
16	*Bacillus sp. M 2-6*	F	I4VFF5	WP_008343003.1	267	LOV+STAS	39
			I4VEQ1	WP_008343295.1	262	LOV+STAS	40
17	*Bacillus stratosphericus LAMA 585*	F	M5RFF6	WP_007501884.1	267	LOV+STAS	41
			M5R3V3	WP_007497556.1	262	LOV+STAS	42
18	*Bacillus subtilis*	F		WP_019259236.1	261	LOV+STAS	43
				WP_019715441.1	261	LOV+STAS	44
	Bacillus subtilis BSn5	F	E8VHV4	YP_004204857.1	261	LOV+STAS	45
19	*Bacillus subtilis subsp. inaquosorum KCTC 13429*	F	L8Q4F3	WP_003238003.1	261	LOV+STAS	46
20	*Bacillus subtilis subsp. natto BEST195*	F	D4G0B4	YP_005562248.1	261	LOV+STAS	47
21	*Bacillus subtilis subsp. spizizenii ATCC 23059*	F	E0TXF2	YP_003867287.1	261	LOV+STAS	48
	Bacillus subtilis subsp. spizizenii ATCC 6633	F	D5N263	WP_003223298.1/YP_003867287.1	261	LOV+STAS	49

	Bacillus subtilis subsp. spizizenii TU-B-10	**F**	G4NZQ8	YP_004878519.1	261	LOV+STAS	50
22	*Bacillus subtilis subsp. subtilis RO-NN-1*	**F**	G4P3K6	YP_005558004.1	261	LOV+STAS	51
	Bacillus subtilis subsp. subtilis str. 168	**F**	O34627-YtvA	NP_390912.1	261	LOV+STAS	52
	Bacillus subtilis subsp. subtilis str. BSP1	**F**	L0D031	YP_007208463.1	261	LOV+STAS	53
	Bacillus subtilis subsp. subtilis str. SC-8	**F**	G4ES72	WP_003229169.1	261	LOV+STAS	54
	Bacillus subtilis XF-1	**F**	M4KWC9	YP_007427967.1	261	LOV+STAS	55
23	*Bacillus vallismortis*	**F**		WP_010329536.1	261	LOV+STAS	56
24	*Bhargavaea cecembensis DSE10*	**F**	M7NUV9	WP_008300509.1	259	LOV+STAS	57
25	*Clostridium tyrobutyricum*	**F**		WP_017750870.1	666	PAS+LOV+PAS+Kinase+RR	58
26	*Halobacillus halophilus ATCC 35676*	**F**	I0JHP4	YP_006178939.1	266	LOV+STAS	59
27	*Halobacillus sp. BAB-2008*	**F**	L5N866	WP_008636934.1	268	LOV+STAS	60
28	*Listeria grayi DSM 20601*	**F**	D7UXG5	WP_003758096.1	257	LOV+STAS	61
29	*Listeria innocua ATCC 33091*	**F**	H1GBU2	WP_003770995.1	266	LOV+STAS	62
	Listeria innocua Clip11262	**F**	Q92DM1	NP_470134.1	253	LOV+STAS	63
	Listeria innocua FSL J1-023	**F**	E3Z5Q4	WP_003765959.1	253	LOV+STAS	64
	Listeria innocua FSL S4-378	**F**	E3YX19	WP_003761128.1/NP_470134.1	253	LOV+STAS	65
30	*Listeria ivanovii ATCC BAA-678*	**F**	G2ZDA1	YP_004854533.1	253	LOV+STAS	66
31	*Listeria marthii FSL S4-120*	**F**	E3YNA7	WP_008947200.1	253	LOV+STAS	67
32	*Listeria monocytogenes*	**F**	L8DRI2	NP_464326.1	253	LOV+STAS	68
	Listeria monocytogenes 07PF0776	**F**	I0CPV0	YP_013419.1	253	LOV+STAS	69
	Listeria monocytogenes 10403S	**F**	G2K4H2	YP_005961931.1/NP_464326.1	253	LOV+STAS	70
	Listeria monocytogenes 4b H7858	**F**	Q4EEF6	WP_003726661.1/YP_013419.1	253	LOV+STAS	71
	Listeria monocytogenes ATCC 19117	**F**	J7PNH7	YP_013419.1	253	LOV+STAS	72
	Listeria monocytogenes EGD-e	**F**	P58724	NP_464326.1	253	LOV+STAS	73
	Listeria monocytogenes F2365	**F**	Q722B8	YP_013419.1	253	LOV+STAS	74
	Listeria monocytogenes F6854	**F**	Q4EN15	EAL05103.1/NP_464326.1	253	LOV+STAS	75
	Listeria monocytogenes F6900	**F**	C8KA37	EEW21003.1/NP_464326.1	253	LOV+STAS	76
	Listeria monocytogenes Finland 1998	**F**	G2KIU9	YP_005970621.1/NP_464326.1	253	LOV+STAS	77
	Listeria monocytogenes FSL F2-208	**F**	E3YE39	WP_003735967.1	253	LOV+STAS	78
	Listeria monocytogenes FSL J1-194	**F**	D4PI54	WP_003726937.1/YP_013419.1	253	LOV+STAS	79
	Listeria monocytogenes FSL J1-208	**F**	H7CIX6	WP_003738433.1	253	LOV+STAS	80
	Listeria monocytogenes FSL J2-071	**F**	D3KP16	EFD90245.1/YP_002350789.1	253	LOV+STAS	81
	Listeria monocytogenes FSL N1-017	**F**	D7UJQ9	EFK41291.1/YP_013419.1	253	LOV+STAS	82
	Listeria monocytogenes FSL N3-165	**F**	C8JWU1	EEW14621.1/NP_464326.1	253	LOV+STAS	83
	Listeria monocytogenes FSL R2-503	**F**	C8K567	EEW19771.1/YP_013419.1	253	LOV+STAS	84
	Listeria monocytogenes FSL R2-561	**F**	G2K6K4	YP_005967717.1/NP_464326.1	253	LOV+STAS	85
	Listeria monocytogenes HPB2262	**F**	D4Q0W9	EFF94760.1/YP_013419.1	253	LOV+STAS	86
	Listeria monocytogenes J0161	**F**	G2JVW6	YP_005964793.1/NP_464326.1	253	LOV+STAS	87
	Listeria monocytogenes J1-220	**F**	F3RIF4	YP_008289002.1/YP_013419.1	253	LOV+STAS	88
	Listeria monocytogenes J1816	**F**	F3RC23	YP_008285930.1/YP_013419.1	253	LOV+STAS	89
	Listeria monocytogenes J2818	**F**	D4PRY3	EFF97759.1/NP_464326.1	253	LOV+STAS	90
	Listeria monocytogenes L312	**F**	J7P482	YP_013419.1	253	LOV+STAS	91
	Listeria monocytogenes N53-1	**F**	L8E287	NP_464326.1	253	LOV+STAS	92
33	*Listeria monocytogenes serotype 1/2a 08-5578*	**F**	D2P049	YP_003412995.1/NP_464326.1	253	LOV+STAS	93
	Listeria monocytogenes serotype 1/2a 08-5923	**F**	D2PB38	YP_003416040.1/NP_464326.1	253	LOV+STAS	94
34	*Listeria monocytogenes serotype 4a HCC23*	**F**	B8DGG7	YP_002350789.1	253	LOV+STAS	95
	Listeria monocytogenes serotype 4a M7	**F**	F8BE06	YP_005946888.1/YP_002350789.1	253	LOV+STAS	96
35	*Listeria monocytogenes serotype 4b Clip81459*	**F**	C1L168	YP_002757524.1/YP_013419.1	252	LOV+STAS	97
	Listeria monocytogenes serotype 4b str. LL195	**F**	K8FAX5	YP_007014391.1	302	LOV+STAS	98
36	*Listeria monocytogenes serotype 4c L99*	**F**	E1UEF6	YP_005925797.1/YP_002350789.1	253	LOV+STAS	99
37	*Listeria monocytogenes serotype 7 str. SLCC2482*	**F**	J7MIR4	YP_013419.1	253	LOV+STAS	100
	Listeria monocytogenes SLCC2372	**F**	J7NJ10	NP_464326.1	253	LOV+STAS	101
	Listeria monocytogenes SLCC2376	**F**	J7P7Y6	YP_002350789.1	253	LOV+STAS	102

	Listeria monocytogenes SLCC2378	**F**	J7P7K7	YP_013419.1	253	LOV+STAS	103
	Listeria monocytogenes SLCC2479	**F**	J7N224	NP_464326.1	253	LOV+STAS	104
	Listeria monocytogenes SLCC2540	**F**	J7PFP1	YP_013419.1	253	LOV+STAS	105
	Listeria monocytogenes SLCC2755	**F**	J7N0R0	YP_013419.1	253	LOV+STAS	106
	Listeria monocytogenes SLCC5850	**F**	J7MN47	NP_464326.1	253	LOV+STAS	107
	Listeria monocytogenes SLCC7179	**F**	J7NYX0	NP_464326.1	253	LOV+STAS	108
	Listeria monocytogenes str. Scott A	**F**	F3YSH3	EGJ24299.1/YP_013419.1	253	LOV+STAS	109
38	*Listeria seeligeri FSL N1-067*	**F**	E3ZND1	WP_003746290.1	266	LOV+STAS	110
	Listeria seeligeri FSL S4-171	**F**	E3ZX98	WP_003751585.1	253	LOV+STAS	111
39	*Listeria seeligeri serovar 1/2b ATCC 35967*	**F**	D3UL64	YP_003463935.1	253	LOV+STAS	112
40	*Listeria welshimeri serovar 6b str. SLCC5334*	**F**	A0AGP3	YP_848958.1	253	LOV+STAS	113
41	*Oceanobacillus iheyensis HTE831*	**F**	Q8ESN8	NP_691509.1	264	LOV+STAS	114
42	*Oceanobacillus kimchii*	**F**		WP_017795669.1	264	LOV+STAS	115
43	*Paenibacillus fonticola*	**F**		WP_019637272.1	580	LOV+GGDEF+EAL	116
44	*Paenibacillus polymyxa (strain E681)*	**F**	E0REH5	YP_003868921.1	825	GAF+LOV+LOV+SPOIIE	117
45	*Paenibacillus sp. A9*	**F**		WP_017813224.1	548	PAS+LOV+Kinase	118
46	*Paenibacillus sp. PAMC 26794*	**F**		WP_017687351.1	382	LOV+Kinase	119
47	*Paenibacillus terrae (strain HPL-003)*	**F**	G7W1W6	YP_005074888.1	367	LOV+Kinase	120
48	*Paenisporosarcina sp. TG-14*	**F**		WP_017381979.1	730	LOV+GAF+GGDEF+EAL	121
49	*Paenisporosarcina sp. TG20*	**F**		WP_019413525.1	735	LOV+GAF+GGDEF+EAL	122
50	*Planococcus antarcticus DSM 14505*	**F**	I4X763	WP_006829023.1	280	LOV+STAS	123
51	*Planococcus donghaensis MPA1U2*	**F**	E7RG70	WP_008430240.1	282	LOV+STAS	124
52	*Planococcus halocryophilus Or1*	**F**	M3F5S5	WP_008498406.1	292	LOV+STAS	125
53	*Saccharibacillus kuerlensis*	**F**		WP_018975129.1	385	LOV+Kinase	126
54	*Salimicrobium sp. MJ3*	**F**	K2GL47	WP_008591203.1	269	LOV+STAS	127
55	*Virgibacillus halodenitrificans*	**F**		WP_019378944.1	258	LOV+STAS	128
56	*Virgibacillus sp. CM-4*	**F**	T0JJ18	WP_021291946.1	263	LOV+STAS	129
57	*Actinomycetospora chiangmaiensis*	**Ac**		WP_018335315.1	715	PAS+LOV+GAF+SPOIIE	130
58	*Arthrobacter aurescens TC1*	**Ac**	A1RDS2	YP_950384.1	727	LOV+ GGDEF+EAL	131
59	*Arthrobacter sp. TB 23*	**Ac**		WP_019481926.1	681	LOV+GGDEF+EAL	132
60	*Beutenbergia cavernae DSM 12333*	**Ac**	C5BXY5	YP_002880641.1	580	LOV+GAF+SPOIIE	133
61	*Blastococcus saxobsidens DD2*	**Ac**	H6RWC2	YP_005329368.1	762	LOV+GGDEF+EAL	134
62	*Cellulomonas fimi DSM 20113*	**Ac**	F4H5H4	YP_004455284.1	594	PAS+LOV+SPOIIE	135
63	*Cellulomonas flavigena DSM 20109*	**Ac**	D5UEA1	YP_003638776.1	570	LOV+GAF+SPOIIE	136
64	*Cellulomonas sp. JC225*	**Ac**		WP_019135604.1	564	LOV+GAF+SPOIIE	137
65	*Frankia sp. BCU110501*	**Ac**		WP_018504846.1	664	LOV+GAF+SPOIIE	138
66	*Frankia sp. CN3*	**Ac**	G6HCZ9	WP_007513898.1	603	LOV+GAF+SPOIIE	139
67	*Frankia sp. str. Eul1c*	**Ac**	E3J501	YP_004015322.1	628	LOV+GAF+SPOIIE	140
68	*Geodermatophilus obscurus DSM 43160*	**Ac**	D2S7Y1	YP_003411932.1	760	PAS+LOV+GAF+SPOIIE	141
69	*Kineococcus radiotolerans SRS30216*	**Ac**	A6WG47	YP_001364050.1	743	GGDEF+LOV+EAL	142
			A6W4U8	YP_001360101.1	723	LOV+GAF+SPOIIE	143
			A6W4X7	YP_001360130.1	408	LOV+SPOIIE	144
70	*Leifsonia rubra CMS 76R*	**Ac**	S7W0I9	WP_021809600.1	653	LOV+GGDEF+EAL	145
71	*Modestobacter marinus BC501*	**Ac**	I4EZ22	YP_006367116.1	750	EAL+DICT+LOV+GGDEF	146
	Modestobacter marinus BC502	**Ac**	I4F5I6	YP_006369358.1	776	PAS+LOV+GAF+SPOIIE	147
72	*Nakamurella multipartita DSM 44233*	**Ac**	C8XJT7	YP_003203637.1	365	PAS+RR+LOV	148
			C8X922	YP_003204109.1	149	Short-LOV	149
73	*Rubrobacter radiotolerans*	**Ac**	E9REA7	BAJ78459.1	85	Short-LOV	150
74	*Rubrobacter xylanophilus DSM 9941*	**Ac**	Q1ARZ9	YP_645641.1	581	LOV+GAF+SPOIIE	151
75	*Agrobacterium albertimagni AOL15*	**P(α)**	K2Q1D4	WP_006728201.1	375	LOV+Kinase	152
76	*Agrobacterium sp. 10MFCol1.1*	**P(α)**		WP_019566834.1	589	LOV+GGDEF+EAL	153
77	*Agrobacterium sp. 224MFTsu3.1*	**P(α)**		WP_020010289.1	369	LOV+Kinase	154
78	*Agrobacterium sp. H13-3*	**P(α)**	F0L3W1	YP_004278691.1	369	LOV+Kinase	155

			F0LEX8	YP_004444410.1	589	LOV+GGDEF+EAL	156
79	*Agrobacterium tumefaciens 5A*	P(α)	H0HAA7	WP_003515223.1	369	LOV+Kinase	157
			H0HGN3	WP_003518335.1	589	LOV+GGDEF+EAL	158
80	*Agrobacterium vitis (strain S4 / ATCC BAA-846)*	P(α)	B9K197	YP_002547361.1	411	LOV+ Kinase	159
81	*Ahrensia sp. R2A130*	P(α)	E0MQF6	WP_009757882.1	325	LOV+Kinase	160
82	*alpha proteobacterium JLT2015*	**P(α)Unc**	M2U2Y4	WP_008602941.1	345	LOV+Kinase	161
83	*alpha proteobacterium L41A*	**P(α)Unc**		WP_017504522.1	364	LOV+Kinase	162
84	*alpha proteobacterium LLX12A*	**P(α)Unc**		WP_017503590.1	200	Short-LOV	163
				WP_017502221.1	866	LOV+PAS+GAF+Kinase	164
85	*Amorphus coralli*	P(α)		WP_018698921.1	773	PAS+LOV+GGDEF+EAL	165
86	*Asticcacaulis excentricus (strain ATCC 15261)*	P(α)	E8RN55	YP_004087039.1	596	LOV+Kinase+RR	166
87	*Aureimonas ureilytica*	P(α)		WP_019998669.1	306	LOV+Kinase	167
				WP_019997685.1	346	LOV+Kinase	168
				WP_019995998.1	406	LOV+Kinase	169
88	*Bradyrhizobium oligotrophicum S58*	P(α)	M4ZKM0	YP_007510402.1	534	LOV+Kinase+RR	170
89	*Bradyrhizobium sp. BTAi1*	P(α)	A5EAE7	YP_001237047.1	534	LOV+Kinase+RR	171
90	*Bradyrhizobium sp. ORS278*	P(α)	A4Z279	YP_001208470.1	534	LOV+Kinase+RR	172
91	*Bradyrhizobium sp. ORS 285*	P(α)	H0RX89	WP_006611907.1	534	LOV+Kinase+RR	173
			H0RX89	WP_006611907.1	534	LOV+Kinase+RR	174
92	*Bradyrhizobium sp. ORS 375*	P(α)	H0SK82	WP_009029541.1	534	LOV+Kinase+RR	175
93	*Bradyrhizobium sp. STM 3809*	P(α)	H0SYI7	WP_008961860.1	534	LOV+Kinase+RR	176
94	*Brevundimonas sp. BAL3*	P(α)	B4WCV5	WP_008259253.1	878	LOV+PAS+GAF+Kinase+RR	177
95	*Brucella abortus (strain 2308 A)*	P(α)	C4IVN9	WP_006016110.1/WP_002970660.1	496	LOV+PAS+ Kinase	178
	Brucella abortus (strain 2308)	P(α)	Q2YKK7	NP_541657.1	463	LOV+PAS+ Kinase	179
	Brucella abortus 01-0065	P(α)	S3WQE0	NP_541657.1	489	LOV+Kinase	180
	Brucella abortus 01-0585	P(α)	S3VF14	NP_541657.1	489	LOV+Kinase	181
	Brucella abortus 01-0648	P(α)	S3P9P5	NP_541657.1	489	LOV+Kinase	182
	Brucella abortus 225/65	P(α)	N7SDC2	NP_541657.1	489	LOV+Kinase	183
	Brucella abortus 355/78	P(α)	N7SLZ0	NP_541657.1	489	LOV+Kinase	184
	Brucella abortus 544	P(α)	N7SWJ8	NP_541657.1	489	LOV+Kinase	185
	Brucella abortus 600/64	P(α)	N7RYR9	NP_541657.1	489	LOV+Kinase	186
	Brucella abortus 63/130	P(α)	N7SCI3	NP_541657.1	489	LOV+Kinase	187
	Brucella abortus 63/138	P(α)	N7TSI7	NP_541657.1	489	LOV+Kinase	188
	Brucella abortus 63/144	P(α)	N7TUX2	NP_541657.1	489	LOV+Kinase	189
	Brucella abortus 63/168	P(α)	N7U9C9	NP_541657.1	489	LOV+Kinase	190
	Brucella abortus 63/294	P(α)	N7TE92	WP_006091747.1	489	LOV+Kinase	191
	Brucella abortus 63/59	P(α)	N7A0H8	NP_541657.1	489	LOV+Kinase	192
	Brucella abortus 64/108	P(α)	N7TIY1	NP_541657.1	489	LOV+Kinase	193
	Brucella abortus 64/122	P(α)	N7A7E6	NP_541657.1	489	LOV+Kinase	194
	Brucella abortus 64/81	P(α)	N7UVS8	NP_541657.1	489	LOV+Kinase	195
	Brucella abortus 65/110	P(α)	N7AJ59	NP_541657.1	489	LOV+Kinase	196
	Brucella abortus 65/157	P(α)	N7U3Y2	NP_541657.1	489	LOV+Kinase	197
	Brucella abortus 65/63	P(α)	N7VHX7	NP_541657.1	489	LOV+Kinase	198
	Brucella abortus 67/781	P(α)	N7ASN3	NP_541657.1	489	LOV+Kinase	199
	Brucella abortus 67/93	P(α)	N7VRX4	NP_541657.1	489	LOV+Kinase	200
	Brucella abortus 68-3396P	P(α)	S3TMU2	NP_541657.1	489	LOV+Kinase	201
	Brucella abortus 76-1413	P(α)	S3RZI8	NP_541657.1	489	LOV+Kinase	202
	Brucella abortus 78/14	P(α)	N7US78	NP_541657.1	489	LOV+Kinase	203
	Brucella abortus 78/32	P(α)	N7WH17	NP_541657.1	489	LOV+Kinase	204
	Brucella abortus 78/36	P(α)	N7A0T6	NP_541657.1	489	LOV+Kinase	205
	Brucella abortus 80/101	P(α)	N7VBV9	NP_541657.1	489	LOV+Kinase	206
	Brucella abortus 80/102	P(α)	N7BFC1	NP_541657.1	489	LOV+Kinase	207
	Brucella abortus 80/108	P(α)	N7BQ67	NP_541657.1	489	LOV+Kinase	208

	Brucella abortus 80/28	P(α)	N7WPT0	NP_541657.1	489	LOV+Kinase	209
	Brucella abortus 80-1399	P(α)	S3RXL3	NP_541657.1	489	LOV+Kinase	210
	Brucella abortus 82-2330	P(α)	S3SEP8	NP_541657.1	489	LOV+Kinase	211
	Brucella abortus 82-3893	P(α)	S3RYB1	NP_541657.1	489	LOV+Kinase	212
	Brucella abortus 84/26	P(α)	N7VRB7	NP_541657.1	489	LOV+Kinase	213
	Brucella abortus 84-0928	P(α)	S3RC75	NP_541657.1	489	LOV+Kinase	214
	Brucella abortus 85/140	P(α)	N7BTA4	NP_541657.1	489	LOV+Kinase	215
	Brucella abortus 85/69	P(α)	N7W4L9	NP_541657.1	489	LOV+Kinase	216
	Brucella abortus 85-1058	P(α)	S3X8D7	NP_541657.1	489	LOV+Kinase	217
	Brucella abortus 863/67	P(α)	N7C384	NP_541657.1	489	LOV+Kinase	218
	Brucella abortus 87/28	P(α)	N7XPS1	NP_541657.1	489	LOV+Kinase	219
	Brucella abortus 87-0095	P(α)	S3W4M5	NP_541657.1	489	LOV+Kinase	220
	Brucella abortus 87-2211	P(α)	S3VXQ7	NP_541657.1	489	LOV+Kinase	221
	Brucella abortus 877/67	P(α)	N7XME5	NP_541657.1	489	LOV+Kinase	222
	Brucella abortus 88/19	P(α)	N7CBP8	NP_541657.1	489	LOV+Kinase	223
	Brucella abortus 88/217	P(α)	N7WYJ1	WP_006091747.1	489	LOV+Kinase	224
	Brucella abortus 88/226	P(α)	N7BPT2	NP_541657.1	489	LOV+Kinase	225
	Brucella abortus 89-0363	P(α)	S3R3J0	NP_541657.1	489	LOV+Kinase	226
	Brucella abortus 90/50	P(α)	N7BZI6	NP_541657.1	489	LOV+Kinase	227
	Brucella abortus 90-0737	P(α)	S3Q4A1	NP_541657.1	489	LOV+Kinase	228
	Brucella abortus 90-0742	P(α)	S3RJ59	NP_541657.1	489	LOV+Kinase	229
	Brucella abortus 90-0775	P(α)	S3QCW3	NP_541657.1	489	LOV+Kinase	230
	Brucella abortus 90-0962	P(α)	S3R6U3	NP_541657.1	489	LOV+Kinase	231
	Brucella abortus 90-1280	P(α)	S3Q5W1	NP_541657.1	489	LOV+Kinase	232
	Brucella abortus 93/1	P(α)	N7D2H4	NP_541657.1	489	LOV+Kinase	233
	Brucella abortus 93/2	P(α)	R8VWD1	NP_541657.1	489	LOV+Kinase	234
	Brucella abortus 94-1313	P(α)	S3QH15	NP_541657.1	489	LOV+Kinase	235
	Brucella abortus A13334	P(α)	G8T504	YP_005114240.1/YP_223408.1	458	LOV+PAS+Kinase	236
	Brucella abortus B10-0018	P(α)	S3NW52	NP_541657.1	489	LOV+Kinase	237
	Brucella abortus B10-0091	P(α)	S3PQB4	NP_541657.1	489	LOV+Kinase	238
	Brucella abortus B10-0973	P(α)	S3PCT0	NP_541657.1	489	LOV+Kinase	239
96	*Brucella abortus biovar 1 str. 9-941*	P(α)	Q577Y7	YP_223408.1	458	LOV+PAS+ Kinase	240
	Brucella abortus bv. 1 str. NI010	P(α)	H3QEE3	EHR17495.1/NP_541657.1	489	LOV+Kinase+PAS	241
	Brucella abortus bv. 1 str. NI016	P(α)	H3QN03	EHR18337.1/NP_541657.1	489	LOV+Kinase+PAS	242
	Brucella abortus bv. 1 str. NI021	P(α)	H3QX28	EHR26941.1/NP_541657.1	489	LOV+Kinase+PAS	243
	Brucella abortus bv. 1 str. NI259	P(α)	H3R3M0	EHR27594.1/NP_541657.1	489	LOV+Kinase+PAS	244
	Brucella abortus bv. 1 str. NI435a	P(α)	H3PCZ9	EHR09267.1/NP_541657.1	489	LOV+Kinase+PAS	245
	Brucella abortus bv. 1 str. NI474	P(α)	H3PNQ1	EHR07958.1/NP_541657.1	489	LOV+Kinase+PAS	246
	Brucella abortus bv. 1 str. NI486	P(α)	H3PTS7	EHR11756.1/NP_541657.1	489	LOV+Kinase+PAS	247
	Brucella abortus bv. 1 str. NI488	P(α)	H3PZJ2	EHR26142.1/NP_541657.1	489	LOV+Kinase+PAS	248
97	*Brucella abortus bv. 2 str.86/8/59*	P(α)	C9V2E2	WP_002966045.1	456	LOV+PAS+Kinase	249
98	*Brucella abortus bv. 3 str. Tulya*	P(α)	C9UTV3	WP_002966045.1	456	LOV+PAS+Kinase	250
99	*Brucella abortus bv. 4 str.292*	P(α)	C9UJF2	WP_002967295.1	179	Short-LOV	251
100	*Brucella abortus bv. 5 str. B3196*	P(α)	D7H767	EFH32827.1/NP_541657.1	489	LOV+PAS+Kinase	252
101	*Brucella abortus bv. 6 str.870*	P(α)	C9U9U0	WP_002966045.1	456	LOV+PAS+Kinase	253
102	*Brucella abortus bv. 9 str.C68*	P(α)	C9VPJ9	WP_002966045.1	456	LOV+PAS+Kinase	254
	Brucella abortus CNGB 1011	P(α)	N7DAK7	NP_541657.1	489	LOV+Kinase	255
	Brucella abortus CNGB 1432	P(α)	N7CIS4	NP_541657.1	489	LOV+Kinase	256
	Brucella abortus CNGB 308	P(α)	N7CYG9	NP_541657.1	489	LOV+Kinase	257
	Brucella abortus CNGB 436	P(α)	N7D8M7	NP_541657.1	489	LOV+Kinase	258
	Brucella abortus CNGB 752	P(α)	N7EJN5	NP_541657.1	489	LOV+Kinase	259
	Brucella abortus CNGB 759	P(α)	N7DJA9	NP_541657.1	489	LOV+Kinase	260
	Brucella abortus CNGB 966	P(α)	N7F2F4	NP_541657.1	489	LOV+Kinase	261

	Brucella abortus F1/06 B1	**P(α)**	N7F3R6	NP_541657.1	489	LOV+Kinase	262
	Brucella abortus F1/06-B21	**P(α)**	N7YE57	NP_541657.1	489	LOV+Kinase	263
	Brucella abortus F10/05-11	**P(α)**	N7YGE1	NP_541657.1	489	LOV+Kinase	264
	Brucella abortus F10/06-3	**P(α)**	N7Z0T9	NP_541657.1	489	LOV+Kinase	265
	Brucella abortus F2/06-8	**P(α)**	N7FEA3	WP_006234595.1	375	LOV+Kinase	266
	Brucella abortus F3/01-300	**P(α)**	N7FLM3	NP_541657.1	489	LOV+Kinase	267
	Brucella abortus F3/07-1	**P(α)**	N7FVJ3	NP_541657.1	489	LOV+Kinase	268
	Brucella abortus F5/04-7	**P(α)**	N7Z418	NP_541657.1	489	LOV+Kinase	269
	Brucella abortus F6/05-2	**P(α)**	N7FAF8	NP_541657.1	489	LOV+Kinase	270
	Brucella abortus F6/05-3	**P(α)**	N7Y6J6	NP_541657.1	489	LOV+Kinase	271
	Brucella abortus F6/05-4	**P(α)**	N7ZND9	NP_541657.1	489	LOV+Kinase	272
	Brucella abortus F6/05-9	**P(α)**	N8A0V9	NP_541657.1	489	LOV+Kinase	273
	Brucella abortus I103_(UK3/01)	**P(α)**	R8WBW5	NP_541657.1	489	LOV+Kinase	274
	Brucella abortus levi gila	**P(α)**	N7GL72	NP_541657.1	489	LOV+Kinase	275
	Brucella abortus LEVI237	**P(α)**	N7GMY5	NP_541657.1	489	LOV+Kinase	276
	Brucella abortus NCTC 8038	**P(α)**	D0ATW6	EEW81936.1/YP_001932550.1	489	LOV+PAS+Kinase	277
	Brucella abortus NI240	**P(α)**	N7GW46	NP_541657.1	489	LOV+Kinase	278
	Brucella abortus NI274	**P(α)**	N7H5I5	NP_541657.1	489	LOV+Kinase	279
	Brucella abortus NI352	**P(α)**	N8A4N3	NP_541657.1	489	LOV+Kinase	280
	Brucella abortus NI380	**P(α)**	N7HEI6	NP_541657.1	489	LOV+Kinase	281
	Brucella abortus NI388	**P(α)**	N7HVQ9	NP_541657.1	489	LOV+Kinase	282
	Brucella abortus NI422	**P(α)**	N8AH36	NP_541657.1	489	LOV+Kinase	283
	Brucella abortus NI492	**P(α)**	N7GUN1	NP_541657.1	489	LOV+Kinase	284
	Brucella abortus NI495a	**P(α)**	N8AG43	NP_541657.1	489	LOV+Kinase	285
	Brucella abortus NI518	**P(α)**	N7I5T2	NP_541657.1	489	LOV+Kinase	286
	Brucella abortus NI593	**P(α)**	N7IMP0	NP_541657.1	489	LOV+Kinase	287
	Brucella abortus NI613	**P(α)**	N7IQC5	NP_541657.1	489	LOV+Kinase	288
	Brucella abortus NI622	**P(α)**	N7HW91	NP_541657.1	489	LOV+Kinase	289
	Brucella abortus NI628	**P(α)**	N7I4W1	NP_541657.1	489	LOV+Kinase	290
	Brucella abortus NI633	**P(α)**	N7JHA9	NP_541657.1	489	LOV+Kinase	291
	Brucella abortus NI639	**P(α)**	N7IUD0	NP_541657.1	489	LOV+Kinase	292
	Brucella abortus NI645	**P(α)**	N7K5J7	NP_541657.1	489	LOV+Kinase	293
	Brucella abortus NI649	**P(α)**	N7K6Q2	NP_541657.1	489	LOV+Kinase	294
	Brucella abortus R42-08	**P(α)**	N7ZQU9	NP_541657.1	489	LOV+Kinase	295
	Brucella abortus RB51-AHVLA	**P(α)**	N8L9B2	NP_541657.1	489	LOV+Kinase	296
	Brucella abortus S19	**P(α)**	B2SB67	YP_001932550.1	489	LOV+PAS+ Kinase	297
103	*Brucella canis 79/122*	**P(α)**	N8AYL0	NP_541657.1	489	LOV+Kinase	298
	Brucella canis ATCC 23365	**P(α)**	A9MBM8	YP_001594536.1/NP_541657.1	463	LOV+PAS+ Kinase	299
	Brucella canis CNGB 1172	**P(α)**	N7KG92	NP_541657.1	489	LOV+Kinase	300
	Brucella canis CNGB 1324	**P(α)**	N9S6M2	NP_541657.1	489	LOV+Kinase	301
	Brucella canis CNGB 513	**P(α)**	N8A4Z2	NP_541657.1	489	LOV+Kinase	302
	Brucella canis F7/05A	**P(α)**	N9TZC9	NP_541657.1	489	LOV+Kinase	303
	Brucella canis HSK A52141	**P(α)**	G8SWF4	YP_005152773.1/YP_223408.1	458	LOV+PAS+Kinase	304
	Brucella canis UK10/02	**P(α)**	N7JSW0	NP_541657.1	489	LOV+Kinase	305
104	*Brucella ceti B1/94*	**P(α)**	C9VFB0	WP_002966045.1	456	LOV+PAS+Kinase	306
	Brucella ceti M13/05/1	**P(α)**	C9TAF8	WP_006641754.1	456	LOV+PAS+Kinase	307
	Brucella ceti M490/95/1	**P(α)**	D1FCE4	WP_002966045.1	456	LOV+PAS+Kinase	308
	Brucella ceti M644/93/1	**P(α)**	C9T165	WP_006641754.1	456	LOV+PAS+Kinase	309
	Brucella ceti str. Cudo	**P(α)**	C0GBC6	WP_006016110.1/WP_002970660.1	496	LOV+PAS+ Kinase	310
105	*Brucella melitensis (strain M5-90)*	**P(α)**	F2GXX4	YP_005602181.1/YP_223408.1	458	LOV+PAS+Kinase	311
	Brucella melitensis (strain M28)	**P(α)**	F2HYM1	YP_005598818.1/YP_223408.1	458	PAS+LOV+Kinase	312
	Brucella melitensis 16M	**P(α)**	Q8YC53	NP_541657.1	489	LOV+PAS+ Kinase	313
	Brucella melitensis 64/150	**P(α)**	N7L6K7	NP_541657.1	489	LOV+Kinase	314

	Brucella melitensis 66/59	P(α)	N7L954	NP_541657.1	489	LOV+Kinase	315
	Brucella melitensis ATCC 23457	P(α)	C0RLA9	YP_002734346.1/NP_541657.1	463	LOV+PAS+ Kinase	316
	Brucella melitensis B115	P(α)	N8KUY5	NP_541657.1	489	LOV+Kinase	317
	Brucella melitensis BG2 (S27)	P(α)	N8BQL3	NP_541657.1	489	LOV+Kinase	318
106	*Brucella melitensis biotype 1 NCTC 10094*	P(α)	D0B724	EEW87416.1/NP_541657.1	489	LOV+PAS+Kinase	319
107	*Brucella melitensis bv. 1 str. Rev.1*	P(α)	D1EUC3	WP_002966045.1	456	LOV+PAS+Kinase	320
108	*Brucella melitensis bv. 2 str.63/9*	P(α)	D0GAY7	EEZ16518.1/NP_541657.1	489	PAS+LOV+Kinase	321
109	*Brucella melitensis bv. 3 str. Ether*	P(α)	D1F3T4	WP_004684957.1	456	LOV+PAS+Kinase	322
	Brucella melitensis CNGB 1076	P(α)	N7KEE3	NP_541657.1	489	LOV+Kinase	323
	Brucella melitensis CNGB 1120	P(α)	N7LPJ2	NP_541657.1	489	LOV+Kinase	324
	Brucella melitensis CNGB 290	P(α)	N7L3G2	NP_541657.1	489	LOV+Kinase	325
	Brucella melitensis F1/06 B10	P(α)	N8BTU0	NP_541657.1	489	LOV+Kinase	326
	Brucella melitensis F10/05-2	P(α)	N7LAN8	NP_541657.1	489	LOV+Kinase	327
	Brucella melitensis F10/06-16	P(α)	N8AVE7	NP_541657.1	489	LOV+Kinase	328
	Brucella melitensis F15/06-7	P(α)	N8L9A1	WP_005975426.1	489	LOV+Kinase	329
	Brucella melitensis F2/06-6	P(α)	N7MP94	NP_541657.1	489	LOV+Kinase	330
	Brucella melitensis F3/02	P(α)	N7MZL6	NP_541657.1	489	LOV+Kinase	331
	Brucella melitensis F5/07-239A	P(α)	N7N8P6	WP_005975426.1	489	LOV+Kinase	332
	Brucella melitensis F6/05-6	P(α)	N7N2Y6	NP_541657.1	489	LOV+Kinase	333
	Brucella melitensis F8/01-155	P(α)	N8CHP3	NP_541657.1	489	LOV+Kinase	334
	Brucella melitensis F9/05	P(α)	N8BBC7	NP_541657.1	489	LOV+Kinase	335
	Brucella melitensis NI	P(α)	G4PL89	YP_005660943.1/NP_541657.1	463	LOV+PAS+Kinase	336
	Brucella melitensis R3/07-2	P(α)	N7NQR7	WP_005975426.1	489	LOV+Kinase	337
	Brucella melitensis UK14/06	P(α)	N8CYC4	NP_541657.1	489	LOV+Kinase	338
	Brucella melitensis UK19/04	P(α)	N7NKY4	NP_541657.1	489	LOV+Kinase	339
	Brucella melitensis UK22/04	P(α)	N8BVK8	NP_541657.1	489	LOV+Kinase	340
	Brucella melitensis UK22/06	P(α)	N7P9M1	NP_541657.1	489	LOV+Kinase	341
	Brucella melitensis UK23/06	P(α)	N8DD80	NP_541657.1	489	LOV+Kinase	342
	Brucella melitensis Uk24/06	P(α)	N8DSU1	NP_541657.1	489	LOV+Kinase	343
	Brucella melitensis UK29/05	P(α)	N8DYS6	NP_541657.1	489	LOV+Kinase	344
	Brucella melitensis UK3/06	P(α)	N8D0C9	NP_541657.1	489	LOV+Kinase	345
	Brucella melitensis UK31/99	P(α)	N8EGG8	WP_005975426.1	489	LOV+Kinase	346
	Brucella melitensis UK37/05	P(α)	N8DI44	NP_541657.1	489	LOV+Kinase	347
110	*Brucella microti CCM 4915*	P(α)	C7LI74	YP_003105374.1	489	LOV+PAS+ Kinase	348
111	*Brucella neotomae 5K33*	P(α)	C9V5Q3	WP_004687274.1	456	LOV+PAS+Kinase	349
112	*Brucella ovis ATCC 25840*	P(α)	A5VUS1	YP_001257576.1	463	LOV+PAS+ Kinase	350
113	*Brucella pinnipedialis B2/94*	P(α)	C9TZH	WP_002966045.1	456	LOV+PAS+Kinase	351
	Brucella pinnipedialis B2/94	P(α)	F9YN64	YP_004757837.1/NP_541657.1	489	LOV+PAS+Kinase	352
	Brucella pinnipedialis M292/94/1	P(α)	D1ELF4	WP_002966045.1	456	LOV+PAS+Kinase	353
114	*Brucella sp. 56/94*	P(α)	N8FIV6	NP_541657.1	489	LOV+Kinase	354
115	*Brucella sp. 63/311*	P(α)	N8FUE5	WP_006164629.1	489	LOV+Kinase	355
116	*Brucella sp. 83/13*	P(α)	D1D1A4	WP_008936815.1	456	LOV+PAS+Kinase	356
117	*Brucella sp. BO1*	P(α)	E0DRB7	WP_008510638.1	489	LOV+PAS+Kinase	357
118	*Brucella sp. BO2*	P(α)	E2PN86	WP_009364301.1	434	LOV+Kinase	358
119	*Brucella sp. F23/97*	P(α)	N8G2R7	NP_541657.1	489	LOV+Kinase	359
120	*Brucella sp. F5/99*	P(α)	D0REC6	EEY24775.1/NP_541657.1	489	LOV+PAS+Kinase	360
121	*Brucella sp. F8/99*	P(α)	N8GJ09	NP_541657.1	489	LOV+Kinase	361
122	*Brucella sp. F96/2*	P(α)	N8FEG5	NP_541657.1	489	LOV+Kinase	362
123	*Brucella sp. NF 2653*	P(α)	E0DXH7	WP_009363103.1	489	LOV+PAS+Kinase	363
124	*Brucella sp. NVSL 07-0026*	P(α)	D6LSE	WP_002966045.1	456	LOV+PAS+Kinase	364
125	*Brucella sp. UK1/97*	P(α)	N8FUZ7	NP_541657.1	489	LOV+Kinase	365
126	*Brucella sp. UK40/99*	P(α)	N8H492	NP_541657.1	489	LOV+Kinase	366
127	*Brucella sp. UK5/01*	P(α)	N7P2W7	NP_541657.1	489	LOV+Kinase	367

128	*Brucella suis 01-5744*	P(α)	N8HLS9	NP_541657.1	489	LOV+Kinase	368
	Brucella suis 1330	P(α)	Q8FW73	NP_699772.1	463	LOV+PAS+ Kinase	369
	Brucella suis 63/252	P(α)	N7QG29	NP_541657.1	489	LOV+Kinase	370
	Brucella suis 63/261	P(α)	N8I3A5	NP_541657.1	489	LOV+Kinase	371
	Brucella suis 92/29	P(α)	N7QIN4	NP_541657.1	489	LOV+Kinase	372
	Brucella suis 92/63	P(α)	N7PMB2	NP_541657.1	489	LOV+Kinase	373
	Brucella suis 94/11	P(α)	N7Q4V8	NP_541657.1	489	LOV+Kinase	374
	Brucella suis ATCC 23445	P(α)	A9WYQ7	YP_001622395.1/NP_541657.1	463	LOV+PAS+ Kinase	375
129	*Brucella suis bv. 3 str686*	P(α)	D0PFT	WP_002966045.1	456	LOV+PAS+Kinase	376
130	*Brucella suis bv. 4 str. 40*	P(α)	D0BGV2	WP_004692249.1	489	LOV+PAS+Kinase	377
131	*Brucella suis bv. 5 str.513*	P(α)	D0P6S3	WP_002966045.1	456	LOV+PAS+Kinase	378
	Brucella suis CNGB 247	P(α)	N8GXW2	NP_541657.1	489	LOV+Kinase	379
	Brucella suis CNGB 786	P(α)	N7Q721	NP_541657.1	489	LOV+Kinase	380
	Brucella suis F12/02	P(α)	N8IR38	NP_541657.1	489	LOV+Kinase	381
	Brucella suis F4/06-146	P(α)	N7QNL3	NP_541657.1	489	LOV+Kinase	382
	Brucella suis F5/03-2	P(α)	N7RVY7	NP_541657.1	489	LOV+Kinase	383
	Brucella suis F5/05-10	P(α)	N8IZA7	NP_541657.1	489	LOV+Kinase	384
	Brucella suis F5/05-4	P(α)	N8HS15	NP_541657.1	489	LOV+Kinase	385
	Brucella suis F7/06-1	P(α)	N8JI74	NP_541657.1	489	LOV+Kinase	386
	Brucella suis F7/06-2	P(α)	N8JJG0	NP_541657.1	489	LOV+Kinase	387
	Brucella suis F7/06-5	P(α)	N8ILB3	NP_541657.1	489	LOV+Kinase	388
	Brucella suis F8/06-1	P(α)	N8K0M4	NP_541657.1	489	LOV+Kinase	389
	Brucella suis F8/06-2	P(α)	N7R8U4	NP_541657.1	489	LOV+Kinase	390
	Brucella suis F8/06-3	P(α)	N8KIG9	NP_541657.1	489	LOV+Kinase	391
	Brucella suis F9/06-1	P(α)	N8KJS5	NP_541657.1	489	LOV+Kinase	392
	Brucella suis VBI22	P(α)	G8NMK8	YP_005108587.1/NP_541657.1	463	LOV+PAS+Kinase	393
132	*Caulobacter crescentus (strain NA1000 / CB15N)*	P(α)	B8GYF7	YP_002515662.1/NP_419104.2	368	LOV+ Kinase	394
	Caulobacter crescentus CB15	P(α)	Q9ABE3	NP_419104.2	449	LOV+ Kinase	395
	Caulobacter crescentus OR37	P(α)	R0EN90	WP_004615751.1	368	LOV+Kinase	396
133	*Caulobacter segnis DSM 7131*	P(α)	D5VP68	YP_003594909.1	366	LOV+Kinase	397
134	*Caulobacter sp. AP07*	P(α)	J3A321	WP_007673901.1	369	LOV+Kinase	398
135	*Caulobacter sp. JGI 0001013-O16*	P(α)		WP_018061565.1	373	LOV+Kinase	399
136	*Caulobacter sp. K31*	P(α)	B0T3W0	YP_001684922.1	360	LOV+ Kinase	400
			B0T127	YP_001686173.1	369	LOV+ Kinase	401
			B0T3W1	YP_001684923.1	922	LOV+3PAS +Kinase+RR	402
137	*Citreicella sp. 357*	P(α)	I1B280	WP_009502647.1	178	Short-LOV	403
138	*Citreicella sp. SE45*	P(α)	D0CZ83	WP_008883518.1	182	Short-LOV	404
139	*Citromicrobium bathyomarinum*	P(α)		WP_010236370.1	335	LOV+Kinase	405
140	*Citromicrobium sp. JLT1363*	P(α)		WP_010408641.1	358	LOV+Kinase	406
				WP_010412930.1	222	LOV+RR	407
141	*Dinoroseobacter shibae DFL 12*	P(α)	A8LHT	YP_001532478.1	338	LOV+ Kinase	408
			A8LNC2	YP_001533236.1	920	LOV+PAS+PAS+Kinase+RR	409
			A8LP63	YP_001533346.1	139	Short-LOV	410
142	*Elioraea tepidiphila*	P(α)		WP_019014442.1	671	LOV+Kinase+RR+HPT	411
143	*Erythrobacter litoralis HTCC2594*	P(α)	Q2NB98	YP_457840.1	225	LOV+HTH	412
			Q2NB77	YP_457861.1	346	LOV+ Kinase	413
			Q2N9L9	YP_458419.1	362	LOV+ Kinase	414
			Q2NCA3	YP_457485.1	360	LOV+ Kinase	415
144	*Erythrobacter sp. NAP1*	P(α)	A3WAQ8	WP_007164748.1	358	LOV+ Kinase	416
145	*Erythrobacter sp. SD-21*	P(α)	A5PBU7	WP_006833624.1	359	LOV+ Kinase	417
146	*Fulvimarina pelagi HTCC2506*	P(α)	Q0G496	WP_007067960.1	420	LOV+ Kinase	418
147	*Gluconacetobacter europaeus*	P(α)		WP_019087014.1	213	LOV+RR	419
148	*Gluconobacter frateurii NBRC 103465*	P(α)		GAD09355.1	550	LOV+Kinase+RR	420

149	*Gluconobacter thailandicus NBRC 3255*	P(α)	M9M8A6	WP_007284404.1	578	LOV+Kinase+RR	421
150	*Granulibacter bethesdensis CGDNIH1*	**P(α)**	Q0BT22	YP_744953.1	185	Short-LOV	422
151	*Hyphomicrobium sp. 99*	P(α)		WP_020186668.1	383	LOV+Kinase	423
152	*Ketogulonicigenium vulgare str. Y25*	P(α)	E3F4V0	YP_003944033.1	483	LOV+PAS+Kinase	424
	Ketogulonicigenium vulgare WSH-001	P(α)	F9YAW8	YP_003944033.1	483	LOV+PAS+Kinase	425
153	*Labrenzia alexandrii DFL-11*	P(α)	B9QXI1	WP_008197038.1	124	Short-LOV	426
			B9QXR5	WP_008189163.1	369	LOV+ Kinase	427
154	*Loktanella hongkongensis*	P(α)		WP_017927856.1	360	LOV+Kinase	428
				WP_017929482.1	330	LOV+Kinase	429
155	*Magnetospirillum magnetotacticum*	P(α)		WP_009866659.1	358	LOV+Kinase	430
		P(α)		WP_009865584.1	425	LOV+Kinase	431
156	*Manganese-oxidizing bacterium (strain SI85-9A1)*	P(α)	Q1YFS4	WP_009210920.1	389	LOV+ Kinase	432
	Manganese-oxidizing bacterium (strain SI85-9A1)	P(α)	Q1YEU2	WP_009211252.1	415	LOV+ Kinase	433
157	*Mesorhizobium australicum LMG 24608*	P(α)	L0KJN1	YP_007304602.1	367	LOV+Kinase	434
	Mesorhizobium australicum LMG 24609	P(α)	L0KP22	YP_007305199.1	382	LOV+Kinase	435
158	*Mesorhizobium ciceri bv. biserrulae (strain HAMBI 2942)*	P(α)	E8TG50	YP_004142370.1	382	LOV+Kinase	436
159	*Mesorhizobium loti*	P(α)		WP_019859431.1	572	LOV+GGDEF+EAL	437
				WP_019859046.1	369	LOV+Kinase	438
160	*Methylobacterium chloromethanicum CM4*	P(α)	B7KUQ2	YP_002422209.1	533	LOV+Kinase+RR	439
			B7KW67	YP_002422352.1	354	LOV+Kinase	440
			B7KN66	YP_002423111.1	366	LOV+Kinase	441
			B7KX89	YP_002424066.1	890	LOV+PAS+GAF+Kinase+RR	442
			B7KVX8	YP_002420722.1/YP_001639119.1	541	LOV+Kinase+RR	443
			B7L0N0	YP_002419681.1	488	LOV+PAS+ Kinase	444
161	*Methylobacterium extorquens AM1*	P(α)	C5B055	YP_002962682.1/YP_001639119.1	541	LOV+Kinase+RR	445
			C5AXM1	YP_002964366.1	533	LOV+Kinase+RR	446
			C5B09	YP_002962726.1	492	LOV+PAS+Kinase	447
			C5AYD0	YP_002964506.1	354	LOV+Kinase	448
			C5AVA5	YP_002961850.1	488	LOV+PAS+Kinase	449
			C5B328	YP_002965335.1	351	LOV+Kinase	450
	Methylobacterium extorquens DM4	P(α)	C7CG25	YP_003067865.1/YP_001639119.1	541	LOV+Kinase+RR	451
			C7CC12	YP_003069406.1	533	LOV+Kinase+RR	452
			C7CDA8	YP_003066654.1	488	LOV+PAS+Kinase	453
			C7CCX6	YP_003069551.1	354	LOV+Kinase	454
			C7CJE3	YP_003070459.1	351	LOV+Kinase	455
	Methylobacterium extorquens DSM 13060	P(α)	H1KGJ9	EHP93379.1/YP_002961850.1	488	LOV+PAS+Kinase	456
			H1KRD3	WP_003604879.1	492	LOV+PAS+Kinase	457
			H1KD56	YP_001926756.1	888	LOV+PAS+GAF+Kinase+RR	458
			H1KIH3	EHP92693.1/YP_001639119.1	541	LOV+Kinase+RR	459
			H1KHY7	WP_003599658.1	366	LOV+Kinase	460
			H1KNL7	EHP90884.1/YP_002964506.1	354	LOV+Kinase	461
			H1KF02	WP_003597944.1	533	LOV+Kinase+RR	462
			H1KRD3	WP_003604879.1	492	LOV+PAS+Kinase	463
	Methylobacterium extorquens PA1	P(α)	A9VYT5	YP_001640598.1	533	LOV+Kinase+RR	464
			A9W705	YP_001640738.1	354	LOV+Kinase	465
			A9W8Q8	YP_001641478.1	366	LOV+Kinase	466
			A9W392	YP_001639119.1	541	LOV+Kinase+RR	467
			A9W12	YP_001638351.1	488	LOV+Kinase	468
162	*Methylobacterium mesophilicum SR1.6/6*	P(α)	M7XWQ9	WP_010687353.1	186	Short-LOV	469
			M7XRI0	WP_010686681.1	1194	LOV+5PAS+Kinase+RR	470
			M7YQT1	WP_010684528.1	458	LOV+Kinase	471
			M7YNE8	WP_010684883.1	164	Short-LOV	472
			M7Y4Z9	WP_010684081.1	334	LOV+Kinase	473

			M7Y7G4	WP_010684609.1	538	LOV+Kinase+RR	474
163	*Methylobacterium nodulans ORS 2060*	P(α)	B8IAH5	YP_002501323.1	559	LOV+Kinase+RR	475
			B8IF91	YP_002496105.1	814	LOV+2PAS+Kinase+RR	476
			B8IJ45	YP_002502143.1	357	LOV+Kinase	477
164	*Methylobacterium populi BJ001*	P(α)	B1ZJK1	YP_001926022.1	531	LOV+Kinase+RR	478
			B1Z812	YP_001923514.1	488	LOV+PAS+ Kinase	479
			B1ZCH2	YP_001926756.1	888	LOV+PAS+GAF+Kinase+RR	480
			B1ZG87	YP_001924280.1	541	LOV+Kinase+RR	481
			B1ZKQ8	YP_001926163.1	354	LOV+Kinase	482
			B1ZGT9	YP_001927152.1	366	LOV+Kinase	483
			B1ZH86	YP_001923025.1	190	Short-LOV	484
			B1ZI10	YP_001923031.1	901	LOV+3PAS+GAF+Kinase	485
165	*Methylobacterium radiotolerans ATCC 27329*	P(α)	B1M4A2	YP_001757080.1	186	Short-LOV	486
			B1LWD2	YP_001753317.1	334	LOV+ Kinase	487
			B1LUV7	YP_001756226.1	539	LOV+Kinase+RR	488
			B1M51	YP_001757160.1	164	Short-LOV	489
			B1LSK7	YP_001755992.1	503	LOV+PAS+Kinase	490
			B1M4V9	YP_001755688.1	812	LOV+2PAS+Kinase+RR	491
166	*Methylobacterium sp. 285MFTsu5.1*	P(α)		WP_020096052.1	186	Short-LOV	492
				WP_020092559.1	539	LOV+Kinase+RR	493
				WP_020091307.1	164	Short-LOV	494
				WP_020093089.1	495	LOV+PAS+Kinase	495
				WP_020093482.1	358	LOV+Kinase	496
				WP_020094544.1	813	LOV+2PAS+Kinase+RR	497
167	*Methylobacterium sp. 4-46*	P(α)	B0UC38	YP_001773223.1	368	LOV+Kinase	498
			B0UAI7	YP_001768942.1	812	LOV+2PAS+Kinase+RR	499
			B0UDT0	YP_001771637.1	544	LOV+Kinase+RR	500
168	*Methylobacterium sp. 77*	P(α)		WP_019903241.1	540	LOV+Kinase+RR	501
				WP_019905522.1	421	LOV+Kinase	502
				WP_019902898.1	499	LOV+PAS+Kinase	503
				WP_019903195.1	384	LOV+Kinase	504
				WP_019903666.1	801	LOV+2PAS+Kinase+RR	505
169	*Methylobacterium sp. 88A*	P(α)		WP_018042785.1	525	LOV+Kinase+RR	506
				WP_018045200.1	374	LOV+Kinase	507
				WP_018044613.1	1061	LOV+4PAS+Kinase+RR	508
				WP_018042266.1	506	LOV+PAS+Kinase	509
				WP_018042735.1	388	LOV+Kinase	510
				WP_018043311.1	801	LOV+2PAS+Kinase+RR	511
170	*Methylobacterium sp. GXF4*	P(α)	I9WQP0	WP_007566581.1	346	LOV+Kinase	512
			I9CC12	WP_007569445.1	489	LOV+Kinase	513
			I9CG57	WP_007567616.1	164	Short-LOV	514
			I9CKA6	WP_007564509.1	807	LOV+2PAS+Kinase+RR	515
			I9WYU0	WP_007560659.1	373	LOV+Kinase	516
			I9LEW2	WP_007565725.1	524	LOV+Kinase+RR	517
171	*Methylobacterium sp. MB200*	P(α)		WP_017485058.1	541	LOV+Kinase+RR	518
				WP_017486386.1	531	LOV+Kinase+RR	519
				WP_017487214.1	488	LOV+PAS+Kinase	520
				WP_017484340.1	842	LOV+PAS+GAF+Kinase	521
				WP_017484125.1	351	LOV+Kinase	522
172	*Methylobacterium sp. WSM2598*	P(α)		WP_018263266.1	368	LOV+Kinase	523
				WP_018259506.1	544	LOV+Kinase+RR	524
				WP_018261724.1	802	LOV+2PAS+Kinase+RR	525
173	*Methylocystis parvus*	P(α)		WP_016919364.1	487	DJ-1+HTH+LOV	526

174	*Methylocystis rosea*	P(α)		WP_018409659.1	149	Short-LOV	527
				WP_018407875.1	488	DJ-1+HTH+LOV	528
				WP_018406994.1	150	Short-LOV	529
175	*Methylocystis sp. SC2*	P(α)	J7QP72	YP_006591864.1	150	Short-LOV	530
176	*Methylocystis sp. SC3*	P(α)	J7QQR2	YP_006590956.1	488	HTH+LOV	531
177	*Microvirga sp. WSM3557*	P(α)	I4YU75	WP_009763567.1	586	LOV+Kinase+RR	532
			I4YYC1	WP_009491709.1	896	LOV+3PAS+Kinase+RR	533
178	*Nitrosomonas sp. AL212*	P(α)	F9ZGQ1	YP_004295366.1	150	Short-LOV	534
179	*Novosphingobium aromaticivorans DSM 12444*	P(α)	Q2G5U0	YP_497617.1	364	LOV+ Kinase	535
			Q2G8Z7	YP_496510.1	223	LOV+HTH	536
180	*Novosphingobium nitrogenifigens DSM 19370*	P(α)	F1ZCX7	WP_008071222.1	196	LOV+RR	537
181	*Novosphingobium sp. AP12*	P(α)	J3AQP3	WP_007676988.1	873	LOV+GAF+Kinase+RR	538
		P(α)	J2P4S6	WP_007687188.1	470	LOV+Kinase	539
182	*Novosphingobium sp. PP1Y*	P(α)	F6ID38	YP_004538180.1	193	Short-LOV	540
183	*Oceanibulbus indolifex HEL-45*	P(α)	A9DXF0	WP_007118258.1	354	LOV+ Kinase	541
184	*Oceanicola granulosus*	P(α)	Q2CIF5	WP_007255024.1	341	LOV+ Kinase	542
185	*Oceanicola sp. S124*	P(α)		WP_010139701.1	346	LOV+Kinase	543
186	*Ochrobactrum anthropi*	P(α)		WP_010658851.1	480	LOV+Kinase	544
	Ochrobactrum anthropi ATCC 49188	P(α)	A6X554	YP_001372187.1	491	LOV+PAS+ Kinase	545
187	*Ochrobactrum intermedium 229E*	P(α)		ERM00762.1	495	LOV+PAS+Kinase	546
	Ochrobactrum intermedium LMG 3301	P(α)	C4WLE3	WP_006468921.1	495	LOV+PAS+ Kinase	547
	Ochrobactrum intermedium M86	P(α)	M5JPW6	WP_006471383.1	491	LOV+Kinase	548
188	*Ochrobactrum sp. CDB2*	P(α)	M3JKE0	WP_007878669.1	496	LOV+PAS+Kinase	549
189	*Ochrobactrum sp. EGD-AQ16*	P(α)		WP_021587245.1	460	LOV+PAS+Kinase	550
190	*Parvularcula bermudensis HTCC2503*	P(α)	E0TE19	YP_003853982.1	367	LOV+Kinase	551
			E0THB6	YP_003855348.1	361	LOV+Kinase	552
191	*Phyllobacterium sp. YR531*	P(α)	J3HS51	WP_008123941.1	485	LOV+Kinase	553
192	*Rhizobium etli bv. mimosae str. Mim1*	P(α)	S5S9C3	YP_008369537.1	345	LOV+Kinase	554
193	*Rhizobium gallicum*	P(α)		WP_018446992.1	325	LOV+Kinase	555
194	*Rhizobium giardinii*	P(α)		WP_018327555.1	336	LOV+Kinase	556
195	*Rhizobium grahamii*	P(α)		WP_016556202.1	349	LOV+Kinase	557
196	*Rhizobium leguminosarum*	P(α)		WP_017991794.1	375	LOV+Kinase	558
				WP_017996607.1	375	LOV+Kinase	559
				WP_018068093.1	345	LOV+Kinase	560
				WP_017959402.1	345	LOV+Kinase	561
				WP_017997493.1	342	LOV+Kinase	562
				WP_017992121.1	345	LOV+Kinase	563
				WP_018485836.1	345	LOV+Kinase	564
				WP_018496455.1	345	LOV+Kinase	565
197	*Rhizobium leguminosarum bv viciae 3841*	P(α)	Q1M667	YP_771353.1	345	LOV+ Kinase	566
198	*Rhizobium leguminosarum bv. trifolii WSM1325*	P(α)	C6B8Y	YP_002978926.1	342	LOV+ Kinase	567
			C6B7A8	YP_002984928.1	375	LOV+ Kinase	568
199	*Rhizobium leguminosarum bv. trifolii WSM2297*	P(α)	J0WGH8	WP_003575436.1	345	LOV+Kinase	569
200	*Rhizobium leguminosarum bv. trifolii WSM2304*	P(α)	B6A1G8	YP_002279192.1	345	LOV+ Kinase	570
201	*Rhizobium leguminosarum bv. trifolii WSM597*	P(α)	J0H7F5	WP_003591079.1	345	LOV+Kinase	571
202	*Rhizobium leguminosarum bv. viciae WSM1455*	P(α)	J0BMT6	WP_003548985.1	345	LOV+Kinase	572
		P(α)	J0BMD1	WP_003548626.1	375	LOV+Kinase	573
203	*Rhizobium lupini HPC(L)*	P(α)	K5C4K8	WP_006697873.1	369	LOV+Kinase	574
204	*Rhizobium sp. 2MFCol3.1*	P(α)		WP_018901391.1	205	Short-LOV	575
				WP_018901378.1	369	LOV+Kinase	576
				WP_018899439.1	476	LOV+PAS+Kinase	577
				WP_018901815.1	482	LOV+PAS+Kinase	578
205	*Rhizobium sp. CF080*	P(α)	J3CCW4	WP_007757319.1	349	LOV+Kinase	579

			J2IVB7	WP_007767523.1	375	LOV+Kinase	580
206	*Rhizobium sp. JGI 0001005-K05*	P(α)		WP_018116037.1	365	LOV+Kinase	581
				WP_018114278.1	387	LOV+Kinase	582
207	*Rhizobium tropici CIAT 899*	P(α)	L0LP99	YP_007335729.1	368	LOV+Kinase	583
208	*Rhodobacter sp. AKP1*	P(α)	L1KCG9	YP_352285.1	176	Short-LOV	584
			L1K6L9	WP_009565994.1	944	PAS+LOV+PAS+Kinase+RR	585
209	*Rhodobacter sphaeroides ATCC 17023*	P(α)	Q3J0K0	YP_353585.1	945	PAS+LOV+PAS+Kinase+RR	586
			Q3J4A0	YP_352285.1	176	Short-LOV	587
210	*Rhodobacter sphaeroides ATCC 17029*	P(α)	A3PLQ1	YP_001044039.1	941	PAS+LOV+2PAS+Kinase+RR	588
			A3PI49	YP_001042787.1/YP_352285.1	176	Short-LOV	589
211	*Rhodobacter sphaeroides KD131*	P(α)	B9KKQ9	YP_002526207.1	941	PAS+LOV+PAS+Kinase+RR	590
			B9KPB3	YP_002524907.1/YP_352285.1	176	Short-LOV	591
212	*Rhodobacter sphaeroides WS8N*	P(α)	F5M486	WP_002720676.1	945	PAS+LOV+Kinase+RR	592
213	*Rhodospirillum centenum ATCC 51521 / SW*	P(α)	B6IQQ9	YP_002296608.1	368	LOV+ Kinase	593
214	*Roseobacter denitrificans OCh 114*	P(α)	Q167W8	YP_682411.1	350	LOV+ Kinase	594
215	*Roseobacter litoralis DSM 6996*	P(α)	F7ZD60	YP_004690232.1	350	LOV+Kinase	595
216	*Roseobacter sp. CCS2*	P(α)	A4EHF4	WP_008232737.1	175	Short-LOV	596
			A4EIJ8	WP_008233475.1	181	Short-LOV	597
217	*Roseomonas cervicalis ATCC 49957*	P(α)	D5RKE3	WP_007004777.1	537	LOV+Kinase+RR	598
218	*Roseomonas sp. B5*	P(α)		WP_019461164.1	807	LOV+2PAS+Kinase+RR	599
219	*Roseovarius sp. HTCC2601*	P(α)	Q0FR10	WP_007797918.1	182	Short-LOV	600
220	*Rubellimicrobium thermophilum DSM 16684*	P(α)	S9SB69	WP_021096554.1	349	LOV+Kinase	601
221	*Ruegeria sp. R11*	P(α)	B7QQ97	WP_008562686.1	194	Short-LOV	602
222	*Sagittula stellata E-37*	P(α)	A3K7J	WP_005861688.1	169	Short-LOV	603
223	*Salipiger mucosus*	P(α)		WP_020039151.1	182	Short-LOV	604
224	*Sphingobium baderi LL03*	P(α)	T0GNC9	WP_021244768.1	868	LOV+PAS+GAF+Kinase	605
225	*Sphingobium indicum B90A*	P(α)	I5BEG6	WP_007685143.1	572	LOV+Kinase+RR	606
226	*Sphingobium japonicum*	P(α)		WP_006948855.1	195	LOV+RR	607
227	*Sphingobium lactosutens DS20*	P(α)	T0IZE5	WP_021224791.1	201	LOV+RR	608
			T0HKJ8	WP_021227208.1	592	LOV+Kinase+RR	609
				EQB17255.1	201	LOV+RR	610
228	*Sphingobium ummariense RL-3*	P(α)	T0KBW2	WP_021316219.1	572	LOV+Kinase+RR	611
229	*Sphingobium xenophagum*	P(α)		WP_017181268.1	582	LOV+Kinase+RR	612
				WP_019051957.1	190	LOV+RR	613
				WP_017181670.1	190	LOV+RR	614
230	*Sphingobium yanoikuyae*	P(α)		WP_010336618.1	185	Short-LOV	615
	Sphingobium yanoikuyae ATCC 51230	P(α)	K9DDM0	WP_004210595.1	460	LOV+Kinase	616
			K9DBT8	WP_004209647.1	196	LOV+RR	617
			K9CRY6	WP_004209577.1	185	Short-LOV	618
			K9CZR2	WP_004208793.1	866	LOV+PAS+GAF+Kinase+RR	619
231	*Sphingomonas echinoides*	P(α)		WP_010406583.1	870	LOV+PAS+GAF+Kinase+RR	620
232	*Sphingomonas elodea*	P(α)		WP_010544137.1	568	LOV+Kinase+RR	621
				WP_010545569.1	193	LOV+RR	622
233	*Sphingomonas melonis*	P(α)		WP_020486392.1	488	LOV+PAS+Kinase	623
				WP_018250488.1	496	LOV+PAS+Kinase	624
				WP_020493474.1	586	LOV+Kinase+RR	625
				WP_018250616.1	569	LOV+Kinase+RR	626
234	*Sphingomonas sp. ATCC 31555*	P(α)		WP_019369245.1	569	LOV+PAS+Kinase	627
				WP_019369958.1	195	LOV+RR	628
235	*Sphingomonas sp. KC8*	P(α)		WP_010126430.1	191	LOV+RR	629
236	*Sphingomonas sp. LH128*	P(α)	J8VLA8	WP_008831260.1	184	Short-LOV	630
			J8VSB8	WP_008829154.1	186	Short-LOV	631
237	*Sphingomonas sp. Mn802worker*	P(α)		WP_019517892.1	547	LOV+Kinase+RR	632

				WP_019515952.1	374	LOV+Kinase	633
238	*Sphingomonas sp. PAMC 26605*	P(α)		WP_010185187.1	368	LOV+Kinase	634
				WP_010183492.1	207	LOV+RR	635
239	*Sphingomonas sp. PAMC 26617*	P(α)		WP_010164100.1	536	LOV+Kinase+RR	636
				WP_010165385.1	207	LOV+RR	637
				WP_010162859.1	849	PAS+LOV+GAF+Kinase+RR	638
				WP_010162636.1	207	LOV+RR	639
				WP_010161173.1	557	LOV+Kinase+RR	640
240	*Sphingomonas sp. PAMC 26621*	P(α)		WP_010215598.1	207	LOV+RR	641
				WP_010217310.1	536	LOV+Kinase+RR	642
241	*Sphingomonas sp. S17*	P(α)	F3WVL3	WP_007404345.1	155	Short-LOV	643
		P(α)	F3WVM0	WP_007404471.1	524	LOV+Kinase+RR	644
242	*Sphingomonas sp. SKA58*	P(α)	Q1N7J1	WP_009822011.1	238	LOV+HTH	645
			Q1NI33	WP_009823476.1	570	LOV+Kinase+RR	646
243	*Sphingopyxis alaskensis RB2256*	P(α)	Q1GUF5	YP_616050.1	195	LOV+HTH	647
244	*Sphingopyxis sp. MC1*	P(α)	N9WD00	WP_003043817.1	566	LOV+GGDEF+EAL	648
			N9UPB3	WP_003050900.1	195	LOV+RR	649
245	*Wenxinia marina*	P(α)		WP_018301439.1	178	Short-LOV	650
246	*Accumulibacter phosphatis (strain UW-1)*	P(β)	C7RJT7	YP_003168707.1	143	Short-LOV	651
247	*Acidovorax avenae*	P(β)		WP_019702225.1	544	LOV+Kinase+RR	652
	Acidovorax avenae DSM 7227	P(β)	F0QBL7	YP_004235943.1	544	LOV+Kinase+RR	653
248	*Acidovorax avenae sub. citrulli AAC00-1*	P(β)	A1TT27	YP_971889.1	544	LOV+Kinase+RR	654
249	*Aromatoleum aromaticum EbN1*	P(β)	Q5P4K8	YP_158656.1	698	Globin+LOV+GGDEF+EAL	655
250	*Blood disease bacterium R229*	P(β)	G2ZKN6	CCA79599.1	1178	3PAS+LOV+GGDEF	656
251	*Burkholderia bryophila*	P(β)		WP_020069641.1	1058	2PAS+LOV+GGDEF+EAL	657
252	*Burkholderia graminis C4D1M*	P(β)	B1FWL0	WP_006047954.1	1036	PAS+PAS+LOV+ GGDEF+EAL	658
253	*Burkholderia kururiensis*	P(β)		WP_017772705.1	1038	2PAS+LOV+GGDEF+EAL	659
254	*Burkholderia phenoliruptrix BR3459a*	P(β)	K0DLX0	YP_006832691.1	1036	2PAS+LOV+GGDEF+EAL	660
255	*Burkholderia phymatum STM815*	P(β)	B2JFQ7	YP_001858578.1	533	LOV+Kinase+RR	661
			B2JCW5	YP_001856630.1	1040	2PAS+LOV+GGDEF+EAL	662
256	*Burkholderia phytofirmans PsJN*	P(β)	B2SX52	YP_001894315.1	1036	PAS+PAS+LOV+ GGDEF+EAL	663
257	*Burkholderia sp. BT03*	P(β)	J3AY22	WP_007745224.1	1040	2PAS+LOV+GGDEF+EAL	664
			J2IBD0	WP_007737745.1	533	LOV+Kinase+RR	665
258	*Burkholderia sp. CCGE 1002*	P(β)	D5WBG3	YP_003604003.1	1055	2PAS+LOV+GGDEF+EAL	666
259	*Burkholderia sp. CCGE 1003*	P(β)	E1T757	YP_003905686.1	1036	2PAS+LOV+GGDEF+EAL	667
260	*Burkholderia sp. CCGE1001*	P(β)	E8YMJ9	YP_004226904.1	1036	2PAS+LOV+GGDEF+EAL	668
261	*Burkholderia sp. Ch1-1*	P(β)	I2ICW6	WP_007179328.1	1036	2PAS+LOV+GGDEF+EAL	669
262	*Burkholderia sp. H160*	P(β)	B5WM15	WP_008921106.1	1028	PAS+PAS+LOV+ GGDEF+EAL	670
263	*Burkholderia sp. JPY251*	P(β)		WP_018431938.1	1055	2PAS+LOV+GGDEF+EAL	671
264	*Burkholderia sp. RPE64*	P(β)		YP_008036462.1	1058	PAS+LOV+PAS+GGDEF+EAL	672
265	*Burkholderia sp. SJ98*	P(β)	K8R5H1	WP_008351594.1	1032	2PAS+LOV+GGDEF+EAL	673
			K8RBL1	WP_008348805.1	950	PAS+LOV+GGDEF+EAL	674
			K8RFA8	WP_008344710.1	403	LOV+Kinase	675
266	*Burkholderia sp. WSM4176*	P(β)		WP_018419411.1	1062	2PAS+LOV+GGDEF+EAL	676
267	*Burkholderia sp.YI23*	P(β)	G8M3Y7	YP_004976128.1	1040	2PAS+LOV+GGDEF+EAL	677
268	*Burkholderia terrae BS001*	P(β)	I5D017	WP_007579791.1	1040	2PAS+LOV+GGDEF+EAL	678
			I5CKV7	WP_007589128.1	192	Short-LOV	679
269	*Burkholderia xenovorans LB400*	P(β)	Q145M2	YP_557019.1	1036	PAS+PAS+LOV+ GGDEF+EAL	680
270	*Burkholderiales bacterium JOSHI_001*	P(β)	H5WRX6	WP_009553309.1	875	LOV+Kinase+GGDEF+HAMP	681
271	*Caldimonas manganoxidans*	P(β)		WP_019559344.1	1200	GAF+2PAS+LOV+2PAS+Kinase+RR	682
272	*Comamonas testosteroni*	P(β)		WP_019042804.1	443	LOV+GGDEF	683
	Comamonas testosteroni CNB-2	P(β)	D0J0D1	YP_003278898.1	458	LOV+PAS+GGDEF	684
	Comamonas testosteroni S44	P(β)	D8D2C5	WP_003062841.1	435	PAS+LOV+GGDEF	685

273	*Duganella zoogloeoides*	P(β)		WP_019924259.1	560	LOV+Kinase+RR	686
274	*Herbaspirillum frisingense GSF30*	P(β)	R0FWK0	WP_006462257.1	546	LOV+Kinase+RR	687
275	*Herbaspirillum seropedicae*	P(β)		WP_017453457.1	546	LOV+Kinase+RR	688
	Herbaspirillum seropedical (strain SmR1)	P(β)	D8IWD2	YP_003775860.1	552	LOV+Kinase+RR	689
276	*Herbaspirillum sp. GW103*	P(β)	I3CVK0	WP_008329090.1	546	LOV+Kinase+RR	690
277	*Herbaspirillum sp. JC206*	P(β)		WP_019142500.1	585	LOV+Kinase+RR	691
278	*Herbaspirillum sp. YR522*	P(β)	J2UWT8	WP_008118789.1	549	LOV+Kinase+RR	692
279	*Herminiimonas arsenicoxydans*	P(β)	A4G2U1	YP_001098955.1	944	CHASE+LOV+GGDEF+EAL	693
			A4G7R2	YP_001100670.1	313	LOV+GGDEF	694
			A4G3M6	YP_001099240.1	818	RR+PAS+LOV+GGDEF+EAL	695
280	*Janthinobacterium sp. CG3*	P(β)		WP_017876122.1	1107	CHASE+LOV+GGDEF+EAL+RR	696
				WP_017876665.1	986	PAS+LOV+GGDEF+EAL+RR	697
281	*Lutiella nitroferrum 2002*	P(β)	B9Z647	WP_008955033.1	1072	RR+GAF+PAS+LOV+GGDEF+EAL	698
282	*Massilia niastensis*	P(β)		WP_020651816.1	562	LOV+Kinase+RR	699
283	*Massilia timonae CCUG 45783*	P(β)	K9DG25	WP_005665462.1	569	LOV+Kinase+RR	700
284	*Methylibium petroleiphilum PM1*	P(β)	A2SIR0	YP_001021684.1	1317	GAF+3PAS+LOV+2PAS+Kinase+ RR	701
285	*Methylovorus sp. SIP3-4*	P(β)	C6X7I8	YP_003051980.1	821	RR+PAS+LOV+GGDEF+EAL	702
286	*Methylovorus sp.str. MP688*	P(β)	E4QL91	YP_004040548.1	821	RR+PAS+LOV+GGDEF+EAL	703
287	*Nitrosomonas sp. (strain Is79A3)*	P(β)	F8GDY9	YP_004695500.1	150	Short-LOV	704
288	*Nitrosomonas sp. AL212*	P(β)	F9ZGQ1	YP_004295366.1	150	Short-LOV	705
289	*Nitrosospira multiformis ATCC 25196*	P(β)	Q2Y837	YP_412476.1	152	Short-LOV	706
290	*Nitrosospira sp. APG3*	P(β)	M5DIF4	WP_004176761.1	157	Short-LOV	707
291	*Oxalobacteraceae bacterium IMCC 9480*	P(β)	F1VZT5	WP_009666397.1	526	LOV+PAS+Kinase	708
292	*Polaromonas napHTHalenivorans CJ2*	P(β)	A1VS30	YP_983379.1	389	LOV+ Kinase	709
293	*Pseudogulbenkiania sp. (strain NH8B)*	P(β)	G2IXN9	YP_004848386.1	1072	RR+GAF+PAS+LOV+GGDEF+EAL	710
294	*Ralstonia solanacearum*	P(β)	B5RXV5	WP_003274043.1	1178	Hamp+2PAS+LOV+GGDEF+EAL	711
				WP_019719220.1	1178	Hamp+2PAS+LOV+GGDEF+EAL	712
			D8P2A9	YP_003747462.1	1178	Hamp+2PAS+LOV+GGDEF+EAL	713
			B5SBH9	YP_002257212.1	1178	Hamp+2PAS+LOV+GGDEF+EAL	714
	Ralstonia solanacearum CMR15	P(β)	D8NG57	YP_006060163.1	1178	Hamp+2PAS+LOV+GGDEF+EAL	715
	Ralstonia solanacearum FQY_4	P(β)	M4UIH4	YP_008237427.1	1178	Hamp+2PAS+LOV+GGDEF+EAL	716
	Ralstonia solanacearum GMI1000	P(β)	Q8XT61	NP_521815.1	1178	HAMP+2PAS+LOV+GGDEF+EAL	717
	Ralstonia solanacearum PSI07	P(β)	D8MYZ9	YP_003749222.1	1178	3PAS+LOV+GGDEF+EAL	718
	Ralstonia solanacearum SD54	P(β)	S7XX18	WP_021155319.1	1178	Hamp+2PAS+LOV+GGDEF+EAL	719
	Ralstonia solanacearum str. Po82	P(β)	F6G7G0	YP_006031432.1	1234	HAMP+PAS+LOV+GGDEF+EAL	720
	Ralstonia solanacearum UW551	P(β)	A3RWI2	WP_003264369.1	1234	HAMP+2PAS+LOV+GGDEF+EAL	721
295	*Ralstonia sp. AU12-08*	P(β)	S9S0D4	WP_021192980.1	1201	Hamp+PAS+LOV+GGDEF+EAL	722
296	*Ralstonia sp. PBA*	P(β)	I9W2M2	WP_009523483.1	688	PAS+LOV+GGDEF+EAL	723
297	*Ralstonia syzygii R24*	P(β)	G3A8G2	CCA87536.1	1178	3PAS+LOV+GGDEF	724
298	*Ramilbacter tataouinensis DSM14655*	P(β)	F5Y2K4	YP_004618386.1	537	LOV+Kinase+RR	725
299	*Rubrivivax benzoatilyticus JA2*	P(β)	F3LSW2	WP_009857995.1	687	LOV+PAS+GGDEF+EAL	726
		P(β)	F3LLJ8	WP_009855783.1	856	2PAS+LOV+PAS+Kinase	727
300	*Rubrivivax gelatinosus NBRC 100245*	P(β)	I0HXU9	YP_005439335.1	877	Hamp+PAS+LOV+Kinase	728
	Rubrivivax gelatinosus NBRC 100246	P(β)	I0HQ12	YP_005436598.1	737	LOV+PAS+GGDEF+EAL	729
301	*Sulfuricella denitrificans skB26*	P(β)	S6AL83	YP_008546461.1	698	LOV+PAS+GGDEF+EAL	730
			S6AFR8	YP_008545773.1	677	LOV+PAS+GGDEF+EAL	731
302	*Thauera phenylacetica B4P*	P(β)	N6ZRB5	WP_004362938.1	864	HAMP+PAS+LOV+PAS+Kinase	732
			N6ZXB3	WP_004364285.1	954	HAMP+2PAS+LOV+PAS+Kinase	733
			N6YKT8	WP_004382816.1	738	Globin+LOV+GGDEF+EAL	734
303	*Thauera sp. 27*	P(β)	N6XKW8	WP_002937138.1	1133	Hamp+2PAS+LOV+PAS+Kinase	735
304	*Acidithiobacillus caldus ATCC 51756*	P(γ)	C6NR21	WP_004867363.1	913	GAF+LOV+PAS+GGDEF+EAL	736
	Acidithiobacillus caldus str. SM-1	P(γ)	F9ZQB1	YP_004747583.1	913	GAF+LOV+PAS+GGDEF+EAL	737
305	*Acidithiobacillus ferrooxidans ATCC 23270*	P(γ)	B7JB43	YP_002426126.1	1057	GAF+LOV+PAS+GAF+GGDEF+EAL	738

306	*Acidithiobacillus sp.GGI-221*	P(γ)	F8XM97	WP_009561169.1	677	LOV+PAS+GAF+GGDEF	739
307	*Acinetobacter brisouii ANC 4119*	P(γ)	N9AXM2	WP_004898317.1	859	GAF+PAS+LOV+GGDEF+EAL	740
308	*Acinetobacter lwoffii SH145*	P(γ)	D0SWD2	WP_004280312.1	859	GAF+LOV+PAS+GGDEF+EAL	741
	Acinetobacter lwoffii	P(γ)		WP_016806740.1	859	GAF+LOV+PAS+GGDEF+EAL	742
				WP_016806740.1	859	GAF+PAS+LOV+GGFEF+EAL	743
	Acinetobacter lwoffii CIP 70.31	P(γ)	N9H487	WP_005100328.1	537	LOV+GGDEF+EAL	744
	Acinetobacter lwoffii NCTC 5866 = CIP 64.10	P(γ)	N9H4F1	WP_005096907.1	859	GAF+PAS+LOV+GGDEF+EAL	745
	Acinetobacter lwoffii NIPH 478	P(γ)	N9HNY3	WP_005106935.1	859	GAF+PAS+LOV+GGDEF+EAL	746
			N9HEH7	WP_005107680.1	864	PAS+LOV+GGDEF+EAL	747
	Acinetobacter lwoffii NIPH 715	P(γ)	N8TVY4	WP_004730980.1	859	GAF+PAS+LOV+GGDEF+EAL	748
309	*Acinetobacter radioresistens DSM 6976*	P(γ)	K6W2B6	WP_005026623.1	855	GAF+PAS+LOV+GGDEF+EAL	749
	Acinetobacter radioresistens NIPH 2130	P(γ)	N9CGQ6	WP_005019674.1	855	GAF+PAS+LOV+GGDEF+EAL	750
	Acinetobacter radioresistens SH164	P(γ)	D0T5K2	WP_005019674.1	855	GAF+PAS+LOV+GGDEF	751
	Acinetobacter radioresistens SK82	P(γ)	C6RJR5	EET83911.1/WP_005019674.1	855	GAF+PAS+LOV+GGDEF+EAL	752
	Acinetobacter radioresistens WC-A-157	P(γ)	J4R2N6	WP_005406298.1	855	GAF+PAS+LOV+GGDEF+EAL	753
310	*Acinetobacter sp. ANC 3789*	P(γ)	N8VFZ2	WP_004749596.1	854	GAF+PAS+LOV+GGDEF+EAL	754
			N8VCG8	WP_004751822.1	846	GAF+PAS+LOV+GGDEF+EAL	755
311	*Acinetobacter sp. ANC 3994*	P(γ)	N8NWV8	WP_004649263.1	861	GAF+PAS+LOV+GGDEF+EAL	756
312	*Acinetobacter sp. CIP 101966*	P(γ)	N9QD69	WP_005262929.1	859	GAF+PAS+LOV+GGDEF+EAL	757
313	*Acinetobacter sp. CIP 102136*	P(γ)	N9PS41	WP_005251802.1	859	GAF+PAS+LOV+GGDEF+EAL	758
314	*Acinetobacter sp. CIP 51.11*	P(γ)	N9PKC5	WP_005246324.1	859	GAF+PAS+LOV+GGDEF+EAL	759
315	*Acinetobacter sp. CIP 53.82*	P(γ)	N9ML36	WP_005177235.1	857	GAF+PAS+LOV+GGDEF+EAL	760
316	*Acinetobacter sp. CIP 64.7*	P(γ)	N9QK63	WP_005266388.1	859	GAF+PAS+LOV+GGDEF+EAL	761
317	*Acinetobacter sp. CIP A162*	P(γ)	N8Q6A8	WP_004646969.1	859	GAF+PAS+LOV+GGDEF+EAL	762
318	*Acinetobacter sp. MDS7A*	P(γ)		WP_019837897.1	857	GAF+LOV+PAS+GGDEF+EAL	763
319	*Acinetobacter sp. NIPH 2171*	P(γ)	N9MLY1	WP_005234449.1	850	GAF+PAS+LOV+GGDEF+EAL	764
320	*Acinetobacter sp. NIPH 713*	P(γ)	N9KQ40	WP_005170912.1	861	GAF+PAS+LOV+GGDEF+EAL	765
321	*Acinetobacter sp. NIPH 899*	P(γ)	N8VGG5	WP_004783140.1	821	GAF+PAS+LOV+GGDEF+EAL	766
322	*Algicola sagamiensis*	P(γ)		WP_018692423.1	320	LOV+GGDEF	767
323	*Aliivibrio fischeri (Vibrio fischeri)*	P(γ)		WP_017019105.1	631	LOV+GGDEF	768
324	*Alishewanella agri BL06*	P(γ)	I9DPX4	WP_008985345.1	1590	PAS+GAF+PAS+LOV+GGDEF+EAL+RR	769
325	*Alishewanella jeotgali KCTC 22429*	P(γ)	H3ZFV5	WP_008950907.1	1590	PAS+GAF+PAS+LOV+GGDEF+EAL+RR	770
326	*Alteromonas macleodii AltDE1*	P(γ)	K7RA87	YP_006975689.1	959	PAS+LOV+Kinase+RR	771
	Alteromonas macleodii ATCC 27126	P(γ)	J9Y2J7	YP_006746817.1	958	PAS+LOV+Kinase+RR	772
	Alteromonas macleodii Black Sea 11	P(γ)	K0CV21	YP_006823794.1	959	PAS+LOV+Kinase+RR	773
	Alteromonas macleodii DSM 17117 DeePecotype	P(γ)	F2GAX1	YP_004426010.1	959	PAS+LOV+Kinase+RR	774
	Alteromonas macleodii English Channel 673	P(γ)	K0CM81	YP_006797832.1	958	LOV+PAS+Kinase+RR	775
	Alteromonas macleodii str. 'Aegean Sea MED64'	P(γ)	S5AQD7	AGP80766.1	959	PAS+LOV+Kinase+RR	776
	Alteromonas macleodii str. 'English Channel 615'	P(γ)	S5ABK9	YP_008191735.1	959	PAS+LOV+Kinase+RR	777
	Alteromonas macleodii str. 'Ionian Sea U4'	P(γ)	S5BBY3	YP_008170856.1	959	PAS+LOV+Kinase+RR	778
			S5B6H2	YP_008170856.1	959	PAS+LOV+Kinase+RR	779
	Alteromonas macleodii str. 'Ionian Sea U8'	P(γ)	S5BMV0	YP_008174998.1	959	PAS+LOV+Kinase+RR	780
327	*Alteromonas sp. SN2*	P(γ)	F5Z4R5	YP_004468204.1	956	PAS+LOV+Kinase+RR	781
328	*Arhodomonas aquaeolei*	P(γ)		WP_018716398.1	146	Short-LOV	782
				WP_018717064.1	848	GAF+LOV+PAS+GGDEF+EAL	783
329	*Bermanella marisrubri*	P(γ)	Q1N482	WP_007018054.1	938	LOV+ Kinase+ RR+RR	784
330	*Catenovulum agarivorans*	P(γ)		WP_016956198.1	1106	PAS+LOV+Kinase+RR	785
				WP_016956198.1	1106	2PAS+LOV+Kinase+RR	786
331	*Chromohalobacter salexigens DSM 3043*	P(γ)	Q1QUF0	YP_574607.1	836	PAS+LOV+PAS+GGDEF+EAL	787
			Q1QUF0	ABE59908.1/YP_574607.1	836	LOV+2PAS+ GGDEF+EAL	788
			Q1QU87	YP_574670.1	144	Short-LOV	789
332	*Dyella ginsengisoli*	P(γ)		WP_017460863.1	820	RR+PAS+LOV+GGDEF+EAL	790
				WP_017462527.1	823	LOV+PAS+GGDEF+EAL	791

				WP_017463133.1	1578	PAS+GAF+PAS+LOV+GGDEF+Globin+EAL	792
333	*Ectothiorhodospira sp.PHS-1*	P(γ)	H1G1Q6	WP_008931310.1	159	Short-LOV	793
			H1G08	WP_008930788.1	150	Short-LOV	794
334	*Fluoribacter dumoffii*	P(γ)		WP_010655036.1	357	LOV+Kinase	795
335	*Glaciecola nitratireducens JCM 12485*	P(γ)	G4QHD6	YP_004871760.1	408	LOV+SPOIIE	796
336	*Glaciecola pallidula DSM 14239 = ACAM 615*	P(γ)	K6ZHY4	WP_006010726.1	408	LOV+SPOIIE	797
337	*Hahella ganghwensis*	P(γ)		WP_020410026.1	1211	RR+PAS+LOV+GGDEF+EAL+RR	798
				WP_020406443.1	1654	HAMP+PAS+LOV+2PAS+Kinase+RR+Hpt	799
338	*Halomonas anticariensis FP35 = DSM 16096*	P(γ)	S2KPA1	WP_016414477.1	1070	RR+2PAS+LOV+GGDEF+EAL	800
			S2KJN8	WP_016418607.1	1329	GAF+PAS+LOV+GGDEF+EAL	801
			S2KLF6	WP_016417969.1	694	PAS+LOV+GGDEF+EAL	802
339	*Halomonas boliviensis LC1*	P(γ)	G9EBS6	WP_007112306.1	931	3PAS+LOV+GGDEF+EAL	803
			G9EDB9	WP_007113307.1	139	Short-LOV	804
340	*Halomonas jeotgali*	P(γ)		WP_017430872.1	139	Short-LOV	805
341	*Halomonas lutea*	P(γ)		WP_019020310.1	141	Short-LOV	806
				WP_019016980.1	856	GAF+LOV+PAS+GGDEF+EAL	807
				WP_019018880.1	857	GAF+LOV+PAS+GGDEF+EAL	808
342	*Halomonas smyrnensis*	P(γ)		WP_016856487.1	144	Short-LOV	809
				WP_016853871.1	1335	GAF+LOV+2PAS+GGDEF+EAL	810
				WP_016855043.1	851	GAF+LOV+PAS+GGDEF+EAL	811
343	*Halomonas sp. A3H3*	P(γ)		WP_022522746.1	144	Short-LOV	812
				WP_022521218.1	684	PAS+LOV+GGDEF+EAL	813
344	*Halomonas sp. GFAJ-1*	P(γ)	H0J093	WP_009097290.1	874	LOV+PAS+GGDEF+EAL	814
			H0J025	WP_009097057.1	145	Short-LOV	815
			H0J1I9	WP_009098247.1	815	2PAS+LOV+GGDEF+EAL	816
345	*Halomonas sp. KM-1*	P(γ)		WP_010628907.1	743	PAS+LOV+GGDEF+EAL	817
				WP_010627148.1	144	Short-LOV	818
				WP_010628900.1	1328	GAF+PAS+LOV+GGDEF+EAL	819
				WP_010627231.1	1157	PAS+LOV+GGDEF+EAL	820
				WP_010629458.1	830	PAS+LOV+GGDEF+EAL+RR	821
346	*Halomonas sp.HAL1*	P(γ)	G4FAF4	WP_008959245.1	709	PAS+LOV+2GGDEF	822
			G4F2H5	WP_008956503.1	927	3PAS+LOV+ GGDEF+EAL	823
			G4FBI0	WP_008959621.1	144	Short-LOV	824
347	*Halomonas sp.TD01*	P(γ)	F7SNC3	WP_009723241.1	816	2PAS+LOV+GGDEF+EAL	825
			F7SIY3	WP_009721710.1	145	Short-LOV	826
348	*Halomonas stevensii*	P(γ)		WP_016916582.1	145	Short-LOV	827
				WP_016915464.1	814	2PAS+LOV+GGDEF+EAL	828
				WP_016915854.1	1161	PAS+LOV+GGDEF+EAL	829
349	*Halomonas titanicae BH1*	P(γ)	L9UD19	WP_009286231.1	685	PAS+LOV+GGDEF+EAL	830
			L9U8I4	WP_009287681.1	171	Short-LOV	831
350	*Halomonas zhanjiangensis*	P(γ)		WP_018918585.1	149	Short-LOV	832
				WP_018917662.1	1323	GAF+PAS+LOV+GGDEF+EAL	833
351	*Halothiobacillus neapolitanus ATCC23641*	P(γ)	D0L135	YP_003263455.1	151	Short-LOV	834
352	*Idiomarinasp.A28L*	P(γ)	F7RZ10	WP_007420466.1	880	GAF+PAS+LOV+GGDEF+EAL	835
353	*Kangiella aquimarina*	P(γ)		WP_018623315.1	870	GAF+PAS+LOV+GGDEF+EAL	836
354	*Kangiella koreensis DSM 16069*	P(γ)	C7R7Z7	YP_003145547.1	887	GAF+PAS+LOV+GGDEF+EAL	837
355	*Kushneria aurantia*	P(γ)		WP_019951323.1	140	Short-LOV	838
356	*Lamprocystis purpurea*	P(γ)		WP_020503819.1	153	Short-LOV	839
				WP_020504715.1	152	Short-LOV	840
357	*Marichromatium purpuratum 984*	P(γ)	F9TYW6	WP_005222470.1	152	Short-LOV	841
			F9TWG1	WP_005220887.1	1118	3PAS+LOV+Kinase+RR	842
358	*Marinobacter adhaerens HP15*	P(γ)	E4PMK6	YP_005887842.1	552	LOV+GGDEF+EAL	843
359	*Marinobacter algicola DG893*	P(γ)	A6EZP7	CCA87536.1	858	GAF+PAS+LOV+GGDEF+EAL	844

360	*Marinobacter lipolyticus SM19*	P(γ)	R8B0L0	WP_012138208.1	887	GAF+PAS+LOV+GGDEF+EAL	845
361	*Marinobacter nanhaiticus D15-8W*	P(γ)	N6WWB0	WP_004580166.1	1520	CHER+PAS+LOV+PAS+GGDEF+EAL	846
			N6WWN8	WP_004580316.1	630	MASE1+LOV+GGDEF	847
362	*Methylobacter marinus*	P(γ)		WP_020158844.1	846	CONTROLLO	848
				WP_020157396.1	152	Short-LOV	849
363	*Methylobacter tundripaludum SV96*	P(γ)	G3J1D6	WP_006893485.1	738	RR+LOV+LOV+PAS+Kinase	850
364	*Methylococcus capsulatus*	P(γ)		WP_017365821.1	640	LOV+GGDEF+EAL	851
	Methylococcus capsulatus str. Bath	P(γ)	Q609J7	YP_113700.1	712	LOV+GGDEF+EAL	852
			Q607S2	YP_114126.1	853	PAS+LOV+3PAS+SPOIIE	853
365	*Methylomicrobium alcaliphilum DSM 19304*	P(γ)	G4T2M2	YP_004918336.1	164	Short-Lov	854
			G4T2L9	YP_004918333.1	148	Short-Lov	855
			G4T0F8	YP_004918135.1	1418	Kinase+2RR+LOV+4PAS+Kinase+RR	856
366	*Methylomicrobium buryatense*	P(γ)		WP_017840040.1	1418	Hpt+2RR+LOV+4PAS+Kinase	857
				WP_017842760.1	164	Short-LOV	858
				WP_017842762.1	148	Short-LOV	859
367	*Methylomonas methanica MC09*	P(γ)	G0A1Q1	YP_004512611.1	150	Short-LOV	860
368	*Methylomonas sp. MK1*	P(γ)		WP_020482233.1	151	Short-LOV	861
369	*Methylosarcina fibrata*	P(γ)		WP_020564946.1	840	CONTROLLO	862
370	*Methylovulum miyakonense*	P(γ)		WP_019864760.1	153	Short-LOV	863
371	*Nitrosococcus halophilus Nc4*	P(γ)	D5BVD1	YP_003525946.1|	149	Short-LOV	864
372	*Nitrosococcus oceani AFC27*	P(γ)	B6C5R4	WP_002813571.1	150	Short-LOV	865
	Nitrosococcus oceani ATCC 19707	P(γ)	Q3J6W8	YP_344958.1	150	Short-LOV	866
373	*Nitrosococcus watsoni C-113*	P(γ)	D8KC92	YP_003762084.1	150	Short-LOV	867
374	*Oceanimonas smirnovii*	P(γ)		WP_019936022.1	854	GAF+LOV+PAS+GGDEF+EAL	868
375	*Pseudoalteromonas citrea*	P(γ)		WP_010362109.1	1105	PAS+LOV+Kinase+RR	869
376	*Pseudoalteromonas flavipulchra*	P(γ)		WP_010607534.1	1103	PAS+LOV+PAS+Kinase+RR	870
377	*Pseudoalteromonas piscicida*	P(γ)		WP_010374796.1	1103	PAS+LOV+PAS+Kinase+RR	871
378	*Pseudoalteromonas rubra*	P(γ)		WP_010380647.1	1107	2PAS+LOV+Kinase+RR	872
379	*Pseudoalteromonas sp. BSi20495*	P(γ)	G7G480	WP_008137009.1	1098	PAS+LOV+Kinase+2RR	873
380	*Pseudoalteromonas sp. Bsw20308*	P(γ)	M5H4P7	WP_007376873.1	1098	PAS+LOV+Kinase+RR	874
381	*Pseudoalteromonas sp. NJ631*	P(γ)		WP_017218143.1	1103	PAS+LOV+PAS+Kinase+RR	875
382	*Pseudoalteromonas sp. SM9913*	P(γ)	E6RNZ1	YP_004068043.1	1101	PAS+LOV+Kinase+2EAL	876
383	*Pseudoalteromonas undina*	P(γ)		WP_010391230.1	1101	PAS+LOV+Kinase+RR	877
384	*Pseudomonas*	P(γ)		WP_010206458.1	155	Short-LOV	878
				WP_018927172.1	158	Short-LOV	879
385	*Pseudomonas aeruginosa B136-33*	P(γ)	M9S7A7	YP_007711618.1	156	Short-LOV	880
386	*Pseudomonas agarici*	P(γ)		WP_017130703.1	154	Short-LOV	881
387	*Pseudomonas alcaligenes*	P(γ)		WP_021217027.1	147	Short-LOV	882
				WP_021699549.1	146	Short-LOV	883
				WP_021218606.1	1234	PAS+LOV+2PAS+Kinase+RR	884
388	*Pseudomonas alcaliphila*	P(γ)		WP_017678393.1	1274	MASE1+PAS+LOV+GGDEF+EAL	885
389	*Pseudomonas amygdali*	P(γ)		WP_010206443.1	452	LOV+Kinase+RR	886
390	*Pseudomonas brassicacearum NFM421*	P(γ)	F2KL83	YP_004356128.1	157	Short-LOV	887
391	*Pseudomonas chlororaphis O6*	P(γ)	I4XX85	WP_009047027.1	157	Short-LOV	888
392	*Pseudomonas chlororaphis subsp. aureofaciens 30-84*	P(γ)	J2EWK4	WP_009042217.1	157	Short-LOV	889
393	*Pseudomonas extremaustralis*	P(γ)		WP_010566244.1	155	Short-LOV	890
394	*Pseudomonas fluorescens*	P(γ)		WP_017338856.1	150	Short-LOV	891
				WP_017336579.1	158	Short-LOV	892
				WP_016987018.1	158	Short-LOV	893
				WP_019691929.1	151	Short-LOV	894
				WP_016976173.1	155	Short-LOV	895
				WP_017531111.1	155	Short-LOV	896
				WP_017135085.1	155	Short-LOV	897

				WP_019689251.1	733	RR+2PAS+LOV+GGDEF+EAL	898
	Pseudomonas fluorescens A506	P(γ)	I2BPA2	YP_006323676.1	525	LOV+Kinase+RR	899
			I2BQI5	YP_006325858.1	155	Short-LOV	900
	Pseudomonas fluorescens BBc6R8	P(γ)	K1AY44	WP_003208484.1	155	Short-LOV	901
	Pseudomonas fluorescens BRIP34879	P(γ)	L7H7A8	WP_003236565.1	524	LOV+Kinase+RR	902
	Pseudomonas fluorescens F113	P(γ)	G8QB22	YP_005210257.1	157	Short-LOV	903
	Pseudomonas fluorescens PF0-1	P(γ)	Q3KHW	YP_346628.1	158	Short-LOV	904
	Pseudomonas fluorescens PF-5	P(γ)	Q4KI48	YP_258085.1	152	Short-LOV	905
	Pseudomonas fluorescens Q2-87	P(γ)	J2F5H6	WP_003178071.1	158	Short-LOV	906
	Pseudomonas fluorescens Q8r1-96	P(γ)	I4JY51	YP_004356128.1	157	Short-LOV	907
	Pseudomonas fluorescens R124	P(γ)	K0WFQ8	WP_003224839.1	153	Short-LOV	908
	Pseudomonas fluorescens R124	P(γ)	K0WYP8	WP_003221745.1	158	Short-LOV	909
	Pseudomonas fluorescens SBW25	P(γ)	C3K1W0	YP_002874656.1	155	Short-LOV	910
	Pseudomonas fluorescens SS101	P(γ)	I4K663	WP_003194067.1	155	Short-LOV	911
	Pseudomonas fluorescens SS101	P(γ)	I4K738	WP_003190461.1	531	LOV+Kinase+RR	912
	Pseudomonas fluorescens WH6	P(γ)	E2XTK3	WP_003174467.1	519	LOV+Kinase+RR	913
			E2XXX1	WP_003176023.1	155	Short-LOV	914
395	*Pseudomonas fragi*	P(γ)		WP_010656027.1	157	Short-LOV	915
				WP_016781829.1	155	Short-LOV	916
396	*Pseudomonas fulva 12-X*	P(γ)	F6ACW6	YP_004473266.1	534	LOV+Kinase+RR	917
			F6AEJ4	YP_004473421.1	150	Short-LOV	918
397	*Pseudomonas fuscovaginae*	P(γ)		WP_010452910.1	519	LOV+RR	919
				WP_010445203.1	154	Short-LOV	920
				WP_017904260.1	154	Short-LOV	921
398	*Pseudomonas luteola*	P(γ)		WP_019367061.1	1072	RR+PAS+LOV+PAS+GGDEF+EAL+RR	922
399	*Pseudomonas mandelii*	P(γ)		WP_010460270.1	150	Short-LOV	923
				WP_010463226.1	159	Short-LOV	924
				WP_019581149.1	158	Short-LOV	925
400	*Pseudomonas mendocina*	P(γ)		WP_017360623.1	1274	MASE1+LOV+2PAS+GGDEF+EAL	926
				WP_021487701.1	1274	MASE1+LOV+2PAS+GGDEF+EAL	927
	Pseudomonas mendocina DLHK	P(γ)	J7UFV0	WP_004373223.1	1254	MASE1+LOV+2PAS+GGDEF+EAL	928
			J7TJL5	WP_003245955.1	148	Short-LOV	929
	Pseudomonas mendocina NK-01	P(γ)	F4DSG2	YP_004381369.1	148	Short-LOV	930
	Pseudomonas mendocina NK-02	P(γ)	F4DZM3	YP_004378203.1	1274	MASE1+PAS+LOV+PAS+GGDEF+EAL	931
	Pseudomonas mendocina ymp	P(γ)	A4XP68	YP_001185866.1	1254	MASE1+LOV+2PAS+ GGDEF+EAL	932
	Pseudomonas mendocina ymp	P(γ)	A4XXH1	YP_001188769.1	148	Short-LOV	933
401	*Pseudomonas monteilii*	P(γ)		WP_016715187.1	148	Short-LOV	934
402	*Pseudomonas nitroreducens*	P(γ)		WP_017519191.1	150	Short-LOV	935
403	*Pseudomonas plecoglossicida NB2011*	P(γ)	S2JT44	WP_016395429.1	142	Short-LOV	936
404	*Pseudomonas poae RE*1-1-14*	P(γ)	M4JYC7	YP_007397135.1	524	LOV+Kinase+RR	937
			M4K6Y0	YP_007398848.1	155	Short-LOV	938
405	*Pseudomonas protegens CHA0*	P(γ)	R4RAL1	YP_007998266.1	152	Short-LOV	939
406	*Pseudomonas pseudoalcaligenes CECT 5344*	P(γ)	I7J111	WP_003462439.1	148	Short-LOV	940
407	*Pseudomonas pseudoalcaligenes KF707*	P(γ)	L8MD26	WP_003449890.1	1068	RR+2PAS+LOV+GGDEF+EAL	941
			L8MMM4	WP_004422222.1	1895	PAS+GAF+PAS+GAF+PAS+LOV+GGDEF+EAL+RR	942
			L8MMQ1	WP_003456550.1	148	Short-LOV	943
408	*Pseudomonas psychrophila*	P(γ)		WP_019410857.1	157	Short-LOV	944
409	*Pseudomonas psychrotolerans L19*	P(γ)	H0JJH7	WP_007162860.1	537	LOV+Kinase+RR	945
			H0J8E5	WP_007158985.1	538	LOV+Kinase+RR	946
410	*Pseudomonas putida*	P(γ)		WP_019097373.1	148	Short-LOV	947
				WP_019752532.1	142	Short-LOV	948
				WP_021782295.1	148	Short-LOV	949
				WP_021784280.1	142	Short-LOV	950

	Pseudomonas putida (Arthrobacter siderocapsulatus)	P(γ)	B5SNZ7		148	Short-LOV	951
	Pseudomonas putida BIRD-1	P(γ)	E4R4W4	YP_005932103.1	142	Short-LOV	952
	Pseudomonas putida BIRD-2	P(γ)	E4RI35	YP_005930752.1	148	Short-LOV	953
	Pseudomonas putida CSV86	P(γ)	L1M649	WP_009395102.1	153	Short-LOV	954
	Pseudomonas putida CSV86	P(γ)	L1M6G2	WP_009395400.1	532	LOV+Kinase+RR	955
	Pseudomonas putida DOT-T1E	P(γ)	I7C6T3	YP_001269793.1	142	Short-LOV	956
	Pseudomonas putida F1	P(γ)	A5W8Z9	YP_001269793.1	142	Short-LOV	957
			A5W4T2	YP_001268326.1	148	Short-LOV	958
	Pseudomonas putida GB-1	P(γ)	B0KGV4	YP_001670835.1	142	Short-LOV	959
	Pseudomonas putida group	P(γ)		WP_016713473.1	142	Short-LOV	960
	Pseudomonas putida H8234	P(γ)	R9V1Y1	YP_008094059.1	148	Short-LOV	961
				YP_008097264.1	142	Short-LOV	962
	Pseudomonas putida HB3267	P(γ)	L0FJK3	YP_007229131.1	148	Short-LOV	963
			L0FRT1	YP_007231313.1	142	Short-LOV	964
	Pseudomonas putida KT2440	P(γ)	Q88E39	NP_746738.1	142	Short-LOV	965
			Q88JB0	NP_744883.1	151	Short-LOV	966
	Pseudomonas putida LS46	P(γ)	M7R4B9	YP_001269793.1	142	Short-LOV	967
			M7R1E0	WP_003254000.1	148	Short-LOV	968
	Pseudomonas putida NBRC 14164	P(γ)		YP_008113666.1	148	Short-LOV	969
				YP_008111769.1	142	Short-LOV	970
	Pseudomonas putida ND6	P(γ)	I3USM9	YP_001269793.1	142	Short-LOV	971
	Pseudomonas putida S11	P(γ)	J8RS26	WP_003260703.1	142	Short-LOV	972
			J8RYG9	WP_003257626.1	148	Short-LOV	973
	Pseudomonas putida S16	P(γ)	F8G2U7	YP_004701648.1	148	Short-LOV	974
			F8G5Y7	YP_004703866.1	142	Short-LOV	975
	Pseudomonas putida TRO1	P(γ)	N9UJ18	YP_001269793.1	142	Short-LOV	976
			N9UCY1	WP_003254000.1	148	Short-LOV	977
	Pseudomonas putida W619	P(γ)	B1JAC4	YP_001750029.1	148	Short-LOV	978
			B1J385	YP_001747686.1	152	Short-LOV	979
411	*Pseudomonas resinovorans NBRC 106553*	P(γ)	S6ANF2	YP_008105095.1	148	Short-LOV	980
412	*Pseudomonas savastanoi pv. savastanoi NCPPB 3335*	P(γ)	D7HZQ3	WP_002553497.1	534	LOV+Kinase+RR	981
413	*Pseudomonas sp. 313*	P(γ)		WP_017641826.1	501	LOV+Kinase+RR	982
				WP_017642205.1	538	LOV+Kinase+RR	983
414	*Pseudomonas sp. 45MFCol3.1*	P(γ)		WP_019647677.1	158	Short-LOV	984
415	*Pseudomonas sp. Ag1*	P(γ)	J0YEQ9	WP_008432316.1	155	Short-LOV	985
416	*Pseudomonas sp. CBZ-4*	P(γ)		WP_017735040.1	155	Short-LOV	986
417	*Pseudomonas sp. CF149*	P(γ)		WP_019824960.1	155	Short-LOV	987
418	*Pseudomonas sp. CF150*	P(γ)	S6KFW9	WP_020302025.1	155	Short-LOV	988
				WP_020303213.1	510	LOV+Kinase+RR	989
419	*Pseudomonas sp. CF161*	P(γ)		WP_020297180.1	157	Short-LOV	990
			S6J587	WP_020296253.1	300	LOV+GGDEF	991
420	*Pseudomonas sp. CFII64*	P(γ)	S6GUX3	WP_020294427.1	153	Short-LOV	992
			S6HC34	WP_020289714.1	759	GAF+LOV+GGDEF+EAL	993
421	*Pseudomonas sp. CFII68*	P(γ)	S6JL65	WP_018604186.1	157	Short-LOV	994
422	*Pseudomonas sp. CFT9*	P(γ)		WP_019821480.1	510	LOV+Kinase+RR	995
423	*Pseudomonas sp. Chol1*	P(γ)	K5Y919	WP_008568795.1	148	Short-LOV	996
424	*Pseudomonas sp. EGD-AK9*	P(γ)		WP_021443742.1	1251	MASE1+LOV+3PAS+GGDEF+EAL	997
				WP_021443846.1	145	Short-LOV	998
425	*Pseudomonas sp. G5(2012)*	P(γ)	S2FLS8	WP_020796677.1	154	Short-LOV	999
426	*Pseudomonas sp. GM102*	P(γ)	J2MPF7	WP_007901653.1	158	Short-LOV	1000
427	*Pseudomonas sp. GM16*	P(γ)	J2VEZ9	WP_007914248.1	158	Short-LOV	1001
428	*Pseudomonas sp. GM17*	P(γ)	J2VFQ9	WP_007929819.1	157	Short-LOV	1002
429	*Pseudomonas sp. GM21*	P(γ)	J2NUZ8	WP_007944641.1	158	Short-LOV	1003

430	*Pseudomonas sp. GM24*	P(γ)	J2QD44	WP_007914248.1	158	Short-LOV	1004
431	*Pseudomonas sp. GM25*	P(γ)	J2PQ86	WP_007957219.1	158	Short-LOV	1005
432	*Pseudomonas sp. GM30*	P(γ)	J2Q414	WP_007968050.1	158	Short-LOV	1006
433	*Pseudomonas sp. GM33*	P(γ)	J3FZ72	WP_007970313.1	158	Short-LOV	1007
434	*Pseudomonas sp. GM41(2012)*	P(γ)	J2ZFJ0	WP_008150687.1	158	Short-LOV	1008
435	*Pseudomonas sp. GM48*	P(γ)	J2S6K2	WP_007986762.1	158	Short-LOV	1009
436	*Pseudomonas sp. GM49*	P(γ)	J3GAS8	WP_008000603.1	158	Short-LOV	1010
437	*Pseudomonas sp. GM50*	P(γ)	J3GK37	WP_008008657.1	158	Short-LOV	1011
438	*Pseudomonas sp. GM60*	P(γ)	J2TXN8	WP_008026981.1	158	Short-LOV	1012
439	*Pseudomonas sp. GM67*	P(γ)	J2UXA4	WP_008037334.1	158	Short-LOV	1013
440	*Pseudomonas sp. GM74*	P(γ)	J3C1S1	WP_008046230.1	156	Short-LOV	1014
441	*Pseudomonas sp. GM78*	P(γ)	J2YG82	WP_008054412.1	154	Short-LOV	1015
442	*Pseudomonas sp. GM79*	P(γ)	J3IFK2	WP_008070532.1	158	Short-LOV	1016
443	*Pseudomonas sp. GM80*	P(γ)	J3IFB0	WP_008084980.1	158	Short-LOV	1017
444	*Pseudomonas sp. GM84*	P(γ)	J3E0M0	WP_008096700.1	142	Short-LOV	1018
445	*Pseudomonas sp. HPB0071*	P(γ)	N2J3H5	WP_010797478.1	558	LOV+Kinase+RR	1019
			N2J7R2	WP_010795793.1	566	LOV+Kinase+RR	1020
			N2J7R2	WP_010795793.1	566	LOV+Kinase+RR	1021
446	*Pseudomonas sp. HYS*	P(γ)		WP_010223533.1	153	Short-LOV	1022
447	*Pseudomonas sp. Lz4W*	P(γ)	M5QYU4	WP_003439967.1	157	Short-LOV	1023
448	*Pseudomonas sp. M47T1*	P(γ)	I4N1D2	WP_008371185.1	152	Short-LOV	1024
449	*Pseudomonas sp. PAMC 25886*	P(γ)		WP_010176982.1	155	Short-LOV	1025
450	*Pseudomonas sp. PAMC 26793*	P(γ)		WP_017479546.1	145	Short-LOV	1026
451	*Pseudomonas sp. PAMC 26793*	P(γ)		WP_017476025.1	155	Short-LOV	1027
452	*Pseudomonas sp. S9*	P(γ)		WP_010486409.1	152	Short-LOV	1028
453	*Pseudomonas sp. UW4*	P(γ)	K9NJA7	YP_007029976.1	148	Short-LOV	1029
			K9NEI7	YP_007027827.1	156	Short-LOV	1030
454	*Pseudomonas sp.TJI-51*	P(γ)	F0EAJ0	WP_009685517.1	382	LOV+Kinase	1031
			F0E265	WP_009682668.1	142	Short-LOV	1032
			F0EAK5	WP_009685532.1	148	Short-LOV	1033
455	*Pseudomonas stutzeri*	P(γ)		WP_019339020.1	148	Short-LOV	1034
				WP_021206844.1	148	Short-LOV	1035
	Pseudomonas stutzeri A1501	P(γ)	A4VKZ3	YP_001172486.1	146	Short-LOV	1036
	Pseudomonas stutzeri CCUG 29243	P(γ)	I4CTM3	YP_006457853.1	146	Short-LOV	1037
	Pseudomonas stutzeri DSM 4166	P(γ)	F2MYU4	YP_005938635.1	146	Short-LOV	1038
	Pseudomonas stutzeri DSM 5190	P(γ)	F8H9C5	YP_004714236.1	148	Short-LOV	1039
	Pseudomonas stutzeri KOS6	P(γ)	K6CEY0	WP_003296418.1	1064	RR+2PAS+LOV+GGDEF+EAL	1040
	Pseudomonas stutzeri NF13	P(γ)	M2UPJ8	WP_003300039.1	146	Short-LOV	1041
	Pseudomonas stutzeri RCH2	P(γ)	L0GJI2	YP_007240543.1	148	Short-LOV	1042
	Pseudomonas stutzeri TS44	P(γ)	I4JMH9	WP_003287724.1	148	Short-LOV	1043
456	*Pseudomonas synxantha BG33R*	P(γ)	I4L697	WP_005791275.1	155	Short-LOV	1044
457	*Pseudomonas syringae*	P(γ)		WP_010432789.1	534	LOV+RR	1045
				WP_010404642.1	259	LOV+Kinase	1046
				WP_017279143.1	502	LOV+Kinase+RR	1047
				WP_017702380.1	502	LOV+Kinase+RR	1048
				WP_017709231.1	502	LOV+Kinase+RR	1049
				WP_019334300.1	502	LOV+Kinase+RR	1050
				WP_020322550.1	534	LOV+Kinase+RR	1051
				WP_020304842.1	534	LOV+Kinase+RR	1052
				WP_020340339.1	500	LOV+Kinase+RR	1053
				WP_020354720.1	534	LOV+Kinase+RR	1054
				WP_017703627.1	502	LOV+Kinase+RR	1055
				WP_017683368.1	534	LOV+Kinase+RR	1056

	Pseudomonas syringae BRIP34876	P(γ)	L7FTW7	WP_003426528.1	534	LOV+Kinase+RR	1057
			L7FQF5	WP_003429130.1	647	LOV+PAS+Kinase+RR	1058
	Pseudomonas syringae BRIP34881	P(γ)	L7G1B0	WP_003426528.1	534	LOV+Kinase+RR	1059
			L7GCE7	WP_003429130.1	647	LOV+PAS+Kinase+RR	1060
	Pseudomonas syringae BRIP39023	P(γ)	L7GTP5	WP_003435337.1	502	LOV+Kinase+RR	1061
	Pseudomonas syringae Cit 7	P(γ)	F3H4Y3	WP_003370902.1	266	LOV+Kinase	1062
458	*Pseudomonas syringae pv. aceris str.M302273PT*	P(γ)	F3JEE1	WP_003401108.1	502	LOV+Kinase+RR	1063
459	*Pseudomonas syringae pv. actinidiae ICMP 19068*	P(γ)	S6P6T0	WP_003379535.1	534	LOV+Kinase+RR	1064
	Pseudomonas syringae pv. actinidiae ICMP 19070	P(γ)	S6R1K7	WP_003379535.1	534	LOV+Kinase+RR	1065
	Pseudomonas syringae pv. actinidiae ICMP 19071	P(γ)	S6MK99	WP_003379535.1	534	LOV+Kinase+RR	1066
	Pseudomonas syringae pv. actinidiae ICMP 19072	P(γ)	S6N632	WP_003379535.1	534	LOV+Kinase+RR	1067
	Pseudomonas syringae pv. actinidiae ICMP 19103	P(γ)	S6MYJ2	WP_003379535.1	534	LOV+Kinase+RR	1068
	Pseudomonas syringae pv. actinidiae ICMP 19104	P(γ)	S6PWM6	WP_003379535.1	534	LOV+Kinase+RR	1069
	Pseudomonas syringae pv. actinidiae ICMP 9855	P(γ)	S6SDG5	WP_003379535.1	534	LOV+Kinase+RR	1070
	Pseudomonas syringae pv. Actinidiae str.M302091	P(γ)	F3I1F9	*WP_003379535.1*	534	LOV+Kinase+RR	1071
460	*Pseudomonas syringae pv. aesculi str. 0893_23*	P(γ)	F3DLV	WP_005730386.1	446	LOV+Kinase+RR	1072
461	*Pseudomonas syringae pv. avellanae str. ISPaVe013*	P(γ)	K2TLM0	WP_003410607.1	502	LOV+Kinase+RR	1073
			K2TFJ1	WP_003416783.1	534	LOV+Kinase+RR	1074
462	*Pseudomonas syringae pv. glycinea str. B076*	P(γ)	E7P672	/WP_004662206.1	534	LOV+Kinase+RR	1075
	Pseudomonas syringae pv. glycinea str. race 4	P(γ)	E7PNY8	WP_004662206.1	534	LOV+Kinase+RR	1076
463	*Pseudomonas syringae pv. japonica str. M301072PT*	P(γ)	F3FF68	WP_003314892.1	534	LOV+Kinase	1077
464	*Pseudomonas syringae pv. maculicola str. ES4326*	P(γ)	F3HHL5	WP_007249531.1	534	LOV+Kinase+RR	1078
			F3HHL5	WP_007249531.1	534	LOV+Kinase+RR	1079
465	*Pseudomonas syringae pv. mori str. 301020*	P(γ)	F3F0F6	WP_005757687.1	254	LOV+Kinase	1080
466	*Pseudomonas syringae pv. morsprunorum str. M302280PT*	P(γ)	F3DZS	WP_005739418.1	534	LOV+Kinase	1081
467	*Pseudomonas syringae pv. oryzae str. 1_6*	P(γ)	F2ZCS	WP_005886808.1	534	LOV+Kinase+RR	1082
468	*Pseudomonas syringae pv. phaseolicola 1448A*	P(γ)	Q48IV1	YP_274680.1	507	LOV+Kinase+RR	1083
469	*Pseudomonas syringae pv. pisi str. 1704B*	P(γ)	F3GB29	WP_003344947.1	534	LOV+Kinase	1084
470	*Pseudomonas syringae pv. syringae B64*	P(γ)	L8NFV2	WP_003426528.1	534	LOV+Kinase+RR	1085
	Pseudomonas syringae pv. syringae B728a	P(γ)	Q4ZSY3	YP_235777.1	534	LOV+Kinase+RR	1086
471	*Pseudomonas syringae pv. tabaci ATCC 11528*	P(γ)	F3JTS8	WP_005777149.1	534	LOV+Kinase+RR	1087
472	*Pseudomonas syringae pv. theae ICMP 3923*	P(γ)	S6P4Z2	WP_003379535.1	534	LOV+Kinase+RR	1088
473	*Pseudomonas syringae pv. tomato DC3000*	P(γ)	Q881J7	NP_792694.1	534	LOV+Kinase+RR	1089
	Pseudomonas syringae pv. tomato T1	P(γ)	E2M7Z9	EEB61765.1/NP_792694.1	534	LOV+Kinase	1090
474	*Pseudomonas tolaasii*	P(γ)		WP_017254000.1	155	Short-LOV	1091
				WP_016971065.1	155	Short-LOV	1092
475	*Pseudomonas veronii*	P(γ)		WP_017847767.1	155	Short-LOV	1093
476	*Pseudomonas viridiflava UASWS0038*	P(γ)	K6BMA6	WP_004885117.1	151	Short-LOV	1094
			K6BTG2	WP_004882331.1	503	LOV+Kinase+RR	1095
477	*Rheinheimera sp. A13L*	P(γ)	F7P0A2	WP_008900325.1	1195	RR+GAF+PAS+LOV+GGDEF+EAL	1096
478	*Rhodanobacter fulvus Jip2*	P(γ)	I4VRA7	WP_007081336.1	813	RR+PAS+LOV+GGDEF+EAL	1097
479	*Rhodobacter sphaeroides WS8N*	P(γ)	F5M2M2	WP_002719387.1	176	Short-LOV	1098
480	*Salinisphaera shabanensis E1L3A*	P(γ)	F7QDZ2	WP_006915464.1	333	LOV+GGDEF	1099
481	*Serratia fonticola*	P(γ)		WP_021805398.1	310	LOV+GGDEF	1100
482	*Serratia plymuthica AS9*	P(γ)	G0B7B1	YP_004500367.1	310	LOV+GGDEF	1101
483	*Serratia sp.AS12*	P(γ)	G0BP57	/YP_004500367.1	310	LOV+GGDEF	1102
484	*Serratia sp.AS13*	P(γ)	G0C2A2	/YP_004500367.1	310	LOV+GGDEF	1103
485	*Shewanella baltica BA175*	P(γ)	G0AVH5	YP_006022696.1	1215	MASE+CHASE+PAS+LOV+GGDEF+EAL	1104
	Shewanella baltica OS 117	P(γ)	G0DM57	YP_006035465.1	1215	MASE+CHASE+PAS+LOV+GGDEF+EAL	1105
	Shewanella baltica OS 183	P(γ)	H1YLW6	EHQ13065.1/YP_006022696.1	1215	MASE+CHASE+PAS+LOV+GGDEF+EAL	1106
	Shewanella baltica OS185	P(γ)	A6WU43	YP_001368395.1	1215	MASE1+CHASE+PAS+LOV+GGDEF+EAL	1107
	Shewanella baltica OS223	P(γ)	B8ECQ3	YP_002360038.1	1215	MASE1+CHASE+PAS+LOV+GGDEF+EAL	1108
486	*Shewanella oneidensis MR-1*	P(γ)	K4PU33	YP_007001418.1	1181	MASE1+CHASE+PAS+LOV+GGDEF+EAL	1109

487	*Shewanella putrefaciens CN-32*	P(γ)	A4YC79	YP_001185361.1	1216	MASE1+CHASE+PAS+LOV+GGDEF+EAL	1110
488	*Shewanella sp. ANA-3*	P(γ)	A0L2H7	YP_871652.1	1228	MASE1+CHASE+PAS+LOV+GGDEF+EAL	1111
489	*Shewanella sp. HN_41*	P(γ)	F7RU72	WP_007652082.1	1209	MASE+CHASE+PAS+LOV+GGDEF+EAL	1112
490	*Shewanella sp. W3-18-1*	P(γ)	A1RE55	YP_961504.1	1216	MASE1+CHASE+PAS+LOV+GGDEF+EAL	1113
491	*Sphingomonas elodea*	P(γ)		WP_010545260.1	186	Short-LOV	1114
492	*Spiribacter salinus M19-40*	P(γ)	R4VQP9	YP_008047228.1	151	Short-LOV	1115
493	*Thalassolituus oleivorans MIL-1*	P(γ)	M5DNS9	YP_007681337.1	155	Short-LOV	1116
494	*Thioalkalimicrobium aerophilum AL3*	P(γ)	G4D8R7	WP_006459544.1	147	Short-LOV	1117
495	*Thioalkalimicrobium cyclicum DSM 14477*	P(γ)	F6D9J9	YP_004536435.1	148	Short-LOV	1118
496	*Thioalkalivibrio*	P(γ)		WP_019024324.1	147	Short-LOV	1119
				WP_018864256.1	147	Short-LOV	1120
				WP_018139391.1	147	Short-LOV	1121
				WP_018174373.1	147	Short-LOV	1122
				WP_018861429.1	147	Short-LOV	1123
				WP_018650214.1	143	Short-LOV	1124
				WP_018169641.1	143	Short-LOV	1125
				WP_018867397.1	130	Short-LOV	1126
				WP_018877756.1	130	Short-LOV	1127
				WP_017942490.1	143	Short-LOV	1128
				WP_018140537.1	143	Short-LOV	1129
				WP_019589553.1	143	Short-LOV	1130
				WP_018870567.1	143	Short-LOV	1131
497	*Thioalkalivibrio nitratireducens DSM 14787*	P(γ)	L0DYC1	YP_007218080.1	150	Short-LOV	1132
498	*Thioalkalivibrio sp. AKL11*	P(γ)		WP_018939800.1	147	Short-LOV	1133
499	*Thioalkalivibrio sp. AKL12*	P(γ)		WP_018951723.1	143	Short-LOV	1134
500	*Thioalkalivibrio sp. AKL17*	P(γ)		WP_018947690.1	143	Short-LOV	1135
501	*Thioalkalivibrio sp. AKL6*	P(γ)		WP_018145821.1	143	Short-LOV	1136
502	*Thioalkalivibrio sp. ALE23*	P(γ)		WP_019022854.1	609	MASE1+LOV+GGDEF	1137
503	*Thioalkalivibrio sp. ALE31*	P(γ)		WP_018875372.1	130	Short-LOV	1138
504	*Thioalkalivibrio sp. ALJ15*	P(γ)		WP_020146677.1	147	Short-LOV	1139
505	*Thioalkalivibrio sp. ALJ16*	P(γ)		WP_018872447.1	143	Short-LOV	1140
506	*Thioalkalivibrio sp. ALJ17*	P(γ)		WP_018953828.1	1218	RR+PAS+GAF+LOV+2PAS+Kinase+RR	1141
507	*Thioalkalivibrio sp. ALJ24*	P(γ)		WP_018936177.1	147	Short-LOV	1142
				WP_018936260.1	625	MASE1+LOV+GGDEF	1143
508	*Thioalkalivibrio sp. ALJT*	P(γ)		WP_019626073.1	143	Short-LOV	1144
509	*Thioalkalivibrio sp. ALMg11*	P(γ)		WP_018949625.1	143	Short-LOV	1145
510	*Thioalkalivibrio sp. ALSr1*	P(γ)		WP_019643012.1	143	Short-LOV	1146
				WP_019643090.1	639	MASE1+LOV+GGDEF	1147
511	*Thioalkalivibrio sp. HL-Eb18*	P(γ)		WP_017926630.1	147	Short-LOV	1148
512	*Thioalkalivibrio sp. HL-EbGR7*	P(γ)	B8GRG0	YP_002513501.1	148	Short-LOV	1149
513	*Thioalkalivibrio sp. K90mix*	P(γ)	D3S9U5	YP_003460536.1	147	Short-LOV	1150
514	*Thioalkalivibrio thiocyanoxidans ARh4*	P(γ)	G4DMF8	WP_006749018.1	150	Short-LOV	1151
515	*Thiocapsa marina5811*	P(γ)	F9U8V	WP_007192233.1	153	Short-LOV	1152
516	*Thiocystis violascens ATCC 17096*	P(γ)	I3Y8C2	YP_006413365.1	515	LOV+PAS+Kinase	1153
517	*Thioflavicoccus mobilis 8321*	P(γ)	L0GV52	YP_007243337.1	155	Short-LOV	1154
518	*Thiomicrospira denitrificans ATCC 33889*	P(γ)	Q30NS0	YP_394596.1	240	HTH+ LOV	1155
519	*Thiomicrospira halophila*	P(γ)		WP_019895319.1	151	Short-LOV	1156
520	*Thiorhodospira sibirica ATCC 700588*	P(γ)	G4E1Z1	WP_006786321.1	1042	LOV+4PAS+Kinase+RR	1157
521	*Thiorhodovibrio sp. 970*	P(γ)	H8Z429	WP_009150501.1	155	Short-LOV	1158
522	*Thiothrix nivea DSM 5205*	P(γ)	I3BYI4	WP_002710300.1	158	Short-LOV	1159
523	*Vibrio alginolyticus NBRC 15630 = ATCC 17749*	P(γ)		YP_008535342.1	1595	HAMP+PAS+LOV+2PAS+Kinase+RR+Hpt	1160
524	*Vibrio fischeri ATCC 700601*	P(γ)	Q5E662	YP_204372.1	631	LOV+GGDEF	1161
	Vibrio fischeri MJ11	P(γ)	B5FD75	YP_002155793.1	631	LOV+GGDEF	1162

	Vibrio fischeri SR5	**P(γ)**	H1QYA1	WP_005418693.1	631	LOV+GGDEF	1163
525	*Vibrio parahaemolyticus 16*	**P(γ)**	B8KBV9	WP_005474670.1	1594	HAMP+PAS+LOV+2PAS+Kinase+RR+Hpt	1164
526	*Vibrio proteolyticus*	**P(γ)**		WP_021706469.1	1600	HAMP+PAS+LOV+2PAS+Kinase+RR+Hpt	1165
527	*Xanthomonas albilineans GPE PC73*	**P(γ)**	D2UAU2	YP_003376125.1	544	LOV+Kinase+RR	1166
528	*Xanthomonas arboricola*	**P(γ)**		WP_016902009.1	520	LOV+Kinase+RR	1167
529	*Xanthomonas axonopodis*	**P(γ)**		WP_016851550.1	540	LOV+Kinase+RR	1168
				WP_017173235.1	356	LOV+Kinase	1169
				WP_017161173.1	334	LOV+Kinase	1170
				WP_017159283.1	540	LOV+Kinase+RR	1171
				WP_017157089.1	540	LOV+Kinase+RR	1172
530	*Xanthomonas axonopodis pv. citri str.306*	**P(γ)**	Q8PJH	NP_642870.1	540	LOV+Kinase+RR	1173
531	*Xanthomonas axonopodis pv. citrumelo F1*	**P(γ)**	G2LWN4	YP_004852094.1	540	LOV+Kinase+RR	1174
532	*Xanthomonas axonopodis pv. malvacearum str. GSPB1386*	**P(γ)**	K8GB13	WP_005914055.1	540	LOV+Kinase+RR	1175
	Xanthomonas axonopodis pv. malvacearum str. GSPB2388	**P(γ)**	K8G4G5	WP_005914055.1	540	LOV+Kinase+RR	1176
533	*Xanthomonas axonopodis pv. punicae str. LMG 859*	**P(γ)**	H1XH4	WP_005929141.1	540	LOV+Kinase+RR	1177
534	*Xanthomonas campestris*	**P(γ)**		WP_010378213.1	541	LOV+Kinase+RR	1178
535	*Xanthomonas campestris pv campestris 8004*	**P(γ)**	Q4UW16	YP_242777.1/NP_637775.1	540	LOV+Kinase+RR	1179
	Xanthomonas campestris pv campestris ATCC 33913	**P(γ)**	Q8P827	NP_637775.1	540	LOV+Kinase+RR	1180
	Xanthomonas campestris pv. campestris B100	**P(γ)**	B0RRL1	YP_001903150.1	540	LOV+Kinase+RR	1181
536	*Xanthomonas campestris pv. raphani 756C*	**P(γ)**	G0CI52	YP_005637715.1	540	LOV+Kinase+RR	1182
537	*Xanthomonas campestris pv. vesicatoria 85-10*	**P(γ)**	Q3BRX8	YP_364485.1	540	LOV+Kinase+RR	1183
538	*Xanthomonas citri pv. mangiferaeindicae LMG 941*	**P(γ)**	H8FDX1	WP_003484907.1	502	LOV+Kinase+RR	1184
539	*Xanthomonas citri subsp. citri Aw12879*	**P(γ)**	M4VYP7	YP_007650222.1	540	LOV+Kinase+RR	1185
540	*Xanthomonas fuscans subsp. aurantifolii str. ICPB 10535*	**P(γ)**	D4TB06	WP_007965960.1	540	LOV+Kinase+RR	1186
	Xanthomonas fuscans subsp. aurantifolii str. ICPB 11122	**P(γ)**	D4SVY4	WP_007965960.1	540	LOV+Kinase+RR	1187
541	*Xanthomonas gardneri ATCC 19865*	**P(γ)**	F0C005	WP_006448485.1	180	Short-LOV	1188
542	*Xanthomonas oryzae*	**P(γ)**		WP_019299959.1	507	LOV+Kinase+RR	1189
543	*Xanthomonas oryzae pv. oryzae MAFF 311018*	**P(γ)**	Q2P13	YP_452017.1	540	LOV+Kinase+RR	1190
	Xanthomonas oryzae pv. oryzae (strain KACC10331 / KXO85)	**P(γ)**	Q5GY20	YP_201786.1	428	LOV+Kinase	1191
	Xanthomonas oryzae pv. oryzae PXO99A	**P(γ)**	B2SUI2	YP_001914487.1	507	LOV+Kinase+RR	1192
544	*Xanthomonas oryzae pv. oryzicola BLS256*	**P(γ)**	G7TAT9	YP_005628219.1	502	LOV+Kinase+RR	1193
545	*Xanthomonas perforans 91-118*	**P(γ)**	F0BMJ4	WP_008571382.1	540	LOV+Kinase+RR	1194
546	*Xanthomonas sacchari*	**P(γ)**		WP_010342969.1	544	LOV+Kinase+RR	1195
547	*Xanthomonas sp. SHU199*	**P(γ)**		WP_017907883.1	544	LOV+Kinase+RR	1196
548	*Xanthomonas sp. SHU308*	**P(γ)**		WP_017914963.1	544	LOV+Kinase+RR	1197
549	*Xanthomonas translucens DAR61454*	**P(γ)**	L7FSH1	WP_003473017.1	542	LOV+Kinase+RR	1198
550	*Xanthomonas translucens pv. graminis ART-Xtg29*	**P(γ)**	K8ZG10	WP_009602159.1	542	LOV+Kinase+RR	1199
551	*Xanthomonas translucens pv. translucens DSM 18974*	**P(γ)**	L0SUV4	WP_003474558.1	542	LOV+Kinase+RR	1200
552	*Xanthomonas vasicola*	**P(γ)**		WP_010363479.1	541	LOV+Kinase+RR	1201
				WP_017116448.1	508	LOV+Kinase+RR	1202
553	*Xanthomonas vesicatoria ATCC 35937*	**P(γ)**	F0BB91	WP_005990538.1	519	LOV+Kinase+RR	1203
554	*Chloroflexus aggregans DSM 9485*	**Chl**	B8GAY9	YP_002465025.1	913	LOV+PAS+PAS+Kinase+RR	1204
555	*Chloroflexus aurantiacus ATCC 29364*	**Chl**	B9LBT8	YP_002571059.1/YP_001636696.1	915	LOV +2PAS +Kinase+RR+Hpt	1205
	Chloroflexus aurantiacus J-10-fl	**Chl**	A9WHI1	YP_001636696.1	915	LOV +2PAS+Kinase+RR+Hpt	1206
556	*Chloroherpeton thalassium ATCC35110*	**Chl**	B3QS59	YP_001996451.1	1333	3PAS+LOV+GAF+PAS+Kinase+RR	1207
557	*Herpetosiphon aurantiacus ATCC 23779*	**Chl**	A9B1I0	YP_001543993.1	1877	HAMP+GAF+LOV+4PAS+GAF+Kinase	1208
558	*Acaryochloris marina MBIC 11017*	**Cya**	B0C6M6	YP_001515761.1	935	PAS+LOV+2PAS+GGDEF+EAL	1209
559	*Acaryochloris sp. CCMEE 5410*	**Cya**		WP_010476320.1	912	2PAS+LOV+PAS+GGDEF+EAL	1210
560	*Anabaena variabilis ATCC 29413*	**Cya**	Q3M6B3	YP_324368.1	1021	LOV+2PAS+GGDEF+EAL	1211
			Q3MED3	YP_321548.1	1820	PAS+LOV+3PAS+GAF+Kinase+ 2RR+Hpt	1212
561	*Arthrospira maxima CS-328*	**Cya**	B5W5T2	WP_006625197.1	1276	PAS+GAF+PAS+GAF+LOV+GGDEF+EAL	1213
			B5VY09	WP_006668677.1	1184	GAF+PAS+LOV+PAS+GAF+Kinase	1214
562	*Arthrospira platensis C1*	**Cya**	K1W4H5	WP_006625197.1	1276	PAS+GAF+LOV+GGDEF+EAL	1215

			K1X4B3	WP_006624369.1	1240	GAF+PAS+LOV+PAS+Kinase	1216
			K1W3R6	WP_006625388.1	452	RR+LOV+GGDEF	1217
563	*Arthrospira platensis NIES-39*	**Cya**	D5A2C5	YP_005067517.1	501	RR+LOV+GGDEF	1218
			D5A3S6	YP_005067758.1	1276	PAS+GAF+LOV+GAF+GGDEF+EAL	1219
			D5A1P6	YP_005071467.1	1240	GAF+2PAS+LOV+GAF+Kinase	1220
564	*Arthrospira platensis str. Paraca*	**Cya**	K6DZN7	WP_006618143.1	502	RR+LOV+GGDEF	1221
			K6DYP8	WP_006618516.1	1276	PAS+GAF+PAS+GAF+LOV+GGDEF+EAL	1222
			K6DR83	WP_006616930.1	1240	GAF+PAS+LOV+PAS+Kinase	1223
565	*Arthrospira sp. PCC 8005*	**Cya**	H1WBC0	WP_008051195.1	542	RR+LOV+GGDEF	1224
			H1WBU5	WP_008051404.1	1279	PAS+GAF+LOV+GAF+PAS+EAL	1225
			H1WDU3	WP_008052387.1	1240	GAF+2LOV+PAS+2GAF+Kinase	1226
566	*Chlorogloeopsis*	**Cya**		WP_016873643.1	917	PAS+LOV+PAS+GGDEF+EAL	1227
				WP_016876159.1	1301	PAS+LOV+3PAS+Kinase	1228
567	*Crinalium epipsammum PCC 9333*	**Cya**	K9VUA3	YP_007140547.1	973	LOV+GAF+Kinase	1229
			K9VVG0	YP_007141588.1	811	LOV+PAS+GAF+Kinase	1230
568	*Crocosphaera watsonii*	**Cya**		WP_021836924.1	274	RR+LOV	1231
				WP_021831117.1	222	RR+LOV	1232
	Crocosphaera watsonii WH 8501	**Cya**	Q4BW45	WP_007308072.1	297	RR+LOV	1233
	Crocosphaera watsonii WH 0003	**Cya**	G5JC80	WP_007312830.1	483	RR+LOV+GGDEF	1234
569	*Cyanobacterium aponinum PCC 10605*	**Cya**	K9Z5T6	YP_007162595.1	1220	LOV+GAF+2PAS+GGDEF+EAL	1235
570	*Cyanothece ATCC 51142*	**Cya**	B1WPX1	YP_001805352.1	483	RR+LOV+GGDEF	1236
	Cyanothece PCC 8801/ Synechococcus sp. PCC 8801	**Cya**	B7JZ07	YP_002372240.1	481	RR+LOV+GGDEF	1237
	Cyanothece sp. CCY0110	**Cya**	A3IMW4	WP_008274722.1	483	RR+LOV+GGDEF	1238
	Cyanothece sp. PCC 8802	**Cya**	C7QX31	YP_003137799.1	481	RR+LOV+GGDEF	1239
	Cyanothece sp.ATCC51472	**Cya**	G6GZ93	EHC21269.1/YP_001805352.1	483	RR+LOV+GGDEF	1240
571	*Cylindrospermum stagnale PCC 7417*	**Cya**	K9WTZ1	YP_007146338.1	2260	cbs+3PAS+GAF+LOV+Kinase	1241
572	*filamentous cyanobacterium ESFC-1*	**Cya**		WP_018397868.1	1587	GAF+LOV+5PAS+GGDEF+EAL	1242
				WP_018398448.1	1504	cbs+GAF+3PAS+LOV+3PAS+Kinase	1243
				WP_018398453.1	1047	GAF+3PAS+LOV+PAS+Kinase	1244
573	*Fischerella muscicola*	**Cya**		WP_016869711.1	1013	CBS+3PAS+LOV+PAS+Kinase	1245
				WP_016859885.1	803	CBS+LOV+PAS+Kinase	1246
574	*Geitlerinema sp. PCC 7105*	**Cya**		WP_017659770.1	1803	2PAS+GAF+6PAS+LOV+2PAS+Kinase	1247
575	***Geitlerinema sp. PCC 7407***	**Cya**	K9S9T8	YP_007110030.1	1135	2PAS+GAF+LOV+PAS+GGDEF+EAL	1248
576	*Geminocystis herdmanii*	**Cya**		WP_017295851.1	1249	LOV+GAF+2PAS+GGDEF+EAL	1249
577	*Gloeocapsa sp. PCC 73106*	**Cya**	L8LH99	WP_006530862.1	1137	GAF+LOV+2PAS+GGDEF+EAL	1250
578	*Halothece sp. PCC 7418 (Synechococcus sp. (strain PCC 7418))*	**Cya**	K9YDF6	YP_007168649.1	731	LOV+PAS+GGDEF+EAL	1251
579	*Leptolyngbya sp. PCC 7376*	**Cya**	K9Q326	YP_007072303.1	1052	GAF+LOV+PAS+GGDEF+EAL	1252
580	*Lyngbya sp. PCC 8106*	**Cya**	A0YVI7	WP_009786481.1	477	RR+LOV+GGDEF	1253
			A0YYE1	WP_009787479.1	1090	PAS+LOV+PAS+GGDEF+EAL	1254
			A0YS28	WP_009785277.1	1261	RR+RR+LOV+PAS+GAF+Kinase+RR	1255
			A0YI13	WP_009782119.1	1006	PAS+LOV+3PAS+GAF+GGDEF	1256
			A0YJV6	WP_009782762.1	1211	GAF+PAS+LOV+PAS+GAF+Kinase	1257
			A0YYE8	*WP_009787486.1*	990	PAS+GAF+LOV+GGDEF+EAL	1258
581	*Mastigocladopsis repens*	**Cya**		WP_017316340.1	897	MHYT+GAF+LOV+MCP	1259
				WP_017315856.1	1497	CBS+LOV+4PAS+Kinase	1260
				WP_017315856.1	1497	CBS+LOV+4PAS+Kinase	1261
582	*Microchaete sp. PCC 7126*	**Cya**		WP_017651577.1	1069	cbs+PAS+GAF+2PAS+LOV+GAF+Kinase	1262
583	*Microcoleus cHTHonoplastes PCC 7420*	**Cya**	B4VK29	WP_006098908.1	2020	2PAS+LOV+4PAS+2GAF+Kinase+ 2RR	1263
			B4VKN6	WP_006099135.1	483	PAS+LOV+Cyclase	1264
			B4VSY1	WP_006101616.1	1145	RR+GAF+LOV+2PAS+GAF+Kinase	1265
			B4VJB8	WP_006098397.1	776	LOV+GAF+Kinase	1266
			B4VY11	WP_006103549.1	1393	2PAS+GAF+LOV+2PAS+GGDEF+EAL	1267
584	*Microcoleus sp. PCC 7113*	**Cya**	K9WJW9	YP_007124111.1	880	LOV+PAS+Kinase+RR	1268

			K9WIF3	YP_007123389.1	1204	RR+3PAS+LOV+GAF+Kinase	1269
			K9WFV8	YP_007122711.1	483	LOV+PAS+GGDEF	1270
			K9W9I5	YP_007120451.1	494	RR+LOV+GGDEF	1271
			K9WEP7	YP_007122079.1	929	PAS+LOV+PAS+GAF+Kinase	1272
			K9WK80	YP_007123343.1	621	RR+LOV+Kinase	1273
585	*Microcoleus vaginatus FGP-2*	Cya	F5UCI6	WP_006631032.1	1102	LOV+4PAS+Kinase	1274
			F5UNN6	WP_006635552.1	1113	LOV+4PAS+GAF+Kinase	1275
			F5UBU5	WP_006631635.1	1024	LOV+4PAS+GGDEF+EAL	1276
			F5UC68	WP_006631762.1	1375	PAS+GAF+LOV+GAF+PAS+2EAL	1277
586	*Nodosilinea nodulosa*	Cya		WP_017296958.1	834	GAF+LOV+Kinase+RR+Hpt	1278
587	*Nostoc punctiforme PCC 73102*	Cya	B2JAQ5	YP_001870050.1	1043	LOV+2PAS+GGDEF+EAL	1279
			B2J8F4	YP_001865874.1	1403	PAS+LOV+PAS+GAF+Kinase+2RR+Hpt	1280
588	*Nostoc sp. ATCC 29411*	Cya	K9QZN6	YP_007078500.1	1019	LOV+2PAS+GGDEF+EAL	1281
589	*Nostoc sp. ATCC 29412*	Cya	K9QT62	YP_007076412.1	1712	CHASE+PAS+LOV+PAS+GAF+Kinase+RR+HPT	1282
590	*Nostoc sp. PCC 7107*	Cya	K9QBD4	YP_007049843.1	1567	CHASE+PAS+LOV+PAS+GAF+Kinase+RR+HPT	1283
591	*Nostoc sp. PCC 7120*	Cya	Q8YSB9	NP_487210.1	1021	LOV+2PAS+GGDEF+EAL	1284
			Q8YT51	NP_486915.1	1817	PAS+LOV+3PAS+GAF+Kinase+ 2RR+Hpt	1285
592	*Oscillatoria acuminata PCC 6304*	Cya	K9TPZ8	YP_007088398.1	1288	PAS+GAF+2PAS+LOV+GGDEF+EAL	1286
593	*Oscillatoria formosa*	Cya		WP_019486854.1	1518	2PAS+GAF+PAS+GAF+LOV+GGDEF+EAL	1287
594	*Oscillatoria nigro-viridis PCC 7112*	Cya	K9VLB1	YP_007117299.1	1344	PAS+GAF+LOV+PAS+GGDEF+EAL	1288
			K9VF92	YP_007114341.1	1003	LOV+3PAS+GGDEF+EAL	1289
			K9VJW6	YP_007115936.1	1113	LOV+4PAS+GAF+Kinase	1290
			K9VDJ7	YP_007114659.1	1102	LOV+2PAS+Kinase	1291
595	*Oscillatoria sp. PCC 10802*	Cya		WP_017717108.1	1992	RR+2PAS+LOV+6PAS+GAF+Kinase+RR	1292
596	*Oscillatoria sp.PCC6506*	Cya	D8FYX	WP_007354786.1	631	PAS+LOV+Kinase	1293
			D8G8N3	WP_007358144.1	1041	LOV+GAF+PAS+GGDEF+2EAL	1294
			D8FU68	WP_007353135.1	1432	GAF+LOV+PAS+LOV+PAS+2GAF+Kinase	1295
			D8G759	WP_007357627.1	1140	LOV+3PAS+GGDEF+2EAL	1296
597	*Pleurocapsa sp. PCC 7327*	Cya	K9T9F1	YP_007082610.1	479	RR+LOV+GGDEF	1297
598	*Pseudanabaena biceps PCC 7429*	Cya	L8MY36	WP_009626941.1	1179	PAS+LOV+GAF+Kinase+RR+HpT	1298
			L8MXD6	WP_009628448.1	945	PAS+LOV+PAS+GAF+Kinase	1299
599	*Pseudanabaena sp. PCC 6802*	Cya		WP_019500070.1	935	LOV+2PAS+Kinase	1300
				WP_019498621.1	737	RR+PAS+LOV+Kinase	1301
600	*Scytonema hofmanni*	Cya		WP_017745401.1	930	MHYT+GAF+LOV+HAMP+MCP	1302
				WP_017746529.1	883	GAF+LOV+PAS+GGDEF+EAL	1303
				WP_017740454.1	949	CBS+LOV+PAS+Kinase	1304
				WP_017744138.1	931	MHYT+GAF+LOV+HAMP+MCP	1305
				WP_017740600.1	923	2RR+LOV+Kinase+RR	1306
601	*Spirulina subsalsa*	Cya		WP_017306948.1	1652	RR+LOV+8PAS+Kinase	1307
				WP_017305004.1	451	PAS+LOV+GGDEF	1308
602	*Synechococcus sp. ATCC 27167*	Cya	K9RZ52	YP_007063296.1	1154	GAF+PAS+LOV+Kinase+RR	1309
603	*Synechococcus sp. PCC 6301*	Cya	Q5N2F7	YP_172033.1	578	LOV+GGDEF+EAL	1310
			Q5N5M8	YP_170910.1	929	RR+PAS+PAS+LOV+ GGDEF+EAL	1311
604	*Synechococcus sp. PCC 7002*	Cya	B1XLQ8	YP_001734607.1	780	GAF+LOV+PAS+GGDEF	1312
605	*Synechococcus sp. PCC 7502*	Cya	K9SZJ8	YP_007107456.1	914	2PAS+LOV+GAF+Kinase	1313
606	*Synechococcus sp. PCC 7942*	Cya	Q31RU9	YP_399207.1	578	LOV+GGDEF+EAL	1314
			Q31NI4	YP_400372.1	929	RR+PAS+PAS+LOV+ GGDEF+EAL	1315
607	*Synechocystis sp. PCC 6803*	Cya	L8ARN4	BAM54579.1	1235	GAF+LOV+3PAS+GGDEF+EAL	1316
			Q55576	NP_442010.1	1244	GAF+LOV+3PAS+GGDEF+EAL	1317
			F7URV3	YP_005652070.1/NP_442010.1	1244	GAF+LOV+3PAS+GGDEF+EAL	1318
608	*Synechocystis sp. PCC 6803 substr. PCC-P*	Cya	H0PFN3	YP_005387047.1/NP_442010.1	1244	GAF+LOV+3PAS+GGDEF+EAL	1319
			H0PAQ0	YP_005409754.1/NP_442010.1	1244	GAF+LOV+3PAS+GGDEF+EAL	1320
609	*Synechocystis sp.PCC 6803 substr. GT-I*	Cya	H0NY98	YP_005383878.1/NP_442010.1	1244	GAF+LOV+3PAS+GGDEF+EAL	1321

610	*Thermosynechococcus elongatus BP-1*	**Cya**	Q8DJE3	NP_682072.1	1353	GAF+PAS+LOV+2GAF +Kinase+ RR	1322
611	*Gemmata obscuriglobus*	**Pl**		WP_010043508.1	823	PAS+LOV+Kinase+RR	1323
				WP_010043417.1	997	RR+2PAS+LOV+PAS+Kinase	1324
				WP_010041727.1	1032	2PAS+LOV+PAS+Kinase+RR	1325
				WP_010042468.1	1178	2PAS+LOV+PAS+GAF+Kinase+RR	1326
612	*planctomycete KSU-1*	**Pl**	I3IH21	WP_007220142.1	1155	GAF+LOV+2PAS+Kinase+RR	1327
613	*Rhodopirellula baltica SH1*	**Pl**	Q7USG5	NP_866145.1	1637	CHEB+CHER+PAS+LOV+PAS+Kinase+ RR	1328
	Rhodopirellula baltica SH28	**Pl**	K5DM72	WP_007330973.1	1637	CHER+2PAS+LOV+PAS+Kinase+RR	1329
	Rhodopirellula baltica SWK14	**Pl**	L7CQZ7	WP_007335941.1	1637	CHER+2PAS+LOV+PAS+Kinase+RR	1330
	Rhodopirellula baltica SWK14	**Pl**	L7C999	WP_007340810.1	1739	CHER+2PAS+LOV+PAS+Kinase+RR	1331
	Rhodopirellula baltica WH47	**Pl**	F2B0L3	WP_007329423.1	1637	RR+CHER-CHERB+3PAS+LOV+Kinase	1332
614	*Rhodopirellula europaea 6C*	**Pl**	M2B329	WP_008657036.1	651	LOV+Kinase	1333
			M2AB22	WP_008661450.1	1637	CHER+2PAS+LOV+PAS+Kinase+RR	1334
			M2B160	WP_008658004.1	1739	CHER+2PAS+LOV+PAS+Kinase+RR	1335
	Rhodopirellula europaea SH398	**Pl**	M5S556	WP_008666956.1	1637	CHER+2PAS+LOV+PAS+Kinase+RR	1336
615	*Rhodopirellula maiorica SM1*	**Pl**	M5RDE8	WP_008703751.1	1471	CHER+2PAS+LOV+PAS+Kinase+RR	1337
616	*Rhodopirellula sallentina SM41*	**Pl**	M5U3R2	WP_008678750.1	1752	CHER+2PAS+LOV+PAS+Kinase+RR	1338
617	*Rhodopirellula sp. SWK7*	**Pl**	M5TB77	WP_009094396.1	1738	CHER+2PAS+LOV+PAS+Kinase+RR	1339
618	*Schlesneria paludicola*	**Pl**		WP_010583390.1	819	PAS+GAF+LOV+Kinase+RR	1340
				WP_010587131.1	925	PAS+LOV+Kinase+RR+HPT	1341
619	*Zavarzinella formosa*	**Pl**		WP_020473798.1	660	LOV+Kinase+RR	1342
620	*Leptospirillum ferriphilum (strain ML-04)*	**Nit**	J9Z9K3	YP_006766144.1	1036	GAF+LOV+PAS+GGDEF+EAL	1343
621	*Leptospirillum ferrodiazotrophum*	**Nit**	C6HUQ3	EES53679.1	1354	2PAS+GAF+LOV+GGDEF+Globin	1344
			C6HW67	EES53172.1	1369	PAS+2GAF+PAS+LOV+GGDEF+Globin	1345
			C6HYK0	EES52285.1	496	PAS+LOV+Kinase	1346
622	*Leptospirillum ferrooxidans (strain C2-3)*	**Nit**	I0IRY1	YP_005470160.1	489	LOV+GGDEF	1347
623	*Leptospirillum sp. Group II 'C75'*	**Nit**		EIJ77392.1	1036	GAF+PAS+LOV+GGDEF+EAL	1348
624	*Leptospirillum sp. Group IV 'UBA BS'*	**Nit**	T0Z8K9	WP_021811090.1	1614	PAS+GAF+PAS+GAF+LOV+GGDEF+EAL	1349
625	*Leptospirillum sp. GrouPII '5-way CG'*	**Nit**	B6AQW0	EDZ38519.1	1035	GAF+LOV+PAS+GGDEF+EAL	1350
626	*Leptospirillum sp. GrouPII UBA*	**Nit**	A3ERH5	EAY57303.1	1036	GAF+LOV+PAS+GGDEF+EAL	1351
627	*Acidobacterium sp. (strain MP5ACTX9)*	**Bact**	E8X7C4	YP_004210485.1	519	LOV+Kinase	1352
628	*Cellulophaga algicola DSM 14237*	**Bact**	E6XF72	YP_004165806.1	628	PAS+LOV+Kinase	1353
			E6XEC5	YP_004165713.1	1083	3PAS+LOV+GAF+Kinase	1354
629	*Chloroherpeton thalassium ATCC 35110*	**Bact**	B3QWA0	YP_001995660.1	190	Short-LOV	1355
630	*Cyclobacterium qasimii M12-11B*	**Bact**	S7V803	WP_020889873.1	972	2PAS+LOV+GAF+Kinase	1356
631	*Eudoraea adriatica*	**Bact**		WP_019670355.1	631	PAS+LOV+PAS+Kinase	1357
632	*Formosa sp. AK20*	**Bact**	M7MMR6	WP_007647936.1	809	PAS+LOV+PAS+GAF+Kinase	1358
633	*Fulvivirga imtechensis AK7*	**Bact**	L8JI64	WP_009583264.1	1336	CHER+PAS+LOV+PAS+Kinase	1359
634	*Granulicella mallensis DSM 23137*	**Bact**	G8NP03	YP_005058137.1	519	LOV+Kinase	1360
635	*Mucilaginibacter paludis DSM 18603*	**Bact**	H1Y366	WP_008509841.1	508	LOV+PAS+Kinase	1361
636	*Muricauda ruestringensis (strain DSM 13258)*	**Bact**	G2PRN8	YP_004786909.1	848	PAS+LOV+GAF+Kinase	1362
637	*Pedobacter agri*	**Bact**		WP_010603242.1	517	LOV+PAS+Kinase	1363
638	*Pedobacter sp. BAL39*	**Bact**	A6E9H1	WP_008239045.1	512	LOV+PAS+Kinase	1364
639	*Polaribacter franzmannii*	**Bact**		WP_018944019.1	836	PAS+LOV+GAF+Kinase	1365
640	*Sphingobacterium sp. (strain 21)*	**Bact**	F4CCK0	YP_004320289.1	520	LOV+PAS+Kinase	1366
641	*Sphingobacterium sp. IITKGP-BTPF85*	**Bact**	T0MYW2	WP_021188605.1	282	LOV+PAS	1367
642	*Zobellia galactanivorans strain DSM 12802*	**Bact**	G0L288	YP_004738261.1	1092	3PAS+LOV+GAF+PAS+Kinase	1368
643	*Deinococcus apachensis*	**DeTh**		WP_019586595.1	285	LOV+GGDEF	1369
644	*Deinococcus peraridilitoris strain DSM 19664*	**DeTh**	L0A6T3	YP_007174032.1	297	LOV+GGDEF	1370
645	*Meiothermus ruber ATCC 35948*	**DeTh**	D3PRD8	YP_003507041.1	578	LOV+2PAS+Kinase	1371
646	*Truepera radiovictrix DSM 17093*	**DeTh**	D7CV48	YP_003706418.1	465	LOV+PAS+Kinase	1372
647	*Sulfuricurvum kujiense DSM 16994*	**P(ε)**	E4U216	YP_004060773.1	730	LOV+PDEase/HD	1373
			E4U0K3	YP_004060520.1	568	PAS+LOV+GGDEF	1374

			E4U0H9	YP_004060496.1	239	RR+LOV	1375
648	*Sulfurovum sp. (strain NBC37-1)*	**P(ε)**	A6Q686	YP_001357352.1	169	Short-LOV	1376
649	*Acidobacterium sp. (strain MP5ACTX9)*	**Acido**	E8X1X9	YP_004217920.1	507	LOV+Kinase	1377
650	*Terriglobus roseus strain DSM 18391*	**Acido**	I3ZG30	YP_006421533.1	525	PAS+LOV+GGDEF	1378
			I3ZDW9	YP_006420772.1	522	LOV+Kinase	1379
			I3ZJG2	YP_006422715.1	862	2PAS+LOV+GGDEF+EAL	1380
651	*Turneriella parva ATCC BAA-1111*	**Spi**	I4BB34	YP_006441997.1	876	RR+2PAS+LOV+PAS+Kinase	1381
652	*bacterium enrichment culture clone pWThLOV*	**Unc**	B5M445	ACH61904.1	1204	GAF+2PAS+LOV+2PAS+Kinase+RR	1382
653	*uncultured bacterium*	**Unc**	K2EZH7	EKE24251.1	859	GAF+PAS+LOV+GGDEF+EAL	1383
			K2FIR8	EKE10710.1	620	LOV+Kinase+RR	1384
654	*Bacteriovorax sp. Seq25_V*	**P(δ)**	T0RBD1	EQC43725.1	193	Short-LOV	1385
655	*Desulfococcus multivorans DSM 2059*	**P(δ)**	S7VBV4	WP_020875425.1	1264	LOV+GAF+PAS+Kinase+RR	1386
656	*Myxococcus fulvus (strain ATCC BAA-855 / HW-1)*	**P(δ)**	F8C6F8	YP_004665424.1	802	MASE1+LOV+Kinase+RR	1387
657	*Myxococcus sp. (contaminant ex DSM 436)*	**P(δ)**		WP_002637984.1	809	MASE1+LOV+Kinase+RR	1388
658	*Myxococcus xanthus*	**P(δ)**	Q84FB2	AAO22901.1	830	MASE1+LOV+Kinase+RR	1389
	Myxococcus xanthus (strain DK 1622)	**P(δ)**	Q1CXA1	YP_634972.1	842	MASE1+LOV+Kinase+RR	1390
