# Supplementary material for: From Plant Infectivity to Growth Patterns: The Role of Blue-Light Sensing in the Prokaryotic World
Source: Plants (Basel). 2014 Jan 27;3(1):70–94. doi: 10.3390/plants3010070 (PMC4844311; doi:10.3390/plants3010070)
Supplement: Supplementary File 1 [file plants-03-00070-s001.zip › plants-46379-supplementary-final/Supplementary Material - S2 - BLUF bacteria.pdf]

Bacterial BLUF domains

Nr.	Species/strain	Group	Accession nr. Uniprot	Accession nr. NCBI	aa nr.	Domains	
1	*Enterococcus gallinarum EGD-AAK12*	**F**		ERE59647.1	405	BLUF+EAL	1
2	*Actinomycetospora chiangmaiensis*	**Ac**		WP_018332181.1	148	Short-BLUF	2
				WP_018331556.1	175	Short-BLUF	3
				WP_018332897.1	150	Short-BLUF	4
				WP_018331430.1	141	Short-BLUF	5
3	*Actinoplanes sp. N902-109*	**Ac**	R4LLI6	YP_007952341.1	135	Short-BLUF	6
4	*Clavibacter michiganensis subsp. michiganensis NCPPB 382*	**Ac**	A5CNT4	YP_001221435.1	142	Short-BLUF	7
			A5CM94	YP_001220898.1	148	Short-BLUF	8
5	*Clavibacter michiganensis subsp. nebraskensis NCPPB 2581*	**Ac**	M5B6Z4	YP_007684954.1	142	Short-BLUF	9
			M5B789	YP_007684430.1	139	Short-BLUF	10
6	*Clavibacter michiganensis subsp. sepedonicus ATCC 33113*	**Ac**	B0RBP8		126	Short-BLUF	11
			B0RFL5	YP_001710824.1	142	Short-BLUF	12
7	*Corynebacterium efficiens DSM 44549*	**Ac**	Q8FMY2	NP_738977.1	158	Short-BLUF	13
8	*Corynebacterium maris DSM 45190*	**Ac**	S5TI47	YP_008371038.1	159	Short-BLUF	14
9	*Curtobacterium flaccumfaciens*	**Ac**		WP_017888306.1	142	Short-BLUF	15
				WP_017888712.1	190	Short-BLUF	16
10	*Curtobacterium sp. B18*	**Ac**		WP_022907696.1	142	Short-BLUF	17
				WP_022907690.1	156	Short-BLUF	18
	Curtobacterium sp. B8	**Ac**		WP_022902403.1	109	Short-BLUF	19
				WP_022904642.1	168	Short-BLUF	20
				WP_022902388.1	149	Short-BLUF	21
				WP_022902223.1	139	Short-BLUF	22
11	*Dietzia alimentaria*	**Ac**		WP_010540020.1	158	Short-BLUF	23
12	*Dietzia sp. UCD-THP*	**Ac**		WP_017837588.1	160	Short-BLUF	24
				WP_017836468.1	158	Short-BLUF	25
13	*Ilumatobacter coccineus YM16-304*	**Ac**	M4ZW07	YP_007562816.1	353	BLUF+GGDEF	26
14	*Leifsonia xyli subsp. cynodontis DSM 46306*	**Ac**		YP_008579210.1	168	Short-BLUF	27
15	*Leifsonia xyli subsp. xyli*	**Ac**	Q6AGS2	YP_061528.1	145	Short-BLUF	28
16	*Leucobacter chromiiresistens*	**Ac**		WP_010155083.1	154	Short-BLUF	29
17	*Microbacterium maritypicum MF109*	**Ac**	T5KEX1	WP_021201284.1	162	Short-BLUF	30
18	*Microbacterium paraoxydans*	**Ac**		WP_018186897.1	149	Short-BLUF	31
				WP_018187668.1	142	Short-BLUF	32
19	*Microbacterium sp. 11MF*	**Ac**		WP_020098083.1	149	Short-BLUF	33
				WP_020098408.1	142	Short-BLUF	34
	Microbacterium sp. B19	**Ac**		WP_022879455.1	146	Short-BLUF	35
	Microbacterium sp. TS-1	**Ac**		GAD34866.1	156	Short-BLUF	36
	Microbacterium sp. UCD-TDU	**Ac**		WP_017829376.1	174	Short-BLUF	37
				WP_017829365.1	193	Short-BLUF	38
20	*Microbacterium testaceum (strain StLB037)*	**Ac**	E8NB71	YP_004224598.1	145	Short-BLUF	39
21	*Afipia sp. 1NLS2*	**P(α)**	D6V881	WP_009340159.1	151	Short-BLUF	40
22	*Ahrensia sp. R2A130*	**P(α)**	E0MSP0	WP_009758676.1	125	Short-BLUF	41
23	*alpha proteobacterium BAL199*	**P(α)U**	A8TQR5	WP_007672921.1	124	Short-BLUF	42
24	*alpha proteobacterium JLT2015*	**P(α)U**	M2T6L9	WP_008603323.1	133	Short-BLUF	43
25	*alpha proteobacterium L41A*	**P(α)U**		WP_017506466.1	135	Short-BLUF	44
26	*alpha proteobacterium SCGC AAA076-C03*	**P(α)U**		WP_020057179.1	142	Short-BLUF	45
27	*alpha proteobacterium SCGC AAA298-K06*	**P(α)U**		WP_020044700.1	140	Short-BLUF	46
28	*Amorphus coralli*	**P(α)**		WP_018701439.1	148	Short-BLUF	47
29	*Asticcacaulis benevestitus*	**P(α)**		WP_018084049.1	161	Short-BLUF	48
				WP_023447472.1	163	Short-BLUF	49

30	*Asticcacaulis biprosthecum C19*	P(α)	F4QHK8	WP_006271926.1	122	Short-BLUF	50
31	*Asticcacaulis sp. AC460*	P(α)		WP_023451483.1	140	Short-BLUF	51
	Asticcacaulis sp. AC466	P(α)		WP_023459633.1	139	Short-BLUF	52
				WP_023456967.1	169	Short-BLUF	53
	Asticcacaulis sp. YBE204	P(α)		WP_023462718.1	148	Short-BLUF	54
32	*Aureimonas ureilytica*	P(α)		WP_019995874.1	108	Short-BLUF	55
				WP_019998548.1	138	Short-BLUF	56
33	*Azospirillum lipoferum (strain 4B)*	P(α)	G7ZDG1	YP_004974645.1	163	Short-BLUF	57
			G7Z5P1	YP_005039978.1	161	Short-BLUF	58
34	*Blastomonas sp. AAP53*	P(α)		WP_017672203.1	135	Short-BLUF	59
35	*Bradyrhizobium sp. STM 3809*	P(α)	H0SXV9	WP_008961634.1	151	Short-BLUF	60
			H0T8Q2	WP_008965377.1	125	Short-BLUF	61
36	*Brevundimonas abyssalis TAR-001*	P(α)	U2ZDP3	WP_021697307.1	100	Short-BLUF	62
37	*Brevundimonas diminuta 470-4*	P(α)	L1QEI8	WP_003168376.1	133	Short-BLUF	63
			L1QGZ1	WP_003168022.1	165	Short-BLUF	64
38	*Brevundimonas diminuta ATCC 11568*	P(α)	F4QZ49	WP_003166443.1	136	Short-BLUF	65
			F4R104	WP_003166654.1	136	Short-BLUF	66
39	*Brevundimonas sp. BAL3*	P(α)	B4W666	WP_008261602.1	136	Short-BLUF	67
			B4WEH9	WP_008264082.1	151	Short-BLUF	68
			B4W667	WP_008262982.1	141	Short-BLUF	69
40	*Brevundimonas subvibrioides ATCC 15264*	P(α)	D9QFJ9	YP_003820137.1	133	Short-BLUF	70
			D9QF25	YP_003818133.1	148	Short-BLUF	71
			D9QJR6	YP_003817291.1	137	Short-BLUF	72
			D9QHQ8	YP_003816957.1	129	Short-BLUF	73
			D9QN89	YP_003817913.1	167	Short-BLUF	74
41	*Caenispirillum salinarum AK4*	P(α)	K9GNQ3	WP_009542082.1	148	Short-BLUF	75
42	*Caulobacter sp. JGI 0001010-J14*	P(α)		WP_019847616.1	155	Short-BLUF	76
	Caulobacter sp. JGI 0001013-D04	P(α)		WP_018113275.1	155	Short-BLUF	77
43	*Citreicella sp. 357*	P(α)	I1ASF9	WP_009506357.1	135	Short-BLUF	78
	Citreicella sp. SE45	P(α)	D0D5D9	WP_008885899.1	131	Short-BLUF	79
44	*Dinoroseobacter shibae DFL 12*	P(α)	A8LL23	YP_001534373.1	135	Short-BLUF	80
45	*Fodinicurvata sediminis*	P(α)		WP_022728487.1	145	Short-BLUF	81
46	*Fulvimarina pelagi HTCC2506*	P(α)	Q0G5H1	WP_007067067.1	144	Short-BLUF	82
			Q0G0Q7	WP_007068840.1	145	Short-BLUF	83
			Q0G114	WP_007068733.1	122	Short-BLUF	84
47	*Henriciella marina*	P(α)		WP_018146618.1	137	Short-BLUF	85
				WP_018146679.1	136	Short-BLUF	86
				WP_018147000.1	146	Short-BLUF	87
48	*Hoeflea phototrophica DFL-43*	P(α)	A9DDP2	WP_007196455.1	130	Short-BLUF	88
49	*Hyphomonas neptunium ATCC 15444*	P(α)	Q0C3J3	YP_759700.1	152	Short-BLUF	89
50	*Jannaschia sp. (strain CCS1)*	P(α)	Q28NL5	YP_510722.1	164	Short-BLUF	90
			Q28QR5	YP_509972.1	150	Short-BLUF	91
			Q28M24	YP_511263.1	135	Short-BLUF	92
51	*Labrenzia aggregata IAM 12614*	P(α)	A0NTY6	WP_006935087.1	152	Short-BLUF	93
52	*Labrenzia alexandrii DFL-11*	P(α)	B9QVG5	WP_008192786.1	152	Short-BLUF	94
53	*Loktanella vestfoldensis*	P(α)		WP_019955376.1	138	Short-BLUF	95
	Loktanella vestfoldensis SKA53	P(α)	A3V3H6	WP_007204263.1	136	Short-BLUF	96
54	*Magnetospirillum gryphiswaldense*	P(α)	A4U007	CAM76214.1	156	Short-BLUF	97
			A4U445	CAM77652.1	151	Short-BLUF	98
55	*Maricaulis maris (strain MCS10)*	P(α)	Q0AMF1	YP_757480.1	152	Short-BLUF	99
			Q0ALH8	YP_757803.1/NP_949618.1	141	Short-BLUF	100
56	*Maricaulis sp. JL2009*	P(α)		WP_022699434.1	141	Short-BLUF	101
57	*Methylobacterium chloromethanicum CM4*	P(α)	B7KRN3	YP_002423488.1/YP_002422117.1	150	Short-BLUF	102

			B7KN60	YP_002423105.1	148	Short-BLUF	103
			B7L1R5	YP_002419387.1	150	Short-BLUF	104
			C5B322	YP_002965329.1	148	Short-BLUF	105
58	*Methylobacterium extorquens AM1*	P(α)	C5AVI4	YP_002961929.1/YP_001638425.1	155	Short-BLUF	106
			C7CE09	YP_003066731.1/YP_001638425.1	155	Short-BLUF	107
	Methylobacterium extorquens DM4	P(α)	C7CJD7	YP_003070453.1	148	Short-BLUF	108
			H1KED3	WP_003597563.1/YP_002965329.1	148	Short-BLUF	109
	Methylobacterium extorquens DSM 13060	P(α)	H1KPI6	WP_003603713.1/YP_001638425.1	155	Short-BLUF	110
			A9VWG7	YP_001637613.1	150	Short-BLUF	111
	Methylobacterium extorquens PA1	P(α)	A9W8Q2	YP_001641472.1	148	Short-BLUF	112
			A9W198	YP_001638425.1	155	Short-BLUF	113
59	*Methylobacterium mesophilicum SR1.6/6*	P(α)	M7XWX4	WP_010687537.1	294	BLUF	114
			M7XZR2	WP_010683805.1	155	Short-BLUF	115
60	*Methylobacterium nodulans ORS 2060*	P(α)	B8IB70	YP_002497588.1	155	Short-BLUF	116
61	*Methylobacterium populi ATCC BAA-705*	P(α)	B1ZGT3	YP_001927146.1	162	Short-BLUF	117
			B1Z908	YP_001923601.1	155	Short-BLUF	118
62	*Methylobacterium radiotolerans ATCC 27329*	P(α)	B1M482	YP_001757060.1	309	Short-BLUF	119
			B1M108	YP_001755241.1	139	Short-BLUF	120
			B1LTA6	YP_001753095.1	143	Short-BLUF	121
			B1M0G5	YP_001758170.1	155	Short-BLUF	122
63	*Methylobacterium sp. 285MFTsu5.1*	P(α)		WP_020092935.1	155	Short-BLUF	123
	Methylobacterium sp. 4-46	P(α)	B0UJP3	YP_001769419.1	150	Short-BLUF	124
			B0ULI8	YP_001770966.1	155	Short-BLUF	125
			B0U8I2	YP_001768746.1	296	Short-BLUF	126
			B0UQT1	YP_001771351.1	162	Short-BLUF	127
			B0UIK0	YP_001770814.1	373	Short-BLUF	128
	Methylobacterium sp. 77	P(α)		WP_019905219.1	155	Short-BLUF	129
	Methylobacterium sp. 88A	P(α)		WP_018045976.1	150	Short-BLUF	130
				WP_018044868.1	155	Short-BLUF	131
	Methylobacterium sp. GXF4	P(α)	I9CMR0	WP_007562637.1	306	Short-BLUF	132
			I9LE30	WP_007566415.1	155	Short-BLUF	133
	Methylobacterium sp. MB200	P(α)		WP_017484131.1	156	Short-BLUF	134
				WP_017487136.1	155	Short-BLUF	135
	Methylobacterium sp. WSM2598	P(α)		WP_018261940.1	290	Short-BLUF	136
64	*Methylocystis rosea*	P(α)		WP_018408523.1	134	Short-BLUF	137
65	*Methylocystis sp. (strain SC2)*	P(α)	J7QPQ2	YP_006590526.1	134	Short-BLUF	138
66	*Oceanicaulis alexandrii*	P(α)		WP_022700825.1	149	Short-BLUF	139
67	*Oceaniovalibus guishaninsula JLT2003*	P(α)	K2HTA6	WP_007425238.1	138	Short-BLUF	140
68	*Octadecabacter antarcticus 307*	P(α)	M9RHP8	YP_007706545.1	143	Short-BLUF	141
			M9R7E3	YP_007705193.1	170	Short-BLUF	142
			M9RA14	YP_007705656.1	170	Short-BLUF	143
			M9RGN7	YP_007705589.1	158	Short-BLUF	144
69	*Octadecabacter arcticus 238*	P(α)	M9RPU8	YP_007699880.1	146	Short-BLUF	145
70	*Pannonibacter phragmitetus*	P(α)		WP_019963279.1	150	Short-BLUF	146
				WP_019963280.1	152	Short-BLUF	147
71	*Paracoccus sp. N5*	P(α)		WP_017999944.1	138	Short-BLUF	148
72	*Paracoccus zeaxanthinifaciens*	P(α)		WP_022708278.1	141	Short-BLUF	149
73	*Parvibaculum lavamentivorans DS-1*	P(α)	A7HQ46	YP_001411686.1/WP_011995320.1	153	Short-BLUF	150
74	*Phaeospirillum molischianum DSM 120*	P(α)	H8FX83	WP_002730899.1	150	Short-BLUF	151
75	*Phenylobacterium zucineum HLK1*	P(α)	B4RAQ0	YP_002132077.1	166	Short-BLUF	152
76	*Polymorphum gilvum LMG 25793*	**P(α)poly**	F2J5Y4	YP_004304542.1	156	Short-BLUF	153
77	*Porphyrobacter sp. AAP82*	P(α)		WP_017663773.1	152	Short-BLUF	154
				WP_017663774.1	130	Short-BLUF	155

78	*Pseudovibrio sp. JE062*	P(α)	B6R848	WP_008551887.1	152	Short-BLUF	156
79	*Puniceispirillum marinum (strain IMCC1322)*	P(α)	D5BS69	YP_003551200.1	411	RRF-BLUF	157
80	*Rhodobacter sp. AKP1*	P(α)	L1KCR3	WP_009563298.1	140	Short-BLUF	158
			L1K965	WP_009564586.1	450	BLUF+SCHICH	159
81	*Rhodobacter sphaeroides (Rhodopseudomonas sphaeroides)*	P(α)	Q53119	YP_351608.1	450	BLUF+SCHICH	160
	Rhodobacter sphaeroides ATCC 17023/2.4.1	P(α)	Q3J677	YP_351608.1	450	BLUF+SCHICH¶	161
			Q3IV98	YP_345277.1	134	Short-BLUF	162
			Q3IYE4	YP_354341.1/WP_011338827.1	140	Short-BLUF	163
	Rhodobacter sphaeroides ATCC 17025	P(α)	A4WX15	YP_001169234.1	448	BLUF+SCHICH	164
			A4WZZ2	YP_001170261.1	135	Short-BLUF	165
	Rhodobacter sphaeroides ATCC 17029	P(α)	A3PG69	YP_001042107.1	450	BLUF+SCHICH	166
			A3PNV7	YP_001044795.1	140	Short-BLUF	167
	Rhodobacter sphaeroides KD131	P(α)	B9KRZ4	YP_002527323.1	450	BLUF+SCHICH	168
			B9KPY5	YP_002527021.1	128	Short-BLUF	169
	Rhodobacter sphaeroides WS8N	P(α)	F3U4S4	WP_002724946.1	174	BLUF+TETR	170
			F5M0G5	EGJ22762.1/YPP_001044795.1	140	Short-BLUF	171
			F5M357	WP_002722303.1	450	BLUF+SCHICH	172
82	*Rhodobacterales bacterium HTCC2255*	P(α)	Q0FFK4	WP_008034321.1	142	Short-BLUF	173
	Rhodobacterales bacterium Y4I	P(α)	B6BAZ4	WP_008557343.1	145	Short-BLUF	174
83	*Rhodopseudomonas palustris (strain HaA2)*	P(α)	Q2J2T2	YP_484139.1	141	Short-BLUF	175
	Rhodopseudomonas palustris BisA53	P(α)	Q07KQ3	YP_782461.1	202	Short-BLUF	176
			Q07NU1	YP_781373.1	172	Short-BLUF	177
			Q07V95	YP_779119.1	146	Short-BLUF	178
			Q07LL8	YP_782146.1	134	Short-BLUF	179
	Rhodopseudomonas palustris BisB18	P(α)	Q211J0	YP_533265.1	225	Short-BLUF	180
			Q21C17	YP_530388.1	147	Short-BLUF	181
			Q214S4	YP_532431.1	131	Short-BLUF	182
	Rhodopseudomonas palustris CGA009	P(α)	Q6NCF0	NP_945875.1	147	Short-BLUF	183
			Q6N1X0	NP_949618.1	157	Short-BLUF	184
			Q6N4H0	NP_948706.1	156	Short-BLUF	185
	Rhodopseudomonas palustris DX-1	P(α)	E6VP72	YP_004108354.1	148	Short-BLUF	186
			E6VKR4	YP_004106926.1	147	Short-BLUF	187
			E6VPR9	YP_004110781.1	157	Short-BLUF	188
	Rhodopseudomonas palustris TIE-1	P(α)	B3QE01	YP_001992821.1	148	Short-BLUF	189
			B3QBC9	YP_001989558.1	147	Short-BLUF	190
			B3Q6V3	YP_001993727.1	157	Short-BLUF	191
84	*Rhodopseudomonas sp. B29*	P(α)		WP_022723876.1	156	Short-BLUF	192
85	*Rhodospirillum centenum ATCC 51521 / SW*	P(α)	B6IP78	YP_002298393.1	148	Short-BLUF	193
86	*Rhodospirillum photometricum DSM 122*	P(α)	H6SMN4	YP_005418139.1	175	Short-BLUF	194
87	*Rhodovulum sp. PH10*	P(α)	J6UCI9	WP_008388881.1	164	Short-BLUF	195
			J6UFI8	WP_008385672.1	144	Short-BLUF	196
88	*Robiginitomaculum antarcticum*	P(α)		WP_017931728.1	138	Short-BLUF	197
89	*Roseibium sp. TrichSKD4*	P(α)	E2CEP5	WP_009466697.1	157	Short-BLUF	198
90	*Roseobacter denitrificans OCh 114*	P(α)	Q169R5	YP_681964.1	131	Short-BLUF	199
91	*Roseobacter litoralis OCh 149*	P(α)	F7ZIV2	YP_004691975.1	131	Short-BLUF	200
92	*Roseobacter sp. AzwK-3b*	P(α)	A6FLZ7	WP_007812297.1	138	Short-BLUF	201
	Roseobacter sp. CCS2	P(α)	A4EDL7	WP_008230522.1	158	Short-BLUF	202
			A4EKD4	WP_008234592.1	95	Short-BLUF	203
	Roseobacter sp. GAI101	P(α)	B7RMG6	WP_008228573.1	108	Short-BLUF	204
	Roseobacter sp. MED193	P(α)	A3X3E5	WP_009811077.1	146	Short-BLUF	205
	Roseobacter sp. SK209-2-6	P(α)	A4EP54	WP_008203735.1	143	Short-BLUF	206
93	*Roseovarius sp. 217*	P(α)	A3W442	WP_009816911.1	137	Short-BLUF	207
	Roseovarius sp. HTCC2601	P(α)	Q0FHM9	WP_007792538.1	131	Short-BLUF	208

	Roseovarius sp. TM1035	P(α)	A6E0Y7	WP_008281285.1	137	Short-BLUF	209
94	*Rubellimicrobium thermophilum DSM 16684*	P(α)	S9QTZ7	WP_021097950.1	142	Short-BLUF	210
95	*Sagittula stellata E-37*	P(α)	A3JXI1	WP_005854872.1	157	Short-BLUF	211
			A3JYT3	WP_005855744.1	177	Short-BLUF	212
96	*Silicibacter sp. TrichCH4B*	P(α)	C9CW04	WP_009176168.1	144	Short-BLUF	213
			C9CW06	WP_009176303.1	131	Short-BLUF	214
	Silicibacter sp.TM1040	P(α)	Q1GF07	YP_614021.1	144	Short-BLUF	215
			Q1GF09	YP_614019.1	169	Short-BLUF	216
97	*Sphingobium baderi LL03*	P(α)	T0GMM3	WP_021243582.1	138	Short-BLUF	217
98	*Sphingobium chlorophenolicum L-1*	P(α)	F6ETZ8	YP_004552311.1	138	Short-BLUF	218
99	*Sphingobium indicum B90A*	P(α)	I5BF60	WP_007684659.1	138	Short-BLUF	219
100	*Sphingobium japonicum BiD32*	P(α)	N1MG97	WP_006950198.1	129	Short-BLUF	220
	Sphingobium japonicum NBRC 101211 / UT26S	P(α)	D4Z033	YP_003544577.1	138	Short-BLUF	221
101	*Sphingobium lactosutens DS20*	P(α)	T0J2S2	WP_021225549.1	133	Short-BLUF	222
102	*Sphingobium quisquiliarum P25*	P(α)	T0H8I1	WP_021237988.1	127	Short-BLUF	223
103	*Sphingobium sp. HDIP04*	P(α)	T0G7F1	WP_020818413.1	138	Short-BLUF	224
	Sphingobium sp. YL23	P(α)		WP_022681831.1	139	Short-BLUF	225
				WP_022683884.1	136	Short-BLUF	226
				WP_022682837.1	133	Short-BLUF	227
104	*Sphingobium ummariense RL-3*	P(α)	T0IST7	WP_021318238.1	137	Short-BLUF	228
105	*Sphingobium xenophagum*	P(α)		WP_019051433.1	129	Short-BLUF	229
				WP_017182074.1	129	Short-BLUF	230
106	*Sphingomonas*	P(α)		WP_010164332.1	130	Short-BLUF	231
107	*Sphingomonas elodea*	P(α)		WP_010543334.1	131	Short-BLUF	232
108	*Sphingomonas phyllosphaerae*	P(α)		WP_022687793.1	153	Short-BLUF	233
109	*Sphingomonas sp. Mn802worker*	P(α)		WP_019517102.1	138	Short-BLUF	234
	Sphingomonas sp. PAMC 26605	P(α)		WP_010188000.1	131	Short-BLUF	235
				WP_010187435.1	215	Short-BLUF	236
				WP_010187036.1	135	Short-BLUF	237
				WP_010186219.1	148	Short-BLUF	238
	Sphingomonas sp. PAMC 26617	P(α)		WP_010163859.1	146	Short-BLUF	239
				WP_010164991.1	147	Short-BLUF	240
	Sphingomonas sp. PAMC 26621	P(α)		WP_010219263.1	240	Short-BLUF	241
				WP_010219976.1	148	Short-BLUF	242
				WP_010217326.1	141	Short-BLUF	243
				WP_010219296.1	134	Short-BLUF	244
				WP_010217042.1	165	Short-BLUF	245
				WP_010219603.1	133	Short-BLUF	246
				WP_010219403.1	147	Short-BLUF	247
				WP_010219691.1	140	Short-BLUF	248
				WP_010220036.1	81	Short-BLUF	249
	Sphingomonas sp. S17	P(α)	F3X316	WP_007406954.1	130	Short-BLUF	250
	Sphingomonas sp. SKA58	P(α)	Q1NAZ4	WP_009821301.1	133	Short-BLUF	251
110	*Sphingomonas wittichii*	P(α)		WP_016746531.1	150	Short-BLUF	252
	Sphingomonas wittichii RW1	P(α)	A5VFD3	YP_001265137.1	164	Short-BLUF	253
111	*Sphingomonas-like bacterium B12*	P(α)		WP_022691481.1	137	Short-BLUF	254
112	*Sphingopyxis alaskensis*	P(α)	Q1GWG4	YP_615341.1	141	Short-BLUF	255
113	*Sphingopyxis baekryungensis*	P(α)		WP_022672508.1	704	GGDEF+EAL+BLUF	256
				WP_022673489.1	136	Short-BLUF	257
114	*Sphingopyxis sp. MC1*	P(α)	N9WHS2	WP_003038631.1	138	Short-BLUF	258
115	*Sulfitobacter guttiformis*	P(α)	J7G2U7	YP_006965188.1	144	Short-BLUF	259
116	*Sulfitobacter sp. EE-36*	P(α)	A3S970	WP_005850501.1	110	Short-BLUF	260
	Sulfitobacter sp. NAS-14.1	P(α)	A3SU32	WP_009825579.1	110	Short-BLUF	261

117	*Variovorax paradoxus B4*	P(α)	T1X5J0	YP_008514845.1	134	Short-BLUF	262
118	*Achromobacter xylosoxidans*	P(β)	F7SWX0	WP_006391283.1	408	BLUF+EAL	263
	Achromobacter xylosoxidans NH44784-1996	P(β)	R4XQI4	YP_008030152.1	408	BLUF+EAL	264
119	*Acidovorax avenae*	P(β)		WP_019702121.1	140	Short-BLUF	265
	Acidovorax avenae ATCC 19860	P(β)	F0QCG3	YP_004236013.1	140	Short-BLUF	266
			F0Q3D8	YP_004236407.1	137	Short-BLUF	267
120	*Acidovorax avenae sub. citrulli AAC00-1*	P(β)	A1TT92	YP_971954.1	140	Short-BLUF	268
			A1TUE4	YP_972356.1	137	Short-BLUF	269
121	*Acidovorax delafieldii 2AN*	P(β)	C5TAV7	WP_005799771.1	140	Short-BLUF	270
			C5TBB6	WP_005799950.1	132	Short-BLUF	271
122	*Acidovorax ebreus TPSY*	P(β)	B9ME34	YP_002552288.1	140	Short-BLUF	272
123	*Acidovorax radicis*	P(β)		WP_010466631.1	132	Short-BLUF	273
				WP_010461918.1	140	Short-BLUF	274
				WP_010465348.1	139	Short-BLUF	275
124	*Acidovorax sp. CF316*	P(β)	J0KJ25	WP_007856976.1	132	Short-BLUF	276
			J1EI97	WP_007855842.1	140	Short-BLUF	277
			J0U4I2	WP_007857182.1	139	Short-BLUF	278
	Acidovorax sp. JS42	P(β)	A1W4E7	YP_985198.1	140	Short-BLUF	279
	Acidovorax sp. KKS102	P(β)	K0IEC4	YP_006856384.1	132	Short-BLUF	280
				YP_006853313.1	140	Short-BLUF	281
			K0I309	YP_006854227.1	139	Short-BLUF	282
	Acidovorax sp. MR-S7	P(β)		WP_020229597.1	140	Short-BLUF	283
	Acidovorax sp. NO-1	P(β)	H0C3A0	WP_008906782.1	139	Short-BLUF	284
			H0BYE8	WP_008905106.1	140	Short-BLUF	285
			H0C4J7	WP_008907226.1	132	Short-BLUF	286
125	*Alicycliphilus denitrificans DSM 14773*	P(β)	F4G8R2	YP_004386855.1	140	Short-BLUF	287
	Alicycliphilus denitrificans JCM 14587	P(β)	E8U0X9	YP_004125618.1	140	Short-BLUF	288
126	*beta proteobacterium KB13*	P(β)U	B6BTG8	WP_009852483.1	135	Short-BLUF	289
127	*beta proteobacterium L13*	P(β)		WP_017509222.1	142	Short-BLUF	290
128	*Betaproteobacteria bacterium MOLA814*	P(β)Unc		WP_023472146.1	141	Short-BLUF	291
129	*Bordetella avium (strain 197N)*	P(β)	Q2L1Z3	YP_786068.1	421	BLUF+EAL	292
130	*Brachymonas chironomi*	P(β)		WP_018715076.1	140	Short-BLUF	293
131	*Burkholderia bryophila*	P(β)		WP_020069946.1	171	Short-BLUF	294
132	*Burkholderia sp. CCGE1003*	P(β)	E1TCW7	YP_003907473.1	174	Short-BLUF	295
133	*Burkholderiales bacterium JOSHI_001*	P(β)	H5WUY1	WP_009548903.1	143	Short-BLUF	296
			H5WRM6	WP_009552427.1	157	Short-BLUF	297
			H5WLF9	WP_009549831.1	142	Short-BLUF	298
			H5WT39	WP_009553481.1	137	Short-BLUF	299
134	*Caldimonas manganoxidans*	P(β)		WP_019560888.1	143	Short-BLUF	300
135	*Chromobacterium violaceum*	P(β)	Q7NSR1	NP_903029.1	141	Short-BLUF	301
136	*Comamonadaceae bacterium CR*	P(β)		YP_008681716.1	140	Short-BLUF	302
				YP_008682224.1	144	Short-BLUF	303
137	*Comamonas testosteroni (strain CNB-2)*	P(β)	D0J3Z8	YP_003277487.1	135	Short-BLUF	304
			D0J4M9	YP_003277583.1	131	Short-BLUF	305
	Comamonas testosteroni ATCC 11996	P(β)	H1RQ19	WP_003076132.1	132	Short-BLUF	306
	Comamonas testosteroni S44	P(β)	D8DEA6	WP_003071555.1	132	Short-BLUF	307
138	*Cupriavidus basilensis OR16*	P(β)	H1S5P4	WP_006158738.1	147	Short-BLUF	308
139	*Cupriavidus necator ATCC 17699*	P(β)	Q0JZS4	YP_841480.1	147	Short-BLUF	309
	Cupriavidus necator ATCC 43291	P(β)	F8GRM4	YP_004682121.1	147	Short-BLUF	310
140	*Cupriavidus sp. BIS7*	P(β)		WP_019447941.1	147	Short-BLUF	311
	Cupriavidus sp. HMR-1	P(β)	L2ELR6	WP_008643847.1	147	Short-BLUF	312
	Cupriavidus sp. HPC(L)	P(β)	K6B9H2	WP_006577586.1	147	Short-BLUF	313
	Cupriavidus sp. UYPR2.512	P(β)		WP_018308878.1	147	Short-BLUF	314

	Cupriavidus sp. WS	P(β)		WP_020204073.1	147	Short-BLUF	315
141	*Cupriavidus taiwanensis*	P(β)		WP_018008408.1	147	Short-BLUF	316
	Cupriavidus taiwanensis LMG 19424	P(β)	B3RBI8	YP_002008317.1	147	Short-BLUF	317
142	*Curvibacter lanceolatus*	P(β)		WP_019576065.1	140	Short-BLUF	318
	Curvibacter lanceolatus	P(β)		WP_019577548.1	140	Short-BLUF	319
143	*Curvibacter putative symbiont of Hydra magnipapillata*	P(β)	C9Y8H7	CBA27840.1	156	Short-BLUF	320
			C9Y9E1	CBA28480.1	425	BLUF+EAL	321
			C9YG67	CBA33160.1	77	Short-BLUF	322
144	*Delftia acidovorans SPH-1*	P(β)	A9BX41	YP_001565411.1	133	Short-BLUF	323
145	*Delftia sp. (strain Cs1-4)*	P(β)	F6ALE0	YP_004488022.1	133	Short-BLUF	324
146	*Duganella zoogloeoides*	P(β)		WP_019920955.1	141	Short-BLUF	325
147	*Herbaspirillum frisingense GSF30*	P(β)	R0FSP4	WP_006463383.1	141	Short-BLUF	326
148	*Herbaspirillum lusitanum*	P(β)		WP_016833369.1	141	Short-BLUF	327
149	*Herbaspirillum seropedicae*	P(β)		WP_017452789.1	141	Short-BLUF	328
	Herbaspirillum seropedicae (strain SmR1)	P(β)	D8IY84	YP_003778114.1	141	Short-BLUF	329
150	*Herbaspirillum sp. CF444*	P(β)	J3D4X2	WP_007882396.1	141	Short-BLUF	330
	Herbaspirillum sp. GW103	P(β)	I3CM34	WP_008333777.1	141	Short-BLUF	331
	Herbaspirillum sp. YR522	P(β)	J3CNJ7	WP_008112004.1	141	Short-BLUF	332
151	*Herminiimonas arsenicoxydans*	P(β)	A4G7R3	CAL62550.2	116	Short-BLUF	333
152	*Hydrogenophaga sp. PBC*	P(β)		WP_009520410.1	140	Short-BLUF	334
153	*Janthinobacterium lividum*	P(β)		WP_010396398.1	135	Short-BLUF	335
				WP_010399694.1	141	Short-BLUF	336
154	*Janthinobacterium sp. CG3*	P(β)		WP_017879051.1	136	Short-BLUF	337
155	*Leptothrix cholodnii SP-6*	P(β)	B1XZW9	YP_001790861.1	141	Short-BLUF	338
156	*Limnobacter sp. MED105*	P(β)	A6GT96	WP_008252022.1	157	Short-BLUF	339
			A6GT66	WP_008251962.1	134	Short-BLUF	340
157	*Limnohabitans sp. Rim28*	P(β)		WP_019425978.1	147	Short-BLUF	341
				WP_019426427.1	140	Short-BLUF	342
	Limnohabitans sp. Rim47	P(β)		WP_019429110.1	147	Short-BLUF	343
				WP_019430089.1	140	Short-BLUF	344
158	*Methylibium petroleiphilum (strain PM1)*	P(β)	A2SEP7	YP_001020271.1	142	Short-BLUF	345
			A2SHW7	YP_001021391.1	97	Short-BLUF	346
159	*Methylotenera mobilis*	P(β)		WP_019897538.1	138	Short-BLUF	347
				WP_019896887.1	159	Short-BLUF	348
	Methylotenera mobilis JLW8	P(β)	C6WT83	YP_003049672.1	162	Short-BLUF	349
160	*Methylotenera sp. 301*	P(β)	D7DKL3	YP_003675036.1	137	Short-BLUF	350
			D7DLZ8	YP_003675269.1	199	Short-BLUF	351
161	*Methyloversatilis sp. NVD*	P(β)		WP_018410763.1	140	Short-BLUF	352
162	*Methyloversatilis universalis*	P(β)		WP_018230298.1	140	Short-BLUF	353
				WP_020164190.1	147	Short-BLUF	354
				WP_018229736.1	141	Short-BLUF	355
	Methyloversatilis universalis FAM5	P(β)	F5RDI2	WP_008061871.1	140	Short-BLUF	356
			F5RC20	WP_008060661.1	141	Short-BLUF	357
163	*Oxalobacteraceae bacterium IMCC9480*	P(β)	F1VUP6	WP_009664611.1	141	Short-BLUF	358
	Oxalobacteraceae bacterium JGI 0001004-K23	P(β)		WP_018058743.1	141	Short-BLUF	359
164	*Polaromonas naphthalenivorans CJ2*	P(β)	A1VKQ2	YP_981151.1	140	Short-BLUF	360
			A1VJM4	YP_980773.1	134	Short-BLUF	361
165	*Polaromonas sp. CF318*	P(β)	J2LWR4	WP_007863266.1	135	Short-BLUF	362
			J2U008	WP_007867959.1	140	Short-BLUF	363
			J2UJU6	WP_007862374.1	135	Short-BLUF	364
	Polaromonas sp. strain JS666	P(β)	Q12F66	YP_547724.1	140	Short-BLUF	365
			Q12FR6	YP_547524.1	135	Short-BLUF	366
166	*Polynucleobacter necessarius STIR1*	P(β)	B1XTH8	YP_001797269.1	135	Short-BLUF	367

167	*Polynucleobacter sp. QLW-P1DMWA-1*	P(β)	A4SVS3	YP_001155151.1	135	Short-BLUF	368
168	*Pseudogulbenkiania ferrooxidans EGD-HP2*	P(β)	U1AEJ0	WP_021475086.1	141	Short-BLUF	369
169	*Ralstonia eutropha H16*	P(β)	Q0JZS4	CAJ96750.1/YP_841480.1	147	Short-BLUF	370
	Ralstonia eutropha JMP134	P(β)	Q46V06	YP_297872.1	147	Short-BLUF	371
170	*Ralstonia metallidurans CH34*	P(β)	Q1LG69	YP_586126.1	147	Short-BLUF	372
171	*Ralstonia pickettii DTP0602*	P(β)		YP_008600000.1	147	Short-BLUF	373
172	*Ralstonia sp. GA3-3*	P(β)	R7XF66	EON18032.1/YP_841480.1	147	Short-BLUF	374
173	*Ramlibacter tataouinensis DSM 14655*	P(β)	F5XYH6	YP_004619171.1	225	Short-BLUF	375
			F5XWS2	YP_004620235.1	140	Short-BLUF	376
174	*Rhodocyclaceae bacterium RZ94*	P(β)		WP_019918775.1	144	Short-BLUF	377
175	*Rhodoferax ferrireducens DSM 15236*	P(β)	Q21YW7	YP_522567.1	140	Short-BLUF	378
176	*Rubrivivax benzoatilyticus JA2*	P(β)	F3LLG6	WP_009855751.1	141	Short-BLUF	379
			F3LQM5	WP_009857208.1	187	Short-BLUF	380
177	*Rubrivivax gelatinosus (strain NBRC 100245 / IL144)*	P(β)	I0HNG0	YP_005436046.1	151	Short-BLUF	381
			I0HXR5	YP_005439301.1	141	Short-BLUF	382
178	*Thauera sp. 27*	P(β)	N6YS95	WP_002936113.1	779	PAS+GGDEF+EAL+BLUF	383
179	*Thiomonas intermedia K12*	P(β)	D5X5X2	YP_003642355.1	159	Short-BLUF	384
180	*Variovorax paradoxus*	P(β)		WP_019654574.1	135	Short-BLUF	385
				WP_019656584.1	140	Short-BLUF	386
	Variovorax paradoxus (strain EPS)	P(β)	E6UYP4	YP_004157058.1	140	Short-BLUF	387
			E6UZL0	YP_004155929.1	140	Short-BLUF	388
			E6UVP8	YP_004153392.1	134	Short-BLUF	389
	Variovorax paradoxus B4	P(β)	T1XG74	YP_008518022.1	140	Short-BLUF	390
	Variovorax paradoxus S110	P(β)	C5CWX4	YP_002945997.1	140	Short-BLUF	391
			C5CNN1	YP_002942913.1	134	Short-BLUF	392
	Variovorax sp. CF313	P(β)	J2TF58	WP_007831506.1	138	Short-BLUF	393
				WP_007837143.1	140	Short-BLUF	394
181	*Verminephrobacter aporrectodeae*	P(β)		WP_010101101.1	140	Short-BLUF	395
182	*Verminephrobacter eiseniae strain EF01-2*	P(β)	A1WMH3	YP_997848.1	133	Short-BLUF	396
			A1WHJ0	YP_996115.1	142	Short-BLUF	397
183	*Acinetobacter*	P(γ)		WP_017398952.1	147	Short-BLUF	398
184	*Acinetobacter baumannii*	P(γ)	B9UN50	ACJ61333.1	125	Short-BLUF	399
				WP_000383195.1	156	Short-BLUF	400
				WP_001102891.1	156	Short-BLUF	401
				WP_019205050.1	110	Short-BLUF	402
	Acinetobacter baumannii ATCC 17978	P(γ)	A3M6V8	ABO12652.2/YP_001847089.1	156	Short-BLUF	403
	Acinetobacter baumannii 6013113	P(γ)	F5IG55	EGJ62365.1/YP_001713224.1	156	Short-BLUF	404
	Acinetobacter baumannii 6013150	P(γ)	F5I0V4	EGJ59752.1/YP_001713224.1	156	Short-BLUF	405
	Acinetobacter baumannii 6014059	P(γ)	F5INK0	EGJ67557.1/YP_001847089.1	156	Short-BLUF	406
	Acinetobacter baumannii AA-014	P(γ)	L9MMX8	ELW87667.1/YP_001847089.1	156	Short-BLUF	407
	Acinetobacter baumannii AB0057	P(γ)	B7IC80	YP_002319929.1	146	Short-BLUF	408
	Acinetobacter baumannii Ab11111	P(γ)	K1KNL6	EKB33264.1/YP_001847089.1	156	Short-BLUF	409
	Acinetobacter baumannii AB210	P(γ)	F5JQ04	EGK47564.1/YP_001847089.1	156	Short-BLUF	410
	Acinetobacter baumannii AB307-0294	P(γ)	B7H0C4	YP_002325120.1	156	Short-BLUF	411
	Acinetobacter baumannii Ab33333	P(γ)	K1L153	EKB37999.1/YP_001847089.1	156	Short-BLUF	412
	Acinetobacter baumannii Ab44444	P(γ)	K1LDN5	EKB42599.1/YP_001847089.1	156	Short-BLUF	413
	Acinetobacter baumannii ABNIH1	P(γ)	F9I778	EGT96279.1/YP_001847089.1	156	Short-BLUF	414
	Acinetobacter baumannii ABNIH10	P(γ)	M8FRG3	EMU01204.1/YP_001713224.1	156	Short-BLUF	415
	Acinetobacter baumannii ABNIH11	P(γ)	M8FCJ0	EMU03061.1/YP_001713224.1	156	Short-BLUF	416
	Acinetobacter baumannii ABNIH13	P(γ)	M8G623	EMU12871.1/YP_001847089.1	156	Short-BLUF	417
	Acinetobacter baumannii ABNIH14	P(γ)	M8G600	EMU15902.1/YP_001847089.1	156	Short-BLUF	418
	Acinetobacter baumannii ABNIH15	P(γ)	M8GPL3	EMU19730.1/YP_001847089.1	156	Short-BLUF	419
	Acinetobacter baumannii ABNIH16	P(γ)	M8HH59	EMU18738.1/YP_001847089.1	156	Short-BLUF	420

	Acinetobacter baumannii ABNIH17	P(γ)	M8I2R0	EMU36405.1/YP_001847089.1	156	Short-BLUF	421
	Acinetobacter baumannii ABNIH18	P(γ)	M8HM77	EMU34022.1/YP_001847089.1	156	Short-BLUF	422
	Acinetobacter baumannii ABNIH19	P(γ)	M8IIQ3	EMU31648.1/YP_001713224.1	156	Short-BLUF	423
	Acinetobacter baumannii ABNIH2	P(γ)	F9IL05	EGT94630.1/YP_001847089.1	156	Short-BLUF	424
	Acinetobacter baumannii ABNIH20	P(γ)	M8HYS5	EMU38102.1/YP_001847089.1	156	Short-BLUF	425
	Acinetobacter baumannii ABNIH22	P(γ)	M8IRW2	EMU37704.1/YP_001847089.1	156	Short-BLUF	426
	Acinetobacter baumannii ABNIH23	P(γ)	M8INV0	EMU43935.1/YP_001847089.1	156	Short-BLUF	427
	Acinetobacter baumannii ABNIH24	P(γ)	M8JP20	EMU48679.1/YP_001847089.1	156	Short-BLUF	428
	Acinetobacter baumannii ABNIH25	P(γ)	M8E8P5	EMT89469.1/YP_001847089.1	156	Short-BLUF	429
	Acinetobacter baumannii ABNIH26	P(γ)	M8ENA7	EMT84227.1/YP_001847089.1	156	Short-BLUF	430
	Acinetobacter baumannii ABNIH3	P(γ)	F9J0C3	EGT93760.1/YP_001847089.1	156	Short-BLUF	431
	Acinetobacter baumannii ABNIH4	P(γ)	F9J5J3	EGU03419.1/YP_001847089.1	156	Short-BLUF	432
	Acinetobacter baumannii ABNIH5	P(γ)	M8E7N4	EMT89085.1/YP_001847089.1	156	Short-BLUF	433
	Acinetobacter baumannii ABNIH6	P(γ)	M8FRA0	EMT97557.1/YP_001713224.1	156	Short-BLUF	434
	Acinetobacter baumannii ABNIH7	P(γ)	M8EIC5	EMT95636.1/YP_001713224.1	156	Short-BLUF	435
	Acinetobacter baumannii AC12	P(γ)	J2Z674	EJN44155.1/YP_001847089.1	156	Short-BLUF	436
	Acinetobacter baumannii AC30	P(γ)	K6HQG6	EKO44813.1/YP_001847089.1	156	Short-BLUF	437
	Acinetobacter baumannii ACICU	P(γ)	B2HUF8	YP_001847089.1	156	Short-BLUF	438
	Acinetobacter baumannii ANC 4097	P(γ)	N9KTG4	ENW69199.1/YP_001713224.1	156	Short-BLUF	439
	Acinetobacter baumannii ATCC 19606	P(γ)	D0C5R9	EEX04334.1/YP_007596771.1	156	Short-BLUF	440
	Acinetobacter baumannii AYE	P(γ)	B0V7Y7	YP_001713224.1	156	Short-BLUF	441
	Acinetobacter baumannii BJAB0715	P(γ)	S5CPN1	YP_008217774.1	156	Short-BLUF	442
	Acinetobacter baumannii Canada BC-5	P(γ)	J4JC81	EJO38969.1/YP_001713224.1	156	Short-BLUF	443
	Acinetobacter baumannii Canada BC1	P(γ)	K6MXH5	EKP56108.1/YP_001713224.1	156	Short-BLUF	444
	Acinetobacter baumannii D1279779	P(γ)	M4QZE8	AGH36110.1/YP_007596771.1	156	Short-BLUF	445
	Acinetobacter baumannii IS-116	P(γ)	K1F8S0	EKA67743.1/YP_001847089.1	156	Short-BLUF	446
	Acinetobacter baumannii IS-123	P(γ)	J4ZJ36	EJO37798.1/WP_001102890.1	156	Short-BLUF	447
	Acinetobacter baumannii IS-143	P(γ)	K1F3U0	EKA73961.1/YP_001847089.1	156	Short-BLUF	448
	Acinetobacter baumannii IS-235	P(γ)	K5EGT2	EKK05136.1/YP_001713224.1	156	Short-BLUF	449
	Acinetobacter baumannii IS-251	P(γ)	K5EN29	EKK16599.1/YP_001713224.1	156	Short-BLUF	450
	Acinetobacter baumannii IS-58	P(γ)	K1F7X7	EKA77090.1/YP_001713224.1	156	Short-BLUF	451
	Acinetobacter baumannii MDR-TJ	P(γ)	I1XZ18	AFI94904.1/YP_001847089.1	156	Short-BLUF	452
	Acinetobacter baumannii MDR-ZJ06	P(γ)	G2JJV4	YP_005526514.1/YP_001847089.1	156	Short-BLUF	453
	Acinetobacter baumannii MSP4-16	P(γ)	M2Z5Q5	EME56213.1/YP_007596771.1	156	Short-BLUF	454
	Acinetobacter baumannii Naval-113	P(γ)	K9C0B8	EKU64146.1/YP_001847089.1	156	Short-BLUF	455
	Acinetobacter baumannii Naval-13	P(γ)	K5PLW1	EKL51476.1/WP_001102890.1	156	Short-BLUF	456
	Acinetobacter baumannii Naval-17	P(γ)	J1MTN3	EJG29655.1/YP_001847089.1	156	Short-BLUF	457
	Acinetobacter baumannii Naval-18	P(γ)	J4UB75	EJP50580.1/WP_001102890.1	156	Short-BLUF	458
	Acinetobacter baumannii Naval-2	P(γ)	K6M6A9	EKP46927.1/YP_001847089.1	156	Short-BLUF	459
	Acinetobacter baumannii Naval-21	P(γ)	K6N8V9	EKP54045.1/YP_001713224.1	156	Short-BLUF	460
	Acinetobacter baumannii Naval-57	P(γ)	L9P2K2	ELX05830.1/YP_007596771.1	156	Short-BLUF	461
	Acinetobacter baumannii Naval-72	P(γ)	K5EE33	EKK13424.1/YP_001847089.1	156	Short-BLUF	462
	Acinetobacter baumannii Naval-78	P(γ)	L9NMD3	ELX00539.1/YP_001847089.1	156	Short-BLUF	463
	Acinetobacter baumannii Naval-81	P(γ)	K4YNM4	EJP60409.1/WP_001102890.1	156	Short-BLUF	464
	Acinetobacter baumannii Naval-82	P(γ)	K6NY26	EKP62400.1/YP_007596771.1	156	Short-BLUF	465
	Acinetobacter baumannii Naval-83	P(γ)	K5Q641	EKL58786.1/YP_001713224.1	156	Short-BLUF	466
	Acinetobacter baumannii NIPH 1362	P(γ)	N8TB24	ENU55812.1/YP_001847089.1	156	Short-BLUF	467
	Acinetobacter baumannii NIPH 146	P(γ)	N8TY29	ENU64868.1/WP_001102890.1	156	Short-BLUF	468
	Acinetobacter baumannii NIPH 1669	P(γ)	N8SXZ9	ENU51242.1/WP_001102890.1	156	Short-BLUF	469
	Acinetobacter baumannii NIPH 1734	P(γ)	N8V464	ENU77902/WP_001102890.1	156	Short-BLUF	470
	Acinetobacter baumannii NIPH 190	P(γ)	N8YYI1	ENV26434.1/YP_007596771.1	156	Short-BLUF	471
	Acinetobacter baumannii NIPH 201	P(γ)	N9GPE0	ENW36793.1/YP_001847089.1	156	Short-BLUF	472
	Acinetobacter baumannii NIPH 2061	P(γ)	N8UQL2	ENU72709.1/YP_001847089.1	156	Short-BLUF	473

	Acinetobacter baumannii NIPH 24	P(γ)	N8NHZ5	ENU14041.1/YP_001847089.1	156	Short-BLUF	474
	Acinetobacter baumannii NIPH 290	P(γ)	N9KFC5	ENW64524.1/YP_001713224.1	156	Short-BLUF	475
	Acinetobacter baumannii NIPH 329	P(γ)	N9IN83	ENW45510.1/WP_002132039.1	156	Short-BLUF	476
	Acinetobacter baumannii NIPH 329	P(γ)	N9II22	ENW43700.1/WP_004708845.1	147	Short-BLUF	477
	Acinetobacter baumannii NIPH 335	P(γ)	N9IRT6	ENW44164.1/YP_001847089.1	156	Short-BLUF	478
	Acinetobacter baumannii NIPH 410	P(γ)	S3TXG4	WP_016653868.1	156	Short-BLUF	479
	Acinetobacter baumannii NIPH 527	P(γ)	N9GQH5	ENW37177.1/YP_001713224.1	156	Short-BLUF	480
	Acinetobacter baumannii NIPH 528	P(γ)	N9JTB2	ENW57311.1/YP_001847089.1	156	Short-BLUF	481
	Acinetobacter baumannii NIPH 60	P(γ)	N8ZBA3	ENV30959/WP_001102890.1	156	Short-BLUF	482
	Acinetobacter baumannii NIPH 601	P(γ)	N9HY38	ENW52033.1/YP_007596771.1	156	Short-BLUF	483
	Acinetobacter baumannii NIPH 615	P(γ)	N8U9R8	ENU68973.1/WP_001102890.1	156	Short-BLUF	484
	Acinetobacter baumannii NIPH 67	P(γ)	N9J4Z4	WP_005131310.1	156	Short-BLUF	485
	Acinetobacter baumannii NIPH 70	P(γ)	N9KCX5	ENW63679.1/YP_001847089.1	156	Short-BLUF	486
	Acinetobacter baumannii NIPH 80	P(γ)	N9L2Z3	WP_005138547.1	156	Short-BLUF	487
	Acinetobacter baumannii OIFC0162	P(γ)	K5ET94	EKK09161.1/WP_001102887.1	156	Short-BLUF	488
	Acinetobacter baumannii OIFC021	P(γ)	L9MFF6	ELW84997.1/WP_002049354.1	156	Short-BLUF	489
	Acinetobacter baumannii OIFC021	P(γ)	L9M8U5	ELW83379.1/WP_002050218.1	141	Short-BLUF	490
	Acinetobacter baumannii OIFC032	P(γ)	J5I903	EJP41595.1/WP_001102890.1	156	Short-BLUF	491
	Acinetobacter baumannii OIFC035	P(γ)	K6NZL3	EKP69017.1/WP_002132039.1	156	Short-BLUF	492
	Acinetobacter baumannii OIFC035	P(γ)	K6NTF7	EKP66878.1/WP_002050218.1	141	Short-BLUF	493
	Acinetobacter baumannii OIFC047	P(γ)	L9NCI1	ELW97145.1/WP_001102887.1	156	Short-BLUF	494
	Acinetobacter baumannii OIFC065	P(γ)	K6LT01	EKP36620.1/YP_001847089.1	156	Short-BLUF	495
	Acinetobacter baumannii OIFC074	P(γ)	K5Q9S4	EKL46135.1/YP_001713224.1	156	Short-BLUF	496
	Acinetobacter baumannii OIFC087	P(γ)	K6LPN9	EKP41027.1/WP_001102890.1	156	Short-BLUF	497
	Acinetobacter baumannii OIFC098	P(γ)	K5PV19	EKL41434.1/YP_001847089.1	156	Short-BLUF	498
	Acinetobacter baumannii OIFC099	P(γ)	K6LQV6	EKP41452.1/WP_001102890.1	156	Short-BLUF	499
	Acinetobacter baumannii OIFC109	P(γ)	J0TMG8	EJG27711.1/WP_001102890.1	156	Short-BLUF	500
	Acinetobacter baumannii OIFC110	P(γ)	K5RVQ7	EKL57522.1/YP_001847089.1	156	Short-BLUF	501
	Acinetobacter baumannii OIFC111	P(γ)	K6M4D7	EKP46227.1/YP_001847089.1	156	Short-BLUF	502
	Acinetobacter baumannii OIFC137	P(γ)	J0S923	EJG10461.1/WP_001102890.1	156	Short-BLUF	503
	Acinetobacter baumannii OIFC143	P(γ)	J0T757	EJG19269.1/WP_001102890.1	156	Short-BLUF	504
	Acinetobacter baumannii OIFC180	P(γ)	K5RHA5	EKL52712.1/YP_001847089.1	156	Short-BLUF	505
	Acinetobacter baumannii OIFC189	P(γ)	J0SZ83	EJG19316.1/YP_001847089.1	156	Short-BLUF	506
	Acinetobacter baumannii OIFC338	P(γ)	L9N142	ELW92889.1/YP_001847089.1	156	Short-BLUF	507
	Acinetobacter baumannii strain 1656-2	P(γ)	E8PHF0	YP_001847089.1	156	Short-BLUF	508
	Acinetobacter baumannii TCDC-AB0715	P(γ)	F0QKL9	YP_005799504.1/YP_001847089.1	156	Short-BLUF	509
	Acinetobacter baumannii TYTH-1	P(γ)	K0HGX0	AFU38785.1/YP_001847089.1	156	Short-BLUF	510
	Acinetobacter baumannii WC-136	P(γ)	K9CVT9	WP_002115327.1	156	Short-BLUF	511
	Acinetobacter baumannii WC-136	P(γ)	K9CRD3	WP_002115633.1	141	Short-BLUF	512
	Acinetobacter baumannii WC-141	P(γ)	K9A8F1	WP_009389951.1	156	Short-BLUF	513
	Acinetobacter baumannii WC-141	P(γ)	K8ZYY4	WP_009387234.1	141	Short-BLUF	514
	Acinetobacter baumannii WC-323	P(γ)	K9BIS2	EKU54817/WP_005230460.1	156	Short-BLUF	515
	Acinetobacter baumannii WC-323	P(γ)	K9BM26	WP_009511737.1	155	Short-BLUF	516
	Acinetobacter baumannii WC-323	P(γ)	K9B986	WP_009515481.1	147	Short-BLUF	517
	Acinetobacter baumannii WC-323	P(γ)	K9BC39	WP_009511693.1	149	Short-BLUF	518
	Acinetobacter baumannii WC-348	P(γ)	K9B241	EKU53718.1/WP_001102887.1	156	Short-BLUF	519
	Acinetobacter baumannii WC-487	P(γ)	K9AXM6	EKU51999/WP_006580584.1	156	Short-BLUF	520
	Acinetobacter baumannii WC-487	P(γ)	K9BLE6	EKU60294.1/WP_002050218.1	141	Short-BLUF	521
	Acinetobacter baumannii WC-692	P(γ)	K1ESG1	EKA71749.1/YP_001847089.1	156	Short-BLUF	522
	Acinetobacter baumannii WC-A-694	P(γ)	K6MFE1	EKP63369.1/WP_001102890.1	156	Short-BLUF	523
	Acinetobacter baumannii WC-A-92	P(γ)	L9M673	WP_001102886.1	156	Short-BLUF	524
	Acinetobacter baumannii ZWS1122	P(γ)	K2ILA9	EKE59799.1/YP_001847089.1	156	Short-BLUF	525
	Acinetobacter baumannii ZWS1219	P(γ)	K2HQ55	EKE59891.1/YP_001847089.1	156	Short-BLUF	526

185	*Acinetobacter baylyi DSM 14961 = CIP 107474*	P(γ)	N9B659	YP_046740.1	155	Short-BLUF	527
			N9BCT4	WP_004927632.1	148	Short-BLUF	528
			N9BED0	YP_046178.1	149	Short-BLUF	529
			N8ZXW2	WP_004927624.1	155	Short-BLUF	530
186	*Acinetobacter beijerinckii ANC 3835*	P(γ)	N9FTS1	WP_005051584.1	157	Short-BLUF	531
			N9F6Z4	WP_005055777.1	155	Short-BLUF	532
			N9FJQ5	WP_005054245.1	138	Short-BLUF	533
			N9FDZ0	WP_005055778.1	149	Short-BLUF	534
	Acinetobacter beijerinckii CIP 110307	P(γ)	N9FHE8	WP_005059436.1	157	Short-BLUF	535
187	*Acinetobacter bereziniae CIP 70.12*	P(γ)	N9F616	WP_005029839.1	155	Short-BLUF	536
			N9F7L1	ENW00876.1/WP_004830862.1	147	Short-BLUF	537
			N9DPW4	WP_005029837.1	149	Short-BLUF	538
	Acinetobacter bereziniae NIPH 3	P(γ)	N8YPM4	ENV21180.1/WP_004831317.1	155	Short-BLUF	539
			N8YQK9	ENV21525.1/WP_004830862.1	147	Short-BLUF	540
			N8YIN9	ENV21179.1/WP_004831316.1	149	Short-BLUF	541
188	*Acinetobacter calcoaceticus*	P(γ)		WP_017392001.1	156	Short-BLUF	542
				WP_017392001.1	156	Short-BLUF	543
				WP_017480957.1	89	Short-BLUF	544
	Acinetobacter calcoaceticus (strain PHEA-2)	P(γ)	F0KGE8	YP_004995739.1	141	Short-BLUF	545
			F0KII1	ADY82317.1/YP_004995999.1	156	Short-BLUF	546
	Acinetobacter calcoaceticus ANC 3680	P(γ)	N9EC09	WP_005039435.1	156	Short-BLUF	547
	Acinetobacter calcoaceticus ANC 3811	P(γ)	R8Y6J9	WP_016137597.1	156	Short-BLUF	548
			R8Y874	WP_016138168.1	161	Short-BLUF	549
	Acinetobacter calcoaceticus DSM 30006 = CIP 81.8	P(γ)	N9F0B6	ENV98323.1/WP_004643428.1	156	Short-BLUF	550
	Acinetobacter calcoaceticus NIPH 13	P(γ)	N8PJK1	ENU09837.1/WP_004643428.1	156	Short-BLUF	551
	Acinetobacter calcoaceticus RUH2202	P(γ)	D0S286	WP_003650777.1	156	Short-BLUF	552
189	*Acinetobacter gerneri*	P(γ)	N8YFI2	WP_004871730.1	157	Short-BLUF	553
190	*Acinetobacter guillouiae CIP 63.46*	P(γ)	N8S7G3	ENU59170.1/WP_004719325.1	142	Short-BLUF	554
			N8THK4	ENU57977.1/WP_004721233.1	155	Short-BLUF	555
			N8S437	WP_004721230.1	149	Short-BLUF	556
	Acinetobacter guillouiae MSP4-18	P(γ)	S3Z3U9	EPH37159.1/WP_004719325.1	142	Short-BLUF	557
			S3ZKX5	EPH38960.1/WP_004721233.1	155	Short-BLUF	558
	Acinetobacter guillouiae NIPH 991	P(γ)	N8WUW8	WP_004822451.1	142	Short-BLUF	559
			N8WXK7	WP_004820747.1	155	Short-BLUF	560
			N8YB72	WP_004820748.1	149	Short-BLUF	561
191	*Acinetobacter gyllenbergii*	P(γ)		WP_023269848.1	147	Short-BLUF	562
				WP_023269642.1	155	Short-BLUF	563
	Acinetobacter gyllenbergii CIP 110306	P(γ)	S3PY91	EPF83591.1/WP_016539987.1	155	Short-BLUF	564
			S3PP26	EPF88044.1/WP_016540194.1	147	Short-BLUF	565
	Acinetobacter gyllenbergii MTCC 11365	P(γ)	S3YW62	EPH35666.1/WP_016539987.1	155	Short-BLUF	566
			S3ZBX9	EPH35880.1/WP_016540194.1	147	Short-BLUF	567
192	*Acinetobacter haemolyticus ATCC 19194*	P(γ)	D4XPD9	WP_004638534.1	128	Short-BLUF	568
	Acinetobacter haemolyticus CIP 64.3	P(γ)	N9F599	WP_005082086.1	141	Short-BLUF	569
	Acinetobacter haemolyticus NIPH 261	P(γ)	N9GP72	WP_005090981.1	141	Short-BLUF	570
193	*Acinetobacter indicus ANC 4215*	P(γ)	S3N2L2	WP_016659098.1	144	Short-BLUF	571
194	*Acinetobacter johnsonii ANC 3681*	P(γ)	N9CQ68	WP_004982048.1	141	Short-BLUF	572
	Acinetobacter johnsonii CIP 64.6	P(γ)	N8RTR2	WP_004696426.1	141	Short-BLUF	573
	Acinetobacter johnsonii SH046	P(γ)	D0SCH6	WP_005400678.1	156	Short-BLUF	574
195	*Acinetobacter lwoffii*	P(γ)		WP_010327040.1	141	Short-BLUF	575
				WP_016806942.1	156	Short-BLUF	576
	Acinetobacter lwoffii CIP 70.31	P(γ)	N9HDC8	ENW29835.1/WP_004278796.1	143	Short-BLUF	577
			N9H602	ENW27250.1/WP_005104921.1	157	Short-BLUF	578
			N9G8Z9	WP_005102339.1	149	Short-BLUF	579

	Acinetobacter lwoffii NCTC 5866 = CIP 64.10	P(γ)	N9FL15	ENW23433.1/WP_005097246.1	143	Short-BLUF	580
			N9FRT2	ENW25392.1/WP_004281912.1	157	Short-BLUF	581
	Acinetobacter lwoffii NIPH 478	P(γ)	N9GA40	WP_005106614.1	143	Short-BLUF	582
			N9G1N1	ENW28923 /WP_004281912.1	157	Short-BLUF	583
	Acinetobacter lwoffii NIPH 715	P(γ)	N8TVU5	ENU62389.1/WP_004278796.1	143	Short-BLUF	584
			N8TZW9	ENU64062.1/WP_004729009.1	157	Short-BLUF	585
	Acinetobacter lwoffii SH145	P(γ)	D0SZQ9	EEY88544.1/WP_004281912.1	157	Short-BLUF	586
			D0ST39	EEY90693.1/WP_004278796.1	143	Short-BLUF	587
196	*Acinetobacter nectaris*	P(γ)		WP_023272517.1	164	Short-BLUF	588
				WP_023274063.1	154	Short-BLUF	589
197	*Acinetobacter nosocomialis*	P(γ)		WP_006581725.1	150	Short-BLUF	590
				WP_017398279.1	158	Short-BLUF	591
				WP_016804882.1	192	Short-BLUF	592
				WP_017392774.1	158	Short-BLUF	593
	Acinetobacter nosocomialis Ab22222	P(γ)	K2PWD6	WP_006580584.1	156	Short-BLUF	594
			K2PVW2	WP_006581982.1	147	Short-BLUF	595
	Acinetobacter nosocomialis NIPH 2119	P(γ)	N8RB52	ENU48161/WP_002049354.1	156	Short-BLUF	596
			N8SP20	ENU47659.1/WP_004708845.1	147	Short-BLUF	597
	Acinetobacter nosocomialis NIPH 386	P(γ)	N8YWQ6	WP_004886335.1	156	Short-BLUF	598
			N8YS47	ENV39612.1/WP_004708845.1	147	Short-BLUF	599
198	*Acinetobacter oleivorans*	P(γ)		WP_023274242.1	147	Short-BLUF	600
199	*Acinetobacter parvus DSM 16617 = CIP 108168*	P(γ)	N8QCF2	WP_004682504.1	142	Short-BLUF	601
200	*Acinetobacter pittii*	P(γ)		WP_023187856.1	141	Short-BLUF	602
	Acinetobacter pittii ANC 3678	P(γ)	N9G3B5	WP_005068354.1	156	Short-BLUF	603
			N9ENJ6	WP_005068879.1	147	Short-BLUF	604
	Acinetobacter pittii ANC 4050	P(γ)	R8YQG3	WP_016140521.1	156	Short-BLUF	605
			R8YMS0	WP_016140719.1	147	Short-BLUF	606
	Acinetobacter pittii ANC 4052	P(γ)	R8YV96	WP_016145865.1	156	Short-BLUF	607
			R8YV13	WP_016145659.1	147	Short-BLUF	608
	Acinetobacter pittii CIP 70.29	P(γ)	N9EKI1	YP_004995999.1	156	Short-BLUF	609
			N9EKQ9	WP_005073889.1	147	Short-BLUF	610
201	*Acinetobacter radioresistens*	P(γ)		WP_017401064.1	208	Short-BLUF	611
				WP_017401136.1	185	Short-BLUF	612
	Acinetobacter radioresistens DSM 6976	P(γ)	K6W0U9	WP_005404197.1	140	Short-BLUF	613
			K6VGN5	ENV88969.1/WP_005025739.1	146	Short-BLUF	614
			K6UYR6	WP_005025735.1	147	Short-BLUF	615
			K6UIB5	WP_005025720.1	158	Short-BLUF	616
	Acinetobacter radioresistens NIPH 2130	P(γ)	N9E4V1	WP_005014499.1	146	Short-BLUF	617
			N9DVX6	WP_005019483.1	146	Short-BLUF	618
			N9E4J8	WP_005014506.1	147	Short-BLUF	619
	Acinetobacter radioresistens SH164	P(γ)	D0T1D2	EEY87475.1/WP_005014499.1	146	Short-BLUF	620
			D0T5A8	EEY86881.1/WP_005019483.1	146	Short-BLUF	621
			D0T1Y5	EEY87678.1/WP_005404197.1	140	Short-BLUF	622
			D0T1A1	WP_005404152.1	152	Short-BLUF	623
			D0T1D4	WP_005404170.1	154	Short-BLUF	624
			D0T1E1	WP_005014522.1	118	Short-BLUF	625
	Acinetobacter radioresistens SK82	P(γ)	C6RNE8	EET82638.1/WP_005404197.1	140	Short-BLUF	626
			C6RPU2	WP_005405185.1	148	Short-BLUF	627
			C6RLZ1	EET83186.1/WP_005014522.1	118	Short-BLUF	628
			C6RQ12	EET82088.1/WP_005019483.1	146	Short-BLUF	629
			C6RLZ9	EET83194.1/WP_005014499.1	146	Short-BLUF	630
			C6RLZ8	EET83193.1/WP_005014506.1	147	Short-BLUF	631
	Acinetobacter radioresistens WC-A-157	P(γ)	J4ZH05	WP_005405555.1	140	Short-BLUF	632

			J4ZPX4	EJO37381.1/WP_005025739.1	146	Short-BLUF	633
			J4Z2C2	WP_005407064.1	146	Short-BLUF	634
			J4JB98	WP_005405508.1	148	Short-BLUF	635
			J4ZHC1	WP_005405601.1	158	Short-BLUF	636
202	*Acinetobacter schindleri CIP 107287*	P(γ)	N9AFC9	WP_004892906.1	142	Short-BLUF	637
			N8ZAE4	ENV45921.1/WP_004897488.1	156	Short-BLUF	638
	Acinetobacter schindleri NIPH 900	P(γ)	N8XZD8	ENV12748.1/WP_004815687.1	142	Short-BLUF	639
			N8WNA0	WP_004813968.1	156	Short-BLUF	640
203	*Acinetobacter soli CIP 110264*	P(γ)	N9BKH9	WP_004938488.1	155	Short-BLUF	641
			N9A8X8	WP_004937197.1	148	Short-BLUF	642
			N9BMU6	ENV57428.1/WP_004937200.1	148	Short-BLUF	643
			N9BJV1	WP_004938129.1	155	Short-BLUF	644
			N9A568	ENV56437.1/WP_004938066.1	152	Short-BLUF	645
	Acinetobacter soli CIP 110264	P(γ)	N9A5P2	ENV56587.1/WP_004938485.1	150	Short-BLUF	646
	Acinetobacter soli NIPH 2899	P(γ)	N9AFQ3	WP_004947954.1	155	Short-BLUF	647
			N9AFF8	WP_004946942.1	148	Short-BLUF	648
			N9BN22	ENV59724.1/WP_004937200.1	148	Short-BLUF	649
			N9AFI2	ENV60082.1/WP_004938066.1	152	Short-BLUF	650
			N9AGU9	ENV60181.1/WP_004938485.1	150	Short-BLUF	651
204	*Acinetobacter sp. 93A2*	P(γ)	Q933L6	YP_046178.1	149	Short-BLUF	652
	Acinetobacter sp. ADP1	P(γ)	Q6FAI1	YP_046754.1	149	Short-BLUF	653
			Q6FAJ5	CAG68918.1/YP_046740.1	155	Short-BLUF	654
			Q6FAH9	YP_046756.1	163	Short-BLUF	655
			Q7BC36	CAG68356.1/YP_046178.1	149	Short-BLUF	656
	Acinetobacter sp. ANC 3880	P(γ)	N9T085	ENX57062.1/WP_005189133.1	155	Short-BLUF	657
			N9T0N2	ENX56900.1/WP_004807238.1	148	Short-BLUF	658
			N9RGG2	ENX57063.1/WP_005189145.1	149	Short-BLUF	659
	Acinetobacter sp. ANC 3929	P(γ)	N9LK71	WP_005152839.1	147	Short-BLUF	660
	Acinetobacter sp. ANC 3994	P(γ)	N8QCC6	WP_004648902.1	145	Short-BLUF	661
	Acinetobacter sp. ANC 4105	P(γ)	N9MQW8	ENW92349.1/WP_005189133.1	155	Short-BLUF	662
			N9L7C0	WP_005188667.1	148	Short-BLUF	663
			N9MGG1	ENW92350.1/WP_005189145.1	149	Short-BLUF	664
	Acinetobacter sp. ATCC 27244	P(γ)	C0VK32	WP_008940976.1	141	Short-BLUF	665
	Acinetobacter sp. CIP 101934	P(γ)	N9NFB7	ENX00710.1/WP_004815687.1	142	Short-BLUF	666
			N9NKY1	ENX03417.1/WP_004897488.1	156	Short-BLUF	667
	Acinetobacter sp. CIP 101966	P(γ)	N9P0F1	WP_005263266.1	143	Short-BLUF	668
			N9NWM1	ENX25468/WP_005104921.1	157	Short-BLUF	669
	Acinetobacter sp. CIP 102082	P(γ)	N8WM85	ENU96377.1/WP_004757740.1	142	Short-BLUF	670
	Acinetobacter sp. CIP 102129	P(γ)	N8VLK0	WP_004761518.1	142	Short-BLUF	671
	Acinetobacter sp. CIP 102136	P(γ)	N9PPP9	WP_005252160.1	143	Short-BLUF	672
			N9NCH2	ENX18668.1//WP_004729009.1	157	Short-BLUF	673
	Acinetobacter sp. CIP 102143	P(γ)	N9TH59	ENX62685.1/WP_004757740.1	142	Short-BLUF	674
	Acinetobacter sp. CIP 102159	P(γ)	N8U5M8	ENU82816.1/WP_004757740.1	142	Short-BLUF	675
	Acinetobacter sp. CIP 102529	P(γ)	N8UNT2	ENU89135.1/WP_004757740.1	142	Short-BLUF	676
	Acinetobacter sp. CIP 102637	P(γ)	N8XF19	WP_004799395.1	142	Short-BLUF	677
	Acinetobacter sp. CIP 110321	P(γ)	R9AZG9	WP_016163638.1	148	Short-BLUF	678
	Acinetobacter sp. CIP 51.11	P(γ)	N9MYK0	WP_005247271.1	143	Short-BLUF	679
			N9N647	WP_005245564.1	157	Short-BLUF	680
	Acinetobacter sp. CIP 53.82	P(γ)	N9MHW6	WP_005178747.1	144	Short-BLUF	681
	Acinetobacter sp. CIP 56.2	P(γ)	N8XEK4	ENV07524.1/WP_004807238.1	148	Short-BLUF	682
	Acinetobacter sp. CIP 64.2	P(γ)	N9N2S2	WP_005241169.1	155	Short-BLUF	683
			N9PKT7	WP_005241451.1	147	Short-BLUF	684
			N9PKB9	WP_005241167.1	148	Short-BLUF	685

	Acinetobacter sp. CIP 64.7	P(γ)	N9QVJ7	ENX30917.1/WP_004278796.1	143	Short-BLUF	686
			N9QHH3	ENX26357.1/WP_004729009.1	157	Short-BLUF	687
	Acinetobacter sp. CIP 70.18	P(γ)	N9ST00	ENX57771.1/WP_005189133.1	155	Short-BLUF	688
			N9T3L2	ENX57935.1/WP_004807238.1	148	Short-BLUF	689
	Acinetobacter sp. CIP A162	P(γ)	N8Q5C3	WP_004645416.1	143	Short-BLUF	690
			N8Q0C9	ENU15332.1/WP_004281912.1	157	Short-BLUF	691
	Acinetobacter sp. COS3	P(γ)		WP_023013276.1	148	Short-BLUF	692
	Acinetobacter sp. DR1	P(γ)	D8JK77	YP_003732271.1	161	Short-BLUF	693
			D8JFY6	YP_003731440.1	156	Short-BLUF	694
	Acinetobacter sp. GG2	P(γ)		WP_019458416.1	156	Short-BLUF	695
				WP_019459106.1	161	Short-BLUF	696
	Acinetobacter sp. HA	P(γ)	I4ZUE1	EIM39833.1/WP_004815687.1	142	Short-BLUF	697
			I4ZQ16	WP_008306942.1	156	Short-BLUF	698
	Acinetobacter sp. MDS7A	P(γ)		WP_019836199.1	157	Short-BLUF	699
	Acinetobacter sp. NBRC 100985	P(γ)	G7GFE4	WP_007482358.1	148	Short-BLUF	700
			G7GDM0	WP_007481279.1	155	Short-BLUF	701
	Acinetobacter sp. NIPH 1847	P(γ)	N9NUS5	ENX05670.1/WP_005230460.1	156	Short-BLUF	702
			N9MA43	WP_005230039.1	153	Short-BLUF	703
			N9MAQ2	WP_005230366.1	147	Short-BLUF	704
			N9NRU6	WP_005230037.1	149	Short-BLUF	705
	Acinetobacter sp. NIPH 1859	P(γ)	N9R7K2	WP_005270554.1	147	Short-BLUF	706
	Acinetobacter sp. NIPH 1867	P(γ)	N9R0C8	WP_005211997.1	155	Short-BLUF	707
			N9SKL2	WP_005212260.1	148	Short-BLUF	708
			N9SJJ0	WP_005211995.1	149	Short-BLUF	709
	Acinetobacter sp. NIPH 2036	P(γ)	S3T8J5	WP_016652452.1	147	Short-BLUF	710
	Acinetobacter sp. NIPH 2100	P(γ)	N9RFY9	WP_005293946.1	147	Short-BLUF	711
	Acinetobacter sp. NIPH 2168	P(γ)	N9Q857	WP_005257675.1	156	Short-BLUF	712
			N9Q7N9	WP_005257454.1	147	Short-BLUF	713
	Acinetobacter sp. NIPH 2171	P(γ)	N9MH86	WP_005236029.1	143	Short-BLUF	714
			N9MRD9	ENX11134/WP_004780330.1	156	Short-BLUF	715
			N9MLJ6	ENX09453.1/WP_004782875.1	146	Short-BLUF	716
			N9P2N4	WP_005235635.1	147	Short-BLUF	717
	Acinetobacter sp. NIPH 3623	P(γ)	N9RE67	ENX36920.1/WP_005230460.1	156	Short-BLUF	718
			N9PWE1	WP_005285390.1	155	Short-BLUF	719
			N9PX04	WP_005285742.1	147	Short-BLUF	720
			N9PWA3	WP_005285387.1	149	Short-BLUF	721
	Acinetobacter sp. NIPH 542	P(γ)	N9RMM9	WP_005306937.1	156	Short-BLUF	722
			N9QI40	WP_005305097.1	147	Short-BLUF	723
	Acinetobacter sp. NIPH 713	P(γ)	N9KUS9	ENW87773.1/WP_005097246.1	143	Short-BLUF	724
			N9KN55	WP_005173613.1	149	Short-BLUF	725
	Acinetobacter sp. NIPH 758	P(γ)	N8UVT4	WP_004772199.1	156	Short-BLUF	726
			N8W7V8	WP_004772105.1	147	Short-BLUF	727
	Acinetobacter sp. NIPH 809	P(γ)	N8QJS9	WP_004653704.1	155	Short-BLUF	728
			N8QQ22	WP_004653401.1	147	Short-BLUF	729
			N8QQ45	WP_004653706.1	149	Short-BLUF	730
	Acinetobacter sp. NIPH 817	P(γ)	N8VUX6	WP_004791296.1	156	Short-BLUF	731
			N8VSG1	WP_004792481.1	161	Short-BLUF	732
	Acinetobacter sp. NIPH 899	P(γ)	N8WTX6	WP_004783886.1	143	Short-BLUF	733
			N8VMF9	ENV00711.1/WP_004780330.1	156	Short-BLUF	734
			N8VIL1	ENU99395.1/WP_004782875.1	146	Short-BLUF	735
	Acinetobacter sp. NIPH 973	P(γ)	N8SCA4	WP_004700647.1	156	Short-BLUF	736
	Acinetobacter sp. P8-3-8	P(γ)		WP_010115938.1	149	Short-BLUF	737
	Acinetobacter sp. RUH2624	P(γ)	D0BZX2	EEX00046.1/WP_004708845.1	147	Short-BLUF	738

			D0BX54	EEX01065.1/WP_006580584.1	156	Short-BLUF	739
	Acinetobacter sp. SH024	P(γ)	D6JU40	EFF86339.1/WP_002115327.1	156	Short-BLUF	740
			D6JTD2	WP_005803474.1	147	Short-BLUF	741
	Acinetobacter sp. WC-743	P(γ)	L9MH55	ELW86565.1/WP_004831317.1	155	Short-BLUF	742
			L9ME93	ELW84587.1/WP_004830862.1	147	Short-BLUF	743
			L9ME60	WP_009585312.1	147	Short-BLUF	744
			L9MHY5	ELW86514.1/WP_004831316.1	149	Short-BLUF	745
205	*Acinetobacter tandoii DSM 14970 = CIP 107469*	P(γ)	R9B235	WP_016166413.1	140	Short-BLUF	746
206	*Acinetobacter ursingii ANC 3649*	P(γ)	N9DFL6	WP_005004065.1	141	Short-BLUF	747
			N9DGY2	WP_005002881.1	155	Short-BLUF	748
	Acinetobacter ursingii DSM 16037 = CIP 107286	P(γ)	N9D109	WP_004989315.1	141	Short-BLUF	749
			N9D4M0	ENV75720.1/WP_004990933.1	155	Short-BLUF	750
	Acinetobacter ursingii NIPH 706	P(γ)	N9QSA7	WP_004997440.1	141	Short-BLUF	751
			N9QT74	ENX49024.1/WP_004990933.1	155	Short-BLUF	752
207	*Acinetobacter venetianus*	P(γ)		WP_017196667.1	147	Short-BLUF	753
				WP_019384317.1	148	Short-BLUF	754
	Acinetobacter venetianus RAG-1 = CIP 110063	P(γ)	N8ZT40	WP_004879195.1	148	Short-BLUF	755
208	*Algicola sagamiensis*	P(γ)		WP_018691603.1	138	Short-BLUF	756
				WP_018694364.1	138	Short-BLUF	757
				WP_018694365.1	140	Short-BLUF	758
209	*Alishewanella agri BL06*	P(γ)	I9DUG2	WP_008983735.1	137	Short-BLUF	759
210	*Alishewanella jeotgali KCTC 22429*	P(γ)	H3ZIX1	WP_008951845.1	137	Short-BLUF	760
211	*Allochromatium vinosum DSM 180*	P(γ)	D3RRS9	YP_003443015.1	1124	PAS+GGDEF+EAL+BLUF	761
			D3RMV8	YP_003444278.1	143	Short-BLUF	762
212	*Alteromonadales bacterium TW-7*	P(γ)	A0XX52	WP_006791331.1	141	Short-BLUF	763
214	*Alteromonas macleodii 'Deep ecotype'*	P(γ)	F2G2W2	AEA96322.1/YP_004425320.1	390	BLUF+EAL	764
	Alteromonas macleodii (strain Balearic Sea AD45)	P(γ)	K0EAG8	YP_006801094.1	395	BLUF+EAL	765
	Alteromonas macleodii (strain Black Sea 11)	P(γ)	K0CTD4	YP_006823174.1	395	BLUF+EAL	766
	Alteromonas macleodii (strain English Channel 673)	P(γ)	K0CJN8	YP_006797210.1	395	BLUF+EAL	767
	Alteromonas macleodii AltDE1	P(γ)	K7RG75	YP_004425320.1	390	BLUF+EAL	768
	Alteromonas macleodii ATCC 27126	P(γ)	J9Y701	YP_006746245.1	405	BLUF+EAL	769
	Alteromonas macleodii str. 'Aegean Sea MED64'	P(γ)	S5ATU0	AGP80153.1	390	BLUF+EAL	770
	Alteromonas macleodii str. 'Ionian Sea U4'	P(γ)	S5AU17	YP_008170230.1	405	BLUF+EAL	771
	Alteromonas macleodii str. 'Ionian Sea U8'	P(γ)	S5BLE9	YP_008170230.1	405	BLUF+EAL	772
215	*Beggiatoa sp. PS*	P(γ)	A7BT71	EDN70265.1	350	BLUF+Cyclase	773
216	*Bermanella marisrubri*	P(γ)	Q1N3J0	WP_007018774.1	163	Short-BLUF	774
217	*Cedecea davisae*	P(γ)		WP_016535954.1	401	BLUF+EAL	775
218	*Cellvibrio sp. BR*	P(γ)	I3IDC4	WP_007638332.1	179	Short-BLUF	776
219	*Citrobacter freundii 4_7_47CFAA*	P(γ)	G9SK57	WP_003833502.1	409	BLUF+EAL	777
	Citrobacter freundii ATCC 8090 = MTCC 1658	P(γ)	K8QN37	WP_003841532.1	409	BLUF+EAL	778
	Citrobacter freundii GTC 09479	P(γ)	M3DUI2	WP_003844033.1	409	BLUF+EAL	779
	Citrobacter freundii GTC 09629	P(γ)	R1HFL0	EOD62395.1/WP_003020858.1	409	BLUF+EAL	780
220	*Citrobacter rodentium (strain ICC168)*	P(γ)	D2TIH4	YP_003365120.1	406	BLUF+ EAL	781
221	*Citrobacter sp. 30_2*	P(γ)	C1M5D7	WP_008784992.1	409	BLUF+ EAL	782
	Citrobacter sp. A1	P(γ)	J0M453	EJF22671.1/WP_003020858.1	409	BLUF+EAL	783
	Citrobacter sp. KTE151	P(γ)	R8WTE8	WP_016153346.1	403	BLUF+EAL	784
	Citrobacter sp. KTE30	P(γ)	R8UVC4	WP_016150366.1	409	BLUF+EAL	785
	Citrobacter sp. KTE32	P(γ)	R8V9S4	WP_016156768.1	403	BLUF+EAL	786
	Citrobacter sp. L17	P(γ)	K8ZLA8	EKU35574.1/WP_003020858.1	409	BLUF+EAL	787
222	*Citrobacter youngae ATCC 29220*	P(γ)	D4B8N0	WP_006684233.1	403	BLUF+ EAL	788
223	*Congregibacter litoralis KT71*	P(γ)U	A4A4X0	EAQ98841.1/WP_008294319.1	160	Short-BLUF	789
			A4ACN4	WP_008296304.1	120	Short-BLUF	790
			A4A4X0	EAQ98841.1/WP_008294319.1	160	Short-BLUF	791

224	*Cronobacter dublinensis 1210*	**P**(γ)	K8AWY9	WP_007716320.1	404	BLUF+EAL	792
	Cronobacter dublinensis 582	**P**(γ)	K8AXH9	WP_007750072.1	404	BLUF+EAL	793
225	*Cronobacter sakazakii E899*	**P**(γ)	F5VN29	WP_004387288.1	403	BLUF+EAL	794
226	*Cronobacter turicensis 564*	**P**(γ)	K8BZ68	WP_007767450.1	362	BLUF+EAL	795
	Cronobacter turicensis DSM 18703 / LMG 23827 / z3032	**P**(γ)	C9XVD7	YP_003211056.1	404	BLUF+ EAL	796
			C9Y5C1	YP_003212605.1	405	BLUF+ EAL	797
227	*Ectothiorhodospira sp. PHS-1*	**P**(γ)	H1G5T1	WP_008932735.1	1090	PAS+GGDEF+EAL+BLUF	798
228	*Endozoicomonas elysicola*	**P**(γ)		WP_020581308.1	159	Short-BLUF	799
229	*Enhydrobacter aerosaccus SK60*	**P**(γ)	C8PY76	WP_007116298.1	190	Short-BLUF	800
			C8PVS3	WP_007115447.1	168	Short-BLUF	801
			C8PY73	WP_007116295.1	143	Short-BLUF	802
			C8Q1B6	WP_007117385.1	217	Short-BLUF	803
			C8PX01	WP_007115875.1	192	Short-BLUF	804
			C8PY74	WP_007116296.1	191	Short-BLUF	805
230	*Enterobacter cloacae subsp. Cloacae strain ATCC 13047*	**P**(γ)	D5CBV1	YP_003612864.1	406	BLUF+ EAL	806
231	*Enterobacter sp. 638*	**P**(γ)	A4WAH8	YP_001176759.1	406	BLUF+ EAL	807
			A4W9Q6	YP_001176487.1/NP_415681.1	418	BLUF+ EAL	808
232	*Enterobacter aerogenes*	**P**(γ)		WP_020079328.1	403	BLUF+EAL	809
	Enterobacter aerogenes ATCC 13048	**P**(γ)	G0EBP4	YP_004593064.1	403	BLUF+EAL	810
	Enterobacter aerogenes EA1509E	**P**(γ)	L8BJB1	YP_007388638.1	**403**	BLUF+EAL	811
233	*Enterobacter asburiae (strain LF7a)*	**P**(γ)	G2SAH3	YP_004828302.1	406	BLUF+EAL	812
			G2S426	YP_004828705.1	404	BLUF+EAL	813
234	*Enterobacter cancerogenus ATCC 35316*	**P**(γ)	D2ZBH9	WP_006174898.1	406	BLUF+ EAL	814
235	*Enterobacter cloacae*	**P**(γ)		WP_023305984.1	406	BLUF+EAL	815
				WP_023299928.1	406	BLUF+EAL	816
				WP_023311325.1	406	BLUF+EAL	817
				WP_023478840.1	410	BLUF+EAL	818
				WP_017457087.1	407	BLUF+EAL	819
				WP_017456801.1	406	BLUF+EAL	820
236	*Enterobacter cloacae complex*	**P**(γ)		WP_022647870.1	406	BLUF+EAL	821
				WP_023292651.1	406	BLUF+EAL	822
	Enterobacter cloacae EC_38VIM1	**P**(γ)	S9ZFC8	WP_021241106.1	406	BLUF+EAL	823
	Enterobacter cloacae EcWSU1	**P**(γ)	G8LLE0	YP_004951716.1	406	BLUF+EAL	824
237	*Enterobacter cloacae subsp. cloacae ENHKU01*	**P**(γ)	J7GAN8	YP_006578373.1	406	BLUF+EAL	825
	Enterobacter cloacae subsp. cloacae GS1	**P**(γ)	I4ZKI1	WP_003857746.1	406	BLUF+EAL	826
	Enterobacter cloacae subsp. Cloacae NCTC 9394	**P**(γ)	D6DT37	YP_007845256.1	406	BLUF+ EAL	827
238	*Enterobacter cloacae subsp. dissolvens SDM*	**P**(γ)	I6SD99	YP_006477315.1	406	BLUF+EAL	828
239	*Enterobacter hormaechei ATCC 49162*	**P**(γ)	F5RSE2	WP_006809041.1	406	BLUF+EAL	829
240	*Enterobacter mori*	**P**(γ)		WP_010430222.1	406	BLUF+EAL	830
241	*Enterobacter radicincitans DSM 16656*	**P**(γ)	J1QQB0	WP_007375061.1	413	BLUF+EAL	831
242	*Enterobacter sakazakii ATCC BAA-894*	**P**(γ)	A7MRH4	YP_001440495.1	406	BLUF+ EAL	832
			A7MJM6	YP_001437322.1	403	BLUF+ EAL	833
243	*Enterobacter sp. Ag1*	**P**(γ)	J0W5B8	WP_008456677.1	396	BLUF+EAL	834
	Enterobacter sp. MGH 14	**P**(γ)		WP_022650917.1	406	BLUF+EAL	835
				WP_023335406.1	406	BLUF+EAL	836
				WP_023327186.1	406	BLUF+EAL	837
	Enterobacter sp. R4-368	**P**(γ)	R9VFU3	YP_008106092.1	407	BLUF+EAL	838
	Enterobacter sp. SST3	**P**(γ)	K4YIM7	WP_008500633.1	406	BLUF+EAL	839
244	*Enterobacteriaceae bacterium LSJC7*	**P**(γ)		WP_017372627.1	408	BLUF+EAL	840
245	*Enterovibrio calviensis*	**P**(γ)		WP_017010526.1	136	Short-BLUF	841
246	*Enterovibrio norvegicus*	**P**(γ)		WP_017003299.1	138	Short-BLUF	842
				WP_016960395.1	138	Short-BLUF	843
				WP_017004081.1	138	Short-BLUF	844

				WP_016961429.1	136	Short-BLUF	845
247	*Erwinia billingiae (strain Eb661)*	**P**(γ)	D8MSM3	YP_003741677.1	405	BLUF+EAL	846
248	*Erwinia tasmaniensis Et1/99*	**P**(γ)	B2VD82	YP_001906720.1	399	BLUF+ EAL	847
249	*Escherichia coli*	**P**(γ)	E2QKT5	YP_002556088.1/NP_753514.1	429	BLUF+EAL	848
	Escherichia coli	**P**(γ)		WP_001391600.1	240	BLUF+EAL	849
	Escherichia coli	**P**(γ)		WP_001468594.1	403	BLUF+EAL	850
	Escherichia coli	**P**(γ)		WP_001361917.1	403	BLUF+EAL	851
	Escherichia coli	**P**(γ)		WP_004015500.1	403	BLUF+EAL	852
	Escherichia coli	**P**(γ)		WP_001743620.1	403	BLUF+EAL	853
	Escherichia coli	**P**(γ)		WP_001740380.1	373	BLUF+EAL	854
	Escherichia coli	**P**(γ)		WP_001718133.1	403	BLUF+EAL	855
	Escherichia coli	**P**(γ)		WP_001698260.1	403	BLUF+EAL	856
	Escherichia coli	**P**(γ)		WP_001691539.1	403	BLUF+EAL	857
	Escherichia coli	**P**(γ)		WP_001695881.1	403	BLUF+EAL	858
	Escherichia coli	**P**(γ)		WP_001687453.1	403	BLUF+EAL	859
	Escherichia coli	**P**(γ)		WP_001465306.1	373	BLUF+EAL	860
	Escherichia coli	**P**(γ)		WP_001355660.1	403	BLUF+EAL	861
	Escherichia coli	**P**(γ)		WP_001295665.1	403	BLUF+EAL	862
	Escherichia coli	**P**(γ)		WP_000958311.1	417	BLUF+EAL	863
	Escherichia coli	**P**(γ)		WP_000958300.1	421	BLUF+EAL	864
	Escherichia coli	**P**(γ)		WP_000958297.1	395	BLUF+EAL	865
	Escherichia coli	**P**(γ)		WP_000958288.1	107	Short-BLUF	866
	Escherichia coli	**P**(γ)		WP_001620330.1	403	BLUF+EAL	867
	Escherichia coli	**P**(γ)		WP_001612829.1	403	BLUF+EAL	868
	Escherichia coli	**P**(γ)		WP_001607239.1	403	BLUF+EAL	869
	Escherichia coli	**P**(γ)		WP_001602271.1	403	BLUF+EAL	870
	Escherichia coli	**P**(γ)		WP_001599720.1	403	BLUF+EAL	871
	Escherichia coli	**P**(γ)		WP_001595014.1	403	BLUF+EAL	872
	Escherichia coli	**P**(γ)		WP_001578179.1	403	BLUF+EAL	873
	Escherichia coli	**P**(γ)		WP_001569733.1	169	Short-BLUF	874
	Escherichia coli	**P**(γ)		WP_001568725.1	403	BLUF+EAL	875
	Escherichia coli	**P**(γ)		WP_001555889.1	403	BLUF+EAL	876
	Escherichia coli	**P**(γ)		WP_001554913.1	403	BLUF+EAL	877
	Escherichia coli	**P**(γ)		WP_001552806.1	403	BLUF+EAL	878
	Escherichia coli	**P**(γ)		WP_001544512.1	403	BLUF+EAL	879
	Escherichia coli	**P**(γ)		WP_001538632.1	403	BLUF+EAL	880
	Escherichia coli	**P**(γ)		WP_001536901.1	403	BLUF+EAL	881
	Escherichia coli	**P**(γ)		WP_001522901.1	403	BLUF+EAL	882
	Escherichia coli	**P**(γ)		WP_001524539.1	403	BLUF+EAL	883
	Escherichia coli	**P**(γ)		WP_001446235.1	403	BLUF+EAL	884
	Escherichia coli	**P**(γ)		WP_001445544.1	403	BLUF+EAL	885
	Escherichia coli	**P**(γ)		WP_000958292.1	154	Short-BLUF	886
	Escherichia coli	**P**(γ)		WP_001374958.1	403	BLUF+EAL	887
	Escherichia coli	**P**(γ)		WP_001370660.1	403	BLUF+EAL	888
	Escherichia coli	**P**(γ)		WP_000243125.1	429	BLUF+EAL	889
	Escherichia coli	**P**(γ)		WP_001437392.1	403	BLUF+EAL	890
	Escherichia coli	**P**(γ)		WP_001433282.1	403	BLUF+EAL	891
	Escherichia coli	**P**(γ)		WP_001429147.1	403	BLUF+EAL	892
	Escherichia coli	**P**(γ)		WP_001419806.1	403	BLUF+EAL	893
	Escherichia coli	**P**(γ)		WP_001386209.1	403	BLUF+EAL	894
	Escherichia coli	**P**(γ)		WP_001362180.1	403	BLUF+EAL	895
	Escherichia coli	**P**(γ)		WP_001366263.1	403	BLUF+EAL	896
	Escherichia coli	**P**(γ)		WP_001358810.1	403	BLUF+EAL	897

	Escherichia coli	**P**(γ)		WP_001357463.1	403	BLUF+EAL	898
	Escherichia coli	**P**(γ)		WP_001317774.1	403	BLUF+EAL	899
	Escherichia coli	**P**(γ)		WP_001312754.1	403	BLUF+EAL	900
	Escherichia coli	**P**(γ)		WP_001348052.1	403	BLUF+EAL	901
	Escherichia coli	**P**(γ)		WP_001328999.1	403	BLUF+EAL	902
	Escherichia coli	**P**(γ)		WP_001324491.1	403	BLUF+EAL	903
	Escherichia coli	**P**(γ)		WP_001328624.1	403	BLUF+EAL	904
	Escherichia coli	**P**(γ)		WP_001308583.1	403	BLUF+EAL	905
	Escherichia coli	**P**(γ)		WP_001307726.1	403	BLUF+EAL	906
	Escherichia coli	**P**(γ)		WP_001463330.1	403	BLUF+EAL	907
	Escherichia coli	**P**(γ)		WP_023158058.1	405	BLUF+EAL	908
	Escherichia coli	**P**(γ)		WP_023146755.1	403	BLUF+EAL	909
	Escherichia coli	**P**(γ)		WP_022645540.1	403	BLUF+EAL	910
	Escherichia coli	**P**(γ)		WP_023356384.1	403	BLUF+EAL	911
	Escherichia coli	**P**(γ)		WP_023307905.1	403	BLUF+EAL	912
	Escherichia coli	**P**(γ)		WP_023146272.1	250	BLUF	913
	Escherichia coli	**P**(γ)		WP_023154785.1	408	BLUF+EAL	914
	Escherichia coli	**P**(γ)		WP_021559105.1	403	BLUF+EAL	915
	Escherichia coli	**P**(γ)		WP_021577349.1	403	BLUF+EAL	916
	Escherichia coli	**P**(γ)		WP_021576129.1	403	BLUF+EAL	917
	Escherichia coli	**P**(γ)		WP_021573430.1	403	BLUF+EAL	918
	Escherichia coli	**P**(γ)		WP_021565763.1	403	BLUF+EAL	919
	Escherichia coli	**P**(γ)		WP_021565021.1	403	BLUF+EAL	920
	Escherichia coli	**P**(γ)		WP_021556714.1	403	BLUF+EAL	921
	Escherichia coli	**P**(γ)		WP_021555271.1	403	BLUF+EAL	922
	Escherichia coli	**P**(γ)		WP_021545114.1	425	BLUF+EAL	923
	Escherichia coli	**P**(γ)		WP_021535563.1	342	BLUF+EAL	924
	Escherichia coli	**P**(γ)		WP_021529521.1	403	BLUF+EAL	925
	Escherichia coli	**P**(γ)		WP_021527398.1	400	BLUF+EAL	926
	Escherichia coli	**P**(γ)		WP_021524037.1	403	BLUF+EAL	927
	Escherichia coli	**P**(γ)		WP_020232864.1	403	BLUF+EAL	928
	Escherichia coli	**P**(γ)		WP_020232494.1	412	BLUF+EAL	929
	Escherichia coli	**P**(γ)		WP_020231333.1	403	BLUF+EAL	930
	Escherichia coli	**P**(γ)		WP_016236982.1	210	Short-BLUF	931
	Escherichia coli	**P**(γ)		WP_016238372.1	403	BLUF+EAL	932
	Escherichia coli	**P**(γ)		WP_001661214.1	403	BLUF+EAL	933
	Escherichia coli	**P**(γ)		WP_001658104.1	403	BLUF+EAL	934
	Escherichia coli	**P**(γ)		WP_001654201.1	403	BLUF+EAL	935
	Escherichia coli	**P**(γ)		WP_001637794.1	403	BLUF+EAL	936
	Escherichia coli	**P**(γ)		WP_001596413.1	403	BLUF+EAL	937
	Escherichia coli	**P**(γ)		WP_001500094.1	286	Short-BLUF	938
	Escherichia coli	**P**(γ)		WP_001498311.1	138	Short-BLUF	939
	Escherichia coli	**P**(γ)		WP_001413030.1	403	BLUF+EAL	940
	Escherichia coli	**P**(γ)		WP_000958306.1	403	BLUF+EAL	941
	Escherichia coli (protein YCGF)	**P**(γ)	Q2LD83	NP_415681.1	403	BLUF+ EAL	942
	Escherichia coli (strain 'clone D i14')	**P**(γ)	G7RPK6	YP_006153556.1/NP_753514.1	429	BLUF+EAL	943
	Escherichia coli (strain 'clone D i2')	**P**(γ)	G7R214	YP_006148637.1/NP_753514.1	429	BLUF+EAL	944
	Escherichia coli (strain ATCC 55124 / KO11)	**P**(γ)	E8YDG2	YP_005278355.1/NP_415681.1	403	BLUF+EAL	945
	Escherichia coli (strain ATCC 9637)	**P**(γ)	E0IV99	YP_006123985.1/NP_415681.1	403	BLUF+EAL	946
	Escherichia coli (strain K12)	**P**(γ)	P75990	NP_415681.1	403	BLUF+EAL	947

	Escherichia coli (strain UM146)	P(γ)	E1RSI8	YP_006111136.1/NP_415681.1	403	BLUF+EAL	948
	Escherichia coli (strain UTI89 / UPEC)	P(γ)	Q1RCT6	YP_540359.1	429	BLUF+ EAL	949
	Escherichia coli 042	P(γ)		YP_006095522.1	429	BLUF+EAL	950
	Escherichia coli 178850	P(γ)	N4NL03	WP_001373149.1	258	Short-BLUF	951
	Escherichia coli 179100	P(γ)	N3IGZ9	WP_001504199.1	371	BLUF+EAL	952
	Escherichia coli 1827-70	P(γ)	E2WUJ4	WP_000958293.1	203	Short-BLUF	953
	Escherichia coli 2362-75	P(γ)	E3XMI4	WP_001339576.1	372	BLUF+EAL	954
	Escherichia coli 2534-86	P(γ)	G1Z313	YP_008564297.1/NP_415681.1	403	BLUF+EAL	955
	Escherichia coli 2722950	P(γ)	N2FEM7	WP_001737956.1	403	BLUF+EAL	956
	Escherichia coli 2747800	P(γ)	M9CTP4	EMW77468.1/WP_001620704.1	373	BLUF+EAL	957
	Escherichia coli 2756500	P(γ)	M9CF46	WP_001729927.1	403	BLUF+EAL	958
	Escherichia coli 2848050	P(γ)	M8ZHS3	WP_001716462.1	403	BLUF+EAL	959
	Escherichia coli 3003	P(γ)	I2YWE3	EII85154.1/YP_002328824.1	403	BLUF+EAL	960
	Escherichia coli 3030-1	P(γ)	G1ZHP6	EGW89164.1/NP_415681.1	403	BLUF+EAL	961
	Escherichia coli 3431	P(γ)	E6B8L1	NP_415681.1	403	BLUF+EAL	962
	Escherichia coli 536	P(γ)		YP_669112.1	403	BLUF+EAL	963
	Escherichia coli 55989	P(γ)		YP_002402341.1	403	BLUF+EAL	964
	Escherichia coli 55989	P(γ)	B7LGR8	NP_415681.1	403	BLUF+ EAL	965
	Escherichia coli 83972	P(γ)	C2DS59	NP_415681.1	403	BLUF+ EAL	966
	Escherichia coli AA86	P(γ)	F5MCT4	NP_415681.1	403	BLUF+EAL	967
	Escherichia coli AI27	P(γ)	I1BDH2	EIE56793.1/WP_000243121.1	429	BLUF+EAL	968
	Escherichia coli ARS4.2123	P(γ)	K3IWY5	EKI29918.1/YP_002328824.1	403	BLUF+EAL	969
	Escherichia coli ATCC 33849 / DSM 4235	P(γ)	C9QYF1	NP_415681.1	403	BLUF+ EAL	970
	Escherichia coli ATCC 8739 / DSM 1576	P(γ)	B1IUC7	NP_415681.1	403	BLUF+ EAL	971
	Escherichia coli B / BL21	P(γ)	C5W442	NP_415681.1	403	BLUF+ EAL	972
	Escherichia coli B str. REL606	P(γ)	C6UG31	NP_415681.1	403	BLUF+ EAL	973
	Escherichia coli B088	P(γ)	D6HVN5	NP_415681.1	403	BLUF+ EAL	974
	Escherichia coli B093	P(γ)	H1DQ46	EHO01026.1/WP_001330601.1	403	BLUF+EAL	975
	Escherichia coli B171	P(γ)	B3WRP4	NP_415681.1	403	BLUF+ EAL	976
	Escherichia coli B185	P(γ)	D6I668	NP_415681.1	403	BLUF+ EAL	977
	Escherichia coli B354	P(γ)	D6J9C2	NP_415681.1	403	BLUF+ EAL	978
	Escherichia coli B7A	P(γ)	B3HJB2	NP_415681.1	403	BLUF+ EAL	979
	Escherichia coli BL21(DE3)	P(γ)	C6EGV1	NP_415681.1	403	BLUF+ EAL	980
	Escherichia coli BW2952	P(γ)	C4ZS80	NP_415681.1	403	BLUF+ EAL	981
	Escherichia coli cloneA_i1	P(γ)	G5KLP3	NP_415681.1	403	BLUF+EAL	982
	Escherichia coli DEC10A	P(γ)	H5DHS1	NP_415681.1	403	BLUF+EAL	983
	Escherichia coli DEC10B	P(γ)	H5E006	NP_415681.1	403	BLUF+EAL	984
	Escherichia coli DEC10C	P(γ)	H5EGS7	NP_415681.1	403	BLUF+EAL	985
	Escherichia coli DEC10D	P(γ)	H5EXJ1	WP_001422010.1	261	Short-BLUF	986
	Escherichia coli DEC10E	P(γ)	H5FCY8	NP_415681.1	403	BLUF+EAL	987
	Escherichia coli DEC11A	P(γ)	H5GAH5	NP_415681.1	403	BLUF+EAL	988
	Escherichia coli DEC11B	P(γ)	H5GRQ2	NP_415681.1	403	BLUF+EAL	989
	Escherichia coli DEC11C	P(γ)	H5H5Q9	NP_415681.1	403	BLUF+EAL	990
	Escherichia coli DEC11D	P(γ)	H5HNB4	NP_415681.1	403	BLUF+EAL	991
	Escherichia coli DEC11E	P(γ)	H5I2D6	NP_415681.1	403	BLUF+EAL	992
	Escherichia coli DEC12A	P(γ)	H5IHF5	NP_415681.1	403	BLUF+EAL	993
	Escherichia coli DEC12B	P(γ)	H5IZB9	NP_415681.1	403	Short-BLUF	994
	Escherichia coli DEC12C	P(γ)	H5JGE4	NP_415681.1	403	BLUF+EAL	995
	Escherichia coli DEC12D	P(γ)	H5JXD2	NP_415681.1	403	BLUF+EAL	996
	Escherichia coli DEC12E	P(γ)	H5KCA3	NP_415681.1	403	BLUF+EAL	997
	Escherichia coli DEC13A	P(γ)	H5KRY6	NP_415681.1	403	BLUF+EAL	998
	Escherichia coli DEC13B	P(γ)	H5L5W5	NP_415681.1	403	BLUF+EAL	999
	Escherichia coli DEC13C	P(γ)	H5LK42	NP_415681.1	403	BLUF+EAL	1000

	Escherichia coli DEC13D	P(γ)	H5LZX0	NP_415681.1	403	BLUF+EAL	1001
	Escherichia coli DEC13E	P(γ)	H5MDZ1	WP_001339576.1	381	BLUF+EAL	1002
	Escherichia coli DEC14A	P(γ)	H5MTG4	NP_415681.1	403	BLUF+EAL	1003
	Escherichia coli DEC14B	P(γ)	H5N8E5	NP_415681.1	403	BLUF+EAL	1004
	Escherichia coli DEC14C	P(γ)	H5NN30	WP_001379733.1	372	BLUF+EAL	1005
	Escherichia coli DEC14D	P(γ)	H5P346	NP_415681.1	403	BLUF+EAL	1006
	Escherichia coli DEC15A	P(γ)	H5PIC2	NP_415681.1	403	BLUF+EAL	1007
	Escherichia coli DEC15B	P(γ)	H5PXP2	NP_415681.1	403	BLUF+EAL	1008
	Escherichia coli DEC15C	P(γ)	H5QCW6	WP_001339576.1	372	BLUF+EAL	1009
	Escherichia coli DEC15D	P(γ)	H5QSM1	NP_415681.1	403	BLUF+EAL	1010
	Escherichia coli DEC15E	P(γ)	H5R7T0	NP_415681.1	403	BLUF+EAL	1011
	Escherichia coli DEC1A	P(γ)	H4HV06	WP_001339576.1	372	BLUF+EAL	1012
	Escherichia coli DEC1B	P(γ)	H4I9V2	NP_415681.1	403	BLUF+EAL	1013
	Escherichia coli DEC1C	P(γ)	H4IQ81	NP_415681.1	403	BLUF+EAL	1014
	Escherichia coli DEC1D	P(γ)	H4J754	NP_415681.1	403	BLUF+EAL	1015
	Escherichia coli DEC1E	P(γ)	H4JKR0	WP_001339576.1	372	BLUF+EAL	1016
	Escherichia coli DEC2A	P(γ)	H4K1Y1	WP_001410290.1	261	Short-BLUF	1017
	Escherichia coli DEC2B	P(γ)	H3KN36	NP_415681.1	403	BLUF+EAL	1018
	Escherichia coli DEC2C	P(γ)	H4KF73	WP_001410290.1	261	Short-BLUF	1019
	Escherichia coli DEC2D	P(γ)	H4KZ77	NP_415681.1	403	BLUF+EAL	1020
	Escherichia coli DEC2E	P(γ)	H4LAN7	NP_415681.1	403	BLUF+EAL	1021
	Escherichia coli DEC3F	P(γ)	H4P354	EHU91294.1/YP_006158540.1	403	BLUF+EAL	1022
	Escherichia coli DEC5A	P(γ)	H4S987	WP_001417812.1	44	Short-BLUF	1023
	Escherichia coli DEC5B	P(γ)	H4SPW0	WP_001418500.1	75	Short-BLUF	1024
	Escherichia coli DEC5C	P(γ)	H4T6P3	EHV41042.1/YP_006158540.1	403	BLUF+EAL	1025
	Escherichia coli DEC5D	P(γ)	H4TM31	WP_001419416.1	372	BLUF+EAL	1026
	Escherichia coli DEC5E	P(γ)	H4U0V1	NP_415681.1	403	BLUF+EAL	1027
	Escherichia coli DEC6A	P(γ)	H4UHN0	NP_415681.1	403	BLUF+EAL	1028
	Escherichia coli DEC6B	P(γ)	H4UYZ3	NP_415681.1	403	BLUF+EAL	1029
	Escherichia coli DEC6C	P(γ)	H4VEK7	WP_001422010.1	261	Short-BLUF	1030
	Escherichia coli DEC6D	P(γ)	H4VUU4	NP_415681.1	403	BLUF+EAL	1031
	Escherichia coli DEC6E	P(γ)	H4W991	NP_415681.1	403	BLUF+EAL	1032
	Escherichia coli DEC7A	P(γ)	H4WPY9	WP_001423841.1	386	BLUF+EAL	1033
	Escherichia coli DEC7B	P(γ)	H4X4A0	NP_415681.1	403	BLUF+EAL	1034
	Escherichia coli DEC7C	P(γ)	H4XJ12	NP_415681.1	403	BLUF+EAL	1035
	Escherichia coli DEC7D	P(γ)	H4XZW2	NP_415681.1	403	BLUF+EAL	1036
	Escherichia coli DEC7E	P(γ)	H4YEQ8	WP_001425462.1	372	BLUF+EAL	1037
	Escherichia coli DEC8A	P(γ)	H4YUP5	WP_001425462.1	372	BLUF+EAL	1038
	Escherichia coli DEC8B	P(γ)	H4ZC69	WP_001425462.1	372	BLUF+EAL	1039
	Escherichia coli DEC8C	P(γ)	H4ZUJ6	NP_415681.1	403	BLUF+EAL	1040
	Escherichia coli DEC8D	P(γ)	H5ABU3	NP_415681.1	403	BLUF+EAL	1041
	Escherichia coli DEC8E	P(γ)	H5ASV4	NP_415681.1	403	BLUF+EAL	1042
	Escherichia coli DEC9A	P(γ)	H5B8B5	NP_415681.1	403	BLUF+EAL	1043
	Escherichia coli DEC9B	P(γ)	H5BP36	NP_415681.1	403	BLUF+EAL	1044
	Escherichia coli DEC9C	P(γ)	H5C4S5	WP_001425462.1	372	BLUF+EAL	1045
	Escherichia coli DEC9D	P(γ)	H5CJX7	NP_415681.1	403	BLUF+EAL	1046
	Escherichia coli DEC9E	P(γ)	H5D173	NP_415681.1	403	BLUF+EAL	1047
	Escherichia coli DH10B	P(γ)	B1XA50	NP_415681.1	403	BLUF+ EAL	1048
	Escherichia coli E101	P(γ)	H1E3B0	NP_415681.1	403	BLUF+EAL	1049
	Escherichia coli E110019	P(γ)	B3ISH0	WP_001299924.1	403	BLUF+ EAL	1050
	Escherichia coli E1167	P(γ)	E9W5A9	NP_415681.1	403	BLUF+EAL	1051
	Escherichia coli E128010	P(γ)	E7HSJ0	WP_000958298.1	417	BLUF+EAL	1052
			E7HNP6	NP_415681.1	403	BLUF+EAL	1053

	Escherichia coli E1520	P(γ)	E9WDJ1	WP_000958294.1	197	Short-BLUF	1054
	Escherichia coli E22	P(γ)	B3ICE9	NP_415681.1	403	BLUF+ EAL	1055
	Escherichia coli E24377A	P(γ)	A7ZKT0	NP_415681.1	403	BLUF+ EAL	1056
	Escherichia coli E482	P(γ)	E9WRS0	NP_415681.1	403	BLUF+EAL	1057
	Escherichia coli EC4100B	P(γ)	E7UMM1	NP_415681.1	403	BLUF+EAL	1058
	Escherichia coli ED1a	P(γ)	B7MTT9	NP_415681.1	403	BLUF+ EAL	1059
	Escherichia coli EPECa14	P(γ)	E7HK90	NP_415681.1	403	BLUF+EAL	1060
	Escherichia coli F11	P(γ)	B3HNR5	NP_415681.1	403	BLUF+ EAL	1061
	Escherichia coli FVEC1302	P(γ)	D7JMN5	NP_415681.1	403	BLUF+ EAL	1062
	Escherichia coli FVEC1412	P(γ)	D6IP27	NP_415681.1	403	BLUF+ EAL	1063
	Escherichia coli G58-1	P(γ)	G2B830	WP_001379733.1	372	BLUF+EAL	1064
	Escherichia coli H252	P(γ)	E9VF91	NP_415681.1	403	BLUF+EAL	1065
	Escherichia coli H263	P(γ)	E9VWR7	NP_415681.1	403	BLUF+EAL	1066
	Escherichia coli H299	P(γ)	F4VTS9	NP_415681.1	403	BLUF+EAL	1067
	Escherichia coli H397	P(γ)	H1EL63	NP_415681.1	403	BLUF+EAL	1068
	Escherichia coli H489	P(γ)	E9XXX0	YP_002998973.1	403	BLUF+EAL	1069
	Escherichia coli H494	P(γ)	H1F5D1	NP_415681.1	403	BLUF+EAL	1070
	Escherichia coli H591	P(γ)	F4VDH0	NP_415681.1	403	BLUF+EAL	1071
	Escherichia coli H736	P(γ)	F4SGE2	NP_415681.1	403	BLUF+EAL	1072
	Escherichia coli HVH 24 (4-5985145)	P(γ)	T5U3L7	EQN85704.1/YP_002328824.1	403	BLUF+EAL	1073
	Escherichia coli HVH 87 (4-5977630)	P(γ)	T6LQJ1	WP_021526772.1	403	BLUF+EAL	1074
	Escherichia coli IAI1	P(γ)	B7LX75	NP_415681.1	403	BLUF+ EAL	1075
	Escherichia coli IAI39	P(γ)	B7NJI1	YP_001744038.1	403	BLUF+ EAL	1076
	Escherichia coli J53	P(γ)	I0ZQT4	EIE36605.1/YP_002292481.1	429	BLUF+EAL	1077
	Escherichia coli JJ1886	P(γ)		YP_008721327.1	403	BLUF+EAL	1078
	Escherichia coli KTE104	P(γ)	L3B881	ELI09680.1/WP_001535319.1	403	BLUF+EAL	1079
	Escherichia coli KTE143	P(γ)	L3VHA7	ELF21054.1/WP_001330601.1	403	BLUF+EAL	1080
	Escherichia coli KTE147	P(γ)	L4J3Y8	WP_001469311.1	403	BLUF+EAL	1081
	Escherichia coli KTE189	P(γ)	L4U2B8	WP_001535319.1	403	BLUF+EAL	1082
	Escherichia coli KTE24	P(γ)	S0WSF1	WP_016234228.1	203	Short-BLUF	1083
	Escherichia coli KTE36	P(γ)	S0Y267	WP_001330601.1/EOU92452.1	403	BLUF+EAL	1084
	Escherichia coli KTE37	P(γ)	S0Y3B4	WP_001330601.1/EOU92469.1	403	BLUF+EAL	1085
	Escherichia coli KTE38	P(γ)	S0Z706	WP_001330601.1/EOV06473.1	403	BLUF+EAL	1086
	Escherichia coli KTE69	P(γ)	S1E0R2	WP_001330601.1/EOV65043.1	403	BLUF+EAL	1087
	Escherichia coli KTE70	P(γ)	S1EQY3	WP_001330601.1/EOV73843.1	403	BLUF+EAL	1088
	Escherichia coli KTE74	P(γ)	S1GJK9	WP_001330601.1/EOV95898.1	403	BLUF+EAL	1089
	Escherichia coli KTE75	P(γ)	L3QAD1	WP_001563125.1	403	BLUF+EAL	1090
	Escherichia coli KTE98	P(γ)	S1GV17	EOV99135.1/WP_001330601.1	403	BLUF+EAL	1091
	Escherichia coli M605	P(γ)	F4SXD2	NP_415681.1	403	BLUF+EAL	1092
	Escherichia coli M718	P(γ)	F4TD47	NP_415681.1	403	BLUF+EAL	1093
	Escherichia coli M863	P(γ)	E9YT62	NP_415681.1	403	BLUF+EAL	1094
	Escherichia coli MS 107-1	P(γ)	D8ELF2	NP_415681.1	403	BLUF+EAL	1095
	Escherichia coli MS 110-3	P(γ)	E5ZQR8	NP_415681.1	403	BLUF+EAL	1096
	Escherichia coli MS 115-1	P(γ)	D7Y2E5	NP_415681.1	403	BLUF+EAL	1097
	Escherichia coli MS 116-1	P(γ)	D8AP06	NP_415681.1	403	BLUF+EAL	1098
	Escherichia coli MS 117-3	P(γ)	E9TJ35	NP_415681.1	403	BLUF+EAL	1099
	Escherichia coli MS 119-7	P(γ)	D8E362	NP_415681.1	403	BLUF+EAL	1100
	Escherichia coli MS 124-1	P(γ)	E1JCK6	NP_415681.1	403	BLUF+EAL	1101
	Escherichia coli MS 145-7	P(γ)	E1IU70	NP_415681.1	403	BLUF+EAL	1102
	Escherichia coli MS 146-1	P(γ)	E1HU81	WP_000958299.1	184	Short-BLUF	1103
	Escherichia coli MS 153-1	P(γ)	E6AF94	NP_415681.1	403	BLUF+EAL	1104
	Escherichia coli MS 16-3	P(γ)	E6AUN3	NP_415681.1	403	BLUF+EAL	1105
	Escherichia coli MS 175-1	P(γ)	D8B0N6	NP_415681.1	403	BLUF+EAL	1106

	Escherichia coli MS 182-1	**P**(γ)	D7YEE0	NP_415681.1	403	BLUF+EAL	1107
	Escherichia coli MS 185-1	**P**(γ)	D8C9B8	NP_415681.1	403	BLUF+EAL	1108
	Escherichia coli MS 187-1	**P**(γ)	D7ZPK1	NP_415681.1	403	BLUF+EAL	1109
	Escherichia coli MS 196-1	**P**(γ)	D8C5N7	NP_415681.1	403	BLUF+EAL	1110
	Escherichia coli MS 198-1	**P**(γ)	D7X3D5	NP_415681.1	403	BLUF+EAL	1111
	Escherichia coli MS 200-1	**P**(γ)	D8BP86	NP_415681.1	403	BLUF+EAL	1112
	Escherichia coli MS 21-1	**P**(γ)	D8AF51	WP_001330601.1	403	BLUF+EAL	1113
	Escherichia coli MS 45-1	**P**(γ)	D7YSY1	NP_415681.1	403	BLUF+EAL	1114
	Escherichia coli MS 57-2	**P**(γ)	E9UH50	NP_415681.1	403	BLUF+EAL	1115
	Escherichia coli MS 60-1	**P**(γ)	E9TPH6	NP_415681.1	403	BLUF+EAL	1116
	Escherichia coli MS 69-1	**P**(γ)	D7ZI44	NP_415681.1	403	BLUF+EAL	1117
	Escherichia coli MS 78-1	**P**(γ)	E1I486	WP_001333133.1	381	BLUF+EAL	1118
	Escherichia coli MS 79-10	**P**(γ)	F8XHY8	NP_415681.1	403	BLUF+EAL	1119
	Escherichia coli MS 84-1	**P**(γ)	D7XUY0	NP_415681.1	403	BLUF+EAL	1120
	Escherichia coli MS 85-1	**P**(γ)	E6BLL3	NP_415681.1	403	BLUF+EAL	1121
	Escherichia coli NC101	**P**(γ)	E0QZI5	NP_415681.1	403	BLUF+EAL	1122
	Escherichia coli O1:K1 / APEC	**P**(γ)	A1AA89	NP_415681.1	403	BLUF+ EAL	1123
	Escherichia coli O103:H2 (strain 12009 / EHEC)	**P**(γ)	C8U6S6	NP_415681.1	403	BLUF+ EAL	1124
	Escherichia coli O104:H4 str. 01-09591	**P**(γ)	F9CGF7	NP_415681.1	403	BLUF+EAL	1125
	Escherichia coli O104:H4 str. 04-8351	**P**(γ)	G5U6U7	NP_415681.1	403	BLUF+EAL	1126
	Escherichia coli O104:H4 str. 09-7901	**P**(γ)	G5TW99	NP_415681.1	403	BLUF+EAL	1127
	Escherichia coli O104:H4 str. 11-3677	**P**(γ)	G5UQE0	NP_415681.1	403	BLUF+EAL	1128
	Escherichia coli O104:H4 str. 11-4404	**P**(γ)	G5VMA5	NP_415681.1	403	BLUF+EAL	1129
	Escherichia coli O104:H4 str. 11-4522	**P**(γ)	G5W1R3	NP_415681.1	403	BLUF+EAL	1130
	Escherichia coli O104:H4 str. 11-4623	**P**(γ)	G5W4F4	NP_415681.1	403	BLUF+EAL	1131
	Escherichia coli O104:H4 str. 11-4632 C1	**P**(γ)	G5WRS9	NP_415681.1	403	BLUF+EAL	1132
	Escherichia coli O104:H4 str. 11-4632 C2	**P**(γ)	G5X6D7	NP_415681.1	403	BLUF+EAL	1133
	Escherichia coli O104:H4 str. 11-4632 C3	**P**(γ)	G5XQC8	NP_415681.1	403	BLUF+EAL	1134
	Escherichia coli O104:H4 str. 11-4632 C4	**P**(γ)	G5XVF0	NP_415681.1	403	BLUF+EAL	1135
	Escherichia coli O104:H4 str. 11-4632 C5	**P**(γ)	G5YFA6	NP_415681.1	403	BLUF+EAL	1136
	Escherichia coli O104:H4 str. C227-11	**P**(γ)	F9HVJ8	NP_415681.1	403	BLUF+EAL	1137
	Escherichia coli O104:H4 str. C236-11	**P**(γ)	G5TEW1	NP_415681.1	403	BLUF+EAL	1138
	Escherichia coli O104:H4 str. LB226692	**P**(γ)	F8YEL3	WP_001386733.1	372	BLUF+EAL	1139
	Escherichia coli O111:H- (strain 11128 / EHEC)	**P**(γ)	C8UNS0	YP_003233971.1	403	BLUF+ EAL	1140
	Escherichia coli O127:H6 (strain E2348/69 / EPEC)	**P**(γ)	B7UQ48	CAS08828.1/YP_002328824.1	403	BLUF+ EAL	1141
	Escherichia coli O150:H5 (strain SE15)	**P**(γ)	D2NHM0	NP_753514.1	429	BLUF+ EAL	1142
	Escherichia coli O157:H- str. 493-89	**P**(γ)	E8HST2	EFX12369.1/YP_006158540.1	403	BLUF+EAL	1143
	Escherichia coli O157:H- str. H 2687	**P**(γ)	E8I6Q4	EFX16781.1/YP_006158540.1	403	BLUF+EAL	1144
	Escherichia coli O157:H43 str. T22	**P**(γ)	H4FLQ5	NP_415681.1	403	BLUF+EAL	1145
	Escherichia coli O157:H7 str. G5101	**P**(γ)	E8HDS1	NP_415681.1	403	BLUF+EAL	1146
	Escherichia coli O157:H7 str. LSU-61	**P**(γ)	E8JDA4	EFX31706.1/YP_006158540.1	403	BLUF+EAL	1147
	Escherichia coli O17:K52:H18 (strain UMN026 / ExPEC)	**P**(γ)	B7N3W4	NP_415681.1	403	BLUF+ EAL	1148
	Escherichia coli O18:K1:H7 (strain IHE3034 / ExPEC)	**P**(γ)	D5D0X4	NP_415681.1	403	BLUF+ EAL	1149
	Escherichia coli O25b:H4-ST131 str. EC958	**P**(γ)	N1N5N1	WP_001295983.1	146	Short-BLUF	1150
	Escherichia coli O26:H11 (strain 11368 / EHEC)	**P**(γ)	C8TQH3	NP_415681.1	403	BLUF+ EAL	1151
	Escherichia coli O44:H18 (strain 042 / EAEC)	**P**(γ)	D3H1V7	NP_753514.1	429	BLUF+ EAL	1152
	Escherichia coli O55:H7 str. 3256-97	**P**(γ)	E8IK46	WP_000958309.1	106	Short-BLUF	1153
	Escherichia coli O55:H7 str. RM12579	**P**(γ)	H6MEI0	AEZ39985.1/YP_006158540.1	403	BLUF+EAL	1154
	Escherichia coli O55:H7 str. USDA 5905	**P**(γ)	E8IY83	EFX26987.1/YP_006158540.1	403	BLUF+EAL	1155
	Escherichia coli O6	**P**(γ)	Q8FI42	NP_753514.1	429	BLUF+ EAL	1156
	Escherichia coli O6:K15:H31 536 / UPEC)	**P**(γ)	Q0TIL8	NP_415681.1	403	BLUF+ EAL	1157
	Escherichia coli O83:H1 (strain NRG 857C / AIEC)	**P**(γ)	E4P2E2	ADR26615.1/YP_006119549.1	403	BLUF+EAL	1158
	Escherichia coli O9:H4 / HS	**P**(γ)	A7ZZ94	NP_415681.1	403	BLUF+ EAL	1159

	Escherichia coli OK1180	P(γ)	E7IPF0	NP_415681.1	403	BLUF+EAL	1160
	Escherichia coli OK1357	P(γ)	E7J2J6	NP_415681.1	403	BLUF+EAL	1161
	Escherichia coli OR:K5:H- (strain ABU 83972)	P(γ)	E1PJK6	NP_753514.1	429	BLUF+EAL	1162
	Escherichia coli P0301867.11	P(γ)	N3F7P9	WP_001484419.1	403	BLUF+EAL	1163
	Escherichia coli P0304816.15	P(γ)	N4BKD7	ENF50218.1/WP_001620704.1	373	BLUF+EAL	1164
	Escherichia coli P0305260.5	P(γ)	N4GMJ1	WP_001648704.1	403	BLUF+EAL	1165
	Escherichia coli PCN033	P(γ)	F7MVM7	NP_415681.1	403	BLUF+EAL	1166
	Escherichia coli RN587/1	P(γ)	E7JQF9	EFZ72697.1/YP_002328824.1	403	BLUF+EAL	1167
	Escherichia coli S88	P(γ)	B7MK58	NP_415681.1	403	BLUF+ EAL	1168
	Escherichia coli SCI-07	P(γ)	H8D7Y9	NP_415681.1	403	BLUF+EAL	1169
	Escherichia coli SE11	P(γ)	B6I9L9	BAG76730.1/YP_002292481.1	429	BLUF+ EAL	1170
	Escherichia coli SE15	P(γ)		YP_003349094.1	429	BLUF+EAL	1171
	Escherichia coli SMS-3-5 / SECEC	P(γ)	B1LI04	YP_001744038.1	403	BLUF+ EAL	1172
	Escherichia coli STEC_7v	P(γ)	F3LXX7	WP_001360582.1	372	BLUF+EAL	1173
	Escherichia coli STEC_94C	P(γ)	G1ZY43	WP_001378868.1	261	Short-BLUF	1174
	Escherichia coli STEC_B2F1	P(γ)	G1Y830	WP_000958302.1	371	BLUF+EAL	1175
	Escherichia coli STEC_C165-02	P(γ)	G1YNH8	WP_001376825.1	372	BLUF+EAL	1176
	Escherichia coli STEC_DG131-3	P(γ)	G2AEF4	WP_001379733.1	372	BLUF+EAL	1177
	Escherichia coli STEC_EH250	P(γ)	G2ATD7	WP_001379733.1	372	BLUF+EAL	1178
	Escherichia coli STEC_H.1.8	P(γ)	G2BMD5	WP_001382007.1	230	Short-BLUF	1179
	Escherichia coli STEC_MHI813	P(γ)	G2C2Z3	WP_001383099.1	372	BLUF+EAL	1180
	Escherichia coli STEC_S1191	P(γ)	G2CJ51	NP_415681.1	403	BLUF+EAL	1181
	Escherichia coli TA124	P(γ)	H1FGR3	WP_001389598.1	403	BLUF+EAL	1182
	Escherichia coli TA143	P(γ)	F4U625	NP_415681.1	403	BLUF+EAL	1183
	Escherichia coli TA206	P(γ)	F4TT04	WP_000958310.1	176	Short-BLUF	1184
	Escherichia coli TA271	P(γ)	F4ULK8	NP_415681.1	403	BLUF+EAL	1185
	Escherichia coli TW10509	P(γ)	E9XMH9	WP_000958307.1	403	BLUF+EAL	1186
	Escherichia coli TX1999	P(γ)	G2CYS1	NP_415681.1	403	BLUF+EAL	1187
	Escherichia coli UMN026	P(γ)		YP_002412195.1	403	BLUF+EAL	1188
	Escherichia coli UMNF18	P(γ)	G0FBW4	NP_415681.1	403	BLUF+EAL	1189
	Escherichia coli UMNK88	P(γ)	F4M2X4	NP_415681.1	403	BLUF+EAL	1190
	Escherichia coli WV_060327	P(γ)	E7U6E5	NP_415681.1	403	BLUF+EAL	1191
	Escherichia coli XH001	P(γ)	G2F1Z8	NP_415681.1	403	BLUF+EAL	1192
	Escherichia coli XH140A	P(γ)	F9QYE5	NP_415681.1		BLUF+EAL	1193
250	*Escherichia hermannii NBRC 105704*	P(γ)	H5V3N0	WP_002436603.1		BLUF+EAL	1194
			H5UZV4	WP_002434328.1	406	BLUF+EAL	1195
251	*Escherichia sp. 3_2_53FAA*	P(γ)	C1HJN0	NP_753514.1	429	BLUF+ EAL	1196
252	*Frateuria aurantia ATCC 33424*	P(γ)	H8L2L8	YP_005377167.1	424	BLUF+EAL	1197
			H8L2L7	YP_005377166.1	439	BLUF+EAL	1198
253	*gamma proteobacterium BDW918*	P(γ)	I2JPC2	WP_008246638.1	138	Short-BLUF	1199
254	*gamma proteobacterium HIMB55*	P(γ)U	H3NRI2	WP_009470377.1	146	Short-BLUF	1200
255	*gamma proteobacterium IMCC1989*	P(γ)	F3LF24	WP_009669485.1	144	Short-BLUF	1201
256	*gamma proteobacterium IMCC2047*	P(γ)U	F3KDB1	WP_008530288.1	150	Short-BLUF	1202
257	*gamma proteobacterium IMCC2047*	P(γ)Unc		EGG99643.1	150	Short-BLUF	1203
258	*gamma proteobacterium IMCC3088*	P(γ)U	F3KYX3	WP_009574606.1	145	Short-BLUF	1204
259	*gamma proteobacterium NOR5-3*	P(γ)U	B8KEY4	WP_009021778.1	161	Short-BLUF	1205
260	*gamma proteobacterium NOR5-3*	P(γ)U	B8KLI1	WP_009023838.1	157	Short-BLUF	1206
261	*gamma proteobacterium NOR5-3*	P(γ)U	B8KT47	WP_009021276.1	149	Short-BLUF	1207
262	*gamma proteobacterium WG36*	P(γ)		WP_017494424.1	405	BLUF+EAL	1208
263	*Gayadomonas joobiniege*	P(γ)		WP_017445646.1	152	Short-BLUF	1209
264	*Gilvimarinus chinensis*	P(γ)		WP_020208650.1	152	Short-BLUF	1210
265	*Glaciecola arctica BSs20135*	P(γ)	K6Y178	WP_007616696.1	136	Short-BLUF	1211
			K6Y8U9	WP_007622166.1	134	Short-BLUF	1212

266	*Glaciecola mesophila KMM 241*	P(γ)	K6XXC1	WP_006993405.1	133	Short-BLUF	1213
267	*Glaciecola nitratireducens JCM 12485*	P(γ)	G4QF34	YP_004870371.1	147	Short-BLUF	1214
268	*Grimontia hollisae CIP 101886*	P(γ)	D0IB49	WP_005505863.1	136	Short-BLUF	1215
269	*Grimontia sp. AK16*	P(γ)	R1IQH4	WP_002541795.1	136	Short-BLUF	1216
270	*Halothiobacillus neapolitanus c2*	P(γ)	D0L046	YP_003263116.1	143	Short-BLUF	1217
271	*Kangiella aquimarina*	P(γ)		WP_018624129.1	143	Short-BLUF	1218
272	*Kangiella koreensis DSM 16069*	P(γ)	C7R790	YP_003147314.1	143	Short-BLUF	1219
273	*Klebsiella oxytoca*	P(γ)		WP_016810135.1	407	BLUF+EAL	1220
	Klebsiella oxytoca	P(γ)		WP_023320606.1	407	BLUF+EAL	1221
	Klebsiella oxytoca	P(γ)		WP_017145797.1	409	BLUF+EAL	1222
	Klebsiella oxytoca	P(γ)		WP_016808785.1	409	BLUF+EAL	1223
	Klebsiella oxytoca 10-5242	P(γ)	H3L520	EHT02598.1/WP_004849017.1	407	BLUF+EAL	1224
	Klebsiella oxytoca 10-5242	P(γ)	H3L8A3	EHS99926.1/WP_004851219.1	408	BLUF+EAL	1225
	Klebsiella oxytoca 10-5243	P(γ)	H3LKF5	WP_004100387.1	407	BLUF+EAL	1226
	Klebsiella oxytoca 10-5243	P(γ)	H3LPD4	WP_004102968.1	390	BLUF+EAL	1227
	Klebsiella oxytoca 10-5243	P(γ)	H3LNU7	EHS97649.1/WP_004102385.1	409	BLUF+EAL	1228
	Klebsiella oxytoca 10-5245	P(γ)	H3M2I6	WP_004111512.1	407	BLUF+EAL	1229
	Klebsiella oxytoca 10-5245	P(γ)	H3M586	EHS97119.1/WP_004113307.1	409	BLUF+EAL	1230
	Klebsiella oxytoca 10-5250	P(γ)	H3N9V3	WP_004130625.1	407	BLUF+EAL	1231
	Klebsiella oxytoca 10-5250	P(γ)	H3MWL6	EHT14305.1/WP_004119787.1	408	BLUF+EAL	1232
	Klebsiella oxytoca ATCC 8724	P(γ)	G8WFT1	YP_005018934.1	407	BLUF+EAL	1233
	Klebsiella oxytoca ATCC 8724	P(γ)	G8WGX1	AEX05998.1/YP_005020237.1	402	BLUF+EAL	1234
	Klebsiella oxytoca E718	P(γ)	I6W3I1	YP_006497531.1	407	BLUF+EAL	1235
	Klebsiella oxytoca M5al	P(γ)	K6JLZ8	WP_004134964.1	407	BLUF+EAL	1236
	Klebsiella oxytoca M5al	P(γ)	K6JK93	WP_004136285.1	405	BLUF+EAL	1237
274	*Klebsiella pneumoniae*	P(γ)	R4YC97	CCI78036.1	403	BLUF+EAL	1238
	Klebsiella pneumoniae	P(γ)	G0GUP8	YP_005953822.1	403	BLUF+EAL	1239
	Klebsiella pneumoniae	P(γ)	G0GPP1	YP_005954630.1	405	BLUF+EAL	1240
	Klebsiella pneumoniae	P(γ)		WP_017899618.1	403	BLUF+EAL	1241
	Klebsiella pneumoniae	P(γ)		WP_021314053.1	403	BLUF+EAL	1242
	Klebsiella pneumoniae	P(γ)		WP_020802923.1	405	BLUF+EAL	1243
	Klebsiella pneumoniae	P(γ)		WP_017879970.1	405	BLUF+EAL	1244
	Klebsiella pneumoniae	P(γ)		WP_021314208.1	405	BLUF+EAL	1245
	Klebsiella pneumoniae	P(γ)		WP_017900341.1	405	BLUF+EAL	1246
	Klebsiella pneumoniae	P(γ)		WP_023288895.1	403	BLUF+EAL	1247
	Klebsiella pneumoniae	P(γ)		WP_023287389.1	403	BLUF+EAL	1248
	Klebsiella pneumoniae	P(γ)		WP_023302151.1	403	BLUF+EAL	1249
	Klebsiella pneumoniae	P(γ)		WP_023283451.1	410	BLUF+EAL	1250
	Klebsiella pneumoniae	P(γ)		WP_023297241.1	403	BLUF+EAL	1251
	Klebsiella pneumoniae	P(γ)		WP_023342830.1	405	BLUF+EAL	1252
	Klebsiella pneumoniae	P(γ)		WP_023341511.1	405	BLUF+EAL	1253
	Klebsiella pneumoniae	P(γ)		WP_023327961.1	405	BLUF+EAL	1254
	Klebsiella pneumoniae	P(γ)		WP_023322569.1	405	BLUF+EAL	1255
	Klebsiella pneumoniae	P(γ)		WP_023316827.1	405	BLUF+EAL	1256
	Klebsiella pneumoniae	P(γ)		WP_023301958.1	405	BLUF+EAL	1257
	Klebsiella pneumoniae	P(γ)		WP_023286396.1	405	BLUF+EAL	1258
	Klebsiella pneumoniae	P(γ)		WP_023318182.1	405	BLUF+EAL	1259
	Klebsiella pneumoniae	P(γ)		WP_023340100.1	405	BLUF+EAL	1260
	Klebsiella pneumoniae 342	P(γ)	B5XQZ5	YP_002238639.1	405	BLUF+ EAL	1261
	Klebsiella pneumoniae 342	P(γ)	B5XZ96	ACI08361.1/YP_002239613.1	403	BLUF+ EAL	1262
	Klebsiella pneumoniae 700603	P(γ)	M7PV04	WP_004886055.1	403	BLUF+EAL	1263
	Klebsiella pneumoniae 700603	P(γ)	M7PCJ7	WP_004206010.1	405	BLUF+EAL	1264
	Klebsiella pneumoniae ATCC BAA-1705	P(γ)	M7QLZ6	EMR23689.1/YP_005226813.1	405	BLUF+EAL	1265

	Klebsiella pneumoniae ATCC BAA-2146	P(γ)	M7QLK1	EMR23559.1/YP_005226813.1	405	BLUF+EAL	1266
	Klebsiella pneumoniae CG43	P(γ)		YP_008659363.1	403	BLUF+EAL	1267
	Klebsiella pneumoniae DMC0799	P(γ)	S7BC21	EPO21704.1/YP_001334448.1	**403**	BLUF+EAL	1268
	Klebsiella pneumoniae hvKP1	P(γ)	M2AJV2	EMB10239.1/YP_001334448.1	403	BLUF+EAL	1269
	Klebsiella pneumoniae hvKP1	P(γ)	M2ATT4	EMB12948.1/YP_001335259.1	405	BLUF+EAL	1270
	Klebsiella pneumoniae JHCK1	P(γ)	M3V4M6	YP_001334448.1	**403**	BLUF+EAL	1271
	Klebsiella pneumoniae JHCK1	P(γ)	M3SU26	EMH92743.1/YP_001335259.1	405	BLUF+EAL	1272
	Klebsiella pneumoniae KP-11	P(γ)	S2CCS2	EOZ72815.1/YP_001334448.1	403	BLUF+EAL	1273
	Klebsiella pneumoniae KP-7	P(γ)	S1TBD0	EOY66353.1/YP_001334448.1	403	BLUF+EAL	1274
	Klebsiella pneumoniae NTUH-K2044	P(γ)	C4X832	YP_002919357.1	422	BLUF+ EAL	1275
	Klebsiella pneumoniae NTUH-K2044	P(γ)	C4X5Q9	YP_002918534.1	403	BLUF+ EAL	1276
	Klebsiella pneumoniae RYC492	P(γ)	M5QAQ1	WP_004893335.1	403	BLUF+EAL	1277
	Klebsiella pneumoniae RYC492	P(γ)	M5Q7X9	EMH98112.1/YP_006636433.1	405	BLUF+EAL	1278
275	*Klebsiella pneumoniae subsp. pneumoniae 1084*	P(γ)	K4HAP6	YP_006637253.1/YP_001334448.1	403	BLUF+EAL	1279
	Klebsiella pneumoniae subsp. pneumoniae 1084	P(γ)	K4H3X6	YP_006636433.1	405	BLUF+EAL	1280
	Klebsiella pneumoniae subsp. pneumoniae ATCC 700721	P(γ)	A6T8V8	ABR77029.1/YP_001335259.1	405	BLUF+ EAL	1281
	Klebsiella pneumoniae subsp. pneumoniae ATCC 700721	P(γ)	A6T6J7	ABR76218.1/YP_001334448.1	403	BLUF+ EAL	1282
	Klebsiella pneumoniae subsp. pneumoniae DSM 30104	P(γ)	J2X7Q0	EJK91023.1/YP_001335259.1	405	BLUF+EAL	1283
	Klebsiella pneumoniae subsp. pneumoniae Ecl8	P(γ)	K4UDV3	CCN29648.1/YP_001335259.1	405	BLUF+EAL	1284
	Klebsiella pneumoniae subsp. pneumoniae HS11286	P(γ)	G8W5H8	YP_005225909.1	403	BLUF+EAL	1285
	Klebsiella pneumoniae subsp. pneumoniae HS11286	P(γ)	G8W1U2	AEW61211.1/YP_005226813.1	405	BLUF+EAL	1286
	Klebsiella pneumoniae subsp. pneumoniae KpMDU1	P(γ)	N9UNX0	WP_002895176.1	103	Short-BLUF	1287
	Klebsiella pneumoniae subsp. pneumoniae KpMDU1	P(γ)	N9UB22	ENY57642.1/YP_005226813.1	405	BLUF+EAL	1288
	Klebsiella pneumoniae subsp. pneumoniae KPNIH1	P(γ)	J2LLB7	EJJ33749.1/YP_005226813.1	405	BLUF+EAL	1289
	Klebsiella pneumoniae subsp. pneumoniae KPNIH10	P(γ)	J2Q500	EJJ73872.1/YP_005226813.1	405	BLUF+EAL	1290
	Klebsiella pneumoniae subsp. pneumoniae KPNIH11	P(γ)	J1Y1B0	EJJ83951.1/YP_005226813.1	405	BLUF+EAL	1291
	Klebsiella pneumoniae subsp. pneumoniae KPNIH12	P(γ)	J2R422	EJJ85679.1/YP_005226813.1	405	BLUF+EAL	1292
	Klebsiella pneumoniae subsp. pneumoniae KPNIH14	P(γ)	J1XPZ0	EJJ86178.1/YP_005226813.1	405	BLUF+EAL	1293
	Klebsiella pneumoniae subsp. pneumoniae KPNIH16	P(γ)	J2SEY9	EJK01005.1/YP_005226813.1	405	BLUF+EAL	1294
	Klebsiella pneumoniae subsp. pneumoniae KPNIH17	P(γ)	J1ZJT8	EJK00872.1/YP_005226813.1	405	BLUF+EAL	1295
	Klebsiella pneumoniae subsp. pneumoniae KPNIH18	P(γ)	J1ZTT7	EJK09919.1/YP_005226813.1	405	BLUF+EAL	1296
	Klebsiella pneumoniae subsp. pneumoniae KPNIH19	P(γ)	J2TV30	EJK17135.1/YP_005226813.1	405	BLUF+EAL	1297
	Klebsiella pneumoniae subsp. pneumoniae KPNIH2	P(γ)	J2BEA0	EJJ34470.1/YP_005226813.1	405	BLUF+EAL	1298
	Klebsiella pneumoniae subsp. pneumoniae KPNIH20	P(γ)	J2TVY5	EJK17435.1/YP_005226813.1	405	BLUF+EAL	1299
	Klebsiella pneumoniae subsp. pneumoniae KPNIH21	P(γ)	J2JB02	EJK27376.1/YP_005226813.1	405	BLUF+EAL	1300
	Klebsiella pneumoniae subsp. pneumoniae KPNIH22	P(γ)	J2VAC5	EJK33703.1/YP_005226813.1	405	BLUF+EAL	1301
	Klebsiella pneumoniae subsp. pneumoniae KPNIH23	P(γ)	J2BVP2	EJK34244.1/YP_005226813.1	405	BLUF+EAL	1302
	Klebsiella pneumoniae subsp. pneumoniae KPNIH4	P(γ)	J2CGH8	EJJ47550.1/YP_005226813.1	405	BLUF+EAL	1303
	Klebsiella pneumoniae subsp. pneumoniae KPNIH5	P(γ)	J1V7T6	EJJ51671.1/YP_005226813.1	405	BLUF+EAL	1304
	Klebsiella pneumoniae subsp. pneumoniae KPNIH6	P(γ)	J1VSE1	EJJ58196.1/YP_005226813.1	405	BLUF+EAL	1305
	Klebsiella pneumoniae subsp. pneumoniae KPNIH7	P(γ)	J1VSD5	EJJ63833.1/YP_005226813.1	405	BLUF+EAL	1306
	Klebsiella pneumoniae subsp. pneumoniae KPNIH8	P(γ)	J1WBB9	EJJ70043.1/YP_005226813.1	405	BLUF+EAL	1307
	Klebsiella pneumoniae subsp. pneumoniae KPNIH9	P(γ)	J1WDS7	EJJ70888.1/YP_005226813.1	405	BLUF+EAL	1308
	Klebsiella pneumoniae subsp. pneumoniae KpQ3	P(γ)	M5GMC5	EKF78982.1/YP_001335259.1	405	BLUF+EAL	1309
	Klebsiella pneumoniae subsp. pneumoniae ST258-K26BO	P(γ)	K4RS57	CCM80630.1/YP_005226813.1	405	BLUF+EAL	1310
	Klebsiella pneumoniae subsp. pneumoniae ST258-K28BO	P(γ)	K4SA97	CCM86960.1/YP_005226813.1	405	BLUF+EAL	1311
	Klebsiella pneumoniae subsp. pneumoniae ST512-K30BO	P(γ)	K4SX07	CCM95184.1/YP_005226813.1	405	BLUF+EAL	1312
	Klebsiella pneumoniae subsp. pneumoniae WGLW1	P(γ)	K1NF33	EKB66739.1/YP_001334448.1	403	BLUF+EAL	1313
			K1M9Z4	EKB66010.1/YP_001335259.1	405	BLUF+EAL	1314
	Klebsiella pneumoniae subsp. pneumoniae WGLW2	P(γ)	K1NR75	EKB79227.1/YP_001334448.1	**403**	BLUF+EAL	1315
			K1N312	EKB75950.1/YP_001335259.1	405	BLUF+EAL	1316
	Klebsiella pneumoniae subsp. pneumoniae WGLW3	P(γ)	K1N1S9	YP_001334448.1/EKB75485.1	**403**	BLUF+EAL	1317
			K1NLB8	EKB77327.1/YP_001335259.1	405	BLUF+EAL	1318

	Klebsiella pneumoniae subsp. pneumoniae WGLW5	**P(γ)**	K1Q3I3	EKB86354.1/YP_001334448.1	403	BLUF+EAL	1319
			K1Q1K2	WP_004184418.1	390	BLUF+EAL	1320
			K1P7W3	EKB85222.1/YP_001335259.1	405	BLUF+EAL	1321
276	*Klebsiella pneumoniae subsp. rhinoscleromatis ATCC 13884*	**P(γ)**	C8TA32	EEW39489.1/YP_001335259.1	405	BLUF+ EAL	1322
	Klebsiella pneumoniae subsp. rhinoscleromatis ATCC 13884	**P(γ)**	C8T1B3	WP_004142148.1	405	BLUF+ EAL	1323
	Klebsiella pneumoniae UHKPC179	**P(γ)**	S7FYI3	YP_001334448.1	403	BLUF+EAL	1324
	Klebsiella pneumoniae UHKPC23	**P(γ)**	R9BEZ7	EOR15588.1/YP_005226813.1	405	BLUF+EAL	1325
	Klebsiella pneumoniae UHKPC57	**P(γ)**	S2EQN8	EPA91839.1/YP_001334448.1	**403**	BLUF+EAL	1326
	Klebsiella pneumoniae UHKPC81	**P(γ)**	S1UVJ0	EOY85408.1/YP_001334448.1	**403**	BLUF+EAL	1327
	Klebsiella pneumoniae VA360	**P(γ)**	M5SGV5	EMI36105.1/YP_005226813.1	405	BLUF+EAL	1328
277	*Klebsiella sp. 1_1_55*	**P(γ)**	D6GDL7	EFD87032.1/WP_008804990.1	405	BLUF+ EAL	1329
			D6GGM4	WP_008805714.1	403	BLUF+ EAL	1330
	Klebsiella sp. 4_1_44FAA	**P(γ)**	G9RCF4	EHL93455.1/YP_001334448.1	403	BLUF+EAL	1331
	Klebsiella sp. KTE92	**P(γ)**	R8XC78	EOQ54795.1/YP_002239613.1	**403**	BLUF+EAL	1332
	Klebsiella sp. KTE92	**P(γ)**	R8XFY6	WP_016160979.1	405	BLUF+EAL	1333
	Klebsiella sp. MS 92-3	**P(γ)**	F3Q8C8	EGF62238.1/YP_001335259.1	405	BLUF+EAL	1334
			F3PZ97	EGF65408.1/YP_001334448.1	403	BLUF+EAL	1335
	Klebsiella sp. OBRC7	**P(γ)**	J5V6R0	WP_004849017.1	**407**	BLUF+EAL	1336
278	*Klebsiella variicola (strain At-22)*	**P(γ)**	D3RGS1	YP_003439664.1	405	BLUF+ EAL	1337
			D3RM34	YP_003440514.1	403	BLUF+ EAL	1338
	Klebsiella variicola CAG:634	**P(γ)**	R5WD52	CDA00595.1/YP_002239613.1	403	BLUF+EAL	1339
			R5WE11	WP_008804990.1	405	BLUF+EAL	1340
			R5X5Z9	WP_022066376.1	390	BLUF+EAL	1341
279	*Lamprocystis purpurea*	**P(γ)**		WP_020505137.1	295	BLUF+PsiE	1342
280	*Legionella drancourtii LLAP12*	**P(γ)**	G9ETS2	WP_006872576.1	155	Short-BLUF	1343
281	*Legionella longbeachae D-4968*	**P(γ)**	D1RLZ8	WP_003636764.1	309	BLUF+PAS	1344
282	*Legionella longbeachae serogroup 1 (strain NSW150)*	**P(γ)**	D3HJ09	YP_003455474.1	708	BLUF+3PAS+GGDEF	1345
283	*Leucothrix mucor*	**P(γ)**		WP_022951283.1	139	Short-BLUF	1346
				WP_022951988.1	143	Short-BLUF	1347
284	*Marichromatium purpuratum 984*	**P(γ)**	F9TWL2	WP_005220255.1	1221	2PAS+GGDEF+EAL+BLUF	1348
			F9TYC6	WP_005221896.1	137	Short-BLUF	1349
285	*marine gamma proteobacterium HTCC2080*	**P(γ)U**	A0Z2D8	WP_007233984.1	150	Short-BLUF	1350
			A0Z4F4	WP_007234700.1	159	Short-BLUF	1351
			A0Z3A0	WP_007234296.1	152	Short-BLUF	1352
286	*marine gamma proteobacterium HTCC2148*	**P(γ)U**	B7S2E6	WP_007230530.1	127	Short-BLUF	1353
287	*Marinobacter adhaerens (strain HP15)*	**P(γ)**	E4PFT3	YP_005885931.1	143	Short-BLUF	1354
			E4PIQ9		185	Short-BLUF	1355
288	*Marinobacter algicola DG893*	**P(γ)**	A6F2F1	WP_007154440.1	188	Short-BLUF	1356
289	*Marinobacter aquaeolei VT8*	**P(γ)**	A1U4G7	YP_960073.1	189	Short-BLUF	1357
290	*Marinobacter hydrocarbonoclasticus ATCC 49840*	**P(γ)**	H8WBH9	YP_005430586.1	189	Short-BLUF	1358
291	*Marinobacter lipolyticus SM19*	**P(γ)**	R8B0A9	WP_012138058.1	151	Short-BLUF	1359
292	*Marinobacter manganoxydans MnI7-9*	**P(γ)**	G6YW66	WP_008175045.1	143	Short-BLUF	1360
			G6YUG7	WP_008174059.1	188	Short-BLUF	1361
293	*Marinomonas mediterranea ATCC 700492*	**P(γ)**	F2JZB3	YP_004315034.1	138	Short-BLUF	1362
			F2JWR3	YP_004313663.1	144	Short-BLUF	1363
294	*Marinomonas posidonica CECT 7376*	**P(γ)**	F6CVF9	YP_004482255.1	143	Short-BLUF	1364
295	*Marinomonas sp. MED121*	**P(γ)**	A3YED7	WP_009835955.1	140	Short-BLUF	1365
			A3YFN4	WP_009833224.1	152	Short-BLUF	1366
			A3YFL0	WP_009833248.1	154	Short-BLUF	1367
	Marinomonas sp. MWYL1	**P(γ)**	A6VYY7	YP_001341601.1	142	Short-BLUF	1368
296	*Methylobacter marinus*	**P(γ)**		WP_020161578.1	289	BLUF+PsiE	1369
297	*Methylobacter tundripaludum SV96*	**P(γ)**	G3ISM9	WP_006890370.1	289	Short-BLUF	1370
298	*Methylohalobius crimeensis*	**P(γ)**		WP_022950030.1	290	BLUF+PsiE	1371

299	*Methylomicrobium alcaliphilum DSM 19304*	P(γ)	G4T071	YP_004916955.1	136	Short-BLUF	1372
			G4T077	YP_004916961.1	294	Short-BLUF	1373
			G4T0S3	YP_004915950.1	141	Short-BLUF	1374
			G4T2T3	YP_004916161.1	1111	PAS+GGDEF+EAL+BLUF	1375
300	*Methylomicrobium buryatense*	P(γ)		WP_017838976.1	294	BLUF+PsiE	1376
				WP_017840127.1	1097	LeuBD+PAS+GGDEF+EAL+BLUF	1377
				WP_017840877.1	141	Short-BLUF	1378
				WP_017840373.1	141	Short-BLUF	1379
				WP_017838971.1	136	Short-BLUF	1380
301	*Methylomicrobium japanense*	P(γ)	Q25BV7	BAE86881.1	141	Short-BLUF	1381
302	*Methylomonas methanica (strain MC09)*	P(γ)	G0A610	YP_004512959.1	120	Short-BLUF	1382
			G0A1P9	YP_004512609.1	292	Short-BLUF	1383
			G0A2A9	YP_004511421.1	1104	PAS+GGDEF+EAL+BLUF	1384
303	*Methylophaga aminisulfidivorans MP*	P(γ)	F5T211	WP_007146401.1	140	Short-BLUF	1385
304	*Methylophaga lonarensis MPL*	P(γ)	M7PJN9	WP_009725326.1	159	Short-BLUF	1386
305	*Methylophaga thiooxidans DMS010*	P(γ)	C0N5Z8	WP_008291053.1	139	Short-BLUF	1387
			C0N9F9	WP_008292188.1	136	Short-BLUF	1388
306	*Neptuniibacter caesariensis*	P(γ)	Q2BIC6	WP_007020013.1	142	Short-BLUF	1389
			Q2BP57	WP_007020760.1	147	Short-BLUF	1390
307	*Nevskia ramosa*	P(γ)		WP_022977191.1	429	BLUF+SCHICH	1391
308	*Pantoea agglomerans*	P(γ)		WP_022625735.1	403	BLUF+EAL	1392
				WP_010671955.1	395	BLUF+EAL	1393
				WP_010670046.1	403	BLUF+EAL	1394
				WP_022624234.1	395	BLUF+EAL	1395
	Pantoea agglomerans 299R	P(γ)	L7BXC6	ELP25506.1/WP_003850648.1	395	BLUF+EAL	1396
			L7BW56	ELP25775.1/WP_003850263.1	403	BLUF+EAL	1397
309	*Pantoea ananatis*	P(γ)		WP_019105298.1	415	BLUF+EAL	1398
	Pantoea ananatis (strain AJ13355)	P(γ)	F2ET35	YP_005195892.1	403	BLUF+EAL	1399
			F2EZT9	YP_003522512.1	397	BLUF+EAL	1400
	Pantoea ananatis (strain LMG 20103)	P(γ)	D4GGD1	CP001875.2/YP_003522512.1	397	BLUF+ EAL	1401
			D4GDM8	YP_003520043.1	403	BLUF+ EAL	1402
	Pantoea ananatis LMG 5342	P(γ)	G9AT61	CCF09855.1/YP_005195892.1	403	BLUF+EAL	1403
			G9AXC0	YP_005197808.1	397	BLUF+EAL	1404
	Pantoea ananatis PA13	P(γ)	G7UHX4	YP_005993091.1/YP_005195892.1	403	BLUF+EAL	1405
			G7UM94	YP_005990549.1	397	BLUF+EAL	1406
310	*Pantoea dispersa EGD-AAK13*	P(γ)	U1TTN2	WP_021509874.1	403	BLUF+EAL	1407
311	*Pantoea sp. aB*	P(γ)	E0M383	WP_008926784.1	405	BLUF+EAL	1408
			E0M391	ELP25506.1/WP_003850648.1	395	BLUF+EAL	1409
			E0M088	EFM19210.1/WP_003850263.1	403	BLUF+EAL	1410
	Pantoea sp. AS-PWVM4	P(γ)	U2M401	WP_021182872.1	403	BLUF+EAL	1411
	Pantoea sp. At-9b	P(γ)	E6WL97	YP_004118738.1	386	BLUF+EAL	1412
			E6WBK2	YP_004116243.1	403	BLUF+EAL	1413
	Pantoea sp. GM01	P(γ)	J2LHM1	WP_007891430.1	403	BLUF+EAL	1414
	Pantoea sp. Sc1	P(γ)	H8DUK7	WP_009093254.1	395	BLUF+EAL	1415
			H8DLJ6	WP_009090445.1	403	BLUF+EAL	1416
			H8DUL4	WP_009093262.1	405	BLUF+EAL	1417
	Pantoea sp. SL1_M5	P(γ)		WP_010256946.1	395	BLUF+EAL	1418
				WP_010245539.1	403	BLUF+EAL	1419
	Pantoea sp. YR343	P(γ)	J2UXE8	WP_008105386.1	403	BLUF+EAL	1420
312	*Pantoea stewartii subsp. stewartii DC283*	P(γ)	H3RKF0	WP_006122006.1	134	Short-BLUF	1421
			H3RCE8	WP_006119001.1	403	BLUF+EAL	1422
313	*Pantoea vagans C9-1 (Pantoea agglomerans C9-1)*	P(γ)	E1SBR1	YP_003930829.1	403	BLUF+EAL	1423
			E1PKB3	YP_003929520.1	404	BLUF+EAL	1424

			E1PKA4	YP_003929511.1	409	BLUF+EAL	1425
314	*Pectobacterium carotovorum*	P(γ)		WP_010274875.1	406	BLUF+EAL	1426
315	*Perlucidibaca piscinae*	P(γ)		WP_022956463.1	150	Short-BLUF	1427
316	*Plautia stali symbiont*	P(γ)		YP_008652566.1	149	Short-BLUF	1428
				YP_008649935.1	398	BLUF+EAL	1429
				WP_010615636.1	386	BLUF+EAL	1430
317	*Pseudoalteromonas atlantica*	P(γ)		WP_011573653.1	138	Short-BLUF	1431
	Pseudoalteromonas atlantica (T6c / BAA-1087)	P(γ)	Q15XU6	YP_660346.1	138	Short-BLUF	1432
			Q15W34	YP_660958.1	133	Short-BLUF	1433
318	*Pseudoalteromonas citrea*	P(γ)		WP_010367293.1	139	Short-BLUF	1434
	Pseudoalteromonas citrea NCIMB 1889	P(γ)	U1KR28	WP_010361781.1	145	Short-BLUF	1435
319	*Pseudoalteromonas luteoviolacea*	P(γ)		WP_023398227.1	138	Short-BLUF	1436
				WP_023397937.1	143	Short-BLUF	1437
	Pseudoalteromonas luteoviolacea B = ATCC 29581	P(γ)	L8D6G6	WP_005493992.1	144	Short-BLUF	1438
			L8DBD1	WP_005493516.1	139	Short-BLUF	1439
320	*Pseudoalteromonas marina mano4*	P(γ)	U1JNR5	WP_010557655.1	141	Short-BLUF	1440
321	*Pseudoalteromonas rubra ATCC 29570*	P(γ)	U1LSW8	WP_010383116.1	142	Short-BLUF	1441
322	*Pseudoalteromonas ruthenica*	P(γ)		WP_022946637.1	140	Short-BLUF	1442
323	*Pseudoalteromonas sp. BSi20311*	P(γ)	G7EN43	WP_008108394.1	141	Short-BLUF	1443
	Pseudoalteromonas sp. BSi20439	P(γ)	G7FK05	GAA73478.1	141	Short-BLUF	1444
	Pseudoalteromonas sp. BSi20480	P(γ)	G7FLG8	WP_008127010.1	141	Short-BLUF	1445
	Pseudoalteromonas sp. BSi20652	P(γ)	G7ED85	WP_008166519.1	141	Short-BLUF	1446
	Pseudoalteromonas sp. PAMC 22718	P(γ)		WP_016900061.1	141	Short-BLUF	1447
	Pseudoalteromonas sp. SM9913	P(γ)	E6RIH7	YP_004067295.1	138	Short-BLUF	1448
324	*Pseudoalteromonas spongiae*	P(γ)		WP_010558562.1	143	Short-BLUF	1449
				WP_010562063.1	142	Short-BLUF	1450
325	*Pseudoalteromonas tunicata D2*	P(γ)	A4C8E2	WP_009838119.1	140	Short-BLUF	1451
			A4CA03	WP_009840043.1	133	Short-BLUF	1452
326	*Pseudoalteromonas undina NCIMB 2128*	P(γ)	U1MG36	WP_010392015.1	141	Short-BLUF	1453
327	*Pseudomonas alcaligenes NBRC 14159*	P(γ)	U2ZK93	WP_021699986.1	144	Short-BLUF	1454
328	*Pseudomonas alcaliphila*	P(γ)		WP_017675860.1	144	Short-BLUF	1455
329	*Pseudomonas pseudoalcaligenes CECT 5344*	P(γ)	I7K7F2	WP_003463988.1	267	Short-BLUF	1456
330	*Psychrobacter aquaticus*	P(γ)		WP_021813036.1	174	Short-BLUF	1457
				WP_021815321.1	156	Short-BLUF	1458
				WP_021812947.1	202	Short-BLUF	1459
				WP_021813035.1	145	Short-BLUF	1460
				WP_021814263.1	210	Short-BLUF	1461
				WP_021815442.1	168	Short-BLUF	1462
331	*Psychrobacter arcticum*	P(γ)	Q4FUS2	YP_263670.1	210	Short-BLUF	1463
332	*Psychrobacter cryohalolentis (strain K5)*	P(γ)	Q1QDR2	YP_579675.1	210	Short-BLUF	1464
			Q1Q9K9	YP_581128.1	196	Short-BLUF	1465
			Q1QAA1	YP_580886.1	169	Short-BLUF	1466
333	*Psychrobacter lutiphocae*	P(γ)		WP_019674133.1	215	Short-BLUF	1467
				WP_019672901.1	213	Short-BLUF	1468
334	*Psychrobacter sp. 1501(2011)*	P(γ)	F5SNV9	WP_007394103.1	209	Short-BLUF	1469
			F5SLW1	WP_007393405.1	216	Short-BLUF	1470
	Psychrobacter sp. G	P(γ)	S4YVW0	YP_008163550.1	169	Short-BLUF	1471
			T1EBM8	YP_008163896.1	207	Short-BLUF	1472
			S4YSN3	YP_008162349.1	210	Short-BLUF	1473
	Psychrobacter sp. PAMC 21119	P(γ)		WP_010201341.1	172	Short-BLUF	1474
				WP_010201340.1	145	Short-BLUF	1475
				WP_010196365.1	207	Short-BLUF	1476
				WP_010201745.1	208	Short-BLUF	1477

	Psychrobacter sp. PRwf-1	P(γ)	A5WCY1	YP_001279472.1	209	Short-BLUF	1478
			A5WEX6	YP_001280167.1	216	Short-BLUF	1479
335	*Psychromonas ingrahamii (strain 37)*	P(γ)	A1SZ77	YP_944391.1	157	Short-BLUF	1480
336	*Rahnella aquatilis ATCC 33071*	P(γ)	H2J1E2	YP_005220397.1	413	BLUF+EAL	1481
	Rahnella aquatilis HX2	P(γ)	H8P0G4	YP_005419028.1	413	BLUF+EAL	1482
337	*Rahnella sp. (strain Y9602)*	P(γ)	E8XZI5	YP_004215373.1	413	BLUF+EAL	1483
338	*Reinekea sp. MED297*		A4BGV1	WP_008047183.1	172	Short-BLUF	1484
339	*Rheinheimera sp. A13L*	P(γ)	F7NUK1	WP_008898335.1	141	Short-BLUF	1485
340	*Salinisphaera shabanensis E1L3A*	P(γ)	F7Q923	WP_006913789.1	142	Short-BLUF	1486
341	*Serratia sp. M24T3*	P(γ)	I0QUJ8	WP_009636506.1	404	BLUF+EAL	1487
342	*Shewanella oneidensis*	P(γ)	Q8EBA3	NP_719171.1	140	Short-BLUF	1488
343	*Shewanella woodyi ATCC 51908*	P(γ)	B1KH49	YP_001762456.1	156	Short-BLUF	1489
344	*Shigella boydii 4444-74*	P(γ)	I6EHF9	EIQ43451.1/WP_000958290.1	133	Short-BLUF	1490
	Shigella boydii 5216-82	P(γ)	F3WJP8	WP_004992970.1	372	BLUF+EAL	1491
	Shigella boydii 965-58	P(γ)	I6DEL8	WP_004999597.1	403	BLUF+EAL	1492
	Shigella boydii ATCC 9905	P(γ)	E7T1P3	WP_004976550.1	372	BLUF+EAL	1493
345	*Shigella dysenteriae 1012*	P(γ)	B3X612	WP_005013749.1	311	BLUF+ EAL	1494
	Shigella dysenteriae 155-74	P(γ)	F3V2I3	WP_000958286.1	342	BLUF+EAL	1495
346	*Shigella flexneri 1485-80*	P(γ)	K0XUP4	EJZ67510.1/WP_000958290.1	133	Short-BLUF	1496
	Shigella flexneri 2850-71	P(γ)	I6BWJ3	WP_005109173.1	261	BLUF+EAL	1497
	Shigella flexneri CCH060	P(γ)	I6C3N2	EIQ14124.1/WP_000958290.1	133	Short-BLUF	1498
	Shigella flexneri J1713	P(γ)	F7R7D0	WP_005101736.1	386	BLUF+EAL	1499
	Shigella flexneri VA-6	P(γ)	F5N0U1	WP_005067857.1	372	BLUF+EAL	1500
347	*Shigella sonnei 53G*	P(γ)	E7K4N8	WP_005140026.1	198	Short-BLUF	1501
348	*Shigella sp. D9*	P(γ)	F4NHE2	EGJ05355.1/WP_000243121.1	429	BLUF+EAL	1502
349	*Simiduia agarivorans SA1 = DSM 21679*	P(γ)		YP_006916092.1	176	Short-BLUF	1503
350	*Spongiibacter tropicus*	P(γ)		WP_022958549.1	139	Short-BLUF	1504
351	*Stenotrophomonas maltophilia*	P(γ)		WP_019661581.1	139	Short-BLUF	1505
				WP_019659714.1	155	Short-BLUF	1506
	Stenotrophomonas maltophilia Ab55555	P(γ)	J7V2U9	WP_005408981.1/YP_001971521.1	144	Short-BLUF	1507
			J7VQR4	WP_005409694.1	155	Short-BLUF	1508
	Stenotrophomonas maltophilia AU12-09	P(γ)	M5TMH3	WP_005416006.1	144	Short-BLUF	1509
			M5TWK9	WP_005416570.1	155	Short-BLUF	1510
	Stenotrophomonas maltophilia D457	P(γ)	I0KM94	YP_006184358.1	144	Short-BLUF	1511
			I0KNV6	YP_006184918.1	155	Short-BLUF	1512
	Stenotrophomonas maltophilia EPM1	P(γ)	M3FAW7	YP_001971521.1	144	Short-BLUF	1513
	Stenotrophomonas maltophilia EPM1	P(β)	M3G6A4	WP_005413472.1	155	Short-BLUF	1514
	Stenotrophomonas maltophilia JV3	P(γ)	G0JUR4	YP_004792041.1	144	Short-BLUF	1515
			G0K4E1	YP_004794149.1	143	Short-BLUF	1516
			G0JY27	YP_004792350.1	142	Short-BLUF	1517
	Stenotrophomonas maltophilia K279a	P(γ)	B2FSB2	YP_001972310.1	155	Short-BLUF	1518
			B2FNU4	YP_001971901.1	144	Short-BLUF	1519
			B2FSB7	YP_001972315.1	132	Short-BLUF	1520
			B2FK66	CAQ45217.1/YP_001971521.1	144	Short-BLUF	1521
			B2FSU5	YP_001972350.1	157	Short-BLUF	1522
			B2FSA5	YP_001972303.1	149	Short-BLUF	1523
	Stenotrophomonas maltophilia MF89	P(γ)	T5KGS5	WP_019658901.1	144	Short-BLUF	1524
			T5L8X7	WP_021202418.1	155	Short-BLUF	1525
	Stenotrophomonas maltophilia RA8	P(γ)	M5D728	YP_002027818.1	144	Short-BLUF	1526
	Stenotrophomonas maltophilia R551-3	P(γ)	B4STM1	YP_002028128.1	142	Short-BLUF	1527
			B4SR64	ACF51135.1/YP_002027818.1	144	Short-BLUF	1528
	Stenotrophomonas maltophilia SKK35	P(γ)	M5CLY6	CCP10895.1	144	Short-BLUF	1529
	Stenotrophomonas sp. SKA14	P(γ)	B8L2D7	WP_008267235.1	144	Short-BLUF	1530

			B8LAI7	WP_008268182.1	142	Short-BLUF	1531
352	*Teredinibacter turnerae*	P(γ)		WP_018415581.1	138	Short-BLUF	1532
				WP_018276843.1	138	Short-BLUF	1533
353	*Thioalkalimicrobium aerophilum AL3*	P(γ)	G4D9V3	WP_006459896.1	135	Short-BLUF	1534
354	*Thioalkalivibrio sp. HL-EbGR7*	P(γ)	B8GPZ7	YP_002515131.1	168	Short-BLUF	1535
	Thioalkalivibrio sp. K90mix	P(γ)	D3SBJ7	YP_003461138.1	196	Short-BLUF	1536
355	*Thiocystis violascens (Chromatium violascens) ATCC 17096*	P(γ)	I3YBW9	YP_006414612.1	597	GGDEF+EAL+BLUF	1537
356	*Thioflavicoccus mobilis 8321*	P(γ)	L0H2H2	YP_007245882.1	293	BLUF+PsiE	1538
357	*Thiorhodococcus drewsii AZ1*	P(γ)	G2DZK4	WP_007040181.1	1199	2PAS+GGDEF+EAL+BLUF	1539
			G2E4Y1	WP_007042057.1	151	Short-BLUF	1540
358	*Thiorhodospira sibirica ATCC 700588*	P(γ)	G4E7K7	WP_006788315.1	139	Short-BLUF	1541
			G4E8F6	WP_006788612.1	1099	2PAS+GGDEF+EAL+BLUF	1542
359	*Thiorhodovibrio sp. 970*	P(γ)	H8Z515	WP_009150825.1	148	Short-BLUF	1543
			H8Z1A9	WP_009148009.1	299	Short-BLUF	1544
360	*uncultured gamma proteobacterium*	P(γ)U	H5SE04	BAL54390.1	113	Short-BLUF	1545
361	*uncultured Thiohalocapsa sp. PB-PSB1*	P(γ)		WP_023410594.1	183	Short-BLUF	1546
				WP_023414210.1	149	Short-BLUF	1547
362	*Vibrio*	P(γ)		WP_017060344.1	139	Short-BLUF	1548
363	*Vibrio breoganii*	P(γ)		WP_017029863.1	141	Short-BLUF	1549
				WP_017031909.1	141	Short-BLUF	1550
364	*Vibrio cholera RC385*	P(γ)	D7HCX4	WP_001885575.1	142	Short-BLUF	1551
	Vibrio cholerae	P(γ)		WP_019829651.1	142	Short-BLUF	1552
365	*Vibrio coralliilyticus ATCC BAA-450*	P(γ)	C9NR36	WP_006958871.1	139	Short-BLUF	1553
			C9NQ17	WP_006958783.1	136	Short-BLUF	1554
			C9NPY7	WP_006958749.1	104	Short-BLUF	1555
			C9NVS2	WP_006961401.1	141	Short-BLUF	1556
	Vibrio coralliilyticus OCN008	P(γ)	U0ET87	WP_019275344.1	141	Short-BLUF	1557
366	*Vibrio crassostreae*	P(γ)		WP_017065379.1	139	Short-BLUF	1558
				WP_017067227.1	139	Short-BLUF	1559
				WP_017070503.1	139	Short-BLUF	1560
367	*Vibrio cyclitrophicus*	P(γ)		WP_016768597.1	139	Short-BLUF	1561
				WP_010440401.1	139	Short-BLUF	1562
				WP_016796603.1	139	Short-BLUF	1563
				WP_016793122.1	139	Short-BLUF	1564
368	*Vibrio genomosp. F10*	P(γ)		WP_017038440.1	139	Short-BLUF	1565
				WP_017034560.1	139	Short-BLUF	1566
	Vibrio genomosp. F6	P(γ)		WP_017052207.1	139	Short-BLUF	1567
369	*Vibrio ichthyoenteri ATCC 700023*	P(γ)	F9S3C4	WP_006712577.1	134	Short-BLUF	1568
370	*Vibrio nigripulchritudo*	P(γ)		WP_022604223.1	138	Short-BLUF	1569
				YP_008620961.1	138	Short-BLUF	1570
				WP_022589537.1	127	Short-BLUF	1571
				WP_022600546.1	127	Short-BLUF	1572
				YP_008641840.1	127	Short-BLUF	1573
	Vibrio nigripulchritudo ATCC 27043	P(γ)	F9THX2	WP_004400464.1	138	Short-BLUF	1574
	Vibrio nigripulchritudo ATCC 27043	P(γ)	F9TGM9	EGU61078.1/WP_004399602.1	127	Short-BLUF	1575
371	*Vibrio ordalii*	P(γ)		WP_010320661.1	145	Short-BLUF	1576
372	*Vibrio orientalis CIP 102891 = ATCC 33934*	P(γ)	C9QIB2	WP_004414323.1	60	Short-BLUF	1577
373	*Vibrio scophthalmi LMG 19158*	P(γ)	F9RIQ0	WP_005592861.1	139	Short-BLUF	1578
374	*Vibrio sinaloensis DSM 21326*	P(γ)	E8M6S4	WP_008076812.1	139	Short-BLUF	1579
375	*Vibrio sp. MED222*	P(γ)	A3Y2H2	WP_009847120.1	142	Short-BLUF	1580
376	*Vibrio splendidus*	P(γ)		WP_017079037.1	139	Short-BLUF	1581
				WP_017090104.1	139	Short-BLUF	1582
				WP_017093202.1	139	Short-BLUF	1583

				WP_017073405.1	139	Short-BLUF	1584
				WP_019824217.1	139	Short-BLUF	1585
	Vibrio splendidus 12B01	**P(γ)**	A3UYZ3	WP_004734641.1	141	Short-BLUF	1586
	Vibrio splendidus Mel32	**P(γ)**	B7VGM9	YP_002417504.1	139	Short-BLUF	1587
377	*Vibrio tasmaniensis*	**P(γ)**		WP_017097344.1	139	Short-BLUF	1588
378	*Vibrio tubiashii ATCC 19109*	**P(γ)**	F9T3Q3	EGU56983.1/WP_004744136.1	151	Short-BLUF	1589
	Vibrio tubiashii NCIMB 1337 = ATCC 19106	**P(γ)**	I1DCC9	WP_004744136.1	151	Short-BLUF	1590
379	*Vibrio vulnificus*	**P(γ)**		WP_017420365.1	139	Short-BLUF	1591
	Vibrio vulnificus	**P(γ)**		WP_017790365.1	139	Short-BLUF	1592
380	*Vibrionales bacterium SWAT-3*	**P(γ)**	A5L553	WP_008222460.1	139	Short-BLUF	1593
381	*Xanthomonadaceae*	**P(γ)**		WP_010486035.1	144	Short-BLUF	1594
382	*Xanthomonas axonopodis*	**P(γ)**		WP_016850851.1	159	Short-BLUF	1595
				WP_017158845.1	158	Short-BLUF	1596
				WP_017156640.1	159	Short-BLUF	1597
383	*Xanthomonas axonopodis pv. citri str.306*	**P(γ)**	Q8PHH6	AAM38122.1/NP_643586.1	147	Short-BLUF	1598
			Q8PKP9	AAM36973./NP_642437.1	158	Short-BLUF	1599
384	*Xanthomonas axonopodis pv. punicae str. LMG 859*	**P(γ)**	H1XN33	WP_005934425.1	128	Short-BLUF	1600
	Xanthomonas axonopodis Xac29-1	**P(γ)**	M4TW81	YP_007636565.1/NP_642437.1	158	Short-BLUF	1601
			M4TZI7	NP_643586.1	147	Short-BLUF	1602
385	*Xanthomonas citri pv. mangiferaeindicae LMG 941*	**P(γ)**	H8FLF5	WP_003490419.1	158	Short-BLUF	1603
386	*Xanthomonas citri subsp. citri Aw12879*	**P(γ)**	M4WEN8	NP_642437.1	158	Short-BLUF	1604
387	*Xanthomonas fuscans subsp. aurantifolii str. ICPB 10535*	**P(γ)**	D4TC91	WP_007975774.1	147	Short-BLUF	1605
			D4SRA6	WP_007962696.1	159	Short-BLUF	1606
388	*Xanthomonas fuscans subsp. fuscans*	P(γ)		YP_008638953.1	159	Short-BLUF	1607
389	*Xanthomonas perforans 91-118*	**P(γ)**	F0BUQ3	WP_008575691.1	184	Short-BLUF	1608
390	*Xanthomonas sp. M97*	**P(γ)**		WP_022971905.1	144	Short-BLUF	1609
391	*Bacteriovorax sp. BAL6_X*	**P(δ)**	T0C040	WP_021268210.1	153	Short-BLUF	1610
392	*Bdellovibrio bacteriovorus str. Tiberius*	**P(δ)**	K7YL37	YP_007021703.1	142	Short-BLUF	1611
393	*Desulfatibacillum alkenivorans AK-01l*	**P(δ)**	B8FK53	YP_002430196.1	144	Short-BLUF	1612
394	*Desulfococcus oleovorans Hxd3*	**P(δ)**	A9A0H4	YP_001529551.1	148	Short-BLUF	1613
395	*Desulfovibrio longus*	**P(δ)**		WP_022663022.1	146	Short-BLUF	1614
396	*Myxococcus xanthus DK1622*	**P(δ)**	Q93SK7		253	Short-BLUF	1615
397	*uncultured Desulfofustis sp. PB-SRB1*	**P(δ)Unc**		WP_023408928.1	144	Short-BLUF	1616
398	*Magnetococcus sp. MC-1*	**P(Magn)**	A0LAZ4	YP_866543.1	1118	PAS+GGDEF+EAL+BLUF	1617
			A0L5S5	YP_864724.1	139	Short-BLUF	1618
			A0LAQ1	YP_866450.1	160	Short-BLUF	1619
399	*Thiovulum sp. ES*	**P(ε)**	J0L733	WP_008350312.1	143	Short-BLUF	1620
			J1FAS2	WP_008350313.1	143	Short-BLUF	1621
400	*zeta proteobacterium SCGC AB-137-C09*	**P(ζ)**		WP_018281521.1	291	BLUF+PsiE	1622
401	*candidate division TG3 bacterium ACht1*	**Unc**		WP_022635648.1	141	Short-BLUF	1623
402	*uncultured bacterium*	**Unc**	K2GM21	EKE24165.1	122	Short-BLUF	1624
403	*uncultured bacterium 581*	**PUnc**	Q6SFE3	AAR38275.1/WP_007234700.1	159	Short-BLUF	1625
404	*uncultured proteobacterium delRiverFos06H03*	**PUnc**	Q58PP1	AAX48191.1	147	Short-BLUF	1626
405	*uncultured proteobacterium.*	**PUnc**	Q8KZ47	AAM48626.1	146	Short-BLUF	1627
406	*Cyanothece sp. PCC 7425 / ATCC 29141*	**Cya**	B8HWP0	YP_002484827.1	145	Short-BLUF	1628
407	*Halothece sp. PCC 7418 (Synechococcus sp. PCC 7418)*	**Cya**	K9YCI0	YP_007168017.1	163	Short-BLUF	1629
408	*Oscillatoria acuminata PCC 6304*	Cya	K9TLZ5	YP_007087096.1	366	BLUF+GGDEF	1630
409	*Pseudanabaena biceps PCC 7429*	Cya	L8MTI5	WP_009629186.1	370	BLUF+GGDEF	1631
410	*Synechococcus sp. (strain ATCC 27167 / PCC 6312)*	**Cya**	K9RSY2	YP_007060755.1	141	Short-BLUF	1632
	Synechococcus sp. PCC 7002	**Cya**	B1XJZ3	YP_001734377.1	145	Short-BLUF	1633
	Synechococcus sp. PCC 7502	**Cya**	K9SXI7	YP_007106786.1	153	Short-BLUF	1634
411	*Synechocystis sp. PCC 6803*	**Cya**	L8AQ59	NP_441709.1	150	Short-BLUF	1635
			F7UR00	BAK50562.1/YP_005651767.1	150	Short-BLUF	1636

			P74295	YP_005651767.1/NP_441709.1	150	Short-BLUF	1637
	Synechocystis sp. PCC 6803 substr. GT-I	**Cya**	H0NXE6	YP_005383576.1	150	Short-BLUF	1638
	Synechocystis sp. PCC 6803 substr. PCC-N	**Cya**	H0P9U8	YP_005409452.1	150	Short-BLUF	1639
	Synechocystis sp. PCC 6803 substr. PCC-P	**Cya**	H0PNV0	YP_005386745.1	150	Short-BLUF	1640
412	*Thermosynechococcus elongatus*	**Cya**	Q8DMN3	NP_680869.1	143	Short-BLUF	1641
413	*Rhodopirellula baltica*	**Pl**	Q7UXU1	NP_864233.1	141	Short-BLUF	1642
	Rhodopirellula baltica WH47	**Pl**	F2AXT1	WP_007328441.1	132	Short-BLUF	1643
414	*Rhodopirellula europaea SH398*	**Pl**	M5S3C7	WP_008667793.1	141	Short-BLUF	1644
415	*Rhodopirellula sallentina SM41*	**Pl**	M5U559	WP_008676976.1	166	Short-BLUF	1645
	Rhodopirellula sp. SWK7	**Pl**	M5SVB2	WP_009104720.1	154	Short-BLUF	1646
			M5TST8	WP_009093809.1	184	Short-BLUF	1647
416	*Candidatus Nitrospira defluvii*	**Nit**	D8PAY1	YP_003796316.1	138	Short-BLUF	1648
417	*Bdellovibrio bacteriovorus*	**Bact**	Q6MPS8	NP_967726.1	142	Short-BLUF	1649
418	*Cellulophaga algicola DSM 14237*	**Bact**	E6X5L6	YP_004163880.1	144	Short-BLUF	1650
419	*Cesiribacter andamanensis AMV16*	**Bact**	M7N429	WP_009196222.1	143	Short-BLUF	1651
420	*Chloroherpeton thalassium ATCC 35110*	**Bact**	B3QX03	YP_001997260.1	145	Short-BLUF	1652
421	*Cytophagales str. B6*	**Bact**		WP_022830361.1	140	Short-BLUF	1653
422	*Dokdonia donghaensis MED134*	**Bact**	A2TPW0	EAQ40501.1	135	Short-BLUF	1654
423	*Eudoraea adriatica*	**Bact**		WP_019668996.1	146	Short-BLUF	1655
424	*Fibrella aestuarina BUZ 2*	**Bact**	I0KAE1	YP_007321687.1	143	Short-BLUF	1656
425	*Fibrisoma limi BUZ 3*	**Bact**	I2GNV8	WP_009284154.1	229	Short-BLUF	1657
426	*Flavobacteria bacterium BBFL7.*	**Bact**	Q26D86	WP_006795753.1	139	Short-BLUF	1658
427	*Flavobacteriaceae bacterium P7-3-5*	**Bact**		WP_019387910.1	136	Short-BLUF	1659
428	*Formosa agariphila KMM 3901*	**Bact**		CDF79453.1	144	Short-BLUF	1660
429	*Fulvivirga imtechensis AK7*	**Bact**	L8JX15	WP_009577900.1	139	Short-BLUF	1661
430	*Gillisia limnaea DSM 15749*	**Bact**	H2BX58	WP_006988327.1	134	Short-BLUF	1662
			H2BW61	WP_006989286.1	133	Short-BLUF	1663
			H2BVP6	WP_006990305.1	135	Short-BLUF	1664
			H2BX59	WP_006988328.1	135	Short-BLUF	1665
431	*Gramella forsetii KT0803*	**Bact**	A0LYB8	YP_860431.1	133	Short-BLUF	1666
432	*Hymenobacter aerophilus*	**Bact**		WP_019949502.1	139	Short-BLUF	1667
				WP_019946446.1	145	Short-BLUF	1668
				WP_019946896.1	145	Short-BLUF	1669
433	*Hymenobacter norwichensis*	**Bact**		WP_022823388.1	142	Short-BLUF	1670
				WP_022823740.1	153	Short-BLUF	1671
				WP_022821755.1	146	Short-BLUF	1672
434	*Krokinobacter sp. (strain 4H-3-7-5)*	**Bact**	F4B036	YP_004431557.1	137	Short-BLUF	1673
			F4B057	YP_004431578.1	151	Short-BLUF	1674
435	*Marivirga tractuosa ATCC 23168*	**Bact**	E4TNQ3	YP_004053598.1	139	Short-BLUF	1675
436	*Mesoflavibacter zeaxanthinifaciens*	**Bact**		WP_010517271.1	142	Short-BLUF	1676
437	*Microscilla marina ATCC 23134*	**Bact**	A1ZRF7	WP_004156404.1	137	Short-BLUF	1677
438	*Mucilaginibacter paludis DSM 18603*	**Bact**	H1Y0M8	WP_008509682.1	140	Short-BLUF	1678
			H1YF64	WP_008506222.1	151	Short-BLUF	1679
			H1Y0M7	WP_008509681.1	137	Short-BLUF	1680
			H1Y8V0	WP_008509607.1	151	Short-BLUF	1681
439	*Nafulsella turpanensis*	**Bact**		WP_017730115.1	142	Short-BLUF	1682
440	*Pedobacter sp. BAL39*	**Bact**	A6E9F4	WP_008239021.1	152	Short-BLUF	1683
440	*Polaribacter dokdonensis MED152*	**Bact**	A2TY78	YP_007671791.1	481	HTH-BLUF	1684
440	*Psychroflexus gondwanensis ACAM 44*	**Bact**	N1WKU3	WP_003440963.1	142	Short-BLUF	1685
440	*Psychroflexus torquis ATCC 700755*	**Bact**	K4IV57	YP_006868110.1	142	Short-BLUF	1686
440	*Segetibacter koreensis*	**Bact**		WP_018614590.1	151	Short-BLUF	1687
440	*Spirosoma linguale DSM 74*	**Bact**	D2QRB5	YP_003388468.1	142	Short-BLUF	1688
			D2QVZ0	YP_003391771.1	137	Short-BLUF	1689

441	*Spirosoma spitsbergense*	**Bact**		WP_020603065.1	152	Short-BLUF	1690
	Spirosoma spitsbergense	**Bact**		WP_020601880.1	152	Short-BLUF	1691
442	*Terriglobus saanensis ATCC BAA-1853*	**Bact**	E8UXB9	YP_004184137.1	153	Short-BLUF	1692
443	*unidentified eubacterium SCB49*	**Bact**	A6ENE1	EDM45157.1	133	Short-BLUF	1693
444	*Winogradskyella psychrotolerans*	**Bact**		WP_020894508.1	136	Short-BLUF	1694
445	*Zobellia galactanivorans DSM 12802*	**Bact**	G0L2R9	YP_004735541.1	145	Short-BLUF	1695
			G0KZL8	YP_004737334.1	147	Short-BLUF	1696
446	*Zunongwangia profunda SM-A87*	**Bact**	D5BH98	YP_003583468.1	133	Short-BLUF	1697
447	*Solibacter usitatus Ellin6076*	**AcidoB**	Q01S10	YP_827845.1	141	Short-BLUF	1698
448	*Lentisphaera araneosa HTCC2155*	**Lent**	A6DQ35	WP_007279966.1	132	Short-BLUF	1699
449	*Chthoniobacter flavus Ellin428*	**Verr**	B4D369	WP_006980682.1	157	Short-BLUF	1700
450	*Coraliomargarita akajimensis DSM 45221*	**Verr**	D5EM63	YP_003549393.1	148	Short-BLUF	1701
451	*Verrucomicrobiae bacterium DG1235*	**Verr**	B5JMC0	WP_008104971.1	141	Short-BLUF	1702
			B5JJ76	WP_008103776.1	157	Short-BLUF	1703
452	*Leptonema illini DSM 21528*	**Spi**	H2CIX2	WP_002774553.1	261	BLUF+GAF ?	1704
453	*Turneriella parva (Leptospira parva) ATCC BAA-1111*	**Spi**	I4B432	YP_006439545.1	353	BLUF+GGDEF	1705
