# Supplementary material for: From Plant Infectivity to Growth Patterns: The Role of Blue-Light Sensing in the Prokaryotic World
Source: Plants (Basel). 2014 Jan 27;3(1):70–94. doi: 10.3390/plants3010070 (PMC4844311; doi:10.3390/plants3010070)
Supplement: Supplementary File 1 [file plants-03-00070-s001.zip › plants-46379-supplementary-final/Supplementary Material - S3 - LOV archaea.pdf]

Archaeal LOV domains

Nr.	Species/strain	Group	Accession nr. Uniprot	Accession nr. NCBI	aa nr.	Domains	Prot. nr
1	*Haladaptatus paucihalophilus DX253*	**Eu A**	E7QV78	WP_007980661.1	621	PAS+LOV+GAF+HTH	1
2	*Halalkalicoccus jeotgali DSM 18796*	**Eu A**	D8J3G3	YP_003737062.1	531	PAS+LOV+GAF+HTH	2
			G0HVV3	YP_004795441.1	726	RR+PAS+LOV+PAS+Kinase	3
3	*Halarchaeum acidiphilum*	**EuA**		WP_021780048.1	548	?-LOV	4
4	*Haloarcula amylolytica JCM 13557*	**EuA**	M0KRQ6	WP_008309224.1	748	RR+LOV+GAF+Kinase	5
			M0KMX5	WP_008310353.1	727	RR+PAS+LOV+Kinase	6
5	*Haloarcula argentinensis DSM 12282*	**EuA**	M0KWF6	WP_005534440.1	748	RR+LOV+GAF+Kinase	7
			M0KU22	WP_005535584.1	726	RR+PAS+LOV+Kinase	8
6	*Haloarcula californiae ATCC 33799*	**EuA**	M0KKE6	WP_007188243.1	726	RR+PAS+LOV+Kinase	9
			M0JSQ9	WP_007190397.1	748	RR+LOV+GAF+Kinase	10
7	*Haloarcula hispanica ATCC 33960*	**EuA**	G0HRB1	YP_004796084.1	748	RR+PAS+LOV+GAF+Kinase	11
8	*Haloarcula japonica DSM 6131*	**EuA**	M0L9V9	WP_004592775.1	748	RR+LOV+GAF+Kinase	12
			M0LBV1	WP_004593543.1	726	RR+PAS+LOV+Kinase	13
			M0LAY8	WP_004594204.1	599	RR+LOV+Kinase	14
9	*Haloarcula marismortui ATCC43049*	**EuA**	Q5UWI7	YP_138072.1	2306	PAS+GAF+4PAS+GAF+4PAS+LOV+3PAS+Kinase	15
			Q5V5P7	YP_134861.1	726	RR+PAS+LOV+PAS+Kinase	16
			Q5V3S3	YP_135535.1	748	RR+PAS+LOV+GAF+Kinase	17
10	*Haloarcula sinaiiensis ATCC 33800*	**EuA**	M0K0A6	WP_004961457.1	748	RR+LOV+GAF+Kinase	18
			M0JUG2	WP_004963306.1	726	RR+PAS+LOV+Kinase	19
			M0JKN0	WP_004966422.1	870	RR+PAS+LOV+3PAS	20
11	*Haloarcula vallismortis ATCC 29715*	**EuA**	M0IYI4	WP_004517958.1	748	RR+PAS+LOV+GAF+Kinase	21
			M0JF54	WP_004516790.1	726	RR+PAS+LOV+Kinase	22
12	*Halobiforma lacisalsi AJ5*	**EuA**		WP_007141411.1	1462	GAF+LOV+3PAS+2GAF+HTH	23
				WP_007142266.1	628	PAS+LOV+HTH	24
13	*Halobiforma nitratireducens JCM 10879*	**EuA**	M0LF83	WP_006674107.1	633	PAS+LOV+HTH	25
14	*Haloferax alexandrinus JCM 10717*	**EuA**	M0I5C6	WP_006600750.1	658	LOV+GAF+HTH	26
			M0I6S2	WP_006600643.1	858	LOV+4PAS+Kinase	27
				WP_004062881.1	725	RR+PAS+LOV+PAS+Kinase	28
15	*Haloferax denitrificans ATCC 35960*	**EuA**	M0JKW4	WP_004967303.1	639	RR+LOV+PAS+Kinase	29
			M0JGT6	WP_004967308.1	390	RR+LOV	30
			M0JA63	WP_004969028.1	659	LOV+GAF+HTH	31
			M0J0Q3	WP_004970246.1	858	LOV+3PAS+Kinase	32
16	*Haloferax elongans ATCC BAA-1513*	**EuA**	M0HS61	WP_008322780.1	661	LOV+GAF+HTH	33
17	*Haloferax gibbonsii ATCC 33959*	**EuA**	M0H5U8	WP_004975760.1	716	RR+PAS+LOV+PAS+Kinase	34
			M0H5P9	WP_004975752.1	369	RR+LOV	35
			M0HJ57	WP_004973634.1	658	LOV+GAF+HTH	36
			M0HIK5	WP_004972991.1	870	RR+LOV+3PAS+Kinase	37
18	*Haloferax larsenii JCM 13917*	**EuA**	M0GTD6	WP_007544473.1	661	LOV+GAF+HTH	38
19	*Haloferax lucentense DSM 14919*	**EuA**	M0GV43	ELZ87863.1/WP_004062881.1	725	RR+PAS+LOV+PAS+Kinase	39
			M0GGW3	WP_004065380.1	658	LOV+GAF+HTH	40
			M0GFK0	WP_004065100.1	858	LOV+3PAS+Kinase	41
20	**Haloferax mediterranei ATCC 33500*	**EuA**	I3R1D3	YP_006348030.1	667	LOV+GAF+HTH	42
21	*Haloferax mucosum ATCC BAA-1512*	**EuA**	M0IDW1	WP_008319778.1	659	LOV+GAF+HTH	43

22	*Haloferax prahovense DSM 18310*	**EuA**	M0G333	WP_008095971.1	712	RR+PAS+LOV+PAS+Kinase	44
			M0GT38	WP_008091440.1	658	LOV+GAF+HTH	45
			M0GL79	WP_008091948.1	871	LOV+3PAS+Kinase	46
23	*Haloferax sp. ATCC BAA-644*	**EuA**		WP_008576999.1	658	LOV+GAF+HTH	47
				WP_008577397.1	858	LOV+3PAS+Kinase	48
	Haloferax sp. ATCC BAA-645	**EuA**	M0G7U5	WP_008577920.1	731	RR+PAS+LOV+PAS+Kinase	49
				WP_008577397.1	858	LOV+3PAS+Kinase	50
				ELZ63079.1/WP_008576999.1	658	LOV+GAF+HTH	51
	Haloferax sp. ATCC BAA-646	**EuA**	M0FM96	WP_008579033.1	817	RR+PAS+LOV+PAS+Kinase	52
				WP_008577397.1	858	LOV+3PAS+Kinase	53
			M0FI62	ELZ58294.1/WP_008576999.1	658	LOV+GAF+HTH	54
	Haloferax sp. BAB2207	**EuA**	L5NY38	WP_008605787.1	646	RR+PAS+LOV+PAS+Kinase	55
			L5NW28	WP_008606020.1	709	LOV+3PAS+Kinase	56
			L5NIY0	WP_008609214.1	575	LOV+GAF+HTH	57
24	*Haloferax sulfurifontis ATCC BAA-897*	**EuA**	M0I143	WP_007276041.1	640	RR+LOV+PAS+Kinase	58
			M0HZH1	WP_007276043.1	369	RR+PAS+LOV	59
			M0IBQ3	WP_007275007.1	606	LOV+GAF+HTH	60
			M0HZS1	WP_007275764.1	858	LOV+PAS+Kinase	61
25	*Haloferax volcanii DSM 3757*	**Eu A**	D4GU42	YP_003536054.1	652	RR+PAS+LOV+Kinase	62
			D4GSN6	YP_003534694.1	858	LOV+3PAS+Kinase	63
			D4GZU7	YP_003534395.1	658	LOV+GAF+HTH	64
26	*Halonotius*	**EuA**		WP_021039170.1	828	2PAS+LOV+2PAS+Kinase	65
27	*Halonotius sp. J07HN4*	**EuA**		WP_021040232.1	763	PAS+LOV+PAS+Kinase	66
				WP_021040161.1	1018	2PAS+LOV+GAF+2PAS+Kinase	67
				WP_021039668.1	966	RR+PAS+LOV+2PAS+Kinase	68
28	*Halonotius sp. J07HN6*	**EuA**		WP_021062171.1	335	RR+LOV (truncated LOV domain)	69
				WP_021061359.1	419	PAS+LOV	70
				WP_021061848.1	697	PAS+LOV+PAS+Kinase	71
29	*Halophilic archaeon DL31*	**EuA**	G2MKJ0	YP_004807311.1	400	LOV+Kinase	72
30	*Halophilic archaeon J07HX64*	**EuA**		WP_021044206.1	666	RR+LOV+HTH	73
31	*Halopiger xanaduensis DSM 18323*	**Eu A**	F8D4L2	YP_004596219.1	658	PAS+LOV+GAF+HTH	74
32	*Haloquadratum walsbyi*	**EuA**		WP_021050808.1	851	RR+PAS+LOV+PAS+Kinase	75
33	*Halorhabdus tiamatea SARL4B*	**Eu A**	F7PMY2	YP_008375964.1	677	RR+LOV+GAF+HTH	76
34	*Halorhabdus utahensis DSM 12940*	**EuA**	C7NQP5	YP_003129237.1	680	RR+LOV+GAF+HTH	77
35	*Halorubrum aidingense JCM 13560*	**EuA**	M0PCX2	WP_008001395.1	596	RR+PAS+LOV+Kinase	78
			M0PAC7	WP_008000810.1	522	RR+ LOV+Kinase	79
			M0PJA7	WP_007998980.1	981	LOV+5PAS+Kinase	80
36	*Halorubrum arcis JCM 13916*	**EuA**	M0PIL7	WP_007996097.1	596	RR+PAS+LOV+Kinase	81
			M0PIN6	WP_007996657.1	501	RR+ LOV+Kinase	82
37	*Halorubrum californiensis DSM 19288*	**EuA**	M0EGK2	WP_008441071.1	594	RR+PAS+LOV+Kinase	83
			M0DYT3	WP_008445922.1	683	PAS+LOV+GAF+PAS+Kinase	84
				WP_008443170.1	743	RR+PAS+LOV+Kinase	85
			M0EG61	WP_008441781.1	507	RR+ LOV+Kinase	86
38	*Halorubrum coriense DSM 10284*	**EuA**	M0E8M1	WP_006114486.1	594	RR+PAS+LOV+Kinase	87
			M0EM75	WP_006112652.1	510	RR+ LOV+Kinase	88
39	*Halorubrum distributum JCM 9100*	**EuA**	M0EQI8	WP_004597500.1	596	RR+PAS+LOV+Kinase	89
			M0EQ99	WP_004596794.1	502	RR+ LOV+Kinase	90
40	*Halorubrum hochstenium ATCC 700873*	**EuA**	M0F978	WP_008584939.1	594	RR+PAS+LOV+Kinase	91
			M0F8B0	WP_008584017.1	486	LOV+Kinase	92

41	*Halorubrum kocurii JCM 14978*	**EuA**	M0PAC1	WP_008847455.1	607	RR+PAS+LOV+Kinase	93
			M0NND4	WP_008849553.1	720	RR+PAS+LOV+Kinase	94
			M0NXJ3	WP_008848851.1	555	LOV+GAF+HTH	95
			M0P7I5	WP_008848390.1	493	RR+LOV+Kinase	96
42	*Halorubrum lacusprofundi ATCC 49239*	**EuA**	B9LXB3	YP_002567701.1	587	PAS+LOV+PAS+kinase	97
			B9LPK0	YP_002566358.1	294	LOV+Kinase	98
43	*Halorubrum lipolyticum DSM 21995*	**EuA**	M0NMJ4	WP_008007944.1	620	RR+PAS+LOV+Kinase	99
			M0NJB8	WP_008008306.1	494	LOV+Kinase	100
44	*Halorubrum litoreum JCM 13561*	**EuA**	M0NPX9	WP_008367060.1	596	RR+PAS+LOV+Kinase	101
			M0NL05	WP_008367611.1	501	RR+LOV+Kinase	102
45	*Halorubrum saccharovorum DSM 1137*	**EuA**	M0E2F0	WP_004047251.1	590	RR+PAS+LOV+Kinase	103
			M0DW79	WP_004048308.1	717	RR+PAS+LOV+PAS+Kinase	104
			M0E4X7	WP_004046404.1	475	RR+LOV+Kinase	105
46	*Halorubrum sp. T3*	**EuA**		WP_017343793.1	596	RR+PAS+LOV+Kinase	106
				WP_017344163.1	506	RR+LOV+Kinase	107
47	*Halorubrum tebenquichense DSM 14210*	**EuA**	M0DVK1	WP_006628902.1	1102	RR+PAS+LOV	108
			M0DE24	WP_006630444.1	461	LOV+Kinase	109
48	*Halorubrum terrestre JCM 10247*	**EuA**	M0D8R1	WP_007345452.1	596	RR+PAS+LOV+Kinase	110
			M0DD56	WP_007345203.1	501	RR+ LOV+Kinase	111
49	*Halosimplex carlsbadense 2-9-1*	**EuA**	M0CWF6	WP_006882975.1	972	RR+PAS+LOV+3PAS+Kinase	112
			M0D066	WP_006882606.1	652	RR+LOV+GAF+HTH	113
			M0D0B9	WP_006882998.1	648	GAF+PAS+LOV+Kinase	114
				WP_006882499.1	1794	LOV+8PAS+GAF+HTH	115
50	*Haloterrigena limicola JCM 13563*	**EuA**	M0CPI1	WP_008009308.1	633	PAS+LOV+GAF+HTH	116
51	*Haloterrigena salina JCM 13891*	**EuA**	M0BXZ7	WP_008896043.1	643	PAS+LOV+GAF+HTH	117
			M0C8L1	WP_008893875.1	652	GAF+PAS+LOV+Kinase	118
52	*Haloterrigena thermotolerans DSM 11522*	**EuA**	M0BP65	WP_006650177.1	627	PAS+LOV+GAF+HTH	119
53	*Haloterrigena turkmenica DSM 5511*	**Eu A**	D2RWH0	YP_003402232.1	644	PAS+LOV+GAF+HTH	120
			D2RZP8	YP_003404760.1	654	GAF+PAS+LOV+Kinase	121
54	*Halovivax asiaticus JCM 14624*	**Eu A**	M0BRR0	WP_007697375.1	674	RR+LOV+GAF+HTH	122
55	*Halovivax ruber XH-70*	**EuA**	L0IC85	YP_007283949.1	674	LOV+GAF+HTH	123
56	*Methanoculleus bourgensis ATCC 43281*	**EuA**		YP_006543787.1	630	2PAS+GAF+LOV+Kinase	124
				YP_006545702.1	971	4PAS+LOV+Kinase	125
57	*Methanoculleus marisnigri ATCC 35101*	**EuA**		YP_001047260.1	888	4PAS+LOV+Kinase	126
				YP_001046186.1	630	2PAS+GAF+LOV+Kinase	127
			A3CUN9	YP_001047071.1	753	2PAS+LOV+Kinase	128
58	*Methanolobus psychrophilus R15*	**EuA**		YP_006922864.1	541	LOV+Kinase+RR	129
59	*Methanoplanus limicola DSM 2279*	**EuA**	H1YYA6	WP_004075781.1	476	PAS+LOV+PAS+Kinase	130
60	*Methanosphaerula palustris ATCC BAA-1556*	**EuA**		YP_002465170.1	968	PAS+LOV+3PAS+Kinase	131
61	*Natrialba chahannaoensis JCM 10990*	**EuA**	M0B9X5	WP_006165579.1	478	RR+LOV+Kinase	132
62	*Natrialba hulunbeirensis JCM 10989*	**EuA**	L9ZYC6	WP_006653494.1	478	RR+LOV+Kinase	133
			M0AA97	WP_006651477.1	736	RR+2PAS+LOV+Kinase	134
63	*Natrialba magadii ATCC 43099*	**Eu A**	D3SVK4	YP_003480174.1	477	RR+LOV+Kinase	135
64	*Natrinema altunense JCM 12890*	**Eu A**	L9ZYN2	WP_007107921.1	629	PAS+LOV+HTH	136
65	*Natrinema gari JCM 14663*	**Eu A**	L9YWZ4	WP_008457298.1	630	PAS+LOV+HTH	137
66	*Natrinema pallidum DSM 3751*	**Eu A**	L9YJK9	WP_006187119.1	649	PAS+LOV+HTH	138
67	*Natrinema pellirubrum DSM 15624*	**Eu A**	L9YNP9	WP_006181371.1	657	LOV+GAF+HTH	139
			L0JQR8	YP_007281871.1	627	PAS+LOV+GAF+HTH	140
68	**Natrinema sp. (strain J7-2)*	**EuA**	I7C1F5	YP_006543291.1	630	PAS+LOV+HTH	141

69	*Natrinema versiforme JCM 10478*	**EuA**	L9XNH7	WP_006432901.1	717	RR+PAS+LOV+PAS+Kinase	142
			L9YBA9	WP_006429256.1	631	PAS+LOV+HTH	143
70	*Natronobacterium gregoryi SP2*	**EuA**	L0AGT8	YP_007176754.1	616	PAS+LOV+GAF+HTH	144
			L0AIG8	YP_007177421.1	1517	GAF+LOV+4PAS+2GAF+HTH	145
71	*Natronococcus amylolyticus DSM 10524*	**EuA**	L9XFR4	WP_005554108.1	618	PAS+LOV+HTH	146
	Natronococcus amylolyticus DSM 10524	**EuA**		WP_005554006.1	634	LOV+PAS+GAF+Kinase	147
72	*Natronococcus jeotgali DSM 18795*	**EuA**	L9WMX9	WP_008427349.1	619	PAS+LOV+HTH	148
73	*Natronococcus occultus SP4*	**EuA**	L0JXN1	YP_007308413.1	634	LOV+PAS+GAF+Kinase	149
			L0JTP7	YP_007308142.1	616	PAS+LOV+HTH	150
74	*Natronolimnobius innermongolicus JCM 12255*	**EuA**	L9XAN4	WP_007258756.1	655	RR+PAS+LOV+PAS+Kinase	151
			L9XE91	WP_007258481.1	645	PAS+LOV+GAF+HTH	152
75	*Natronomonas moolapensis 8.8.11*	**EuA**		YP_007486593.1	583	LOV+PAS+Kinase	153
				YP_007486271.1	673	RR+LOV+GAF+HTH	154
				YP_007487143.1	723	LOV+Kinase	155
				YP_007488415.1	652	RR+LOV+GAF+PAS	156
				YP_007487711.1	583	PAS+LOV+PAS+Kinase	157
76	*Natronomonas pharaonis DSM 2160*	**EuA**	Q3ITW5	YP_325987.1	681	RR+LOV+GAF+HTH	158
			Q3IM51	YP_327664.1	596	PAS+LOV+PAS+Kinase	159
77	*Natronorubrum bangense JCM 10635*	**EuA**	L9WG87	WP_006066370.1	646	PAS+LOV+HTH	160
				WP_006067023.1	480	LOV+PAS+Kinase	161
78	*Natronorubrum sulfidifaciens JCM 14089*	**EuA**	L9W7D2	WP_008162495.1	638	PAS+LOV+HTH	162
79	*Natronorubrum tibetense*	**EuA**		WP_006090010.1	1005	PAS+LOV+3PAS+Kinase	163
80	*Natronorubrum tibetense GA33*	**EuA**	L9W0I4	WP_006088978.1	646	PAS+LOV+HTH	164
			L9VKX1	WP_006092104.1	563	PAS+LOV+PAS+Kinase	165
81	*Salinarchaeum sp. Harcht-Bsk1*	**EuA**		YP_008054154.1	874	RR+PAS+LOV+2PAS+Kinase	166
82	*Halogeometricum borinquenseATCC 700274*	**EuA**	**E4NMD1**	YP_004036781.1	648	GAF+PAS+LOV+Kinase	167
							1
