# Supplementary material for: From Plant Infectivity to Growth Patterns: The Role of Blue-Light Sensing in the Prokaryotic World
Source: Plants (Basel). 2014 Jan 27;3(1):70–94. doi: 10.3390/plants3010070 (PMC4844311; doi:10.3390/plants3010070)
Supplement: Supplementary File 1 [file plants-03-00070-s001.zip › plants-46379-supplementary-final/Supplementary Material - S4 - legenda.pdf]

S4: a. Domain abbreviations (according to Interpro: N. J. Mulder, R. Apweiler, T. K. Attwood, A. Bairoch, D. Barrel, A. Bateman, M. Biswas, P. Bradley, P. Bucher, R. R. Copley, E. Courcelle, U. Das, R. Durbin, L. Falquet, W. Fleischmann, S. Griffiths-Jones, D. Haft, N. Harte, N. Hulo, D. Kahn, A. Kanapin, M. Krestyaninova, R. Lopez, I. Letunic, D. Lonsdale, V. Silventoinen, S. E. Orchard, M. Pagni, D. Peyruc, C. P. Ponting, J. Selengut, F. Servant, C. J. Sigrist, R. Vaughan and E. M. Zdobnov, The InterPro Database, 2003 brings increased coverage and new features., Nucl. Acids Res., 2003, 31, 315-318.

**BLUF**	Blue Light sensing Using Flavin domain
**CHASE**	Cyclases/Histidine kinases Associated Sensory Extracellular
**CHEB**	CHEB-type response regulator
**CHER**	CHER-type S-adenosylmethionine-dependent methyltransferase
**Cyclase**	Adenylyl cyclase class-3/4/guanylyl cyclase
**DICT**	Domain associated with diguanylate Cyclases and phosphodiesterases
**EAL**	Diguanylate phosphodiesterase named after conserved amino acids
**GAF**	cGMP-specific phosphodiesterases, cyanobacterial adenylate cyclases, and formate hydrogen lyase tra
**GGDEF**	Diguanylate cyclase named after conserved amino acids
**Hamp**	linker domain in Histidine kinases, Adenyl cyclases, Methyl-accepting proteins and Phosphatases
**Hpt**	histidine phosphotransfer domain
**HTH**	Helix Turn Helix/DNA binding domain
**Kinase**	Hitidine kinase domain
**LOV**	Light Oxygen and Voltage domain, PAS subfamily; here sensors of blue light
**MASE1**	Predicted integral membrane sensory domain found bacterial signaling proteins
**MCP**	Methyl-accepting chemotaxis proteins
**PAS**	Per Arnt Sim domain
**PsiE**	Phosphate-starvation-induced PsiE-like
**RR**	CheY-type Response regulator, receiver domain
**SCHIC**	Sensor containing heme instead of cobalamin
**SPOIIE**	Sporulation stage II, protein E C-terminal/Protein phosphatase 2C-related
**STAS**	Sulphate Transporter and AntiSigma factor antagonist
**TETR**	Tetracycline transcriptional regulator

S4. b. Abbreviations for phyla

F	Firmicutes
Ac	Actinobacteria
P(α)	α-proteobacteria>Caulobacterales
P(α)	α-proteobacteria>Parvularculales
P(α)	α-proteobacteria>Rhizobiales
P(α)	α-proteobacteria>Rhodobacterales
P(α)	α-proteobacteria>Rhodospirillales
P(α)	α-proteobacteria>Sphingomonadales
P(α)U	α-proteobacteria>Uncultured
P(α)poly	α-proteobacteria>Polymorphum
P(β)	β-proteobacteria>Burkholderiales
P(β)	β-proteobacteria>Gallionellales
P(β)	β-proteobacteria>Methylophilales
P(β)	β-proteobacteria>Neisseriales
P(β)	β-proteobacteria>Nitrosomonadales
P(β)	β-proteobacteria>Rhodocyclales
P(β)U	β-proteobacteria>Uncultured
P(γ)	γ-proteobacteria>Acidithiobacillales
P(γ)	γ-proteobacteria>Alteromonadales
P(γ)	γ-proteobacteria>Chromatiales
P(γ)	γ-proteobacteria> Enterobacteriales
P(γ)	γ-proteobacteria>Legionellales
P(γ)	γ-proteobacteria>Methylococcales
P(γ)	γ-proteobacteria>Oceanospirillales
P(γ)	γ-proteobacteria>Pseudomonadales
P(γ)Rei	γ-proteobacteria>Reinekea
P(γ)	γ-proteobacteria>Salinisphaerales

P(γ)	γ-proteobacteria>Thiotrichales
P(γ)U	γ-proteobacteria>Uncultured
P(γ)	γ-proteobacteria>Vibrionales
P(γ)	γ-proteobacteria>Xanthomonadales
P(δ)	δ-proteobacteria
P(ε)	ε-proteobacteria
P(Magn)	Proteobacteria>Magnetococcales
Chl	Chloroflexi
Cya	Cyanobacteria>Chroococcales
Cya	Cyanobacteria>Gloeobacteria
Cya	Cyanobacteria>Nostocales
Cya	Cyanobacteria> Oscillatoriales
Pl	Planctomycetes
Nit	Nitrospirae
Acido	Acidobacteria
Bact/Ch	Bacteroidetes/Chlorobi
DeTh	Deinococcus-Thermus
Lent	Lentisphaerae
Verr	Verrucomicrobia
Spi	Spirochaetes
EuA	Euryarchaeota
